# Switching between mono and doubly reduced odd alternant hydrocarbon: designing a redox catalyst†‡

**DOI:** 10.1039/d0sc05972b

**Published:** 2020-12-23

**Authors:** Jasimuddin Ahmed, Paramita Datta, Arpan Das, Stephy Jomy, Swadhin K. Mandal

**Affiliations:** Department of Chemical Sciences, Indian Institute of Science Education and Research-Kolkata Mohanpur-741246 India swadhin.mandal@iiserkol.ac.in; Department of Chemistry, Indian Institute of Technology Madras Chennai-600036 India

## Abstract

Since the early Hückel molecular orbital (HMO) calculations in 1950, it has been well known that the odd alternant hydrocarbon (OAH), the phenalenyl (PLY) system, can exist in three redox states: closed shell cation (12π e^−^), mono-reduced open shell neutral radical (13π e^−^) and doubly reduced closed shell anion (14π e^−^). Switching from one redox state of PLY to another leads to a slight structural change owing to its low energy of disproportionation making the electron addition or removal process facile. To date, mono-reduced PLY based radicals have been extensively studied. However, the reactivity and application of doubly reduced PLY species have not been explored so far. In this work, we report the synthesis of the doubly reduced PLY species (14π e^−^) and its application towards the development of redox catalysis *via* switching with the mono-reduced form (13π e^−^) for aryl halide activation and functionalization under transition metal free conditions without any external stimuli such as heat, light or cathodic current supply.

## Abstract 2

A doubly reduced redox non-innocent phenalenyl based transition metal free catalyst has been designed and utilized in the development of the C–C cross coupling reaction through the activation of aryl halides at room temperature.

## Graphical abstract

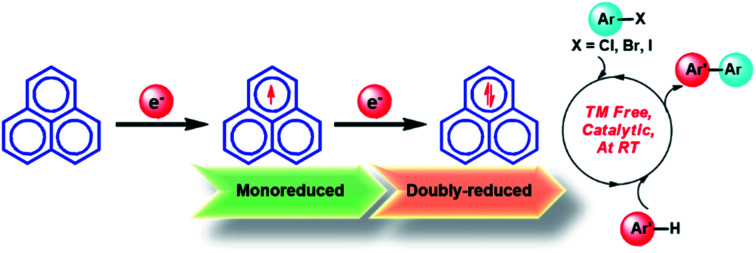

## Introduction

**Chart 1:**
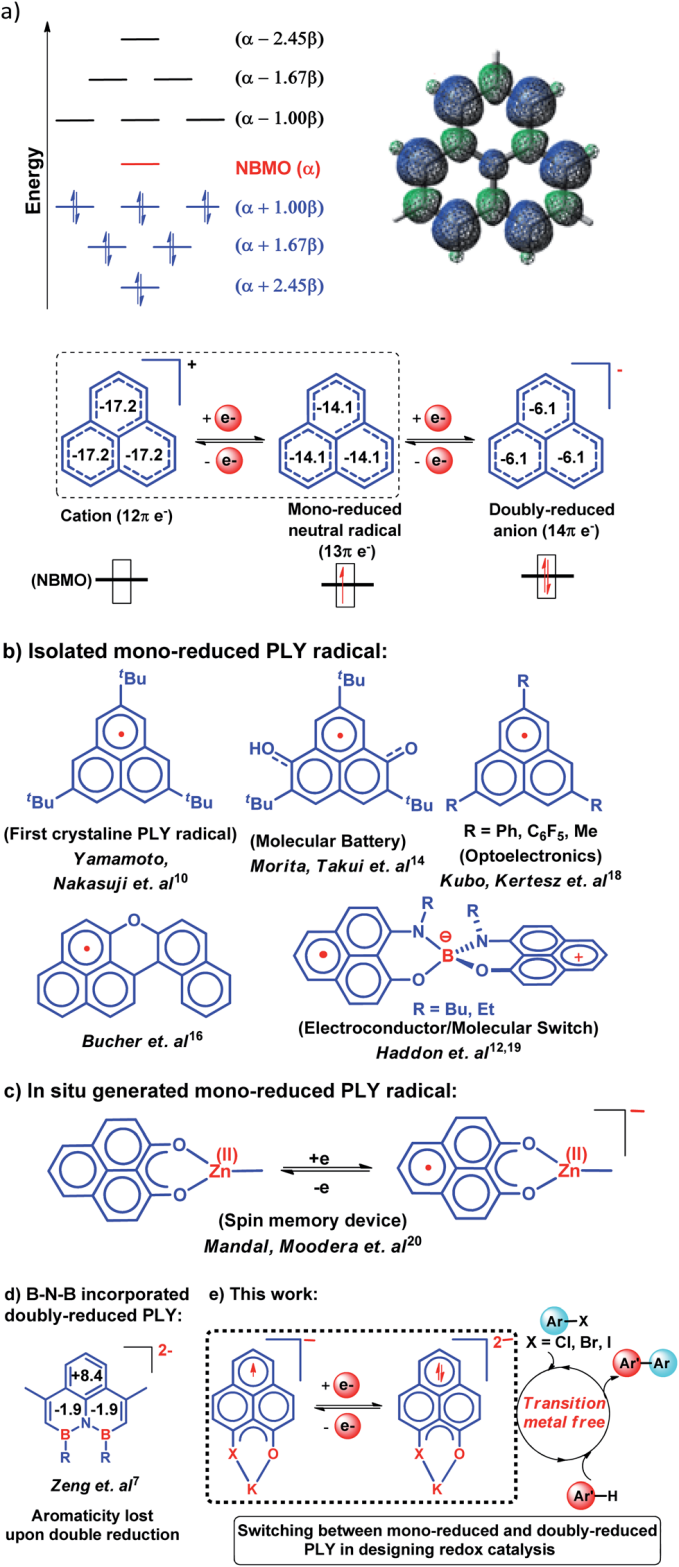
(a) HMO diagram of phenalenyl indicating the NBMO and its spin density plot along with the three redox states of phenalenyl molecules displaying their NICS values as the aromaticity index; (b) mono-reduced PLY and its various applications; (c) the *in situ* generated PLY radical and its application in a spin memory device; (d) the first isolated doubly reduced PLY by incorporation of a B–N–B fragment resulting in significant loss of aromaticity as indicated by positive or near zero NICS values. (e) This work: development of a doubly reduced PLY moiety and its switching with mono-reduced PLY for designing redox catalysts for the transition metal-free C–C cross coupling reaction under ambient conditions through activation of aryl halides.

In 1950, Hückel molecular orbital (HMO) theory suggested the existence of non-bonding molecular orbitals (NBMOs) in odd alternant hydrocarbons (OAHs) with a positive MO coefficient in the alternative carbon atoms and a nearly zero coefficient in the rest of the carbon atoms (spin density plot, Chart 1a).^1,2^ A theoretical study by Fukui *et al.* revealed that electrons occupying the NBMO are mainly responsible for the chemical reactivity of such OAHs.^3^ Phenalenyl (PLY) is a well known odd alternant hydrocarbon and its radical state was first realized in the solution state by Calvin *et al.*^4^ and Reid *et al.*,^5^ respectively, during the late 1950s. Due to the non-bonding character of the frontier molecular orbital (FMO), phenalenyl molecules can exist in three redox states, closed shell cation (12π e^−^), open shell mono-reduced neutral radical (13π e^−^) and closed shell doubly reduced anion (14π e^−^), with similar π-electron delocalization energy within the Hückel MO approximation (Chart 1a).^6^ Such a claim is also supported by recent DFT calculations based on the negative nucleus independent chemical shift (NICS) values on addition of electron(s) (Chart 1a) which indicate the preservation of aromaticity upon the sequential reduction process of the PLY moiety.^7,8^ This unique MO arrangement of phenalenyl molecules makes it a good amphoteric redox system. In 1975, Haddon drew attention to the potential of phenalenyl as an OAH in the design of organic metals and superconductors in his seminal proposal^9^ based on its mono-reduced radical state (Chart 1b). However, isolation of the mono-reduced phenalenyl radical has been challenging due to the spin distribution over the selective carbon atoms (spin density plot, Chart 1a), which readily undergo C–C sigma dimerization between two radical molecules. In 1999, Nakasuzi first isolated the mono-reduced phenalenyl radical by blocking the dimerization through steric protection (Chart 1b).^10^ Later on, a large number of PLY radicals were isolated and characterized by X-ray crystallography (Chart 1b).^11–19^ The mono-reduced phenalenyl based radicals have been used as building blocks for various intriguing materials. Such materials range from organic conductors,^11–13^ molecular batteries,^14^ and quantum spin simulators^15^ to magnetic materials displaying simultaneous bistability in multiple physical channels,^19^ realizing the elusive resonating valence bond (RVB) ground state^12,13^ once postulated by Pauling and Anderson. Later, we demonstrated that such mono-reduced phenalenyl based radicals can be generated *in situ* without isolation which plays a key role in the construction of spin memory devices (Chart 1c).^20^ Recently, such *in situ* generated mono-reduced phenalenyl based radicals were reported in designing cathode materials for single component H_2_O_2_ fuel cells^21^ and in electron transfer catalysis during various organic transformations.^22–25^ Such approaches using *in situ* generated mono-reduced phenalenyl radicals open the possibility of bridging two seemingly dissimilar areas such as spin-electronics and catalysis.^26^

Moreover, the spin distribution across the phenalenyl carbon centers and their association through π–π interaction have been subjects of intensive investigation by several theoretical and spectroscopic studies.^27–29^ The potential of mono-reduced OAHs, such as the phenalenyl radical, has been extensively discussed in various excellent documents by Morita and Takui,^30^ Hicks,^31^ Kubo^32^ and Kertesz.^33^ It may be highlighted that to date, mono-reduced phenalenyl radicals (13π e^−^ species) have been extensively studied while the doubly reduced phenalenyl species (14π e^−^) has not been characterized, though such a doubly reduced phenalenyl species has been postulated nearly six decades ago based on early Hückel MO calculations and realized by Reid and co-workers in the solution state but never isolated or utilized.^6^ In 2017, Zeng *et al.* first isolated a doubly reduced dianionic phenalenyl moiety by incorporation of a “B–N–B” fragment in the phenalenyl (PLY) backbone (Chart 1d).^7^ However, such structural modification of the parent phenalenyl ring failed to preserve its aromaticity upon double-reduction (near zero and positive NICS values, see Chart 1d). Such a significant loss in aromaticity in B–N–B–PLY upon double reduction^7^ raises concerns about its stability and hence its further application. As a result, the present literature lacks reports on the synthesis and utilization of 14π e^−^ doubly reduced phenalenyl species and their application. In this study, we have addressed such challenges by synthesizing a doubly reduced 14π e^−^ PLY species and devised a redox catalytic method for the transition metal free C–C cross coupling reaction by direct C–H arylation of arenes/heteroarenes with aryl halides under ambient conditions. In this context, a K(i) coordinated PLY moiety (12π e^−^) was reduced by one electron to generate a mono-reduced radical (13π e^−^) and by two electrons to generate a doubly reduced species (14π e^−^) consecutively without any significant compromise in its overall aromaticity (*vide infra*). Furthermore, the FMO calculations predict that two electron reduction to the phenalenyl moiety can accumulate sufficiently high energy for a single electron transfer process to the LUMO of aryl halides (*vide infra*). This understanding encouraged us to strategize a redox catalytic method using the doubly reduced OAH for the transition metal free C–C cross coupling reaction by direct C–H arylation of arenes/heteroarenes with aryl halides at room temperature under ambient conditions (without heating, light or cathodic reduction). In this regard, it may be noted that aryl halides (mainly bromides and chlorides) are considered to be the most challenging substrates to activate in the C–C cross-coupling reaction dealing with direct C–H bond functionalization of arenes/heteroarenes (Chart 1e). Accomplishing such a process without transition metals is extremely difficult and has not been achieved so far under ambient conditions (without heat, light or cathodic reduction).§§The use of potassium in the present reaction method was performed strictly inside a glove-box under a nitrogen atmosphere.^34–36^

## Results and discussion

### Isolation and characterization of the catalyst

**Scheme 1:**
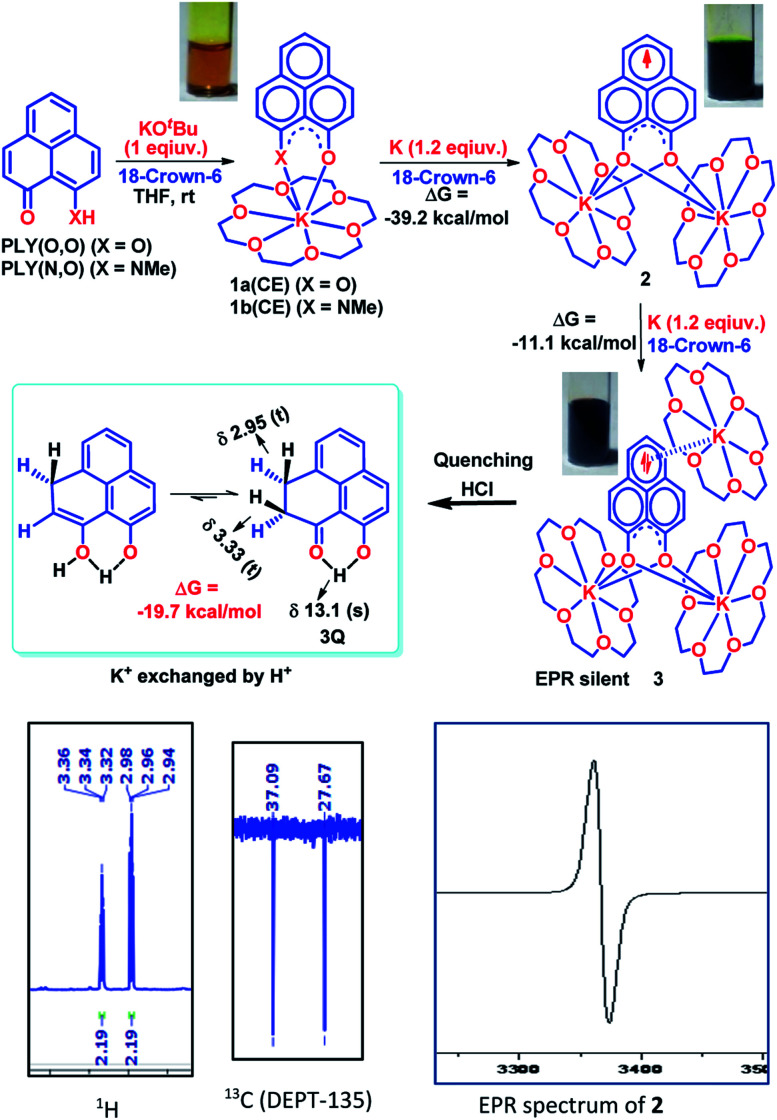
Preparation of the PLY–K complexes (1a(CE) and 1b(CE), 12π e^−^ species) and consecutive one electron reduction by K to generate mono-reduced 13π e^−^ species (2) and doubly reduced 14π e^−^ species (3), respectively; quenching the 14π e^−^ species by replacing all K^+^ ions with H^+^ ions through HCl treatment; (insets: EPR spectrum of 2 and selected part of ^1^H and ^13^C (DEPT-135) NMR spectra of 3Q).

**Fig. 1:**
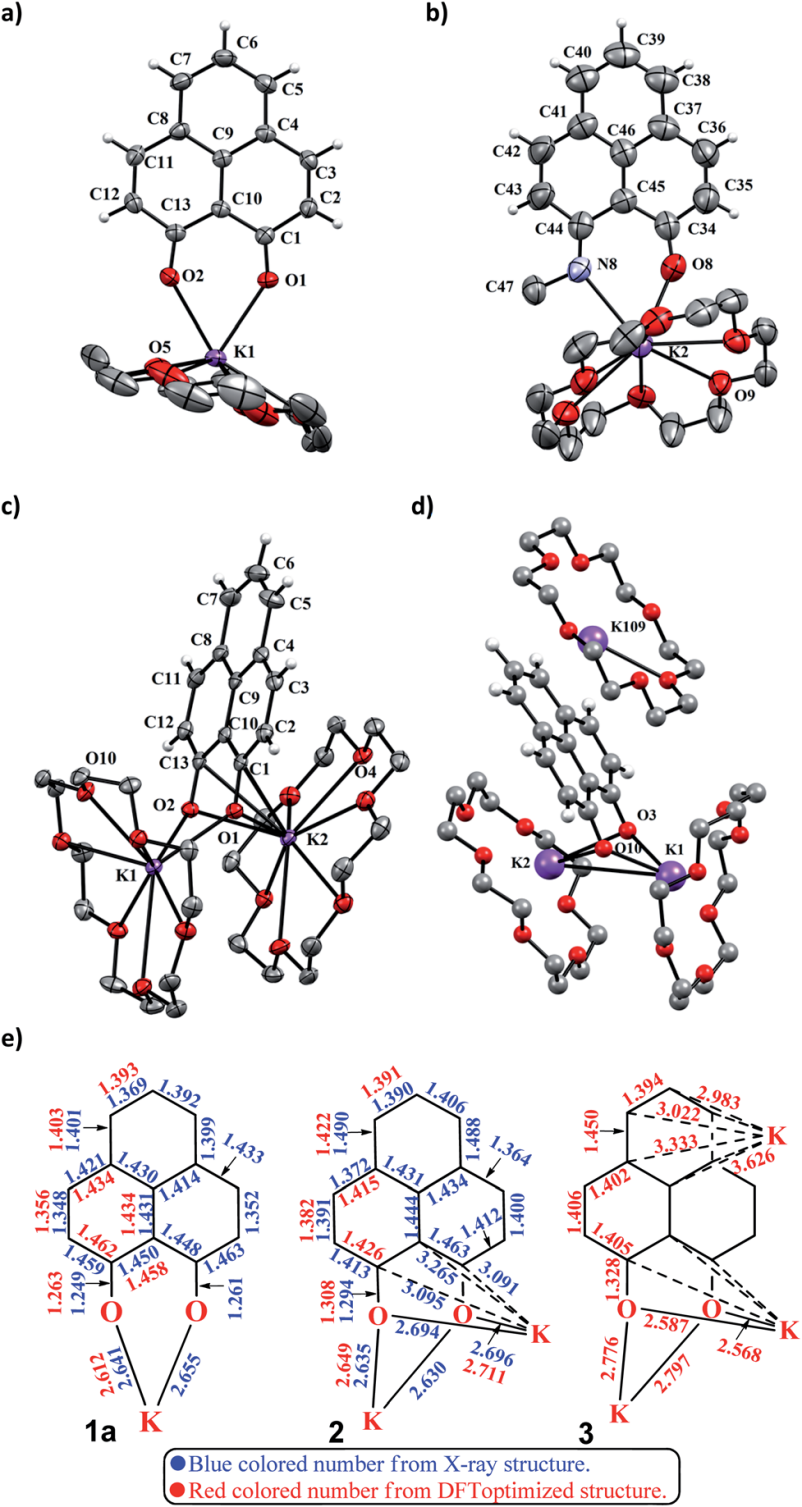
(a) X-ray crystallographic structure of 1a(CE). Ellipsoids are shown at 50% probability, selected bond lengths [Å] and bond angles [deg]: K1–O1 2.6414, K1–O2 2.6551, O1–C1 1.2613, O2–C13 1.2490, O1–K1–O2 63.2271; (b) X-ray crystallographic structure of 1b(CE). Ellipsoids are shown at 50% probability, selected bond lengths [Å] and bond angles [deg]: K2–O8 2.5976, K2–N8 2.8418, O8–C34 1.2550, N8–C44 1.2939, O8–K2–N8 60.9823, K2–O8–C34 147.4870, K2–N8–C44 139.1102; (c) X-ray crystallographic structure of 2. Ellipsoids are shown at 50% probability, selected bond lengths [Å] and bond angles [deg]: K1–O1 2.6289, K1–O2 2.6350, O1–C1 1.2943, O2–C13 1.2955, K1–O1–K2 95.9253, K1–O2–K2 95.8503; (d) DFT optimized structure of 3. A few selected bond lengths [Å] and bond angles [deg]: K1–K2 4.0573, K1–O3–K2 97.7281; (e) bond distance analysis of 1a(CE), 2 and 3 by X-ray structure and DFT study.

**Fig. 2:**
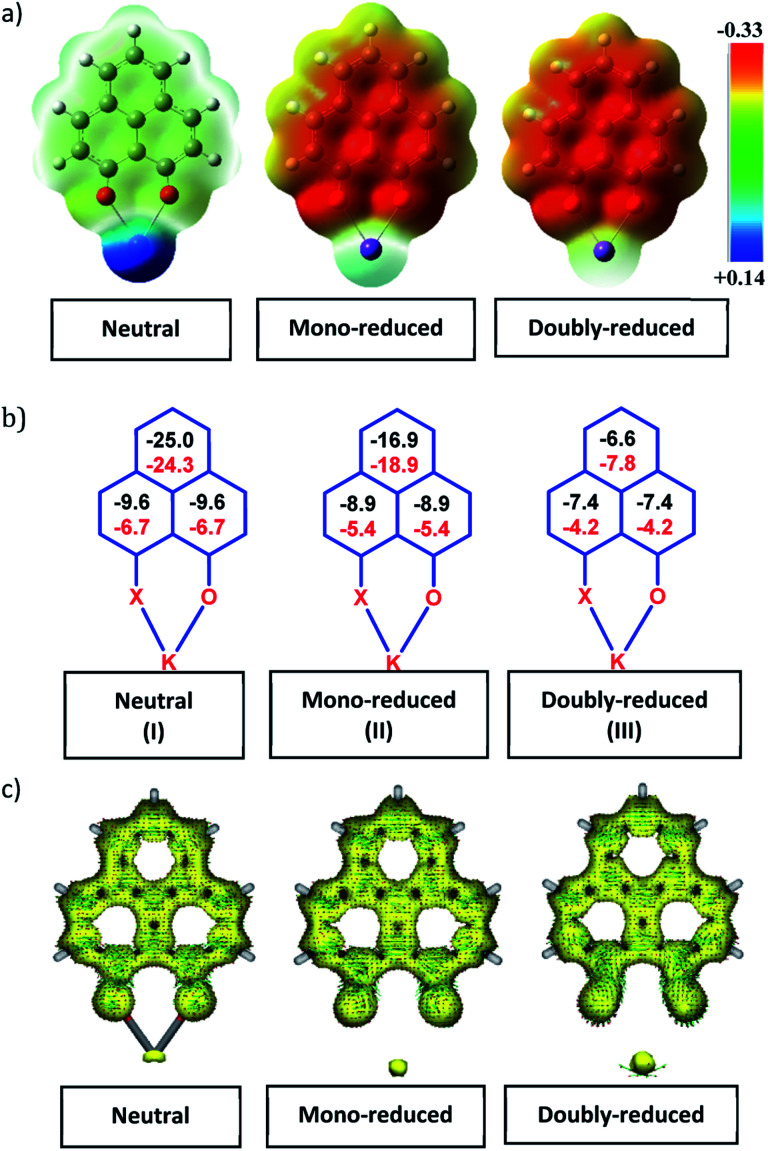
(a)Total charge density plots of the three redox states of the PLY(O,O)–K complex (Isovalue 0.002); (b) NICS values for 1a (in black) and 1b (in red) of the three different redox states; (c) anisotropy of the Induced Current Density (ACID) plots of 1a in the three different redox states.

To achieve the doubly reduced PLY species, first, the K(i) complex with 9-hydroxyphenalenone (PLY(O,OH)) was prepared (Scheme 1). Addition of 18-crown-6 helped to obtain yellow crystals of PLY(O,O)–K(18-crown-6), 1a(CE) (CE signifies the presence of crown ether), from toluene. Similarly, PLY(N,O)–K(18-crown-6), the 1b(CE) complex, was prepared using the PLY(NH,O) ligand and characterized by X-ray crystallography. Cyclic voltammetry measurements in DMF of 1a show two reduction waves at ^1^*E*_red_ −1.5 V and ^2^*E*_red_ −1.95 V (*vs.* Ag/AgCl, see the ESI, Fig. S1‡). Chemical reduction of 1a(CE) with 1.2 equivalents of K in THF resulted in a green solution which shows a strong EPR signal with *g* = 2.0001 (Scheme 1, inset), indicating formation of a mono-reduced anionic 13π electron phenalenyl based radical, 2. The green crystals of 2 were grown from a mixture of THF (1.2 mL) and toluene (200 μL) at −35 °C inside an argon filled glovebox. The X-ray structure established the molecular structure of 2 (Fig. 1c). Further treatment of complex 2 with another equivalent of K in THF resulted in a dark brown solution (Scheme 1). This brown reaction mixture shows NMR activity and EPR inactivity. The EPR silencing is indicative of an integer spin as anticipated for a doubly reduced PLY species, also an observation explicitly noted earlier.^11^ These observations clearly suggest the formation of a doubly reduced diamagnetic 14π e^−^ phenalenyl species. Despite multiple trials, we failed to obtain the crystals of this doubly reduced species even in the presence of an additional equivalent of 18-crown-6 to trap all K(i) ions present in 3. The structures of all these three redox species (containing 12π e^−^, 13π e^−^ and 14π e^−^ in the PLY moieties) were optimized by the DFT method and the optimized structure of 3 is shown in Fig. 1d along with the X-ray structures of 1a(CE), 1b(CE) and 2 (Fig. 1a–c). Further, to trap the doubly reduced PLY species all K^+^ ions were replaced with H^+^ ions upon treating the brown solution of 3 with aq. HCl (800 μL of 35% HCl solution in water) which resulted in a colorless solid displaying two triplet resonances in the aliphatic region at *δ* 2.95 and *δ* 3.33 ppm in the ^1^H NMR spectrum (see the inset of Scheme 1: selected part of the ^1^H NMR spectrum of 3Q) each having a two proton intensity. Such an unusual shift of aromatic protons indicates dearomatization of the PLY ring. With the help of mass, ^1^H, ^13^C and DEPT-135 spectra, it was revealed to be the quenched species 3Q (inset of Scheme 1) in which three K^+^ ions of 3 are replaced by three H^+^ ions (in black). Furthermore, this observation of PLY ring dearomatization clearly indicates that the (–CH=CH–) alkene fragment of PLY transforms to an alkane (–CH_2_–CH_2_–) fragment upon two electron reduction and addition of 2H^+^ (from HCl). It may be noted that hydrogen atom abstraction by the mono-reduced B–N–B embedded PLY radical has been reported by Wagner and coworkers very recently^37^ and the dearomatization of the PLY radical by abstraction of a hydride (H^+^ + 2e^−^) has been demonstrated earlier.^24^ Such a conclusion was also supported by our isotope labelling experiment with DCl and by characterizing the deuterium incorporated product (see the ESI, Fig. S17–S21‡). Additionally, from the bond distance analyses of 1a(CE) and 2 and 3 (Fig. 1e), it may be noted that the alternative C–C bonds of the phenalenyl molecule become elongated and shortened gradually upon mono- to double-electron reduction (from 1a(CE) to 2 to 3). This conclusion is fully consistent with the total electron density plots of the three redox states of the PLY(O,O)–K compound (1a) which show that upon consecutive reduction, the negative charge gradually accumulates over the PLY moiety (Fig. 2a). Further, the Nucleus Independent Chemical Shift (NICS) values of these complexes were calculated to check the effect of successive addition of electrons on the aromaticity of the PLY ring for both 1a and 1b (Fig. 2b). The negative NICS values of all three redox states of 1a and 1b indicate that PLY moieties upon consecutive addition of electrons preserve their aromaticity (Fig. 2b). Anisotropy of the Induced Current Density (AICD) plots following the method developed by Herges and coworkers^38,39^ also authenticate this fact of retaining aromaticity over gradual addition of electrons transforming 12π e^−^ to 13π e^−^ to 14π e^−^ PLY species (Fig. 2c). These studies confirm that the consecutive reductions in the PLY moiety can retain its aromaticity and its anticipated stability which was also predicted by recent calculation of the different redox states of pristine PLY.^7,8^ Such an observation made us curious about utilizing such doubly reduced PLY species in catalytic organic transformation.

### Designing a redox catalyst

**Fig. 3:**
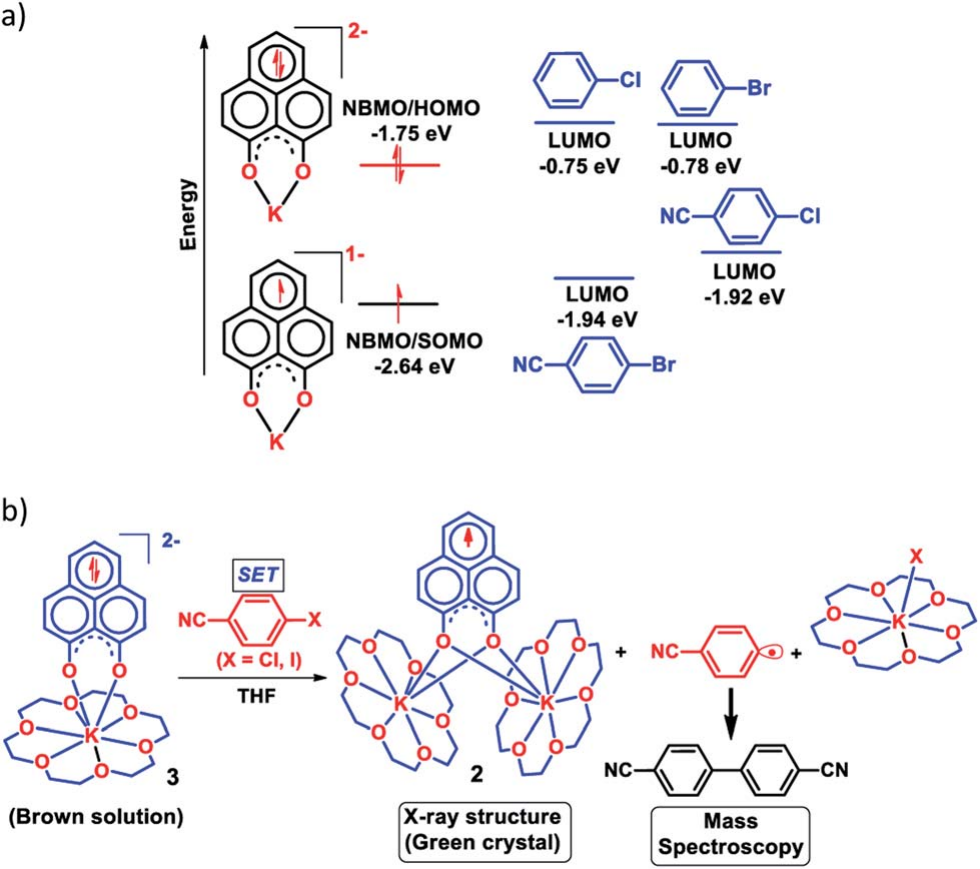
(a) Theoretical study for FMO energy calculation of various aryl halides and the catalyst was performed with B3LYP/6-31g+(d,p) in DMF (CPCM); (b) stoichiometric reaction between doubly reduced 3 (14π e^−^ species) and 4-chlorobenzonitrile resulting in 2 (13π e^−^ species) after single electron transfer, bicyanophenyl and KX indicating C–X bond splitting.

Next, we became interested if such doubly reduced PLY species can be used in designing a redox catalyst aimed at activating aryl halides. Previously, the mono-reduced PLY based radicals were used to design a catalytic protocol for direct C–H arylation of arenes and heteroarenes with an aryl diazonium salt as the coupling partner^25^ but such mono-reduced PLY radicals failed to perform the single electron transfer (SET) process on aryl halides to generate aryl radicals. Its worth noting that the activation of aryl halides and their functionalization with arenes or heteroarenes' C–H bond is usually carried out using transition metals such as Ru, Pd, Fe, Ni, Cu, *etc.*^40,41^ There has been a constant necessity to replace the transition metals in such processes and as an alternative, the base promoted homolytic aromatic substitution (BHAS) reaction^42–46^ was developed using excess base and an organic ligand in semi-stoichiometric loading which acts as a radical initiator; however, the reaction works only at high temperature (>120 °C). In an elegant approach, in 2014, König and coworkers established a new methodology avoiding metals which combined chemical and photochemical methods to achieve a reduction potential high enough to split the aryl halides.^34^ Following this principle developed by König, very recently, two other reports appeared for aryl halide activation by double excitation of which the first excitation was electrochemical while the second one was accomplished by photochemical excitation.^35,36^ However, in all these methods, the catalyst was required to be regenerated at the end of the catalytic cycle by a constant supply of electrons either photochemically or electrochemically and hence they neither work under ambient conditions nor can they be considered truly catalytic (as a disposable/external source of electrons is required to regenerate the catalyst). In this regard, we explored the possibility of the doubly reduced PLY species for activation of aryl halides under ambient conditions. To check whether such a doubly reduced PLY species is capable of transferring its electron to aryl halides, at first a DFT calculation was performed. A preliminary theoretical calculation indicates that two electron reduction to the phenalenyl moiety can indeed generate sufficiently high energy electrons in its HOMO for transfer to the LUMO of aryl halides (Fig. 3a). From this calculation, it may be noted that the substrates with LUMO energy in between the SOMO (−2.64 eV) of mono-reduced PLY (13π e^−^ species) and HOMO (−1.75 eV) energy of doubly reduced PLY species (14π e^−^ species) can be activated. This calculation predicts that an electron transfer process is thermodynamically feasible from the doubly reduced PLY species to the LUMO of 4-chlorobenzonitrile (Fig. 3a). To confirm such an electron transfer process as predicted by DFT calculations, at first, a control experiment was carried out, when the brown colored double reduced compound 3 (14π e^−^ species) was treated with 4-chlorobenzonitrile in a 1 : 1 ratio in THF, which showed an immediate color change from brown to green at room temperature.

The green solution next produced deep green crystals of 2 at −35 °C (also confirmed by the X-ray measurements). The formation of 2 (13π e^−^ species) confirms the single electron transfer from 3 (14π e^−^ species) to 4-chlorobenzonitrile. On accepting the electron, 4-chlorobenzonitrile splits into the cyanophenyl radical and Cl^−^ ion. Two such cyanophenyl radicals couple into the corresponding biaryl species (Fig. 3b) as detected by mass spectrometry. Furthermore, formation of KX was confirmed by the X-ray structure of KI trapped with 18-crown-6 when a similar study was carried out with 4-iodoanisole (Fig. 3b). All these observations clearly establish a single electron transfer (SET) from doubly reduced 3 to accomplish (aryl)C–X bond splitting under ambient conditions. This understanding prompted us to devise a catalytic method for the transition metal free C–C cross coupling reaction by direct C–H arylation of arenes/heteroarenes with aryl halides at room temperature under ambient conditions (without heating, light or cathodic reduction).

### Catalytic activity

**Scheme 2:**
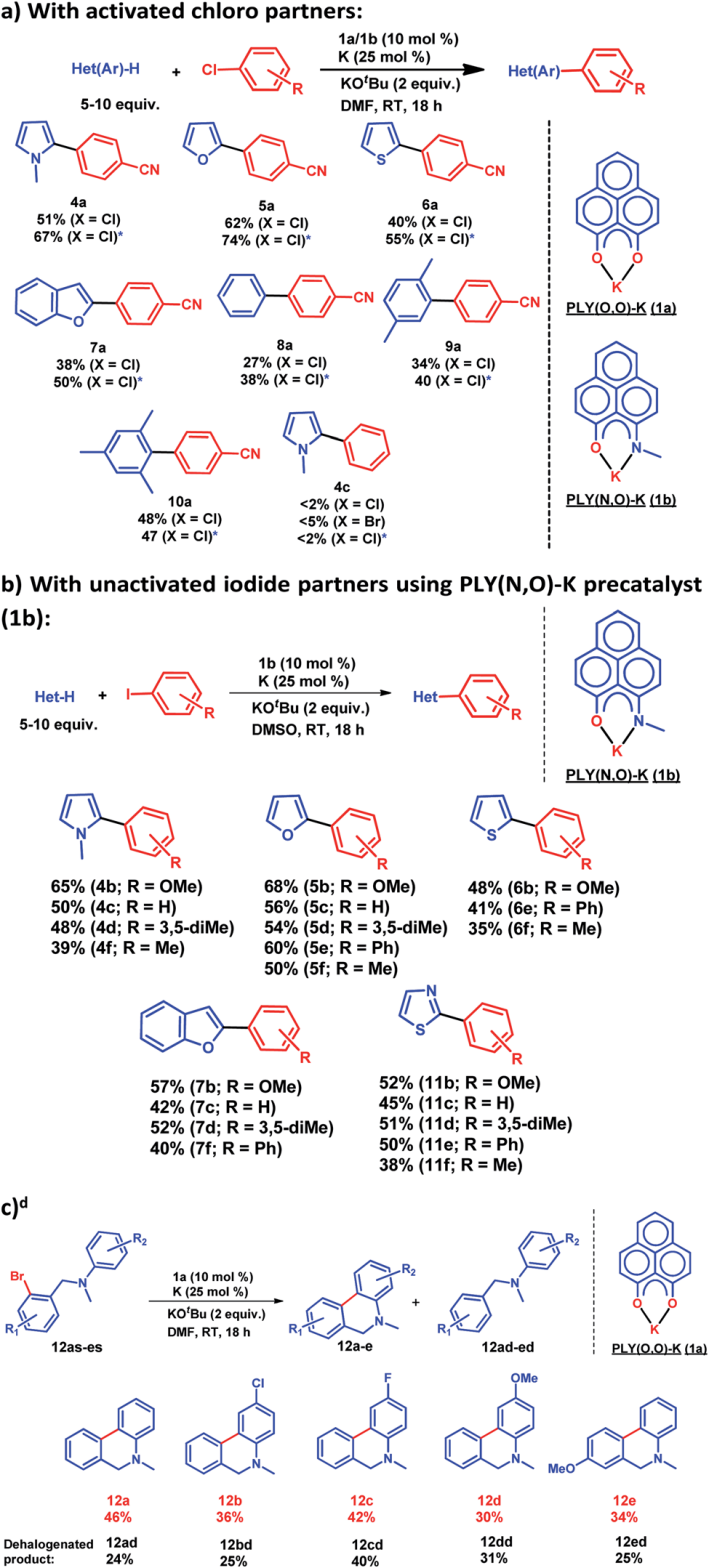
Catalytic activation of aryl halide at room temperature: (a) direct C–H arylation with activated aryl chloride, * catalysis performed with 1b as a precatalyst; (b) direct C–H arylation with unactivated aryl iodide using 1b as a precatalyst; ^*d*^ yield determined by NMR conversion; (c) intramolecular coupling by aryl bromide activation using 1a as a precatalyst.

After this preliminary understanding, the catalytic activity of such a doubly reduced PLY species was checked in direct C–H arylation of arenes and heteroarenes with aryl halides at room temperature. Delightfully, after optimization of various reaction parameters (see the ESI, Table S6‡), it was found that 10 mol% 1a and 25 mol% K in the presence of 2 equivalents KO^*t*^Bu in DMF deliver 51% yield for direct C–H arylation of *N*-methyl pyrrole with 4-chlorobenzonitrile along with ∼10% dehalogenated product. Changing to 1b in place of 1a under the optimized conditions improved the isolated yield further to 67%. A sub-stoichiometric (only 0.25 equiv.) amount of the reducing agent (K) is enough to accomplish such a process and thus continuous supply of electrons is not required unlike earlier approaches (constant supply of electrons by the photochemical or cathodic reduction method).^34–36^ Moreover, this method works at ambient temperature which sharply contrasts with the earlier reported BHAS reaction procedures^42–46^ requiring high temperature (usually above 100 °C). This protocol was further tested with various heteroarenes and arenes, which showed moderate to good reactivity in forming products 4a–10a (up to 74% isolated yield). It may be highlighted that this methodology is successful for the arylation of unactivated arenes such as benzene, xylene and mesitylene along with various heteroarenes (Scheme 2) and was not accomplished in previous transition metal free processes under ambient conditions.^34–36^ However this protocol was successful with activated aryl chloride and bromide substrates but it failed for unactivated aryl chlorides or bromides (Scheme 2a). Moreover, it works successfully with unactivated aryl iodides. A series of unactivated aryl iodide substrates were subjected to such direct C–H arylation of heteroarenes under ambient conditions resulting in moderate to very good yields of desired products (Scheme 2b; 35–65% yields; 4b–f, 5b–f, 6b,e,f, 7b–e, 11b–f). Next, this method was utilized for the preparation of various alkaloid cores by intramolecular aryl bromide bond activation in moderate yields (up to 46% yield) of C–C coupled products 12a–e (Scheme 2c) along with the formation of dehalogenated products (24–40%, 12ad–ed). With this catalytic result, we could establish that this double reduction process with phenalenyl based OAHs is an efficient approach to activate and functionalize aryl halides which gives the opportunity of tuning the catalyst with the help of ligand backbone modifications. The yields of the biarylated product and broad substrate scopes which include the C–H arylation of various heteroarenes as well as the arene partners, make this protocol attractive over the previous transition metal-free methods.^34–36^

### Mechanistic investigation

**Fig. 4:**
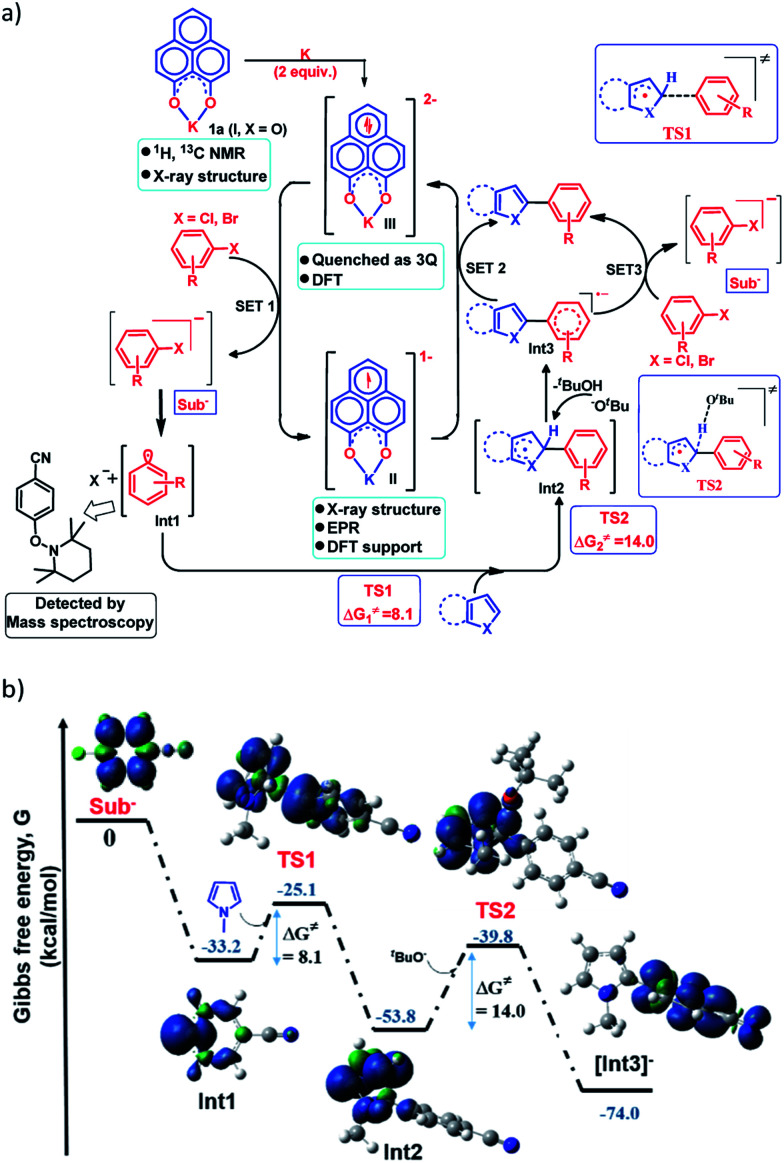
(a) Proposed mechanistic cycle for the C–H arylation reaction by aryl halide activation; (b) reaction pathway by DFT calculations along with the geometry and spin density plot (α-spin in blue and β-spin in green color) of the reactive intermediates and transition states.

Next, to establish the mechanism of this transition metal free C–H arylation reaction activating aryl halides, a combined approach consisting of DFT calculations and the results from a series of control experiments was employed. Based on this, a plausible mechanistic scheme is proposed in Fig. 4a. At first, the experiment depicted in Fig. 3b suggests that the doubly reduced species (III) can transfer an electron to the aryl halide generating Sub^−^ followed by an aryl radical and transforms into the mono-reduced species (II) giving a preliminary idea of a single electron transfer (SET) process as the reaction initiation step (Fig. 4a). Next, a full mechanistic cycle has been worked out using theoretical calculations considering (wb97xd/6-31g(d) level of theory) the anionic aryl halide (Sub^−^) as the starting point, which was produced after SET from the doubly reduced PLY(III) to aryl halide (Fig. 4b). The anionic aryl halide (Sub^−^) splits into an aryl radical (Int1) and a halide anion, which is an exothermic process. The aryl radical was characterized by its trapping with the free radical TEMPO. This aryl radical attacks the C2 center of the *N*-methyl pyrrole coupling partner *via* transition state TS1 (Δ*G*^≠^ = 8.1 kcal mol^−1^) to generate the intermediate Int2. The reaction energetics calculation for this particular step corresponding to TS1 with five different aryl radicals shows a linear correlation between the exothermicity (Δ*G*^0^) and the transition state energy barrier (Δ*G*^≠^) of the reaction (see the ESI, Fig. S100 and S101‡). This observation indicates a net charge transfer between the aryl radical and the arene π-electron cloud.^47^ Next, Int2 undergoes a proton abstraction by the O^*t*^Bu anion *via*TS2 with a transition state energy of 14.0 kcal mol^−1^, which is accessible at room temperature. This proton abstraction results in the formation of an anionic product intermediate (Int3) which can undergo a favorable electron transfer to the mono-reduced PLY–K complex (II) to regenerate the active catalyst (doubly reduced PLY–K complex, III) or it can initiate another set of reactions by transferring one electron to the aryl halide directly and delivers the cross-coupled product (radical chain propagation, *vide infra*).

### Nelsen's four point calculation for electron transfer energetics

**Fig. 5:**
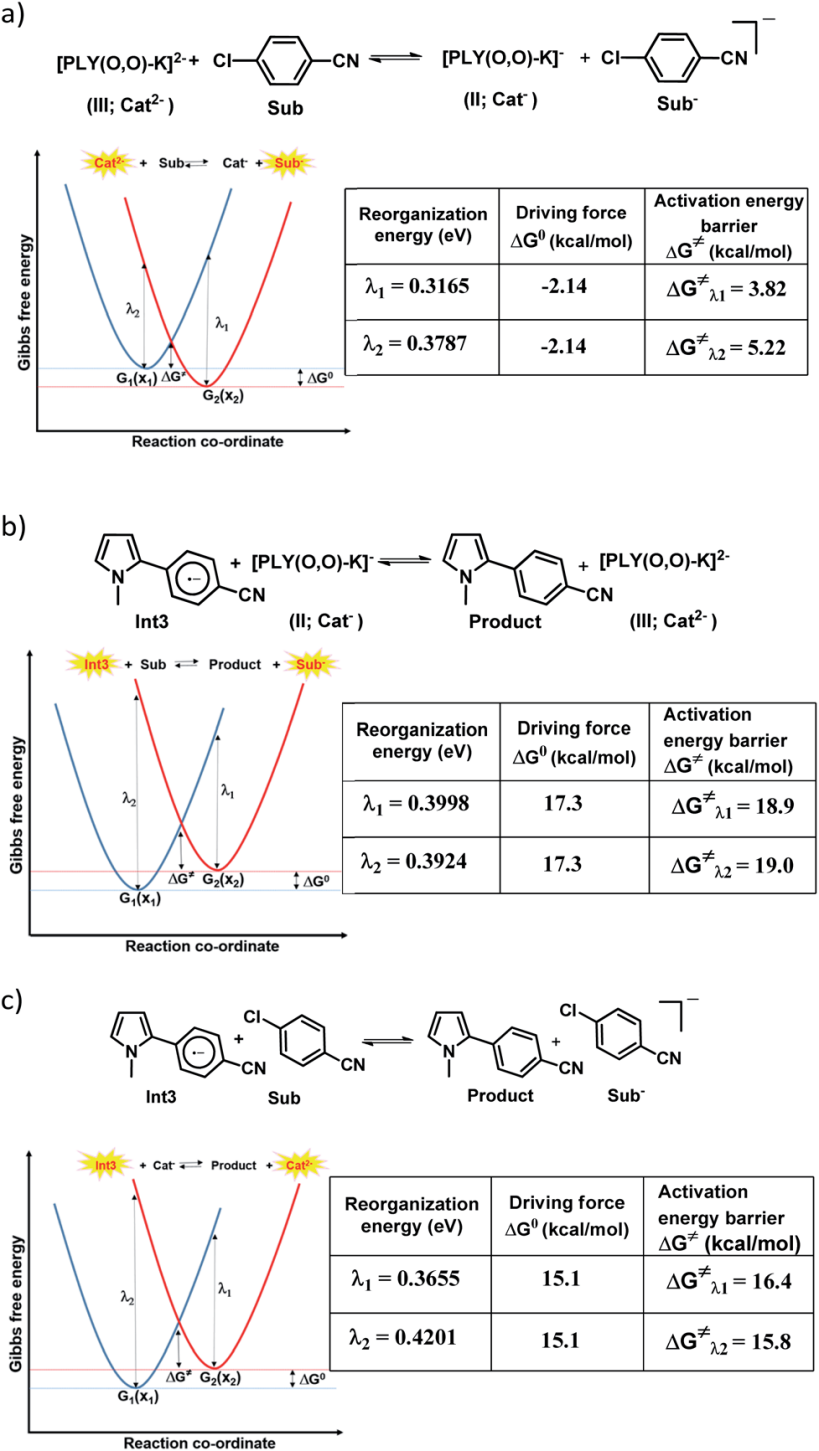
Parabolic PESs for reactants (blue) and products (red) in an electron self-exchange process as a function of the reaction coordinate along with the theoretically calculated reorganization energies (*λ*), reaction driving force (Δ*G*^0^) and the activation energy barrier (Δ*G*^≠^); (a) the electron transfer from the doubly reduced PLY moiety to aryl halides; (b) catalyst regeneration at the final step of product formation; (c) the radical chain propagation process.

However, the straightforward reaction path calculation (Fig. 4b) with the help of transition state realization did not enable us to understand the electron transfer energetics. In this regard, we took advantage of Nelsen's four-point calculation based Marcus–Hush theory of electron transfer. This method has been considered a well accepted tool to understand electron transfer kinetics.^48–50^ Such a calculation considers the parabolic potential energy surface (PES) for both the reactants and products and the electron transfer occurs only when the reactants and products have the same energy as per the Franck–Condon approximation. The full calculation method is described in the Experimental section and the results are presented in Fig. 5. For the reaction initiation step which involves electron transfer from doubly reduced PLY (III; Cat^2−^) to the 4-chlorobenzonitrile partner (SET1), the four-point calculation shows that the activation energy 
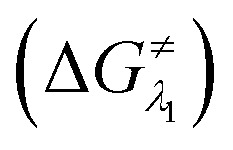
 for the forward reaction is 3.82 kcal mol^−1^ and that for the backward reaction is 
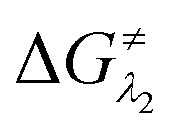
 = 5.22 kcal mol^−1^ which are accessible at room temperature (Fig. 5a). In the catalyst regeneration step where the product like anionic species (Int3) transfers electrons to the mono-reduced PLY(O,O)–K complex (II; Cat^−^) to regenerate the catalytically active doubly reduced PLY(O,O)–K species III (*via* SET2), the activation energy barriers are 18.9 and 19.0 kcal mol^−1^ for the forward and backward reactions, respectively, which are again achievable at room temperature (Fig. 5b). Next, we considered the possibility of radical chain propagation by transferring the electron to another set of substrates (4-chlorobenzonitrile) from Int3 (SET3). The activation energy barriers (15.4 and 16.8 kcal mol^−1^ for the forward and backward reactions, respectively) are almost comparable with those for the catalyst regeneration step, which indicates the thermodynamic feasibility of the radical chain propagation process (Fig. 5c). However, a series of blank reactions without using the catalyst in the presence of only 0.25 equiv. of K and 2 equivalents of KO^*t*^Bu resulted in up to 30% yield of the biarylated products (4a, 5a, 7a, and 8a; see the ESI, Pages S47–S48‡) which reflects the inefficiency of the radical chain propagation process alone to deliver the observed yields. For example, the presence of 0.1 equiv. catalyst improves the yield significantly up to 74%. The inefficiency of the radical chain propagation process was also evident in the C–H functionalization reaction with aryl halides using a continuous source of electrons by cathodic or photochemical reduction.^34–36^ Further, when the precatalyst was changed from 1a to 1b, it was anticipated that the substitution of electronegative O by N atoms on the spin bearing carbon on PLY would result in increasing energy of the HOMO and it was calculated to be −1.63 eV whereas for the O,O substituted PLY–K complex the HOMO energy was −1.75 eV after its double reduction. Such a higher energy HOMO in doubly reduced PLY(N,O)–K (1b) in fact offered overall 10–15% improved yield (up to 74% yield; starred entries, Scheme 2a) as compared to the precatalyst 1a for direct C–H arylation of arenes and heteroarenes. Such catalytic outcome influenced by the nature of the pre-catalyst clearly suggests its dependence on catalysts' orbital energy housing the injected electrons.

## Conclusions

The doubly reduced PLY species was theoretically predicted nearly six decades ago; however, it has never been isolated or utilized. Herein we demonstrate that switching between the mono and doubly reduced phenalenyl species can be a basis to design a new redox catalytic method. The doubly reduced species was utilized in the catalytic activation of aryl halides without using any transition metal under ambient conditions. Further, tuning of the energy of the orbital housing the doubly reduced electron as observed for the N,O-PLY based precatalyst opens the possibility of expanding the scope of such a catalytic concept.

## Experimental section

### General consideration

All solvents used in the experiments were distilled from calcium hydride (for DMF and DMSO) or sodium/benzophenone (for THF and toluene) under inert conditions prior to use. All chemicals were purchased from commercial sources and used as received. The ^1^H, ^13^C NMR spectra were recorded on 400 and 500 MHz spectrometers in CDCl_3_ with a residual undeuterated solvent (CDCl_3_, 7.26/77.0) as an internal standard. Chemical shifts (*δ*) are given in ppm, and *J* values are given in Hz. All chemical shifts were reported in ppm using tetramethylsilane as a reference. Chemical shifts (*δ*) downfield from the reference standard were assigned positive values. Column chromatography including thin-layer chromatography (TLC) was performed on silica gel (Merck silica gel 100–200 mesh). Evaporation of solvents was performed under reduced pressure using a rotary evaporator. High-resolution mass spectra (HRMS) were obtained on a Bruker maXis impact. ESI-mass spectra were recorded on a Waters Micro-MS mass spectrometer. EPR spectroscopic measurements were performed on a Bruker (X-band) spectrometer. All the glassware and NMR tubes used for the experiments were kept in an oven at 120 °C overnight (12 h). X-ray crystallographic measurements were performed on an Agilent X-ray diffractometer.

### Synthesis of the PLY(O,O)–K complex (1a and 1a(CE))

Inside a nitrogen filled glovebox, 0.1 mmol (20 mg) PLY(OH,O), 0.1 mmol (12 mg) KO^*t*^Bu and 0.12 mmol (25 mg) 18-crown-6 were mixed together with 1.2 mL toluene in a glass vial. The reaction mixture was stirred at room temperature for 10 min. The clear reddish solution was transferred to another vial and kept for crystallization at −35 °C along with 0.5 mL toluene as a co-solvent. Rod shaped crystals were grown on allowing to stand overnight. A suitable crystal was coated with precooled inert oil and was analyzed by X-ray crystallography. 1a was prepared following the same procedure without adding 18-crown-6 and it was collected as a precipitate from THF.

### Synthesis of the PLY(N,O)–K complex (1b and 1b(CE))

Inside a nitrogen filled glovebox, 0.1 mmol (20 mg) PLY(NH,O), 0.1 mmol (2 mg) KH and 0.12 mmol (25 mg) 18-crown-6 were mixed together with 1.2 mL toluene in a glass vial. The reaction mixture was stirred at room temperature for 10 min. The clear red solution was transferred to another vial and kept for crystallization at −35 °C. Rod shaped crystals were grown on allowing to stand overnight. A suitable crystal was coated with precooled inert oil and was analyzed by X-ray crystallography. 1b was prepared following the same procedure without adding 18-crown-6 and it was collected as a precipitate from THF.

### Synthesis of the PLY(O,O)–K mono-reduced complex (2)

Inside a nitrogen filled glovebox, 0.1 mmol (20 mg) PLY(OH,O), 0.1 mmol (12 mg) KO^*t*^Bu and 0.12 mmol (25 mg) 18-crown-6 were mixed together with 1.2 mL THF in a glass vial. 0.12 mmol (5 mg) K and 0.12 mmol (25 mg) 18-crown-6 were added to the reaction mixture. The final reaction mixture was stirred for 10 min and the red colored solution slowly turned into a green colored solution. The clear green solution was transferred to another vial and kept for crystallization at −35 °C along with 0.2 mL toluene as a co-solvent. Deep green hexagonal shaped crystals were grown on allowing to stand overnight. A suitable crystal was coated with precooled inert oil and was analyzed by X-ray crystallography.

### Preparation of the doubly reduced PLY(O,O)–K species (3)

Inside a nitrogen filled glovebox, 0.1 mmol (20 mg) PLY(OH,O), 0.1 mmol (12 mg) KO^*t*^Bu and 0.12 mmol (25 mg) 18-crown-6 were mixed together with 2 mL THF in a glass vial. 0.24 mmol (12 mg) K and 0.24 mmol (50 mg) 18-crown-6 were added to the reaction mixture. The final reaction mixture was stirred at room temperature. After 10 min, the red colored solution slowly turned into a green colored solution and after another 15 min, this green color slowly turned into dark brown. The doubly reduced PLY species was trapped by HCl treatment (see below) and also optimized by DFT calculation.

### Isolation of the quenched doubly reduced PLY(O,O)–K species (3Q)

Inside a nitrogen filled glovebox, 0.1 mmol (20 mg) PLY(OH,O), 0.1 mmol (12 mg) KO^*t*^Bu and 0.12 mmol (25 mg) 18-crown-6 were mixed together with 2 mL THF in a glass vial. 0.24 mmol (12 mg) K and 0.24 mmol (50 mg) 18-crown-6 were added to the reaction mixture. The final reaction mixture was stirred at room temperature. After 10 min, the red colored solution slowly turned into a green colored solution and after another 15 min, this green color slowly turned into a dark brown solution. Next, 0.8 mL aq. HCl solution (35%) was added into the brown solution. The organic compound was extracted in ethyl acetate from water, and after solvent evaporation the organic compound was dried. The product was isolated by column chromatography using hexane as the eluent over silica. The isolated product was characterized by mass and NMR spectroscopic measurements.

### General procedure for intermolecular coupling reactions

1a/1b (I) (0.024 mmol) and K (0.06 mmol) were taken in 1.2 mL DMF/DMSO in a 25 mL pressure tube. This mixture was stirred at room temperature for 30 min. Substrates (0.24 mmol) and KO^*t*^Bu (0.48 mmol) were added to the resulting solution of the catalyst inside a nitrogen filled glovebox. The final reaction mixture was stirred for an appropriate amount of time at room temperature. After completion of the reaction, the products were extracted in 25 mL ethyl acetate and dried over anhydrous sodium sulphate. The solvent was removed under reduced pressure to obtain the crude products. The biarylated products were isolated by column chromatography over silica gel using a hexane and ethyl acetate mixture solvent as the eluent.

### General procedure for intramolecular coupling reactions

1a/1b (I) (0.024 mmol) and K (0.06 mmol) were taken in 1.2 mL DMF in a 25 mL pressure tube. This mixture was stirred at room temperature for 30 min. Substrates (0.24 mmol) and KO^*t*^Bu (0.48 mmol) were added to the resulting solution of the catalyst inside a nitrogen filled glovebox. The final reaction mixture was stirred for an appropriate amount of time at room temperature. After completion of the reaction, the products were extracted in 25 mL ethyl acetate and dried over anhydrous sodium sulphate. The solvent was removed under reduced pressure to obtain the crude products. NMR (^1^H and ^13^C) spectroscopic measurements of all the reaction mixtures were carried out to characterize the products and the conversion was calculated from the intensity of ^1^H NMR peaks.

### Computational details

Theoretical calculations were performed with the Gaussian16 program suite.^51^ All theoretical calculations were carried out using the density functional theory (DFT) method with Becke's three-parameter hybrid exchange functional and the Lee–Yang–Parr correlation functional (B3LYP) employing the 6-31G(d) basis set^52,53^ for all atoms. Anisotropy of the Induced Current Density (ACID) plots (B3LYP/6-311g(d,p)) were calculated by using the method developed by Herges and only π-orbitals were considered. CSGT NMR calculations were performed for these ACID plots.^38,39^ The plots were generated using AICD-3.0.2 version with a threshold vector of 1.5 Å and an isovalue of 0.04. Nucleus Independent Chemical Shift (NICS) values were calculated (B3LYP/6-311G(d,p)) using the standard GIAO procedure.^54^ The CPCM solvent model was used in these calculations.^53^

### Nelsen's four-point calculation using Marcus–Hush theory:

The electron transfer energetics calculation has been carried out by Nelsen's four-point calculation based on Marcus–Hush theory of electron transfer.^55–58^ Gibbs free energy (*G* in Hartrees) of geometries for the anion optimized structures (Opt) and the neutral single point (SP) computations at the geometries obtained after optimization at the wB97XD/6-31+(d)^53,59^ theory level have been calculated. For example, the donor and acceptor pair D–A has been considered to explain the theoretical model. *λ*_1_ and *λ*_2_ represent the reorganization energies of the forward and backward reactions, respectively.

*G*(D^−^): Gibbs free energy of the anionic donor.

*G*(A): Gibbs free energy of the neutral acceptor.

*G*(Dsp): Gibbs free energy of the neutral donor in the geometry of the anionic donor.

*G*(D^−^sp): Gibbs free energy of the anionic donor in the geometry of the neutral donor.

*G*(A^−^sp): Gibbs free energy of the anionic acceptor in the geometry of the neutral acceptor.

*G*(Asp): Gibbs free energy of the neutral acceptor in the geometry of the anionic acceptor.

1 *G*_1_(*x*_1_) = *G*(D^−^) + *G*(A)

2 *G*_2_(*x*_2_) = *G*(D) + *G*(A^−^)

3 *G*_2_(*x*_1_) = *G*(Dsp) + *G*(A^−^sp)

4 *G*_1_(*x*_2_) = *G*(Dsp) + *G*(A^−^sp)

Driving energy calculation:

5 Δ*G*^0^ = *G*_2_(*x*_2_) − *G*_1_(*x*_1_)

Reorganization energy calculation:

6 *G*_2_(*x*_1_) − *G*_1_(*x*_1_) = *λ*_1_ + Δ*G*^0^

7 *G*_1_(*x*_2_) − *G*_2_(*x*_2_) = *λ*_2_ + Δ*G*^0^

Activation energy calculation:8
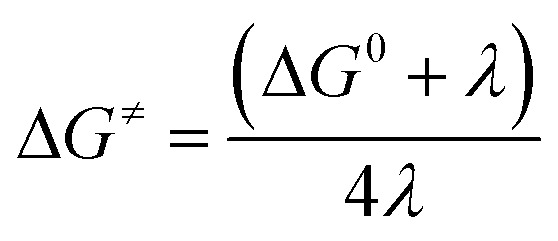

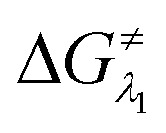
 and 
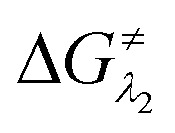
 represent the activation energy barriers of the forward and backward reactions, respectively.

## Conflicts of interest

There are no conflicts to declare.

## Supplementary Material

SC-012-D0SC05972B-s001

SC-012-D0SC05972B-s002

## References

[cit1] Longuet-Higgins H. C. (1950). J. Chem. Phys..

[cit2] Lefebvre R., Dearman H. H., Mcconnell H. M. (1960). J. Chem. Phys..

[cit3] Fukui K., Yonezawa T., Shingu H. (1952). J. Chem. Phys..

[cit4] Sogo P. B., Nakazaki M., Calvin M. (1957). J. Chem. Phys..

[cit5] Reid D. H. (1958). Tetrahedron.

[cit6] Reid D. H. (1965). Q. Rev., Chem. Soc..

[cit7] Wei H., Liu Y., Gopalakrishna T. Y., Phan H., Huang X., Bao L., Guo J., Zhou J., Luo S., Wu J., Zeng Z. (2017). J. Am. Chem. Soc..

[cit8] Suzuki S., Morita Y., Fukui K., Sato K., Shiomi D., Takui T., Nakasuji K. (2005). J. Am. Chem. Soc..

[cit9] Haddon R. C. (1975). Nature.

[cit10] Goto K., Kubo T., Yamamoto K., Nakasuji K., Sato K., Shiomi D., Takui T., Kubota M., Kobayashi T., Yakusi K. (1999). J. Am. Chem. Soc..

[cit11] Chi R., Itkis M. E., Patrick B. O., Barclay B. O., Reed R. W., Oakley R. T., Cordes A. W., Haddon R. C. (1999). J. Am. Chem. Soc..

[cit12] Pal S. K., Itkis M. E., Tham F. S., Reed R. W., Oakley R. T., Haddon R. C. (2005). Science.

[cit13] Mandal S. K., Samanta S., Itkis M. E., Jensen D. W., Reed R. W., Oakley R. T., Tham F. S., Donnadieu B., Haddon R. C. (2006). J. Am. Chem. Soc..

[cit14] Morita Y., Nishida S., Murata T., Moriguchi M., Ueda A., Satoh M., Arifuku K., Sato K., Takui T. (2011). Nat. Mater..

[cit15] Ueda A., Suzuki S., Yoshida K., Fukui K., Sato K., Takui T., Nakasuji K., Morita Y. (2013). Angew. Chem., Int. Ed..

[cit16] Anamimoghadam O., Symes M. D., Long D.-L., Sproules S., Cronin L., Bucher G. J. (2015). J. Am. Chem. Soc..

[cit17] Morita Y. and Nishida S., Stable radicals: Fundamentals and applied aspects of odd-electron compounds, ed R. Hicks, John Wiley & Sons, 2010, vol. 606pp ISBN: 978-0-470-77083-2

[cit18] Mou Z., Uchida K., Kubo T., Kertesz M. (2014). J. Am. Chem. Soc..

[cit19] Itkis M. E., Chi X., Cordes A. W., Haddon R. C. (2002). Science.

[cit20] Raman K. V., Kamerbeek A. M., Mukherjee A., Atodiresei N., Sen T. K., Lazic R., Caciuc V., Michel R., Stalke D., Mandal S. K., Blugel S., Munzenberg M., Moodera J. S. (2013). Nature.

[cit21] Pariyar A., Vijaykumar G., Bhunia M., Dey S. K., Singh S. K., Kurungot S., Mandal S. K. (2015). J. Am. Chem. Soc..

[cit22] Vijaykumar G., Pariyar A., Ahmed J., Shaw B. K., Adhikari D., Mandal S. K. (2018). Chem. Sci..

[cit23] Ahmed J., Sreejyothi P., Vijaykumar G., Jose A., Raj M., Mandal S. K. (2017). Chem. Sci..

[cit24] Bhunia M., Sahoo S. R., Shaw B. K., Vaidya S., Pariyar A., Vijaykumar G., Adhikari D., Mandal S. K. (2019). Chem. Sci..

[cit25] Ahmed J., Chakraborty S., Jose A., Sreejyothi P., Mandal S. K. (2018). J. Am. Chem. Soc..

[cit26] Mukherjee A., Sau S. C., Mandal S. K. (2017). Acc. Chem. Res..

[cit27] Small D. (2004). et al.. J. Am. Chem. Soc..

[cit28] Tian Y.-H., Kertesz M. (2010). J. Am. Chem. Soc..

[cit29] O'Connor G. D. (2011). et al.. J. Am. Chem. Soc..

[cit30] Morita V., Suzuki S., Sato K., Takui T. (2011). Nat. Chem..

[cit31] Hicks R. G. (2011). Nat. Chem..

[cit32] Kubo T. (2015). Chem. Rec..

[cit33] Kertesz M. (2019). Chem. - Eur. J..

[cit34] Ghosh I., Ghosh T., Bardagi J. I., König B. (2014). Science.

[cit35] Kim H., Kim H., Lambert T. H., Lin S. (2020). J. Am. Chem. Soc..

[cit36] Cowper N. G. W., Chernowsky C. P., Williams O. P., Wickens Z. K. (2020). J. Am. Chem. Soc..

[cit37] Scholz A. S., Massoth J. G., Bursch M., Mewes J.-M., Hetzke T., Wolf B., Bolte M., Lerner H.-W., Grimme S., Wagner M. (2020). J. Am. Chem. Soc..

[cit38] Geuenich D., Hess K., Koehler F., Herges R. (2005). Chem. Rev..

[cit39] Herges R., Geuenich D. (2001). J. Phys. Chem. A.

[cit40] Hassan J., Sevignon M., Gozzi C., Schulz E., Lemaire M. (2002). Chem. Rev..

[cit41] Colletto C., Panigrahi A., Fernandez-Casado J., Larrosa I. (2018). J. Am. Chem. Soc..

[cit42] Yanagisawa S., Ueda K., Taniguchi T., Itami K. (2008). Org. Lett..

[cit43] Sun C.-L., Li H., Yu D.-G., Yu M., Zhou X., Lu X.-Y., Huang K., Zheng S.-F., Li B.-J., Shi Z.-J. (2010). Nat. Chem..

[cit44] Shirakawa E., Itoh K.-I., Higashino T., Hayashi T. (2010). J. Am. Chem. Soc..

[cit45] Liu W., Cao H., Zhang H., Zhang H., Chung K. H., He C., Wang H., Kwong F. Y., Lei A. (2010). J. Am. Chem. Soc..

[cit46] Zhou S., Doni E., Anderson G. M., Kane R. G., MacDougall S. W., Ironmonger V. M., Tuttle T., Murphy J. (2014). J. Am. Chem. Soc..

[cit47] Wiberg K. B., Hadad C. H., Sieber S., Schleyer P. v. R. (1992). J. Am. Chem. Soc..

[cit48] Small D. W., Matyushov D. V., Voth G. A. (2003). J. Am. Chem. Soc..

[cit49] Vuilleumier R., Tay K. A., Jeanmairet G., Borgis D., Boutin A. (2012). J. Am. Chem. Soc..

[cit50] Rosokha S. V., Kochi J. K. (2007). J. Am. Chem. Soc..

[cit51] Frisch M. J. , et al. Gaussian 16, Revision B.01, Fox, Gaussian, Inc., Wallingford CT, 2016

[cit52] Becke A. D. (1993). J. Chem. Phys..

[cit53] Lee C., Yang W., Parr R. G. (1988). Phys. Rev. B.

[cit54] Chen Z., Wannere C. S., Corminboeuf C., Puchta R., Schleyer P. R. (2005). Chem. Rev..

[cit55] Nelsen S. F., Weaver M. N., Luo Y., Pladziewicz J. R., Ausman L. K., Jentzsch T. L., O'Konek J. J. (2006). J. Phys. Chem. A.

[cit56] Nelson J., Kwiatkowski J. J., Kirkpatrick J., M Frost J. (2009). Acc. Chem. Res..

[cit57] Marcus R. A. (1964). Annu. Rev. Phys. Chem..

[cit58] Hush N. S. (1958). J. Chem. Phys..

[cit59] Salzner U., Aydin A. (2011). J. Chem. Theory Comput..

