# Supplementary material for: Switching between mono and doubly reduced odd alternant hydrocarbon: designing a redox catalyst
Source: Chem Sci. 2020 Dec 23;12(8):3039–49. doi: 10.1039/d0sc05972b (PMC8179390; doi:10.1039/d0sc05972b)
Supplement: SC-012-D0SC05972B-s001 [file SC-012-D0SC05972B-s001.pdf]

Electronic Supplementary Material (ESI) for Chemical Science.
This journal is © The Royal Society of Chemistry 2020

Supporting Information

Switching between mono- and doubly-reduced odd alternant hydrocarbon: Designing redox catalyst

Jasimuddin Ahmed,[a] Paramita Datta,[a] Arpan Das,[a] Stephy Jomy[a,b] and Swadhin K. Mandal[a]*

[a]Department of Chemical Sciences, Indian Institute of Science Education and Research-Kolkata, Mohanpur-741246, India.

[b]Department of Chemistry, Indian Institute of Technology Madras, Chennai-600036, India.

*Correspondence to: swadhin.mandal@iiserkol.ac.in

1. Experimental section……………………………………………………………………...S4
2. Preparation and characterization of phenalenyl ligand and
the K-phenalenyl complexes……………………………………………………….....S5
I. Synthesis and characterization PLY(O,O)-K (**1a** and **1a(CE)**)……..............................S5
II. Synthesis and characterization PLY(N,O)-K (**1b** and **1b(CE)**)………………………S6
III. Isolation, characterization and crystallization of
PLY(O,O)-K monoreduced complex (**2**)……………………………………………S6
IV. Synthesis and characterization of doubly-reduced PLY(O,O)-K complex (**3**)………..S7
V. Trapping and characterization of doubly-reduced PLY(O,O)-K………………………S8
VI. Trapping and characterization of deuterium leveled doubly-reduced PLY(O,O)-K…..S9
3. Cyclic voltametric experiment of potassium phenalenyl complex……………………..S11
4. ^{1}H, ^{13}C NMR spectra and mass spectra of various phenalenyl species
and K-complexes…………………………………………………………….......S12
5. Computational Details……………………………………………………………….S24
6. Theoretically predicted ^{1}H NMR spectra……………………………………….......S25
7. Electrostatic potential maps of PLY(N,O)-K…………………………………………...S26
8. Anisotropic Induced Current Density (AICD) plots……………………………………S27
9. Model example for Nuclear Independent Chemical Shift (NICS) calculation…………S29
10. Theoretical calculations for all reduction processes……………………………………S34
11. Optimized geometries of three different redox states of PLY(O,O)-K complex….........S35
12. DFT calculation for the quenching experiment by HCl treatment
of doubly-reduced PLY-K complex……………………………………………………S36
13. Frontier molecular orbital diagrams of three different redox states
of K-phenalenyl complex……………………………………………………………...S37
14. Crystallographic and data collection parameters for **1a(CE)**, **1a(CE), 2**………………S39
15. Reaction Optimization for direct C-H arylation of N-methyl pyrrole
with 4-chlorobenzonitrile coupling partner……………………………………………S46
16. C-H arylation of arenes/heteroarenes with 4-chlorobenzonitrile
in absence of catalyst……………………………………………………………………S47

17. General procedure for C-H arylation of arenes/heteroarenes with aryl halides……….S48
18. General procedure for intramolecular coupling reactions……………………………..S48
19. Radical scavenging experiment in presence of TEMPO………………………………S49
20. Radical trapping experiment…………………………………………………………...S50
21. The analytical and spectral characterization data of the catalytic products……………S51
22. ^{1}H and ^{13}C NMR Spectra of the catalytic products……………………………………S66
23. Mass spectra……………………………………………………………………………S101
24. All computational details and theoretically obtained parameter……………………….S104
25. Coordinates of all DFT optimized structures…………………………………………..S117
26. Reference……………………………………………………………………………......S168

1. Experimental Section

Materials and Methods:

All solvents except THF and toluene used in the experiments were distilled from calcium hydride under inert condition prior to use. Toluene and THF were distilled using Na/benzophenone. All chemicals were purchased and used as received. The ^{1}H, ^{13}C NMR spectra were recorded on 400 and 500 MHz spectrometers in $CDCl_3$ with residual undeuterated solvent ($CDCl_3$, 7.26/77.0) as an internal standard. Chemical shifts (δ) are given in ppm, and J values are given in Hz. All chemical shifts were reported in ppm using tetramethylsilane as a reference. Chemical shifts (δ) downfield from the reference standard were assigned positive values. Column chromatography including thin-layer chromatography (TLC) was performed on silica gel (Merck silica gel 100-200 mesh). Evaporation of solvents was performed under reduced pressure using a rotary evaporator. High-resolution mass spectra (HRMS) were obtained on a Bruker maXis impact. ESI-mass spectra were recorded on a water Micro-MS mass spectrometer. EPR spectroscopic measurements were performed in Bruker (X-band) spectrometer. All the glassware and NMR tubes used for experiments were kept in oven at 120 °C for overnight (12h). X-ray crystallographic measurements were performed in Agilent X-ray diffractometer. All chemicals have been directly purchased from Sigma-Aldrich. 9-Hydroxyphenalenone ligand has been prepared by following the literature report[1]. The starting materials for intramolecular coupling reactions have been prepared by following the previous literature[2].

2. Preparation and characterization of phenalenyl ligand and the K-phenalenyl complexes:

I. Synthesis and characterization PLY(O,O)-K complex (1a and 1a(CE)):

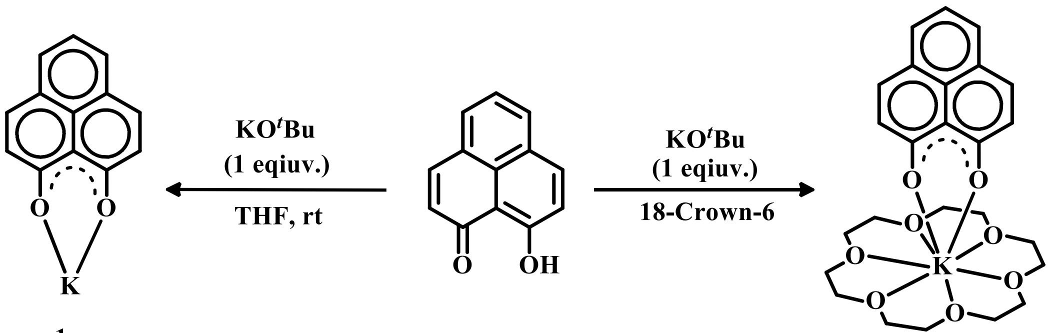

1a **1a(CE)**

Inside an argon filled glovebox, 0.1 mmol (20 mg) PLY(O,OH), 0.1 mmol (12 mg) KO^{t}Bu and 0.12 mmol (25 mg) 18-crown-6 were mixed together with 1.2 mL THF in a glass vial. The reaction mixture was stirred at room temperature for 10 min. The clear reddish solution was transferred to another vial and for crystallization under -35 ^{o}C along with 0.5 mL toluene as co-solvent. Rod shaped crystals were grown at overnight standing. A suitable crystal was coated with precooled inert oil and was analyzed by solid state structure. Also it was characterized by NMR spectroscopy.

The **1a** has been prepared following same procedure without adding 18-crown-6 and it was collected as precipitate.

^{1}H NMR (500 MHz, DMSO-d$_6$, 298K) δ (ppm) 3.55 (s, 24H), 6.56 (d, 2H, J = 8 Hz), 7.05 (t, 1H, J = 6 Hz), 7.57 (dd, 4H, J_1 = 8 Hz, J_2 = 12 Hz).

^{13}C{^{1}H} NMR (125 MHz, DMSO-d$_6$, 298K) δ (ppm) 69.9, 115.7, 118.2, 123.5, 129.1, 131.9, 132.3, 135.2, 179.7.

DEPT 135 (125 MHz, DMSO-d$_6$, 298K) δ (ppm) 69.9 (s, CH$_2$), 118.2 (CH), 129.1 (CH), 131.9 (CH), 135.2 (CH).

II. Synthesis and characterization PLY(N,O)-K (1b and 1b(CE)):

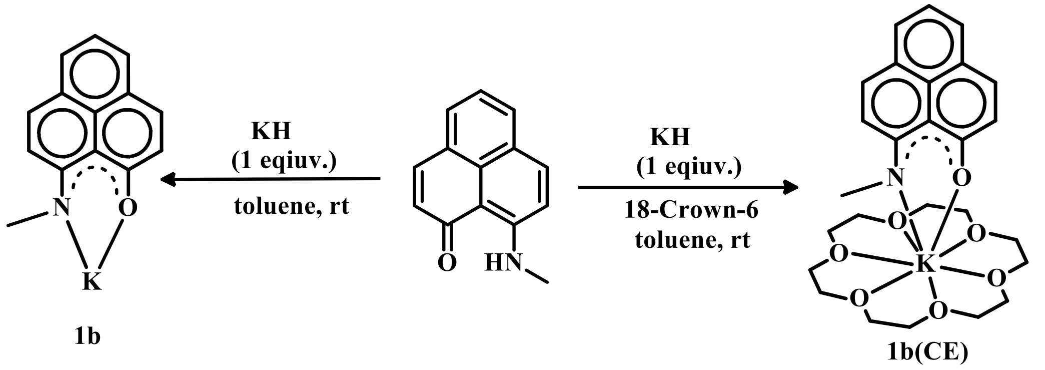

Inside an argon filled glovebox, 0.1 mmol (22 mg) PLY(O,NH), 0.1 mmol (4 mg) KH and 0.12 mmol (25 mg) 18-crown-6 were mixed together with 1.2 mL toluene in a glass vial. The reaction mixture was stirred at room temperature for 2-3 mins. The clear reddish solution was transferred to another vial and for crystallization under -35 ^{o}C along. Red colored block shaped crystals were grown at overnight standing. A suitable crystal was coated with precooled inert oil and mounted under 100 K and analyzed by solid state structure. Instability of this molecule in DMSO and very low solubility in THF or toluene does not allow us to record the NMR spectrum.

1b was prepared following the same procedure without adding 18-crown-6 and it was collected as precipitate.

III. Synthesis and characterization of PLY(O,O)-K mono-reduced complex (2):

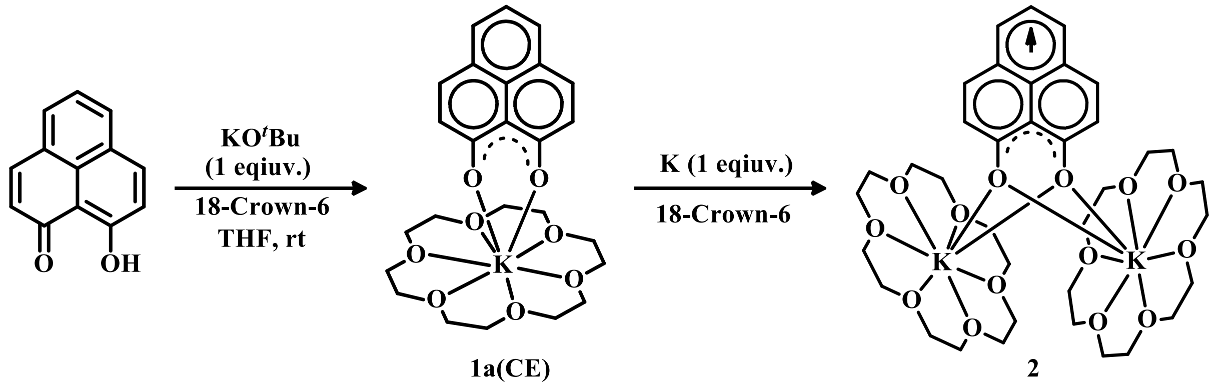

Inside an argon filled glovebox, 0.1 mmol (20 mg) PLY(O,OH), 0.1 mmol (12 mg) KO^{t}Bu and 0.12 mmol (25 mg) 18-crown-6 were mixed together with 1.2 mL THF in a glass vial. 0.12 mmol (5 mg) K and 0.12 mmol (25 mg) 18-crown-6 were added in the reaction mixture. The final reaction mixture was stirred for 10 min and the red colored solution slowly turned into a green colored solution. The clear green solution was transferred to another vial and for crystallization under -35 °C along with 0.5 mL toluene as co-solvent. Blocked shaped crystals were grown at overnight standing. A suitable crystal was coated with precooled inert oil and was analyzed by solid state structure.

EPR measurement of this green crystal shows a sharp signal at g = 2.0001 in X-band. NMR spectroscopic measurement shows broad NMR signals in the ^{1}H NMR spectrum.

IV. Synthesis and characterization of doubly-reduced PLY(O,O)-K complex (3):

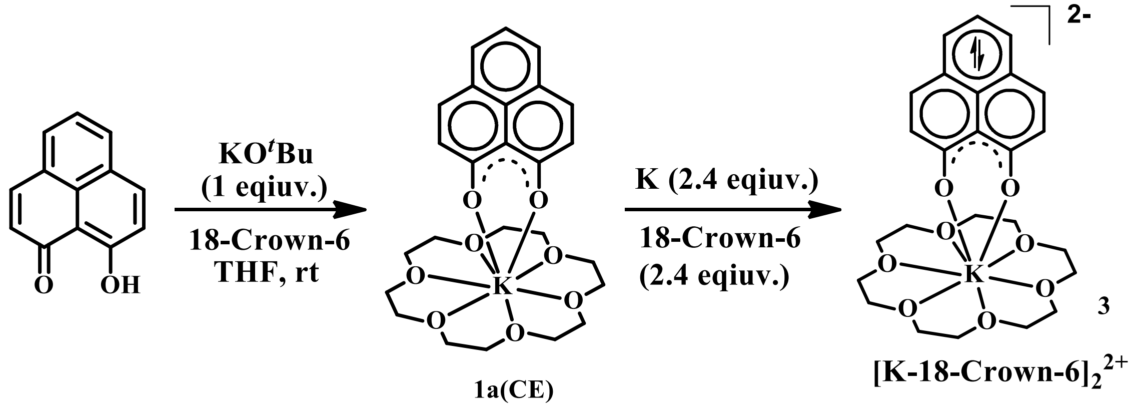

Inside an argon filled glovebox, 0.1 mmol (20 mg) PLY(O,OH), 0.1 mmol (12 mg) KO^{t}Bu and 0.12 mmol (25 mg) 18-crown-6 were mixed together with 2 mL THF in a glass vial. 0.24 mmol (12 mg) K and 0.24 mmol (50 mg) 18-crown-6 were added in the reaction mixture. The final reaction mixture was stirred at room temperature, after 10 min the red colored solution slowly turned into a green colored solution and after another 15 min, this green color slowly turns into

dark brown solution. EPR measurement of this brown colored solution shows it as EPR silent. ^{1}H NMR spectroscopic measurement of this brown solution shows resonance signal at upfield region with respect to the neutral PLY(O,O)-K (Figure S10).

V. Trapping and characterization of doubly-reduced PLY(O,O)-K:

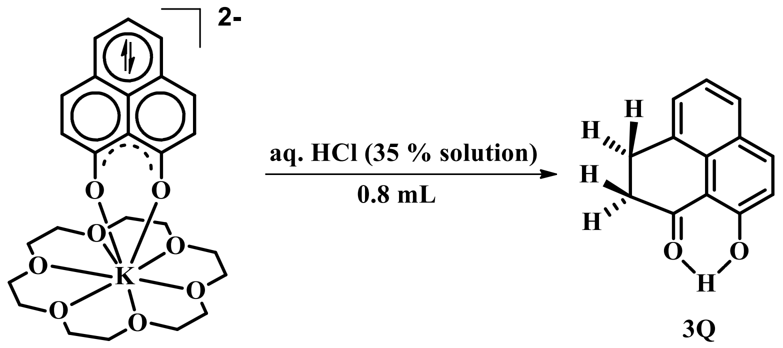

3 3Q

Inside an argon filled glovebox, 0.1 mmol (20 mg) PLY(O,OH), 0.1 mmol (12 mg) KO^{t}Bu and 0.12 mmol (25 mg) 18-crown-6 were mixed together with 2 mL THF in a glass vial. 0.24 mmol (12 mg) K and 0.24 mmol (50 mg) 18-crown-6 were added in the reaction mixture. The final reaction mixture was stirred at room temperature, after 10 min, the red colored solution slowly turned into a green colored solution and after another 15 min, this green color slowly turns into dark brown solution. 0.8 mL aq. HCl solution (35%) was added into that brown solution. The organic compound was extracted in ethylacetate from water and after solvent evaporation, the organic compound was dried. The product was isolated from column chromatography using hexane as eluent over silica. The isolated product was characterized by mass and NMR spectroscopic measurements.

^{1}H NMR (500 MHz, $CDCl_3$, 298K) δ (ppm) 2.94 (t, 2H, J = 6 Hz), 3.33 (t, 2H, J = 6 Hz), 7.16 (d, 1H, J = 10 Hz), 7.33 (t, 1H, J = 7 Hz), 7.41 (d, 1H, J = 8 Hz), 7.66 (d, 1H, J = 8.4 Hz), 7.97 (d, 1H, J = 8.2 Hz), 13.1 (s, 1H).

^{13}C{^{1}H} NMR (125 MHz, $CDCl_3$, 298K) δ (ppm) 27.6, 37.1, 110.2, 119.3, 124.0, 126.6, 126.7, 127.1, 130.7, 131.6, 138.1, 162.5, 203.8.

DEPT 135 (125 MHz, $CDCl_3$, 298K) δ (ppm) 27.6 (CH2), 37.1 (CH2), 119.3 (CH), 124.0 (CH), 126.6 (CH), 126.7 (CH), 138.1 (CH).

VI. Trapping and characterization of deuterium leveled doubly-reduced PLY(O,O)-K:

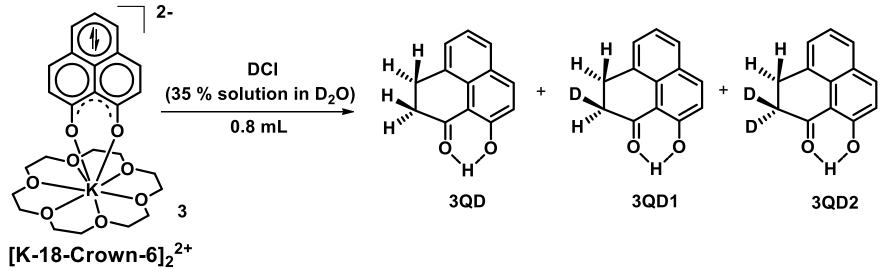

Inside an argon filled glovebox, 0.1 mmol (20 mg) PLY(O,OH), 0.1 mmol (12 mg) KO^{t}Bu and 0.12 mmol (25 mg) 18-crown-6 were mixed together with 2 mL THF in a glass vial. 0.24 mmol (12 mg) K and 0.24 mmol (50 mg) 18-crown-6 were added to the reaction mixture. The final reaction mixture was stirred at room temperature, after 10 min, the red colored solution slowly turned into green colored solution and after another 15 min this green color slowly turned into dark brown solution. 0.8 mL DCl solution (35% in D_2O) was added into that brown solution. The organic compound was extracted in ethyl acetate from water, after solvent evaporation the organic compound was dried. The reaction mixture was subjected to the mass spectrometric measurement

(HRMS). The mass spectrum shows the presence of non-deuterated, mono-deuterated and di-deuterated quenched species along with these we could found PLY(O,OH). The mass spectrum has been presented in Figure S17.

The product was isolated by column chromatography using hexane as eluent over silica. The isolated product was characterized by mass and NMR spectroscopic measurements.

^{1}H NMR (500 MHz, $CDCl_3$, 298K) δ (ppm) 2.94 (m, 0.73 H), 3.33 (m, 2.34H), 7.16 (d, 1H, J = 10 Hz), 7.33 (t, 1H, J = 7 Hz), 7.41 (d, 1H, J = 8 Hz), 7.66 (d, 1H, J = 8.4 Hz), 7.97 (d, 1H, J = 8.2 Hz), 13.1 (s, 1H).

DEPT 135 (125 MHz, $CDCl_3$, 298K) δ (ppm) 27.6 (CH_2, m), 37.1 (CH, d), 119.3 (CH), 124.0 (CH), 126.6 (CH), 126.7 (CH), 138.1 (CH).

^{2}D NMR (500 MHz, $CHCl_3$, 298K) δ (ppm) 2.93 (s, CD_2).

3. Cyclic voltammetric study:

Cyclic voltammetric study of PLY(O,O)-K complex (**1a**) was carried out in N*n*Bu_4ClO_4 0.1 M solution in DMF using Ag/AgCl reference electrode with 100mVs^{-1} at room temperature under N_2 atmosphere.

a)

b)

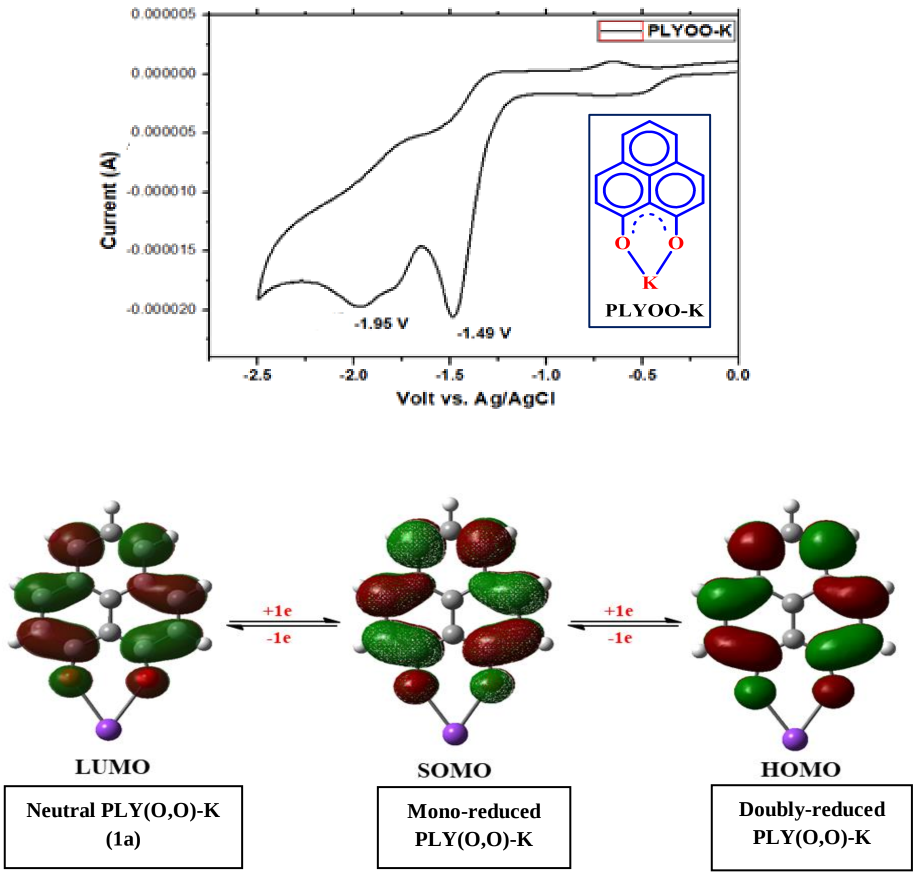

Figure S1. a) Cyclic voltammogram of **1a**; b) Frontier Molecular Orbital (FMO) diagram of three different redox states.

4. ^{1}H, ^{13}C NMR spectra and mass spectra of various phenalenyl species and K-complexes:

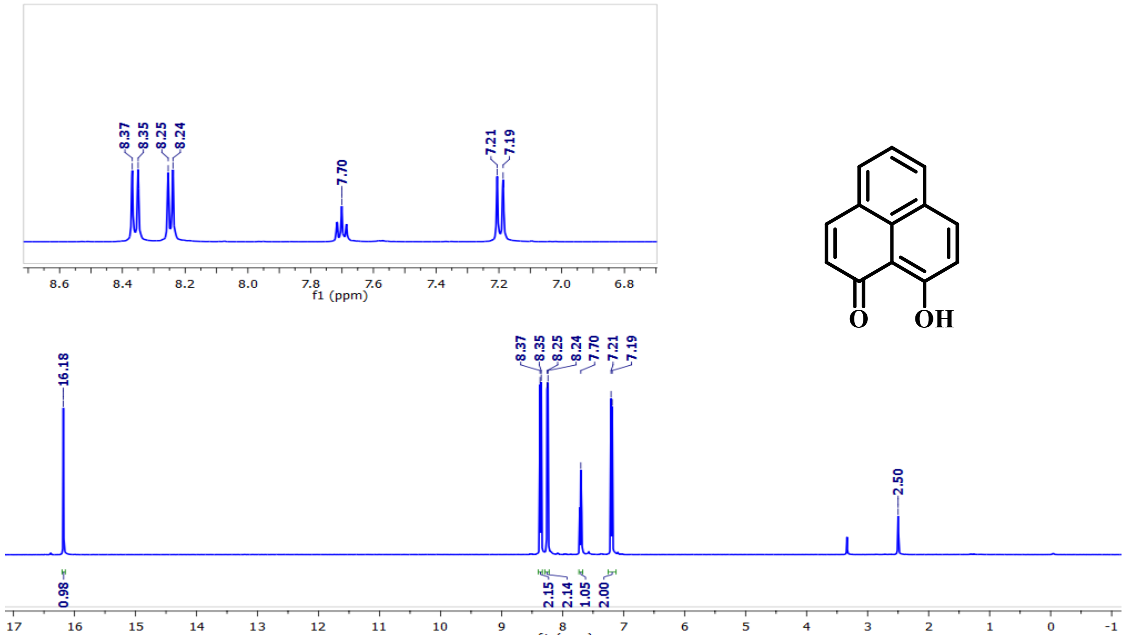

Figure S2. ^{1}H NMR ($CDCl_3$) spectrum of 9-hydroxyphenalenone (PLY(OH,O)).

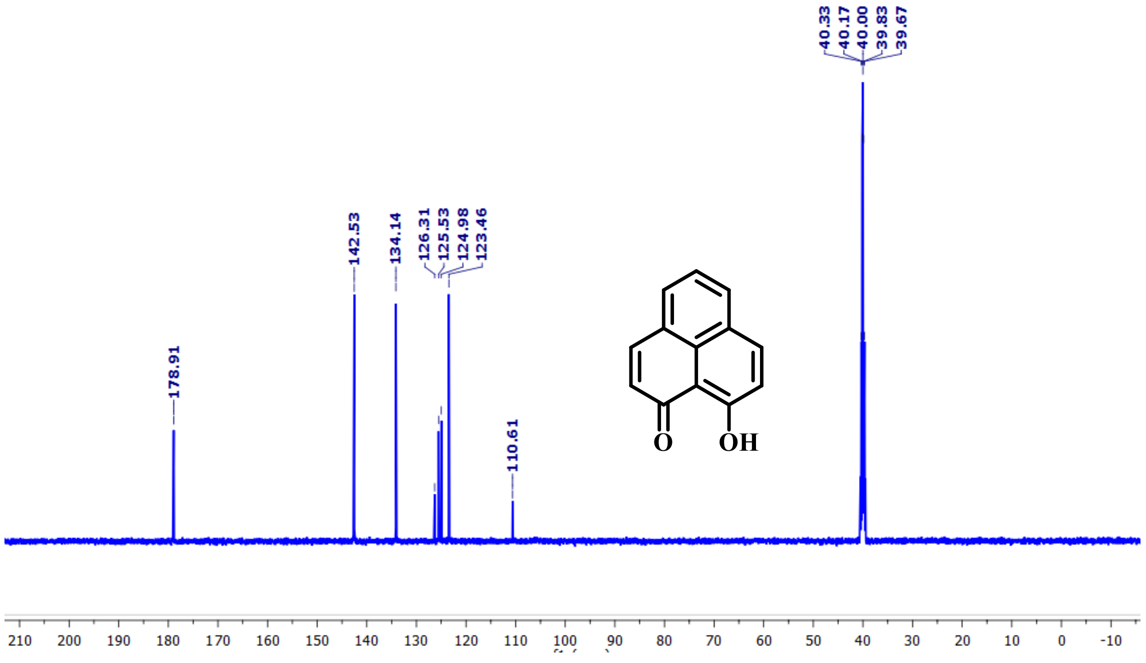

Figure S3. ^{13}C NMR ($CDCl_3$) spectrum of 9-hydroxyphenalenone (PLY(OH,O)).

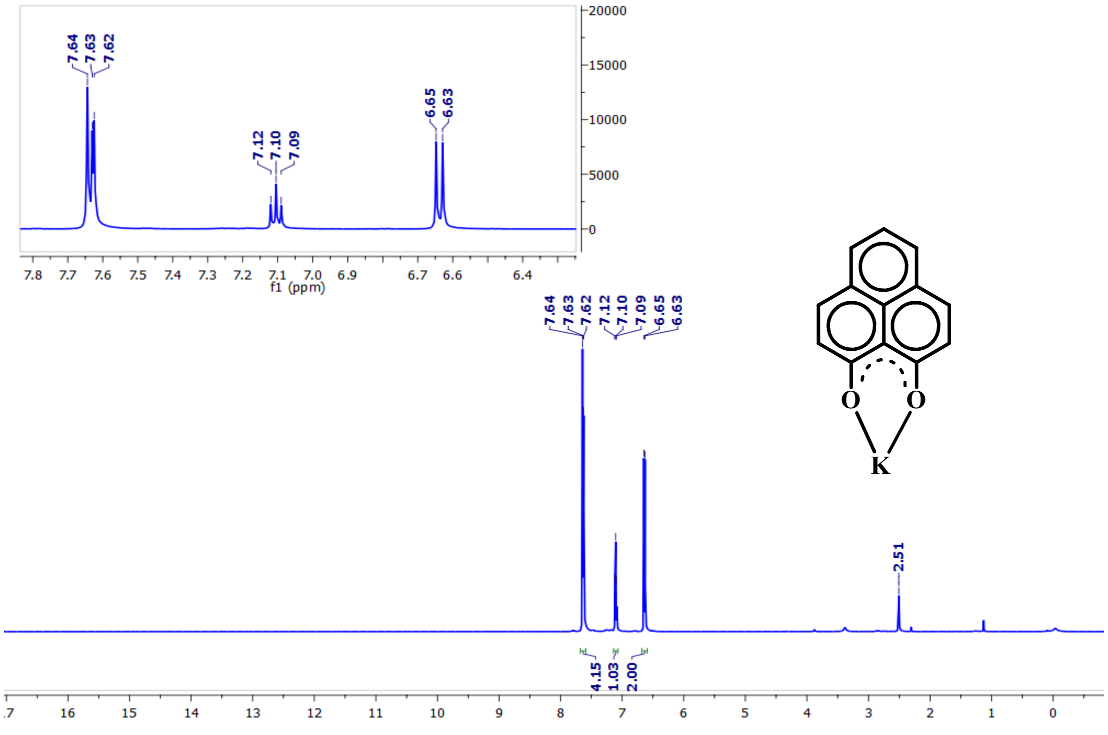

Figure S4. ^{1}H NMR (DMSO-d_6) spectrum of PLY(O,O)-K complex (**1a**).

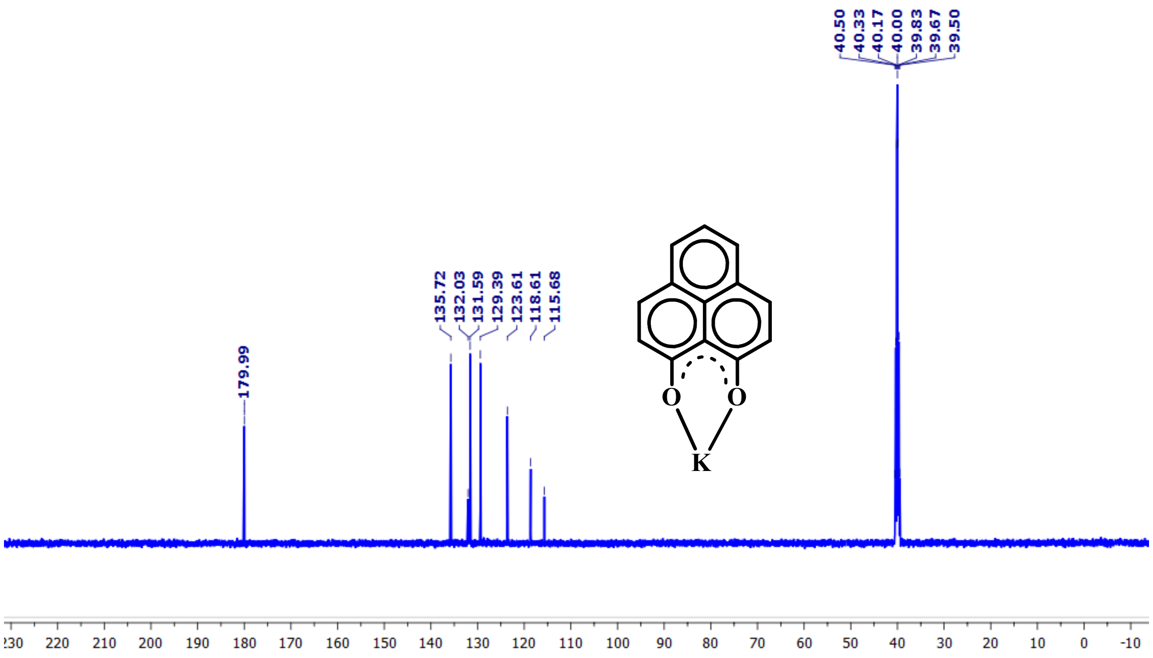

Figure S5. ^{13}C NMR (DMSO-d_6) spectrum of PLY(O,O)-K complex (**1a**).

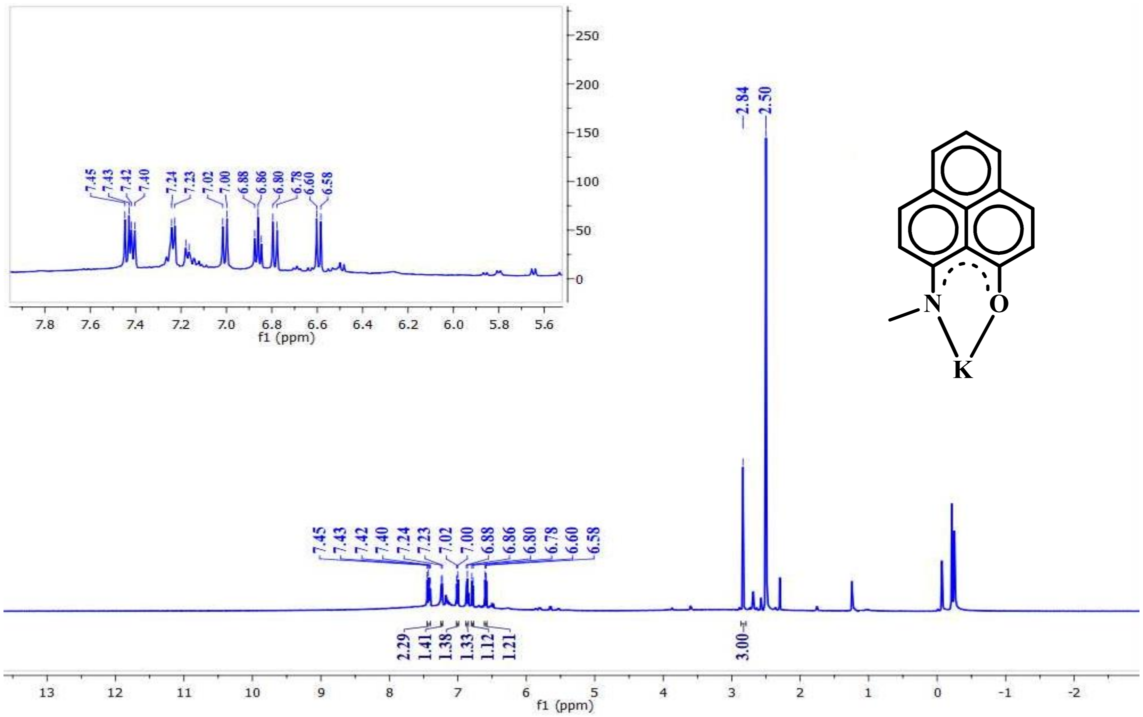

Figure S6. ^{1}H NMR (DMSO-d_6) spectrum of PLY(N,O)-K complex (**1b**)

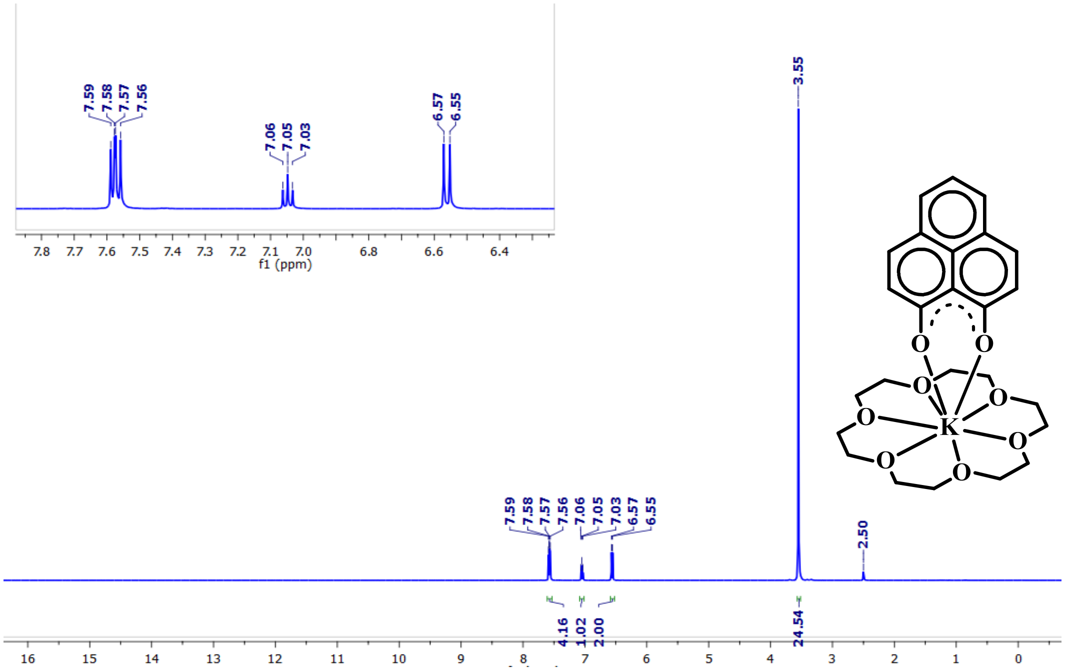

Figure S7. ^{1}H NMR (DMSO-d_6) spectrum of PLY(O,O)-K(18-crown-6) complex (**1a(CE)**).

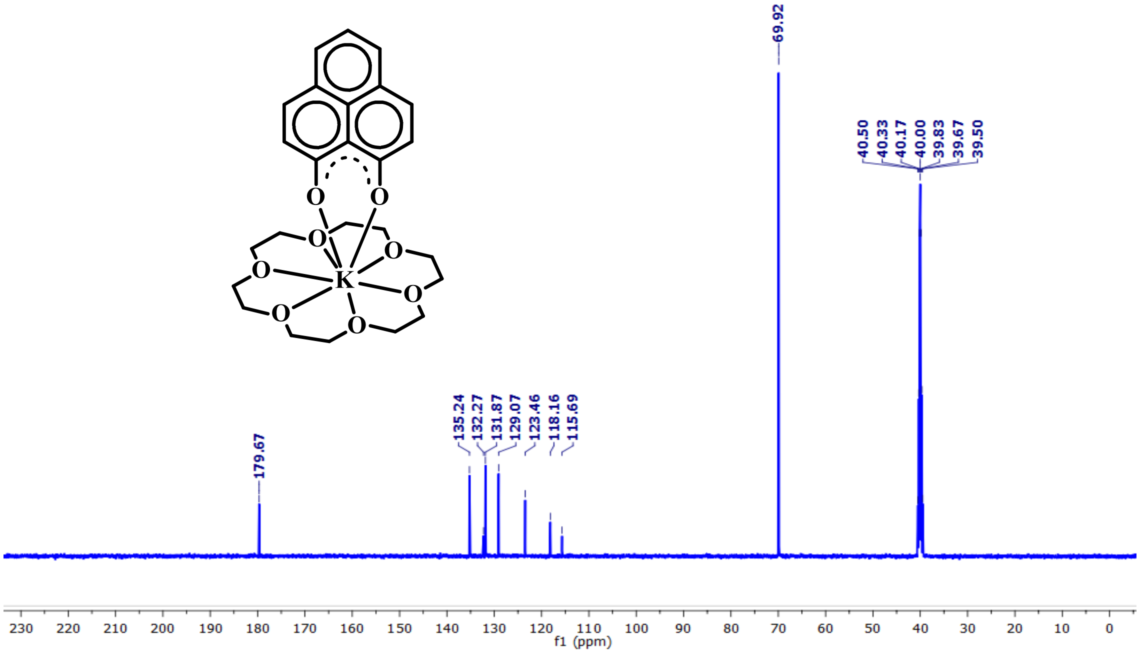

Figure S8. ^{13}C NMR (DMSO-d_6) spectrum of PLY(O,O)-K(18-crown-6) complex (**1a(CE)**).

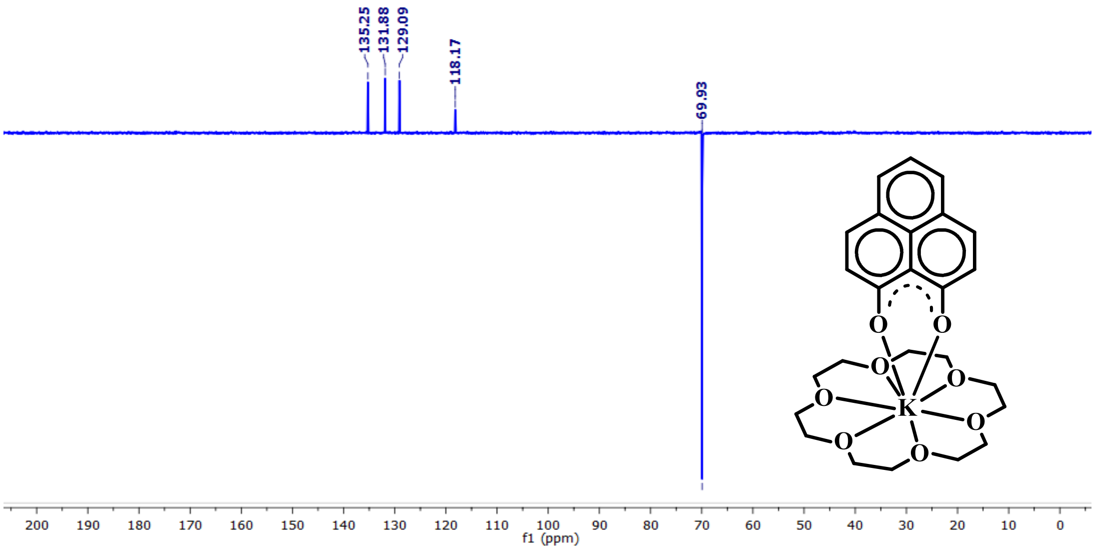

Figure S9. (^{13}C) DEPT 135 NMR (DMSO-d_6) spectrum of PLY(O,O)-K(18-crown-6) complex (**1a(CE)**).

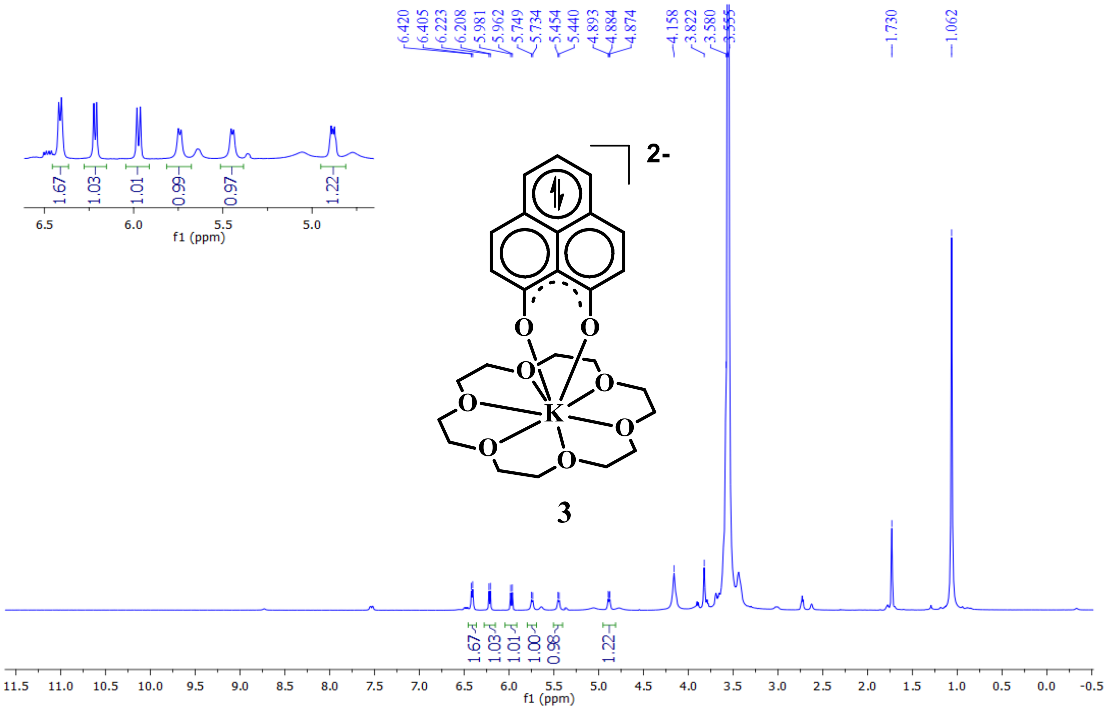

Figure S10. ^{1}H NMR (THF-d_8) spectrum of reaction mixture of doubly-reduced PLY species **3**.

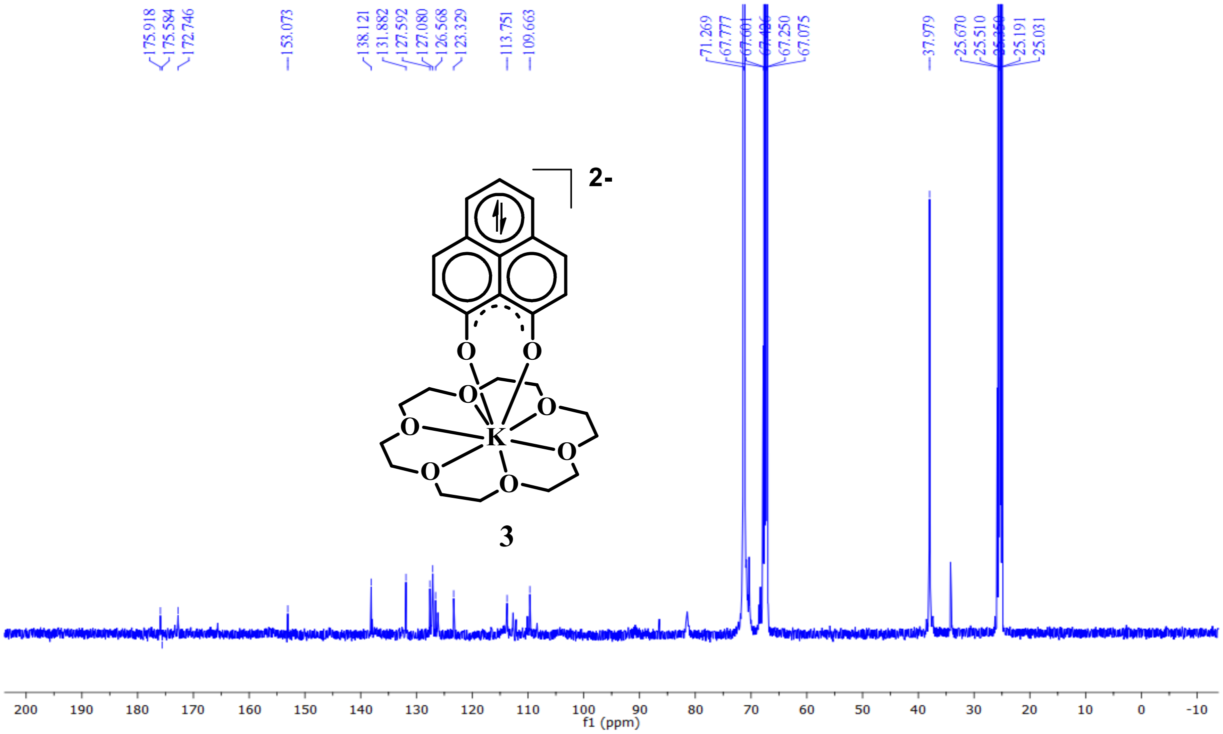

Figure S11. ^{13}C NMR (THF-d_8) spectrum of reaction mixture of doubly-reduced PLY species **3**.

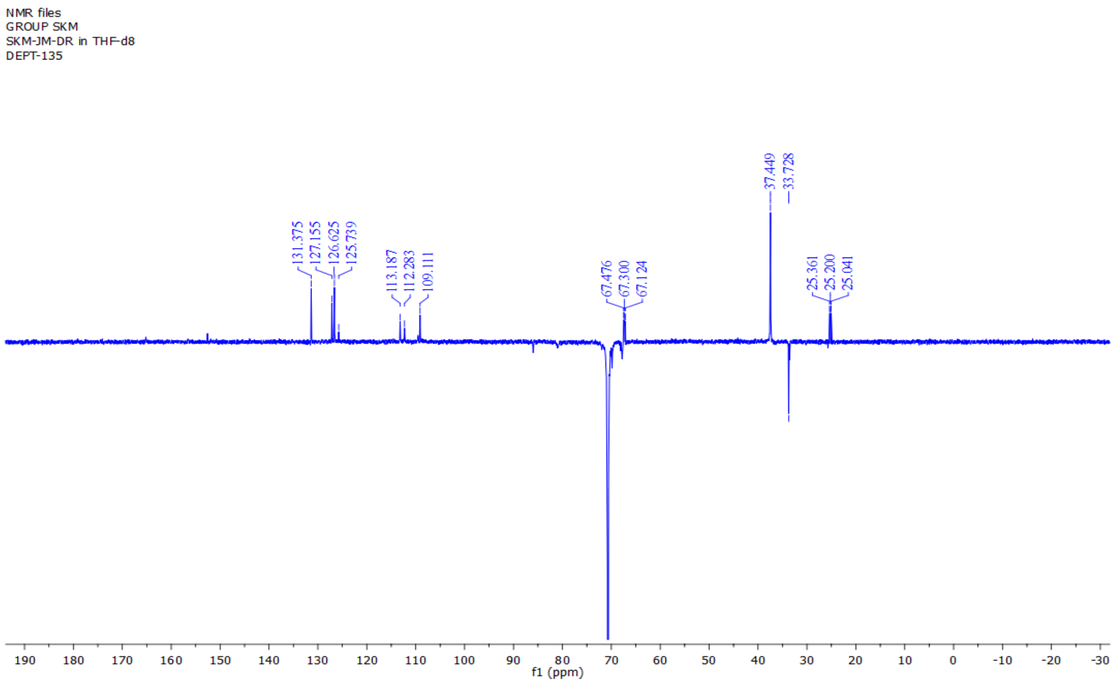

Figure S12. (^{13}C) DEPT 135 NMR (THF-d_8) spectrum of reaction mixture of doubly-reduced PLY species **3**.

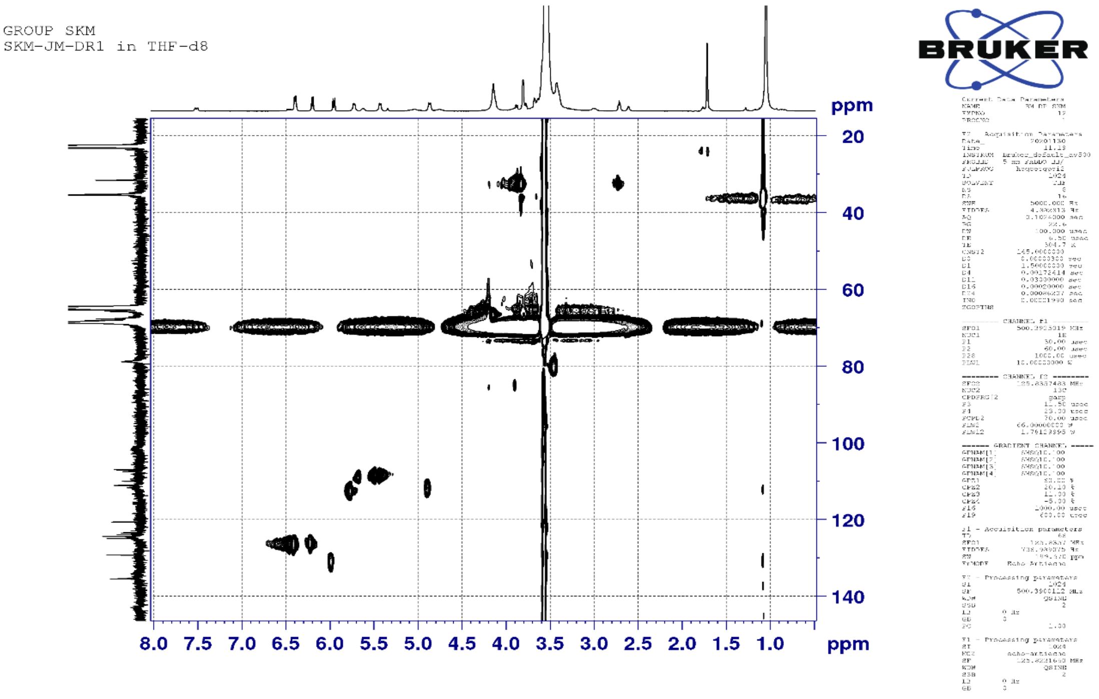

Figure S13. HSQC NMR (THF-d_8) spectrum of reaction mixture of doubly-reduced PLY species **3**.

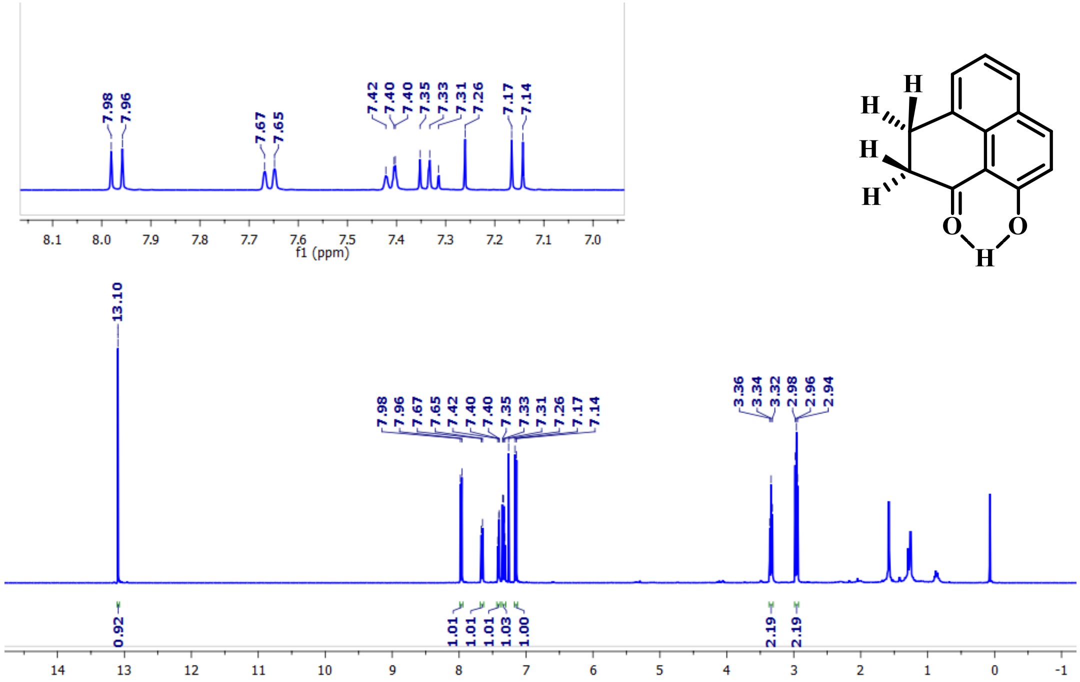

Figure S14. ^{1}H NMR ($CDCl_3$) spectrum of quenched PLY(O,O) (**3Q**).

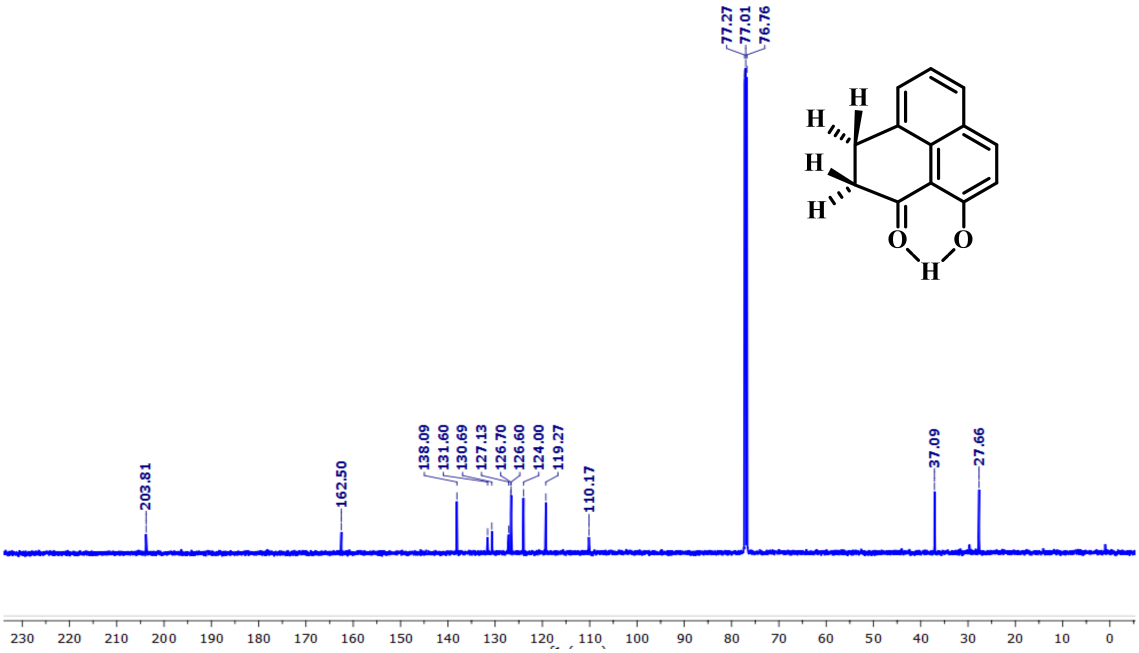

Figure S15. ^{13}C NMR ($CDCl_3$) spectrum of quenched PLY(O,O) (**3Q**).

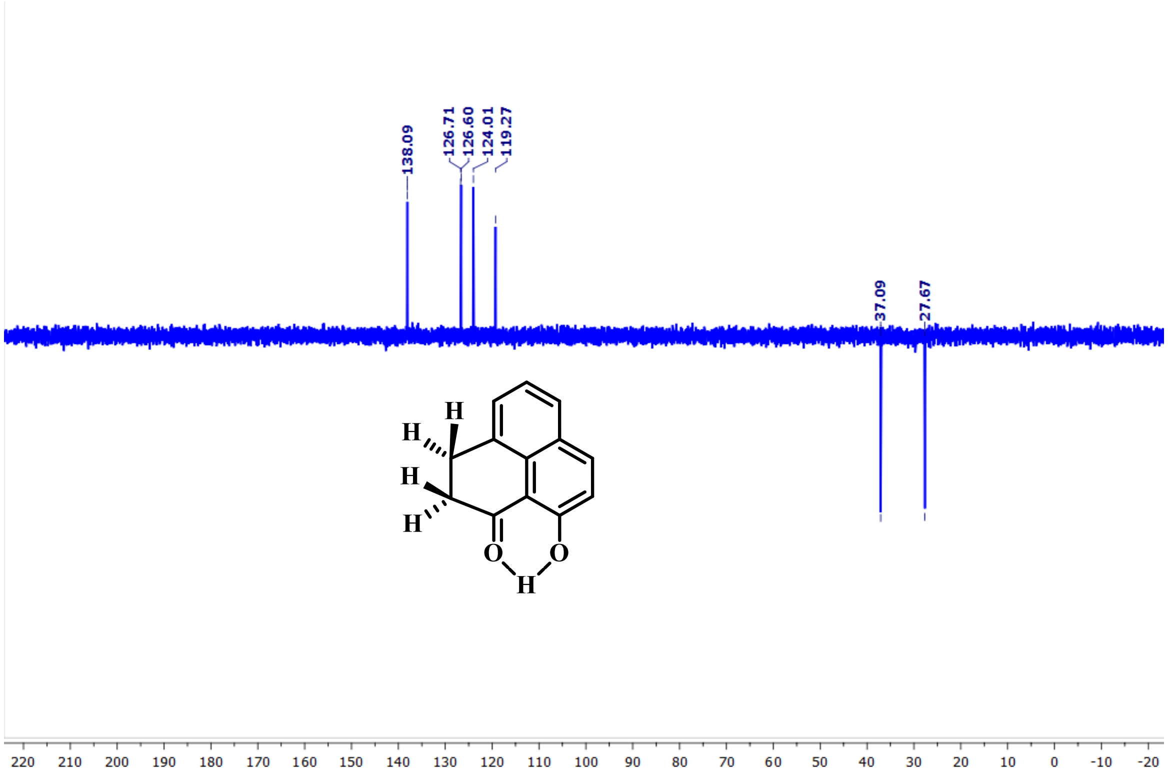

Figure S16. (^{13}C) DEPT135 NMR ($CDCl_3$) spectrum of quenched PLY(O,O) (**3Q**).

Display Report

Analysis Info

Analysis Name	D:\Data\User data\2020\NOV\skm-jmd1.d	Acquisition Date	12/9/2020 2:51:52 PM
Method	Tune_pos_Standard.m	Operator	IISER Kolkata
Sample Name	skm-jmd1	Instrument	maXis impact 8282001.00127
Comment			

Acquisition Parameter

Source Type	ESI	Ion Polarity	Positive	Set Nebulizer	0.5 Bar
Focus	Active	Set Capillary	3400 V	Set Dry Heater	200 °C
Scan Begin	50 m/z	Set End Plate Offset	-500 V	Set Dry Gas	4.0 l/min
Scan End	1000 m/z	Set Charging Voltage	2000 V	Set Divert Valve	Source
		Set Corona	0 nA	Set APCI Heater	0 °C

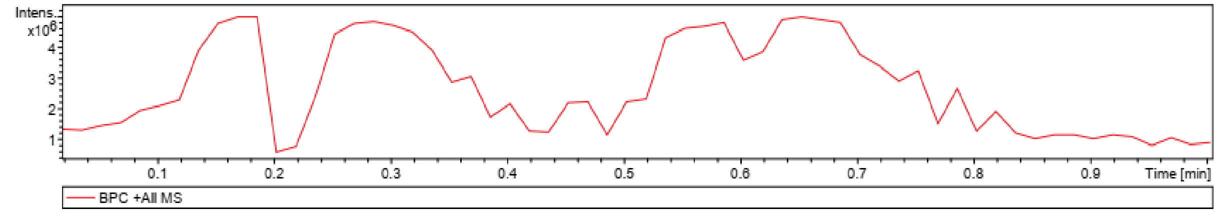

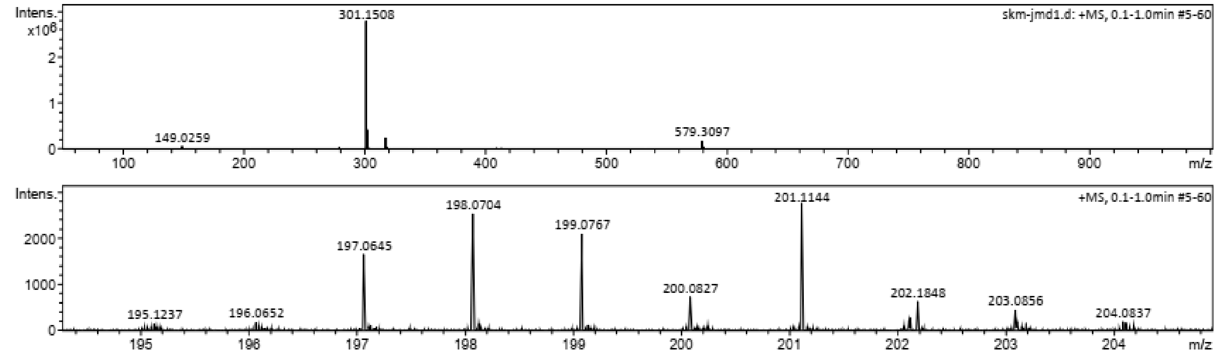

skm-jmd1.d

Bruker Compass DataAnalysis 4.1 printed: 12/9/2020 5:25:52 PM by: IISER Kolkata Page 1 of 1

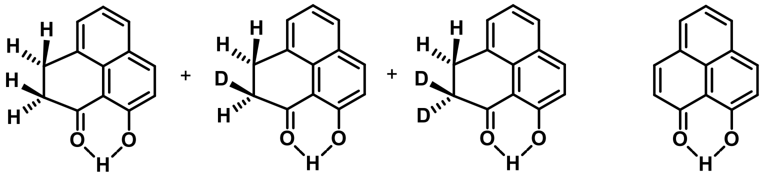

	3QD	3QD1	3QD2	PLY(O,O)
Caculated values	M+H$^+$: 199.0756	M+H$^+$: 200.0817	M+H$^+$: 201.0880	M+H$^+$: 197.0598
Observed values	199.0767	200.0827	201.1144	197.0645

Figure S17. Mass spectrum of reaction mixture of quenched PLY(O,O) with DCl.

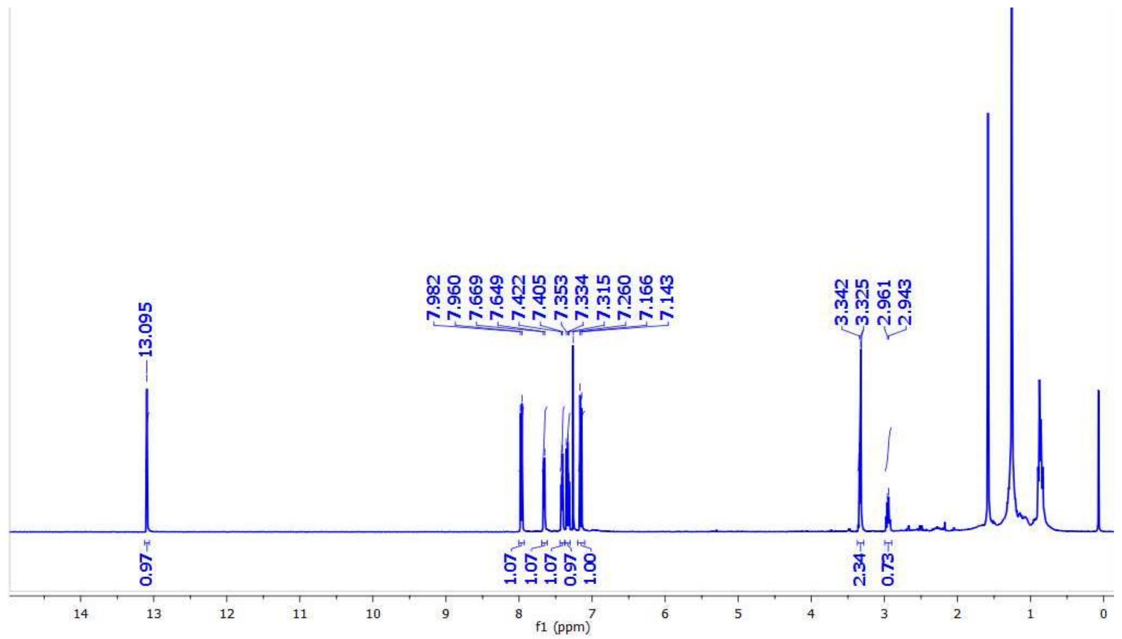

Figure S18. ^{1}H NMR ($CDCl_3$) spectrum of quenched PLY(O,O) with DCl (35% solution in D_2O).

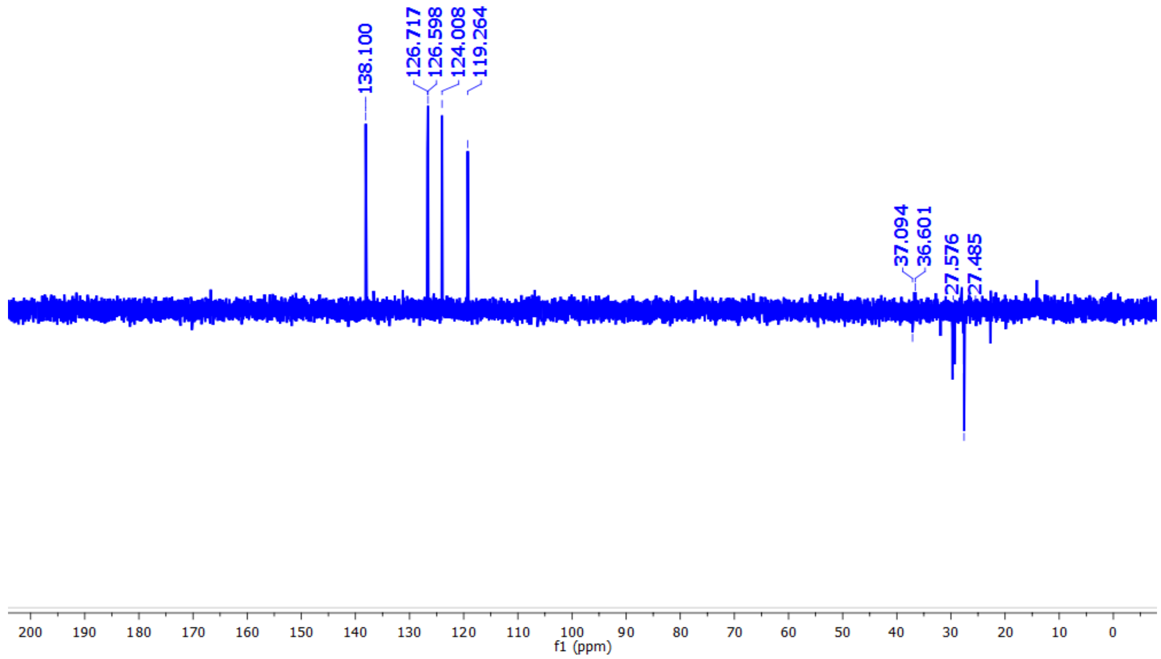

Figure S19. (^{13}C) DEPT 135 NMR ($CDCl_3$) spectrum of quenched PLY(O,O) with DCl (35% solution in D_2O) (**3QD**).

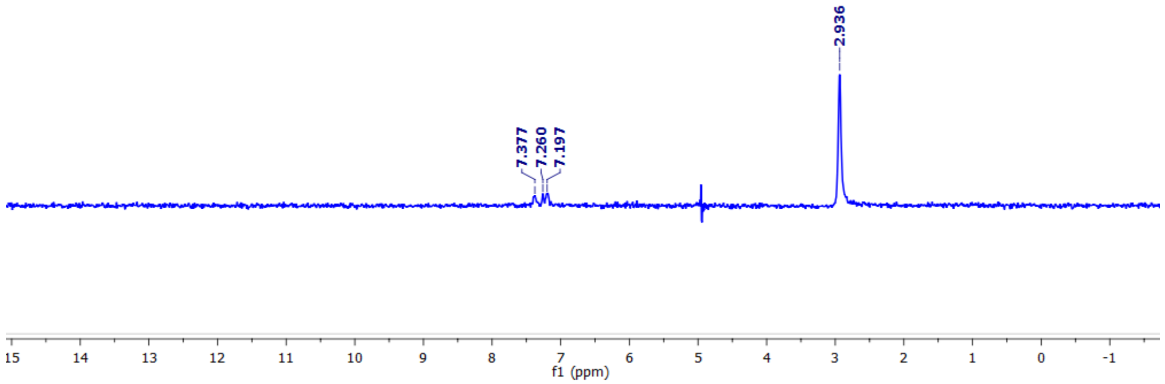

Figure S20. D NMR ($CHCl_3$) spectrum of quenched PLY(O,OH) with DCl (35% solution in D_2O).

Acquisition Parameter

Source Type	ESI	Ion Polarity	Positive	Set Nebulizer	0.3 Bar
Focus	Active	Set Capillary	3400 V	Set Dry Heater	200 °C
Scan Begin	50 m/z	Set End Plate Offset	-500 V	Set Dry Gas	4.0 l/min
Scan End	1000 m/z	Set Charging Voltage	2000 V	Set Divert Valve	Source
		Set Corona	0 nA	Set APCI Heater	0 °C

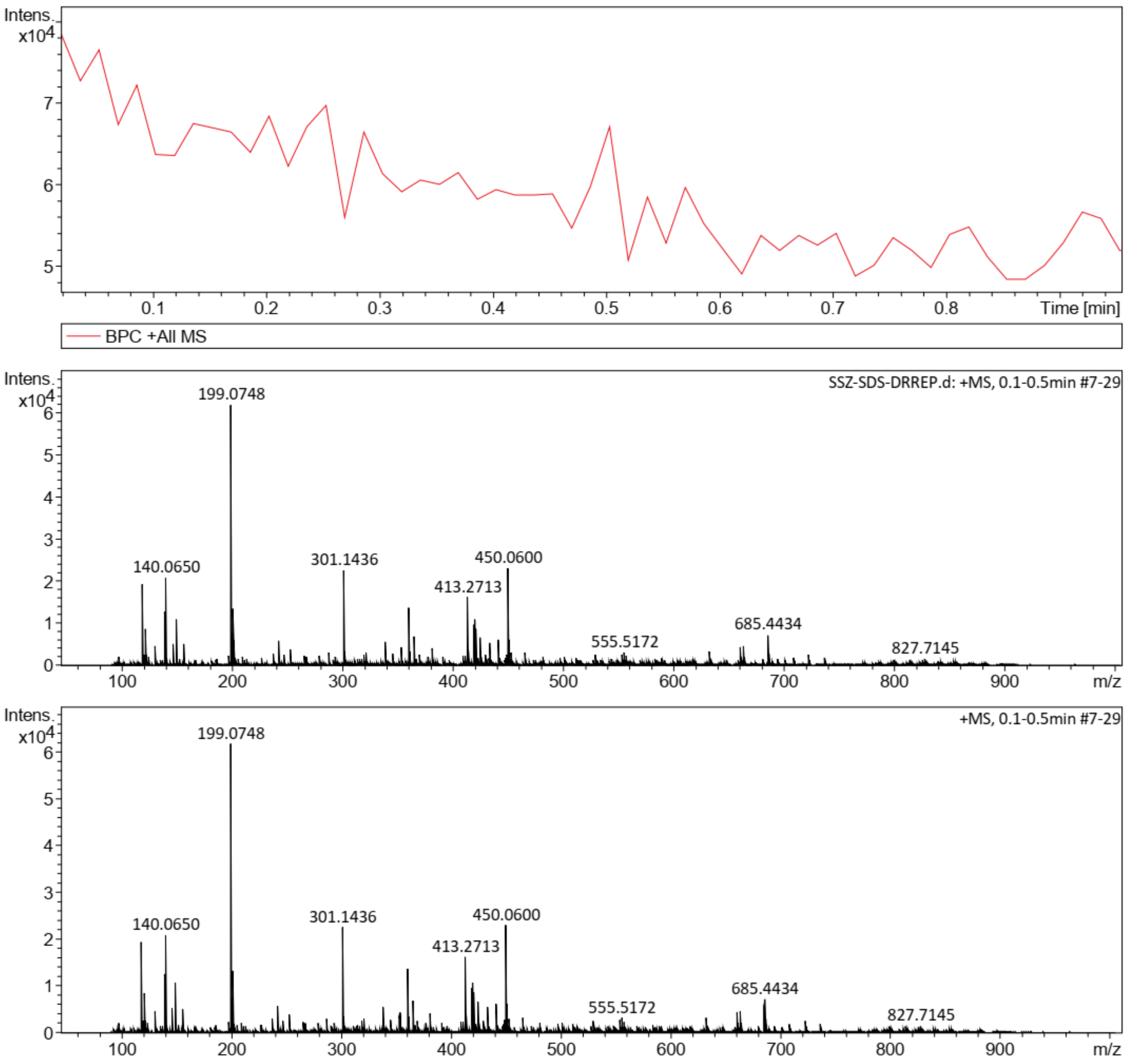

Figure S21. Mass Spectrum of quenched PLY(O,O) (**3Q**).

5. Computational Details:

Theoretical calculations were performed with the Gaussian16 program suite[3]. All theoretical calculations were carried out using the density functional theory (DFT) method with Becke's three-parameter hybrid exchange functionals and the Lee-Yang-Parr correlation functional (B3LYP) employing the 6-31G(d) basis set[4-5] for all atoms. Anisotropic Induced Current Density (ACID) plots (B3LYP/6-311g(d,p)) were calculated by using the method developed by Herges and only π-orbitals are considered. CSGT NMR calculation was performed for this ACID plots[6,7]. The plots were generated using with AICD-3.0.2 version with threshold vector 1.5 Å and isovalue 0.04. Nuclear Independent Chemical Shift (NICS) values were calculated (B3LYP/6-311G(d,p)) using the standard GIAO procedure[8]. CPCM solvent model has been used in these calculations[9].

6. Theoretically predicted ^{1}H NMR spectra:

Structure of neutral PLY(O,O)-K complex and doubly-reduced **3** have been optimized by DFT at the level of B3LYP with basis set 6-31+g(d,p). ^{1}H NMR resonances have been predicted by GIAO method considering THF(CPCM) as solvent model.

Neutral PLY(O,O)-K complex (1a):

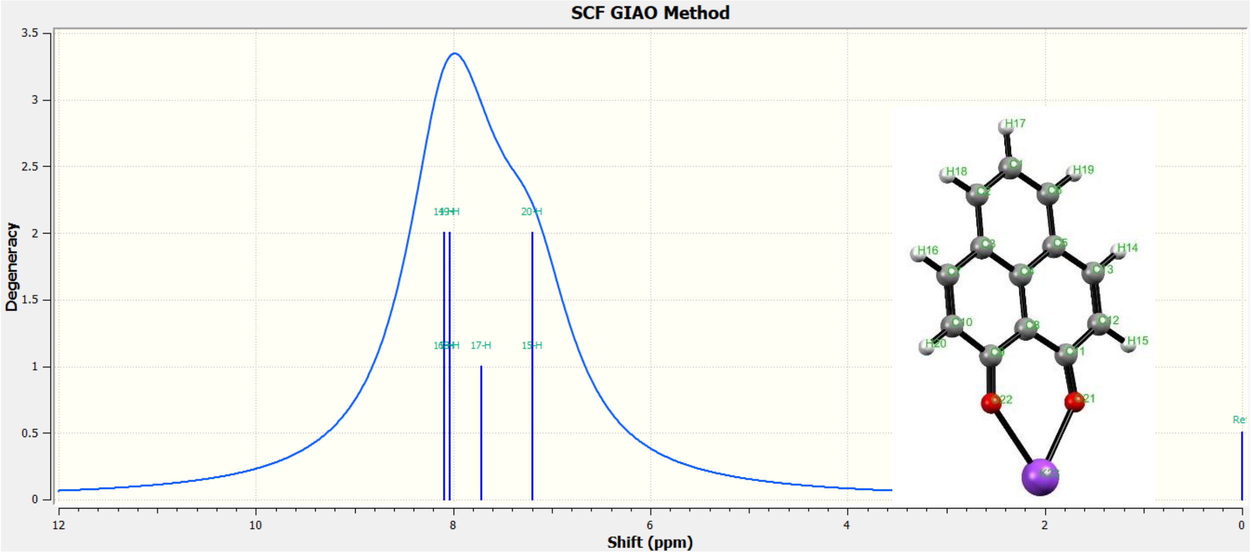

Doubly-reduced PLY(O,O)-K complex:

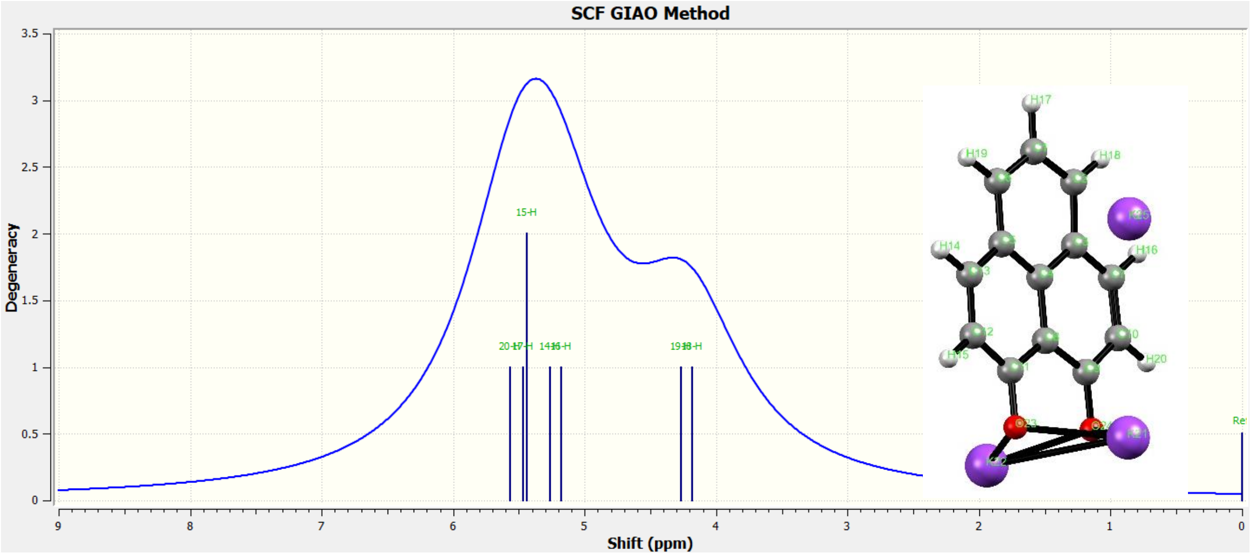

7. Electrostatic potential maps of PLY(N,O)-K:

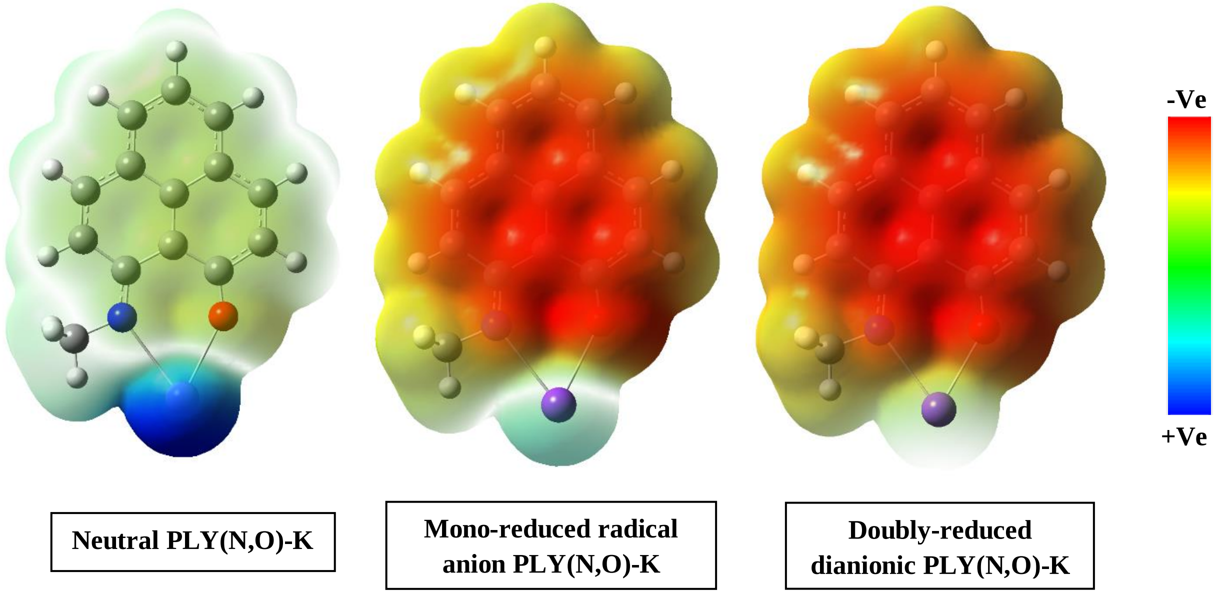

Figure S22: Electrostatic potential maps of three redox states of PLY(N,O)-K complex (Isovalue = 0.002).

8. Anisotropic Induced Current Density (AICD) plots:

a) Neutral PLY(O,O)-K complex (**1a**):

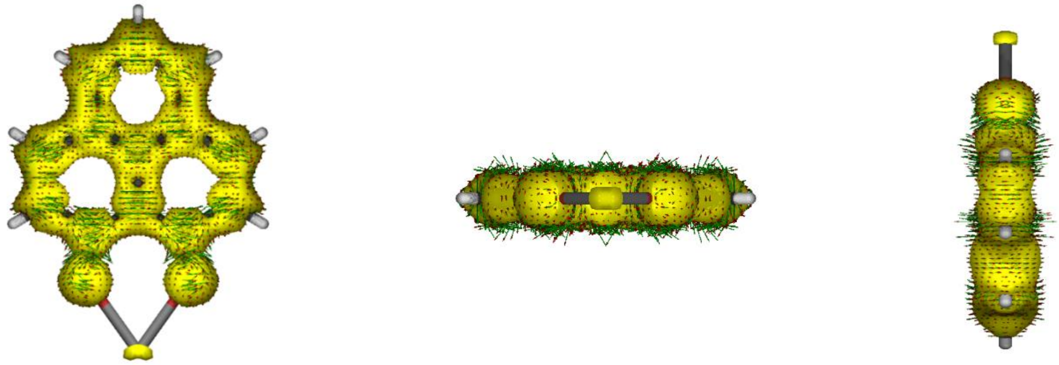

b) Mono-reduced PLY(O,O)-K complex:

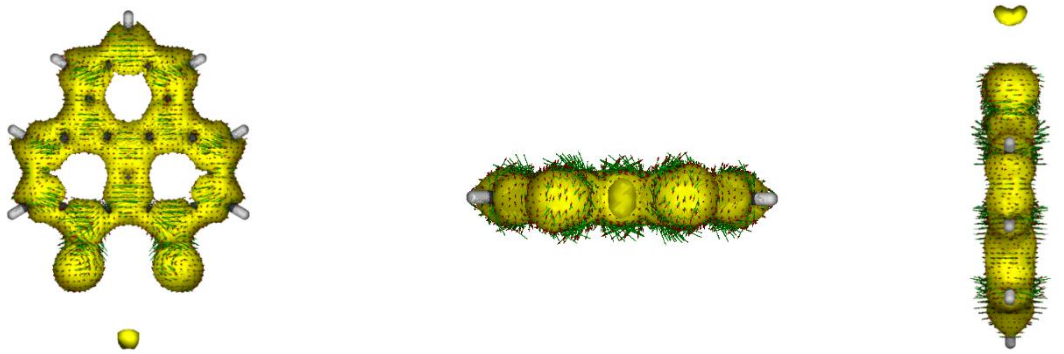

c) Doubly-reduced PLY(O,O)-K complex:

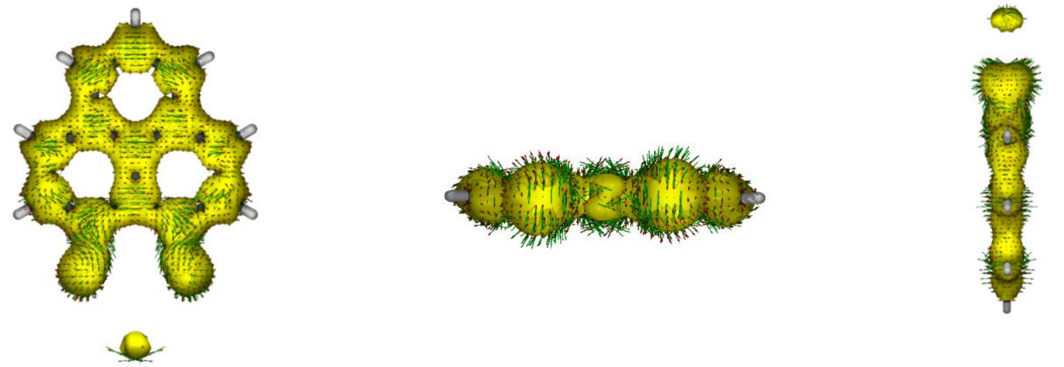

Figure S23. Three different views of ACID plots of different redox states of PLY(O,O)-K complex, a) For neutral PLY(O,O)-K complex (**1a**); b) For mono-reduced radical PLY (O,O)-K complex; c) For doubly-reduced PLY-(O,O)-K complex.

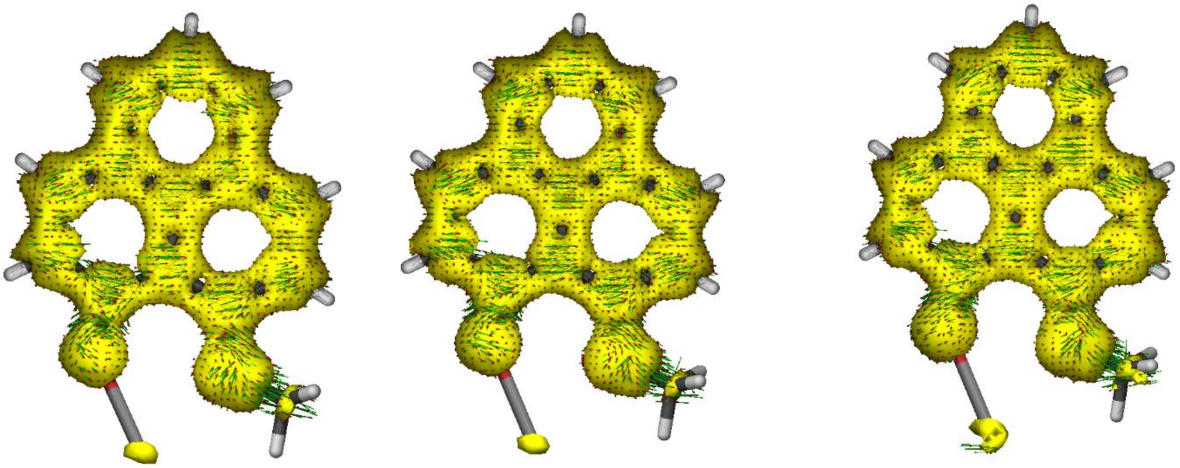

Neutral PLY(N,O)-K

Mono-reduced radical anion PLY(N,O)-K

Doubly-reduced dianion PLY(N,O)-K

Figure S24: ACID plot of and PLY(N,O)-K complex (**1b**) in three different redox states.

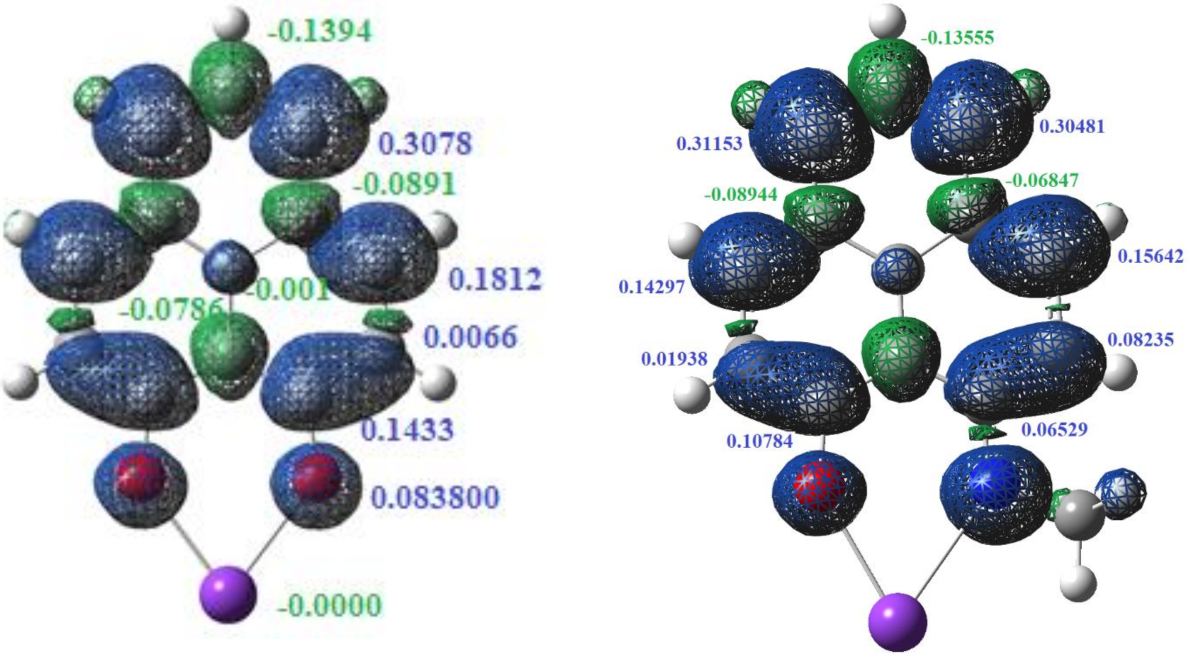

Mono-reduced radical anion PLY(O,O)-K

Mono-reduced radical anion PLY(N,O)-K

Figure S25. Spin density plots of mono-reduced PLY moieties.

9. Model example for Nuclear Independent Chemical Shift (NICS) calculation:

I. NICS calculation for the top aromatic ring of PLY(O,O)-K (1a):

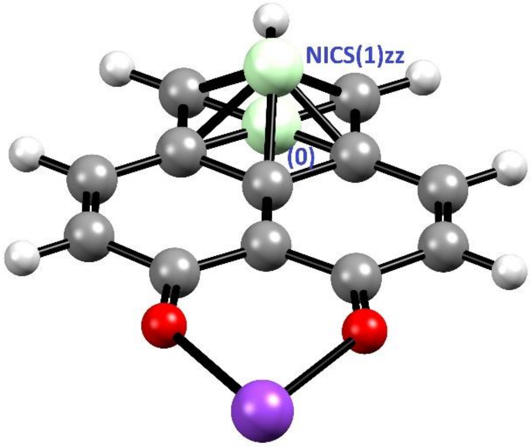

PLY(O,O)-K neutral:

NICS(1)zz = 25.0 ppm

PLY(O,O)-K anion:

NICS(1)zz = 16.9 ppm

PLY(O,O)-K dianion:

NICS(1)zz = 6.6 ppm

II. NICS calculation for the two equivalent aromatic ring (side rings) of PLY(O,O)-K (1a):

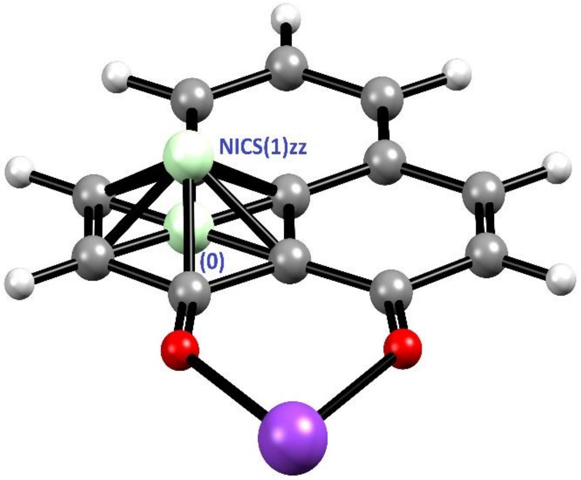

PLY(O,O)-K neutral:

NICS(1)zz = 9.6 ppm

PLY(O,O)-K anion:

NICS(1)zz = 8.9 ppm

PLY(O,O)-K dianion:

NICS(1)zz = 7.4 ppm

SCF GIAO Magnetic shielding tensor (ppm):

1.

a) PLY(O,O)-K (top ring):

6. Bq (0) Isotropic = 8.1380 Anisotropy = 0.6068

XX= 8.5419 YX= -0.0215 ZX= -0.0331

XY= 0.0022 YY= 7.9282 ZY= 0.0833

XZ= 0.0013 YZ= 0.0797 ZZ= 7.9440

Eigenvalues: 7.8542 8.0173 8.5425

7. Bq Isotropic = 10.3276 Anisotropy = 22.4018

XX= 24.9996 YX= 3.5564 ZX= 1.9117

XY= 0.6070 YY= 3.1251 ZY= 0.2707

XZ= 0.4828 YZ= 0.2647 ZZ= 2.8581

Eigenvalues: 2.6920 3.0287 25.2621

b) PLY(O,O)-K (side rings).

6 Bq Isotropic = 2.1849 Anisotropy = 12.8656

XX= -8.0601 YX= -0.1569 ZX= -0.0102

XY= -0.1674 YY= 10.7372 ZY= -0.8200

XZ= 0.0030 YZ= 0.0181 ZZ= 3.8776

Eigenvalues: -8.0615 3.8543 10.7620

7 Bq Isotropic = 5.3896 Anisotropy = 9.4100

XX= 9.5909 YX= 4.3790 ZX= 4.6344

XY= 1.0645 YY= 4.6776 ZY= -0.2043

XZ= 1.6222 YZ= 0.2760 ZZ= 1.9002

Eigenvalues: 0.5656 3.9402 11.6629

2.a) Mono-reduced PLY(O,O)-K (top ring)

6 Bq Isotropic = 5.1816 Anisotropy = 5.2957

XX= -1.1644 YX= -0.1149 ZX= -0.0231

XY= -0.3037 YY= 8.1521 ZY= -0.2903

XZ= 0.4094 YZ= -0.2811 ZZ= 8.5571

Eigenvalues: -1.1727 8.0054 8.7121

7 Bq Isotropic = 7.5944 Anisotropy = 14.4618

<u>XX= 16.9327</u> YX= 2.8929 ZX= 1.8749

XY= 0.2226 YY= 2.9397 ZY= 0.0492

XZ= 0.8646 YZ= 0.1579 ZZ= 2.9108

Eigenvalues: 2.7268 2.8207 17.2356

b) Mono-reduced PLY(O,O)-K (side rings):

6 Bq Isotropic = 2.2606 Anisotropy = 12.9684

XX= -8.7395 YX= 0.2155 ZX= 0.4257

XY= 0.0326 YY= 10.8219 ZY= -0.9934

XZ= 0.1245 YZ= -0.4488 ZZ= 4.6993

Eigenvalues: -8.7461 4.6217 10.9062

7 Bq Isotropic = 5.2616 Anisotropy = 8.1911

<u>XX= 8.8841</u> YX= 3.6864 ZX= 3.7492

XY=   1.0212   YY=   4.3829   ZY=   -0.1436

XZ=   1.7475   YZ=   0.4858   ZZ=   2.5177

Eigenvalues: 1.3187   3.7437   10.7223

3.

a) Doubly-reduced PLY(O,O)-K (top ring):

6 Bq   Isotropic =   1.0290   Anisotropy =   12.5250

XX=   -13.5032   YX=   -0.3105   ZX=   0.1891

XY=   -0.8729   YY=   7.7619   ZY=   -0.9362

XZ=   1.2629   YZ=   -0.8651   ZZ=   8.8282

Eigenvalues: -13.5416   7.2495   9.3790

7 Bq   Isotropic =   3.8663   Anisotropy =   5.4113

XX=   6.5940   YX=   2.2258   ZX=   1.9996

XY=   -0.1141   YY=   2.3540   ZY=   -0.3106

XZ=   1.6413   YZ=   -0.0240   ZZ=   2.6508

Eigenvalues: 1.5162   2.6088   7.4738

b) Doubly-reduced PLY(O,O)-K (side rings):

6 Bq(0)   Isotropic =   2.1511   Anisotropy =   12.8986

XX=   -9.7551   YX=   0.7836   ZX=   1.2722

XY=   0.2615   YY=   10.6000   ZY=   -1.0092

XZ=   0.2191   YZ=   -0.7020   ZZ=   5.6084

Eigenvalues: -9.8068   5.5099   10.7502

7 Bq(1) Isotropic = 4.8140 Anisotropy = 6.9933

XX= 7.3866 YX= 2.8677 ZX= 2.8169

XY= 0.9055 YY= 3.6386 ZY= 0.1098

XZ= 2.3355 YZ= 2.1437 ZZ= 3.4168

Eigenvalues: 2.1096 2.8562 9.4762

10. Theoretical calculations for all reduction processes:

The theoretical calculation for the different redox process have been carried out by DFT with B3LYP of calculation using 6-31g(d) basis set.

a. Gibbs free energy change for the mono-reduced PLY(O,O)-K complex preparation from neutral PLY(O,O)-K complex.

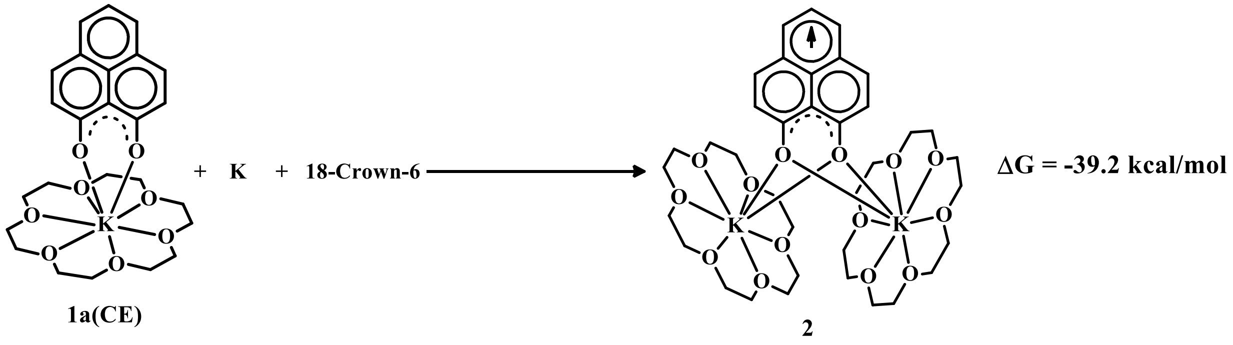

b. Gibbs free energy change for the doubly-reduced PLY(O,O)-K complex preparation from mono-reduced PLY(O,O)-K complex.

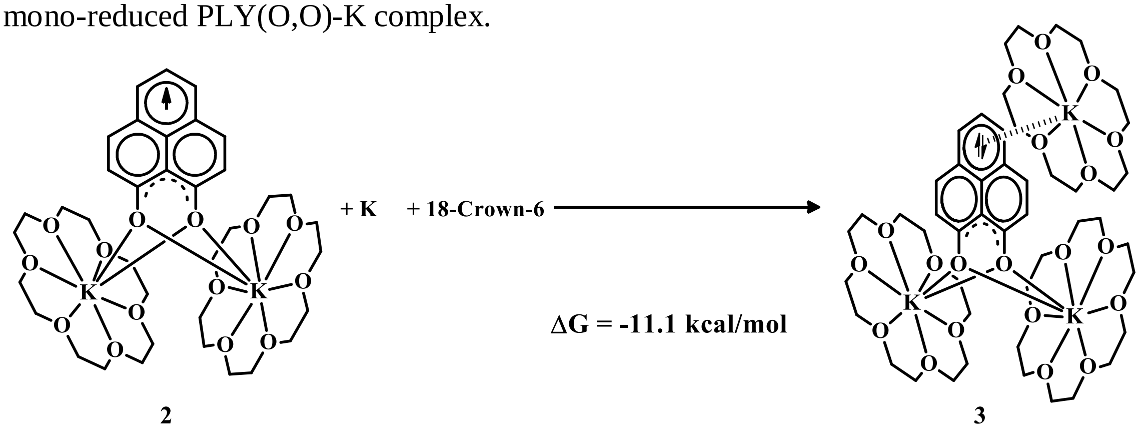

Scheme S1: Gibbs free energy change for different reduction processes. a) For mono reduction; b) for double reduction of PLY(O,O)-K(18-crown-6) complex.

11. Optimized geometries of three different redox states of PLY(O,O)-K complex:

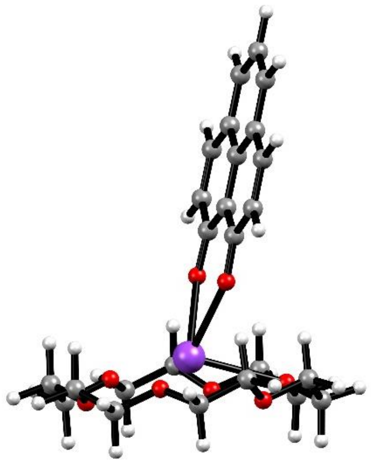

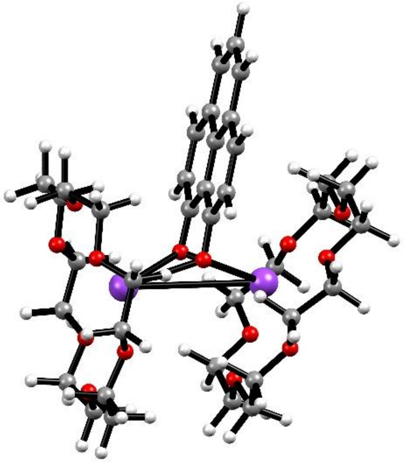

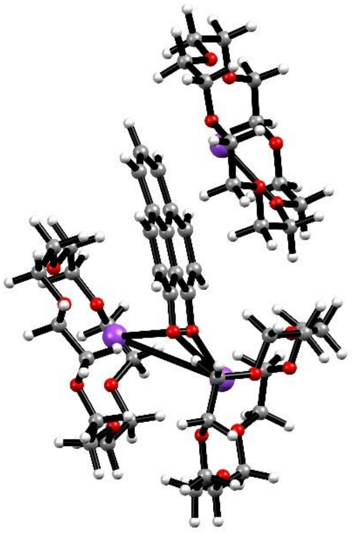

[PLY-K(18-Crown-6)](1a(CE)) **[PLY-K(18-Crown-6)$_2$](2)** **[PLY-K(18-Crown-6)$_3$](3)**

Table S1: Energies, enthalpies, and free energies (in Hartree) of the structures calculated with B3LYP/6-31G(d).

Structure	ZPE	ΔE	ΔH	ΔG	E	H	G	IF(cm^{-1})	Infrared
**PLY-Kc**	0.53557	0.56889	0.56984	0.46855	-2172.52029	-2172.51934	-2172.62063		
**K**	0.00000	0.00141	0.00236	-0.0158	-599.88896	-599.88801	-599.90620		
**Crown**	0.37011	0.38972	0.39066	0.32065	-922.58725	-922.58631	-922.65632		
**PLY-2Kc**	0.90639	0.96278	0.96372	0.81065	-3695.09349	-3695.09254	-3695.24562		
**PLY-3Kc**	1.27698	1.35680	1.35775	1.15323	-5217.62234	-5217.62140	-5217.82592		

12. DFT calculation for the quenching experiment by HCl treatment of doubly-reduced PLY(O,O)-K complex (3):

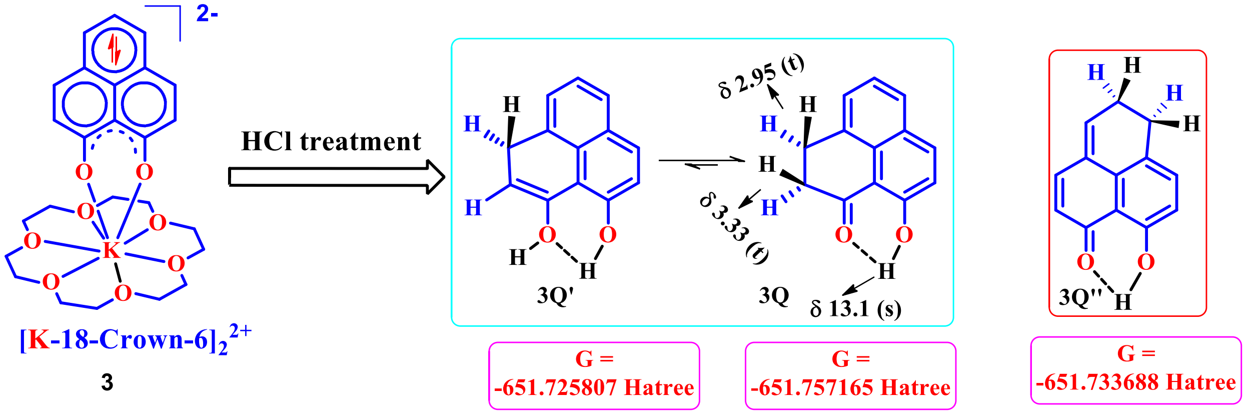

ΔG = -19.7 kcal/mol

Optimized Structures:

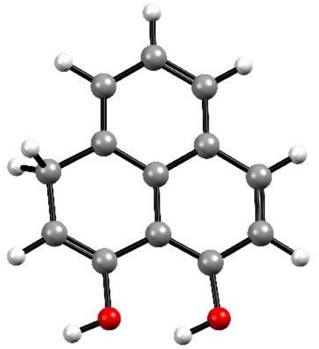

PLY-H (OH,OH)
3Q’

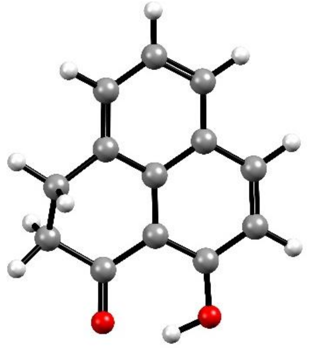

PLY-H₂ (O,OH)1
3Q

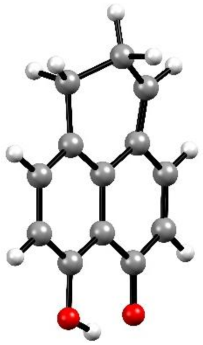

PLY-H₂ (O,OH)2
3Q’’

Table S2: Energies, enthalpies, and free energies (in Hartree) of the structures calculated with B3LYP/6-31G(d).

Structure	ZPE	ΔE	ΔH	ΔG	E	H	G	IF(cm^{-1})	Infrared
**PLY-H**	0.19658	0.20781	0.20876	0.15966	-651.67765	-651.67670	-651.72580		
**PLY-H$_2$1**	0.19792	0.20850	0.20944	0.16188	-651.71054	-651.70959	-651.75716		
**PLY-H$_2$2**	0.19721	0.20796	0.20891	0.16101	-651.68673	-651.68578	-651.73368		

13. Frontier molecular orbital diagrams of three different redox states of K-phenalenyl complex:

a) Neutral PLY(O,O)-K complex (**1a**).

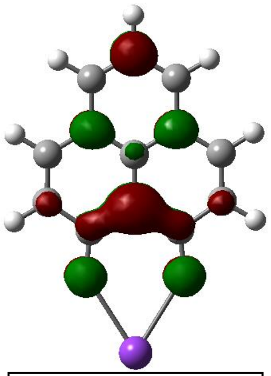

HOMO (-5.48 eV)

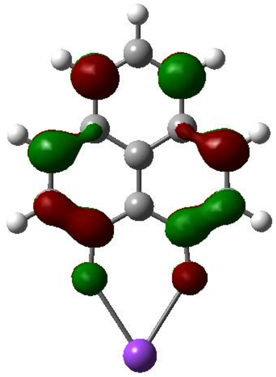

LUMO (-2.07 eV)

b) Mono-reduced PLY(O,O)-K complex (**II**).

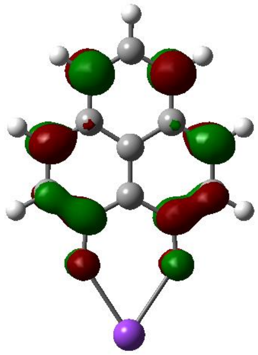

SOMO (-2.64 eV)

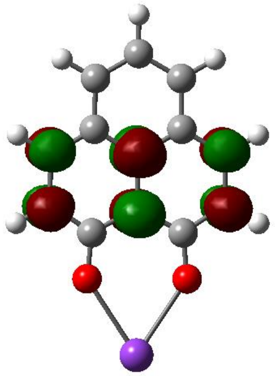

LUMO (-0.25 eV)

c) Doubly-reduced PLY(O,O)-K complex (**III**):

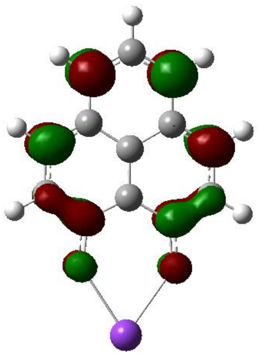

HOMO (-1.75 eV)

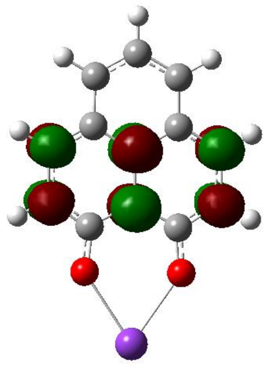

LUMO (-0.18 eV)

Figure S26. Frontier molecular orbital diagrams and energies of three different redox states of PLY(O,O)-K complex (**1a**).

a) Neutral PLY(N,O)-K complex(**1b**).

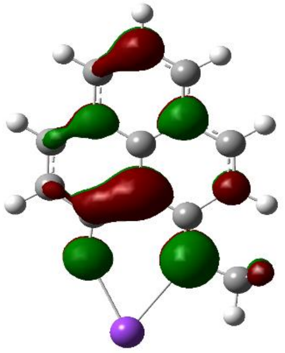

HOMO (-4.96 eV)

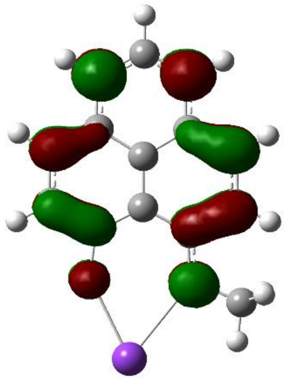

LUMO (-1.87 eV)

b) Mono-reduced PLY(N,O)-K complex.

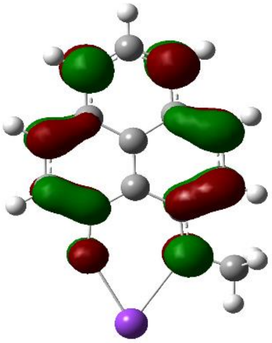

SOMO (-2.45 eV)

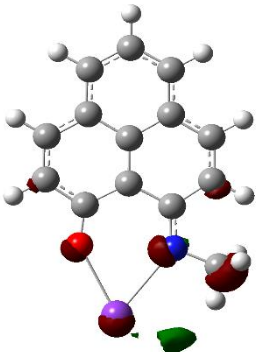

LUMO (-0.27 eV)

c) Doubly-reduced PLY(N,O)-K complex:

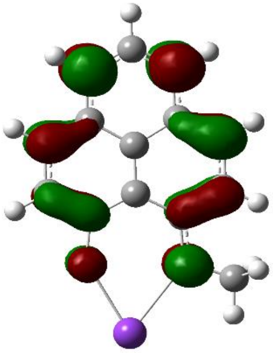

HOMO (-1.68 eV)

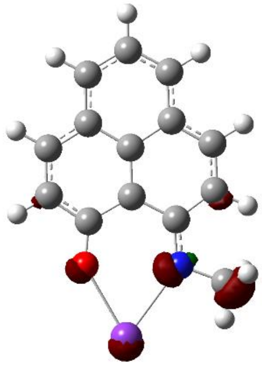

LUMO (-0.20 eV)

Figure S27. Frontier molecular orbital diagrams and energies of three different redox states of PLY(N,O)-K complex (**1b**).

14. Crystallographic and data collection parameters for 1a(CE), 1b(CE), 2:

X-ray crystallographic details:

Suitable single crystals of **1a(CE)**, **1b(CE)** and **2** were selected and mounted under nitrogen atmosphere using the X-TEMP2 and intensity data were collected on a Super Nova, Dual, Cu at zero, Eos diffractometer. Both the crystals were kept at 100 K during data collection. Using Olex2 [10], the structure was solved with the ShelX[11] structure solution program using Intrinsic Phasing and refined with the ShelXL[11] refinement package using Least Squares minimisation. All nonhydrogen atoms were refined with anisotropic displacement parameters. Crystallographic data (including structure factors) for the structures have been deposited with the Cambridge Crystallographic Data Centre. Copies of the data can be obtained free of charge from The Cambridge Crystallographic Data Centre *via* www.ccdc.cam.ac.uk/data_request/cif. CCDC 1991245, 2015680 and 1991246 contains the supplementary crystallographic data of compounds **1a(CE)**, **1b(CE)** and **2** respectively for this paper.

Table S3: Crystal data and structure refinement for PLY(O,O)-K[18-crown-6] (1a(CE)).

CCDC	1991245
Empirical formula	$C_{25}H_{31}KO_8$
Formula weight	498.60
Temperature/K	100.00(10)
Crystal system	Monoclinic
Space group	$P2_1/c$
a/Å	8.3399(4)
b/Å	27.1397(12)
c/Å	11.0226(5)
α/°	90
β/°	100.071(4)
γ/°	90
Volume/Å^3	2456.4(2)
Z	4
ρ_{calc}g/cm^3	1.348
μ/mm^{-1}	2.296
F(000)	1056.0
Crystal size/mm^3	0.3 × 0.15 × 0.1
Radiation	CuKα (λ = 1.54184)
2Θ range for data collection/°	6.514 to 132.732

Index ranges	$-8 \leq h \leq 9$, $-32 \leq k \leq 31$, $-12 \leq l \leq 13$
Reflections collected	16231
Independent reflections	4218 [R_{int} = 0.0429, R_{sigma} = 0.0345]
Data/restraints/parameters	4218/2/295
Goodness-of-fit on F^2	1.045
Final R indexes [I>=2σ (I)]	R_1 = 0.1134, wR_2 = 0.3001
Final R indexes [all data]	R_1 = 0.1199, wR_2 = 0.3068
Largest diff. peak/hole / e $Å^{-3}$	1.52/-0.78

Table S4: Crystal data and structure refinement for PLY(N,O)-K[18-crown-6] (1b(CE)).

Identification code	JASI_NOPLY
Empirical formula	$C_{66}H_{81}K_2N_2O_{14}$
Formula weight	1204.52
Temperature/K	100.00(10)
Crystal system	Monoclinic
Space group	$P2_1/c$
a/Å	14.2195(10)
b/Å	34.5380(19)
c/Å	13.8905(9)
α/°	90
β/°	113.176(8)
γ/°	90
Volume/Å^3	6271.3(8)
Z	4
ρ_{calc}g/cm^3	1.276
μ/mm^{-1}	1.874
F(000)	2564.0
Crystal size/mm^3	0.25 × 0.2 × 0.15
Radiation	CuKα (λ = 1.54184)
2Θ range for data collection/°	6.762 to 133.364

Index ranges	$-16 \leq h \leq 16$, $-41 \leq k \leq 39$, $-16 \leq l \leq 15$
Reflections collected	39527
Independent reflections	10947 [R_{int} = 0.0885, R_{sigma} = 0.0638]
Data/restraints/parameters	10947/0/712
Goodness-of-fit on F^2	1.053
Final R indexes [I>=2σ (I)]	R_1 = 0.1198, wR_2 = 0.3097
Final R indexes [all data]	R_1 = 0.1510, wR_2 = 0.3471
Largest diff. peak/hole / e $Å^{-3}$	0.90/-0.75
CCDC number	2015680

Table S5: Crystal data and structure refinement for PLY(O,O)-K_2[18-crown-6]$_2$ (2).

CCDC	1991246
Empirical formula	$C_{51}H_{69}K_2O_{14}$
Formula weight	984.26
Temperature/K	100.00(10)
Crystal system	Orthorhombic
Space group	$P2_12_12_1$
a/Å	14.23700(10)
b/Å	18.7126(2)
c/Å	19.1013(2)
α/°	90
β/°	90
γ/°	90
Volume/Å^3	5088.80(8)
Z	4
ρ_{calc}g/cm^3	1.285
μ/mm^{-1}	2.176
F(000)	2100.0
Crystal size/mm^3	0.25 × 0.15 × 0.1
Radiation	CuKα (λ = 1.54184)
2Θ range for data collection/°	6.612 to 132.292
Index ranges	$-16 \leq h \leq 16$, $-22 \leq k \leq 22$, $-18 \leq l \leq 22$

Reflections collected	34892
Independent reflections	8827 [R_{int} = 0.0273, R_{sigma} = 0.0212]
Data/restraints/parameters	8827/0/606
Goodness-of-fit on F^2	1.028
Final R indexes [I>=2σ (I)]	R_1 = 0.0407, wR_2 = 0.1069
Final R indexes [all data]	R_1 = 0.0415, wR_2 = 0.1077
Largest diff. peak/hole / e $Å^{-3}$	0.55/-0.43
Flack parameter	0.450(2)

15. Reaction Optimization for direct C-H arylation of N-methyl pyrrole with 4-chlorobenzonitrile coupling partner:

Catalyst PLY(O,O)-K (0.024 mmol) and reducing agent (0.06 mmol) were taken in 1.2 mL solvent in a 25 mL pressure tube. This mixture was allowed to stir at room temperature for 30 mins. N-methylpyrrole (1.2 mmol), 4-chlorobenzonitril (0.24 mmol) and base (0.48 mmol) were added to the resulting solution of catalyst inside a nitrogen filled glovebox. The final reaction mixture was allowed to stir for appropriate time at room temperature. After completion of the reaction, product was extracted in 25 mL ethylacetate and dried over anhydrous sodium sulphate. The solvent was removed under reduced pressure to get the crude product. Reaction conversion was calculated by ^{1}H NMR spectrum of the crude reaction mixture using 1,4-dimethoxybenzene as the internal standard.

Table S6: Reaction optimization table.

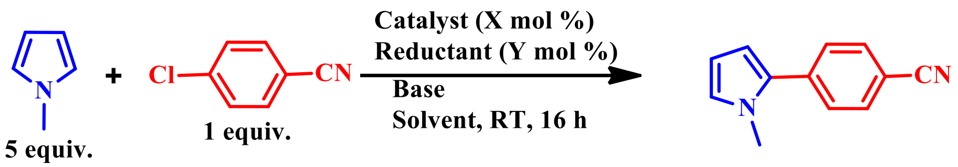

Entry	Catalyst (mol %)	Reductant (mol%)	Base (equiv.)	Solvent	Yield
1	PLY(O,O)-K (5)	K (15)	KO^{t}Bu (2)	DMSO	12%
2	PLY(O,O)-K (10)	K (25)	KO^{t}Bu (2)	DMSO	15%
**3**	**PLY(O,O)-K (10)**	**K (25)**	**KO^{t}Bu (2)**	**DMF**	**54%(51%[a])**

4	PLY(O,O)-K (10)	K (25)	KO^{t}Bu (2)	DMAc	40%
5	PLY(O,O)-K (10)	-	KO^{t}Bu (2)	DMF	10%
6	-	K (25)	KO^{t}Bu (2)	DMF	23%
7	Fe(PLY(O,O))$_3$ (10)	K (30)	KO^{t}Bu (2)	DMF	<10%
8	PLY(O,O)-K (10)	K (15)	KO^{t}Bu (2)	DMAc	25%
9	PLY-O,O-K (10)	K (25)	KO^{t}Bu (2)	THF	0 %
10	PLY(O,O) cat (10)	K (25)	KO^{t}Bu (2)	DMF	<10 %
11[b] 12[c]	PLY(O,O)-K (10) PLY(O,O)-K (10)	K (25) K (25)	KO^{t}Bu (2) KO^{t}Bu (2)	DMF DMF	52 % <2%
13	PLY(N,O)-K (10)	K (25)	KOtBu (2)	DMF	67%
14	-	K (1.5 equiv)	KO^{t}Bu (2)	DMF	30%

[a] Isolated yield; [b] Reaction was carried out under dark condition; [c]The reaction was performed with chlorobenzene coupling partner.

16. C-H arylation of arenes/heteroarenes with 4-chlorobenzonitrile in absence of catalyst.

Potassium (0.06 mmol), N-methylpyrrole (1.2 mmol), 4-chlorobenzonitril (0.24 mmol) and KO^{t}Bu (0.48 mmol) were taken in 1.2 mL n,n-dimethylformamide in a 25 mL pressure tube inside an argon filled glovebox. The final reaction mixture was allowed to stir for appropriate 5time at room

temperature. After completion of the reaction, product was extracted in 25 mL ethylacetate and dried over anhydrous sodium sulphate. The solvent was removed under reduced pressure to get the crude product. Reaction conversion was calculated by ^{1}H NMR spectrum of the crude reaction mixture using 1,4-dimethoxybenzene as the internal standard.

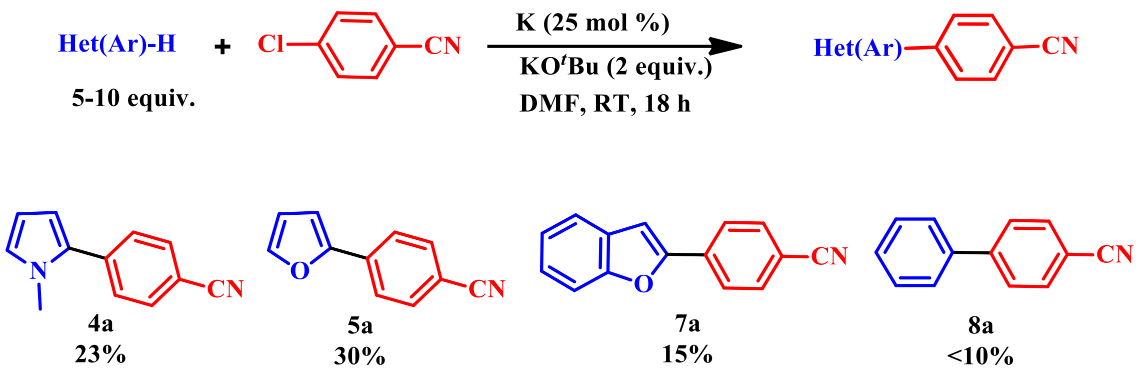

17. General procedure for C-H arylation of arenes/heteroarenes with aryl halides:

PLY(O,O)-K (0.024 mmol) and K (0.06 mmol) were taken in 1.2 mL DMF/DMSO solvent in a 25 mL pressure tube. This mixture was allowed to stir at room temperature for 30 mins. Arene/heteroarenes, aryl halide partner (0.24 mmol) and KO^{t}Bu (0.48 mmol) were added to the resulting solution of catalyst inside a nitrogen filled glovebox. After the final reaction mixture was allowed to stir for appropriate time at room temperature. After completion of the reaction, product was extracted in 25 mL ethylacetate and dried over anhydrous sodium sulphate. The solvent was removed under reduced pressure and crude product was purified by column chromatography over silica gel (100-200 mesh) using hexane/EtOAc mixture to yield the pure desired products.

18. General procedure for intramolecular coupling reactions:

In a typical method, PLY(O.O)-K (**1a**, 0.024 mmol) and K (0.06 mmol) were taken in 1.2 mL DMF in a 25 mL pressure tube. This mixture was allowed to stir at room temperature for 30 mins. Substrates (5as-5es, 0.24 mmol) and KO^{t}Bu (0.48 mmol) were added to the resulting solution of catalyst inside an argon filled glovebox. The final reaction mixture was allowed to stir for appropriate time at room temperature. After completion of the reaction, products were extracted in 25 mL ethylacetate and dried over anhydrous sodium sulphate. The solvent was removed under reduced pressure to get the crude products. NMR (^{1}H and ^{13}C) spectroscopic measurements of all the reaction mixtures were carried out to characterize the products and the conversion were calculated from ^{1}H NMR peak intensities.

19. Radical scavenging experiment in presence of TEMPO:

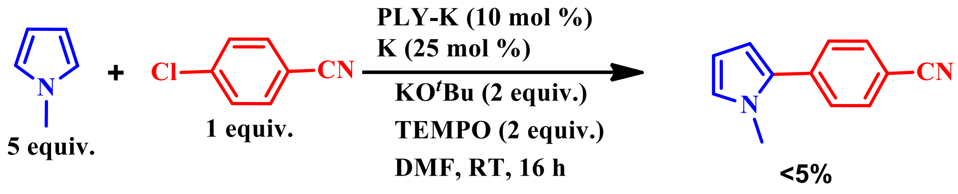

PLY(O,O)-K (**1a**, 0.024 mmol) and K (0.06 mmol) were taken in 1.2 mL DMF in a 25 mL pressure tube. This mixture was allowed to stir at room temperature for 30 mins. N-methylpyrrole (1.2 mmol), 4-chlorobenzonitril (0.24 mmol) and KO^{t}Bu (0.48 mmol) were added to the resulting solution of catalyst inside a nitrogen filled glovebox. TEMPO (0.48 mmol) was added in the resulting reaction mixture. The final reaction mixture was allowed to stir for appropriate time at room temperature. After completion of the reaction, product was extracted in 25 mL ethylacetate and dried over anhydrous sodium sulphate. The solvent was removed under reduced pressure and crude product was subjected for ^{1}H NMR spectroscopic measurement.

20. Radical trapping experiment:

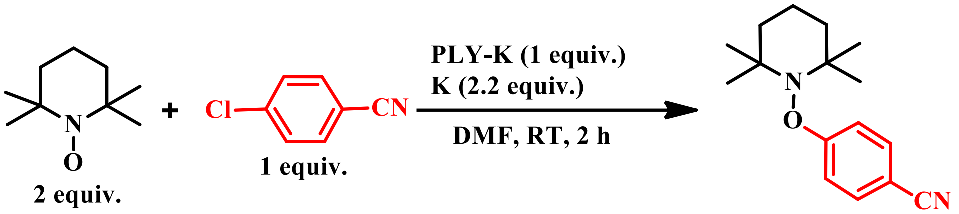

PLY(O,O)-K (**1a**, 0.24 mmol) and K (0.53 mmol) were taken in 1.2 mL DMF in a 25 mL pressure tube. This mixture was allowed to stir at room temperature for 30 min, 4-chlorobenzonitril (0.24 mmol) was added to the resulting solution of catalyst inside an argon filled glovebox. TEMPO (0.48 mmol) was added in the resulting reaction mixture. The final reaction mixture was allowed to stir for 2h at room temperature. After completion of the reaction, product was extracted in 25 mL ethylacetate and dried over anhydrous sodium sulphate. The mass spectroscopic measurement of this crude reaction mixture was carried out in acetonitrile solvent (Figure 98).

21. Catalyst recovery after catalytic reaction:

PLY(O,O)-K (0.107 mmol) and K (0.23 mmol) were taken in 2 mL DMF/DMSO solvent in a 25 mL pressure tube. This mixture was allowed to stir at room temperature for 30 mins. N-methylpyrrole (5.3 mmol), 4-chlorobenzonitriler (1.07 mmol) and KO^{t}Bu (0.214 mmol) were added to the resulting solution of catalyst inside a nitrogen filled glovebox. After the final reaction mixture was allowed to stir for appropriate time at room temperature. After completion of the reaction, product was extracted in 25 mL ethylacetate and dried over anhydrous sodium sulphate. The solvent was removed under reduced pressure. The phenalenyl parts were collected together from column chromatography with 10% ethylacetate in hexane mixture. The ^{1}H NMR spectrum

of this residues (Figure S96) shows the presence of PLY(O,OH) along with other species. We could isolate 6 mg of PLY(O,OH) from this mixture which indicates ~30% recovery of the catalyst.

21. The analytical and spectral characterization data of the catalytic products

4-(1-methylpyrrole)benzonitrile (4a):[12]

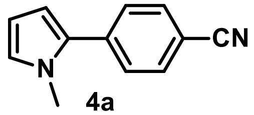

4a

The crude product was purified by column chromatography using silica gel (100-200 mesh) with 5% ethylacetate in hexane. The compound was obtained as a white solid.

^{1}**H NMR** (400 MHz, $CDCl_3$, 298K) δ (ppm) 3.72 (s, 3H), 6.23 (dd, 1H, J_1 = 4 Hz, J_2 = 2 Hz), 6.34 (m, 1H), 6.78 (t, 1H, J = 4 Hz), 7.50 (d, 2H, J = 8 Hz), 7.67 (d, 2H, J = 8 Hz).

^{13}**C{^{1}H} NMR** (100 MHz, $CDCl_3$, 298K) δ (ppm) 35.4, 108.5, 109.6, 110.7, 118.9, 125.8, 128.2, 132.2, 132.6, 137.6.

2-(4-Cyanophenyl)furan (5a): [13]

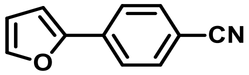

5a

The crude product was purified by column chromatography using silica gel (100-200 mesh) with hexane. The compound was obtained as a white solid.

^{1}**H NMR** (400 MHz, $CDCl_3$, 298K) δ (ppm) 6.52-6.54 (m, 1H), 6.81 (d, 1H, J = 2 Hz), 7.53 (s, 1H), 7.65 (d, 2H, J = 8 Hz), 7.74 (d, 2H, J = 8 Hz).

^{13}**C{^{1}H} NMR** (100 MHz, $CDCl_3$, 298K) δ (ppm) 108.1, 110.2, 112.2, 118.9, 123.9, 132.5, 134.6, 143.6, 151.9.

2-(4-Cyanophenyl)thiophene (6a):[13]

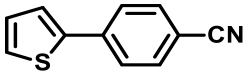

6a

The crude product was purified by column chromatography using silica gel (100-200 mesh) with 2% ethylacetate in hexane. The compound was obtained as a white solid.

^{1}**H NMR** (400 MHz, $CDCl_3$, 298K) δ (ppm) 7.13 (t, 1H, J = 4 Hz), 7.40-7.43 (m, 2H), 7.64-7.71 (m, 4H).

^{13}**C{^{1}H} NMR** (100 MHz, $CDCl_3$, 298K) δ (ppm) 110.6, 118.8, 125.1, 126.1, 127.0, 128.6, 132.7, 138.6, 142.1.

4-(benzofuran-2-yl)benzonitrile (7a):[14]

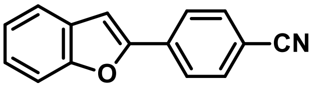

7a

The crude product was purified by column chromatography using silica gel (100-200 mesh) with 5% ethylacetate in hexane. The compound was obtained as a white solid.

^{1}**H NMR** (400 MHz, $CDCl_3$, 298K) δ (ppm) 7.18 (s, 1H), 7.27 (d, 1H, J = 8 Hz), 7.35 (t, 1H, J = 8 Hz), 7.55 (m, 1H), 7.63 (d, 1H, J = 8 Hz), 7.73 (d, 2H, J = 9 Hz), 7.95 (d, 2H, J = 8 Hz).

^{13}**C{^{1}H} NMR** (100 MHz, $CDCl_3$, 298K) δ (ppm) 104.5, 111.5, 111.6, 118.7, 121.5, 123.4, 125.1, 125.6, 128.6, 132.6, 134.4, 153.5, 155.2.

4-Cyanobiphenyl (8a): [13]

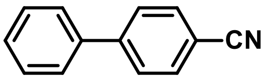

8a

The crude product was purified by column chromatography using silica gel (100-200 mesh) with hexane. The compound was obtained as a white solid.

^{1}H NMR (400 MHz, $CDCl_3$, 298K) δ (ppm) 7.41 (d, 1H, J = 8 Hz), 7.47 (t, 2H, J = 7 Hz), 7.59 (d, 2H, J = 8 Hz), 7.70 (q, 4H, J = 8.2 Hz).

^{13}C{^{1}H} NMR (100 MHz, $CDCl_3$, 298K) δ (ppm) 110.9, 118.9, 127.2, 127.7, 128.6, 129.1, 132.6, 139.2, 145.7.

1-Cyano-4-(2, 4-dimethylbenzene) (9a):[15]

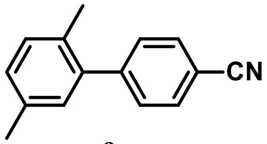

9a

The crude product was purified by column chromatography using silica gel (100-200 mesh) with hexane. The compound was obtained as a white solid.

^{1}H NMR (400 MHz, DMSO-d6$_3$, 298K) δ (ppm) 2.21 (s, 3H), 2.36 (s, 3H), 7.01 (s, 1H), 7.13 (d, 1H, J = 3 Hz), 7.18 (d, 1H, J = 4 Hz), 7.42 (dd, 2H, J_1 = 8 Hz, J_2 = 2Hz), 7.69 (dd, 2H, J_1 = 8 Hz, J_2 = 2 Hz).

^{13}C{^{1}H} NMR (100 MHz, DMSO-d6, 298K) δ (ppm) 19.8, 20.8, 110.6, 118.9, 129.0, 129.9, 130.0, 130.6, 131.9, 135.6, 139.8, 147.0.

1-Cyano-4-mesitylbenzene (10a): [13]

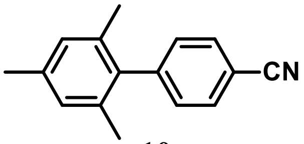

10a

The crude product was purified by column chromatography using silica gel (100-200 mesh) with hexane. The compound was obtained as a white solid.

^{1}H NMR (400 MHz, $CDCl_3$, 298K) δ (ppm) 1.98 (s, 6H), 2.34 (s, 3H), 6.96 (s, 2H), 7.27 (d, 2H, *J* = 8 Hz), 7.70 (d, 2H, *J* = 8 Hz).

^{13}C{^{1}H} NMR (100 MHz, $CDCl_3$, 298K) δ (ppm) 20.5, 21.0, 110.6, 118.9, 128.3, 130.3, 132.3, 135.3, 137.1, 138.0, 146.4.

4-(1-methylpyrrole)anisole (4b): [16]

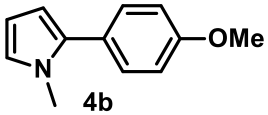

4b

The crude product was purified by column chromatography using silica gel (100-200 mesh) with 10% ethylacetate in hexane. The compound was obtained as a light yellow colored solid.

^{1}H NMR (400 MHz, $CDCl_3$, 298K) δ (ppm) 3.64 (s, 3H), 3.85 (s, 3H), 6.16-6.17 (m, 1H), 6.19-6.21 (m, 1H), 6.70 (t, 1H, *J* = 6 Hz), 6.95 (d, 2H, *J* = 8 Hz), 7.32 (d, 2H, *J* = 9 Hz).

^{13}C{^{1}H} NMR (100 MHz, $CDCl_3$, 298K) δ (ppm) 34.8,55.3, 107.5, 107.9, 113.7, 123.0, 125.9, 130.0, 134.3, 158.6.

4-(1-methylpyrrole)benzene (4c): [16]

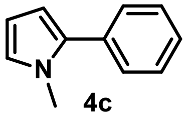

4c

The crude product was purified by column chromatography using silica gel (100-200 mesh) with 10% ethylacetate in hexane. The compound was obtained as a light yellow colored solid.

^{1}H NMR (400 MHz, $CDCl_3$, 298K) δ (ppm) 3.67 (s, 3H), 6.20-6.24 (m, 2H), 6.72 (t, 1H, J = 2 Hz), 7.29-7.32 (m, 1H), 7.40-7.42 (m, 4H).

^{13}C{^{1}H} NMR (100 MHz, $CDCl_3$, 298K) δ (ppm) 35.0, 107.7, 107.6, 123.6, 126.7, 128.3, 128.6, 133.3, 134.6.

4-(1-methylpyrrole)3, 5-dimethylbenzene (4d): [16]

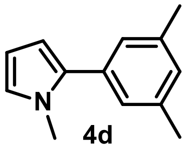

4d

The crude product was purified by column chromatography using silica gel (100-200 mesh) with 10% ethylacetate in hexane. The compound was obtained as a colorless soild.

^{1}H NMR (400 MHz, $CDCl_3$, 298K) δ (ppm) 2.35 (s, 3H), 3.66 (s, 3H), 6.18 (d, 2H, J = 4 Hz), 6.69 (t, 1H, J = 2 Hz), 6.95 (s, 1H), 7.02 (s, 2H).

^{13}C{^{1}H} NMR (100 MHz, $CDCl_3$, 298K) δ (ppm) 21.4, 35.0,107.6, 108.4, 123.3, 126.5, 128.4, 133.2, 134.8, 137.8.

4-(1-methylpyrrole)biphenyl (4e):[17]

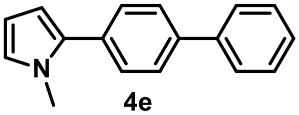

4e

The crude product was purified by column chromatography using silica gel (100-200 mesh) with 10% ethylacetate in hexane. The compound was obtained as a brown colored oil.

^{1}H NMR (400 MHz, $CDCl_3$, 298K) δ (ppm) 3.74 (s, 3H), 6.26 (t, 1H, *J* = 2 Hz), 6.31 (m, 1H), 6.77 (t, 1H, *J* = 1 Hz), 7.38 (t, 1H, *J* = 2 Hz), 7.46-7.52 (m, 4H), 7.65 (d, 4H, *J* = 8 Hz).

^{13}C{^{1}H} NMR (100 MHz, $CDCl_3$, 298K) δ (ppm) 35.1, 107.8, 108.8, 123.9, 127.0, 127.0, 127.3, 127.5, 127.9, 128.8, 128.8, 128.8, 131.4, 132.3, 134.2, 139.4, 140.6.

4-(1-methylpyrrole)toluene (4f):[18]

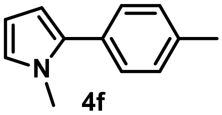

4f

The crude product was purified by column chromatography using silica gel (100-200 mesh) with 10% ethylacetate in hexane. The compound was obtained as a brown colored oil.

^{1}H NMR (400 MHz, $CDCl_3$, 298K) δ (ppm) 2.39 (s, 3H), 3.66 (s, 3H), 6.20 (d, 2H, *J* = 2 Hz), 6.71 (t, 1H, *J* = 2 Hz), 7.21 (d, 2H, *J* = 8 Hz), 7.31 (d, 2H, *J* = 8 Hz).

^{13}C{^{1}H} NMR (100 MHz, $CDCl_3$, 298K) δ (ppm) 21.1, 34.9, 107.6, 108.2, 123.3, 128.6, 129.0, 130.4, 134.6, 136.5.

2-(4-Methoxyphenyl)furan (5b):[14]

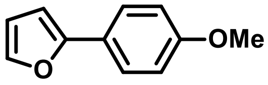

5b

The crude product was purified by column chromatography using silica gel (100-200 mesh) with 2% ethylacetate in hexane. The compound was obtained as a colorless solid.

^{1}H NMR (400 MHz, $CDCl_3$, 298K) δ (ppm) 3.82 (s, 3H), 6.43 (t, 1H, *J* = 4 Hz), 6.49 (d, 1H, *J* = 4 Hz), 6.91 (d, 2H, *J* = 8 Hz), 7.41 (s, 1H), 7.59 (d, 2H, *J* = 8 Hz).

^{13}C{^{1}H} NMR (100 MHz, $CDCl_3$, 298K) δ (ppm) 55.3, 103.3, 111.5, 114.1, 124.0, 125.2, 141.4, 154.0, 159.0.

2-Phenylfuran (5c):[14]

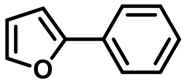

5c

The crude product was purified by column chromatography using silica gel (100-200 mesh) with hexane. The compound was obtained as a colorless solid.

^{1}H NMR (400 MHz, $CDCl_3$, 298K) δ (ppm) 6.47 (m, 1H), 6.65 (d, 1H, *J* = 5 Hz), 7.24-7.28 (m, 1H), 7.38 (t, 2H, *J* = 6 Hz), 7.68 (d, 2H, *J* = 8 Hz), 7.47 (s, 1H).

^{13}C{^{1}H} NMR (100 MHz, $CDCl_3$, 298K) δ (ppm) 105.0, 111.7, 123.9, 127.4, 128.7, 130.9, 142.1, 154.1.

2-(3, 5-Dimethlphenyl)furan (5d):[19]

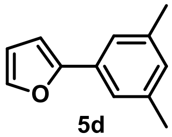

5d

The crude product was purified by column chromatography using silica gel (100-200 mesh) with 2% ethylacetate in hexane. The compound was obtained as a yellow colored oil.

^{1}H NMR (400 MHz, $CDCl_3$, 298K) δ (ppm) 2.35 (s, 6H), 6.45 (m, 1H), 6.60 (d, 1H, J = 4 Hz), 6.90 (s, 1H), 7.30 (s, 2H), 7.44 (s, 1H).

^{13}C{^{1}H} NMR (100 MHz, $CDCl_3$, 298K) δ (ppm) 21.3, 104.7, 111.5, 121.6, 129.1, 130.7, 138.2, 141.8, 154.3.

2-Biphenylfuran (5e):[20]

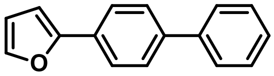

5e

The crude product was purified by column chromatography using silica gel (100-200 mesh) with 2% ethylacetate in hexane. The compound was obtained as a colorless solid.

^{1}H NMR (400 MHz, $CDCl_3$, 298K) δ (ppm) 6.5 (q, 1H, J_1 = 4Hz, J_2 = 1Hz), 6.7 (d, 1H, J = 4Hz), 7.45 (t, 2H, J = 8Hz), 7.50 (d, 1H, J = 2Hz), 7.63 (d, 4H, J = 8Hz), 7.75 (d, 2H, J = 8Hz).

^{13}C{^{1}H} NMR (100 MHz, $CDCl_3$, 298K) δ (ppm) 105.1, 111.7, 124.2, 126.9, 127.3, 128.8, 129.8, 139.9, 140.6, 142.1, 153.7.

2-(4-Methylphenyl)furan (5f):[14]

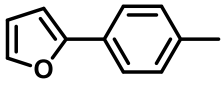

5f

The crude product was purified by column chromatography using silica gel (100-200 mesh) with 5% ethylacetate in hexane. The compound was obtained as a colorless solid.

^{1}H NMR (400 MHz, $CDCl_3$, 298K) δ (ppm) 2.35 (s, 3H), 6.44 (m, 1H), 6.57 (d, 1H, J = 4 Hz), 7.17 (d, 2H, J = 8 Hz), 7.43 (s, 1H), 7.55 (d, 2H, J = 8 Hz).

^{13}C{^{1}H} NMR (100 MHz, $CDCl_3$, 298K) δ (ppm) 21.2, 104.2, 111.5, 123.7, 128.2, 129.3, 137.1 141.6, 154.2.

2-(4-Methoxyphenyl)thiophene (6b):[14]

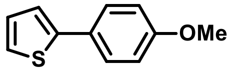

6b

The crude product was purified by column chromatography using silica gel (100-200 mesh) with 5% ethylacetate in hexane. The compound was obtained as a colorless solid.

^{1}H NMR (400 MHz, $CDCl_3$, 298K) δ (ppm) 3.84 (s, 3H), 6.92 (d, 2H, J = 8 Hz), 7.05 (dd, 1H, J_1 = 4 Hz, J_2 = 1 Hz), 7.21 (t, 2H. J = 4 Hz), 7.54 (d, 2H, J = 8 Hz).

^{13}C{^{1}H} NMR (100 MHz, $CDCl_3$, 298K) δ (ppm) 55.3, 114.2, 122.1, 123.8, 127.2, 127.4, 127.9, 144.3, 159.2.

2-Biphenylthiophene (6e):[21]

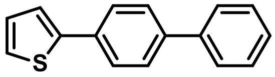

6e

The crude product was purified by column chromatography using silica gel (100-200 mesh) with 2% ethylacetate in hexane. The compound was obtained as a colorless solid.

^{1}H NMR (400 MHz, $CDCl_3$, 298K) δ (ppm) 7.10-7.12 (m, 1H), 7.31 (d, 1H, J = 6 Hz), 7.36-7.38 (m, 2H), 7.46 (t, 2H, J = 8 Hz), 7.62 (m, 4H), 7.70 (d, 2H, J = 9 Hz).

^{13}C{^{1}H} NMR (100 MHz, $CDCl_3$, 298K) δ (ppm) 123.1, 124.8, 126.3, 126.9, 127.4, 127.5, 128.0, 128.8, 133.4, 140.2, 140.5, 144.0.

2-(4-Methylphenyl)thiophene (6f):[14]

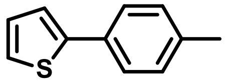

6f

The crude product was purified by column chromatography using silica gel (100-200 mesh) with hexane. The compound was obtained as a yellow colored oil.

^{1}H NMR (400 MHz, $CDCl_3$, 298K) δ (ppm) 2.36 (s, 3H), 7.06 (dd, 1H, J_1 = 7 Hz, J_2 = 4 Hz), 7.18 (d, 2H, J = 8 Hz), 7.23-7.27 (m, 2H), 7.51 (d, 2H, J = 8 Hz).

^{13}C{^{1}H} NMR (100 MHz, $CDCl_3$, 298K) δ (ppm) 21.1, 122.5, 124.3, 125.9, 127.9, 129.5, 131.6, 137.3, 144.6.

4-(benzofuran-2-yl)benzene (7b):[14]

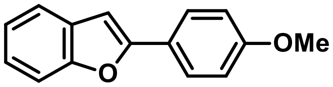

7b

The crude product was purified by column chromatography using silica gel (100-200 mesh) with 5% ethylacetate in hexane. The compound was obtained as a brown colored solid

^{1}H NMR (400 MHz, $CDCl_3$, 298K) δ (ppm) 3.87 (s, 3H), 6.89 (s, 1H), 6.98 (d, 2H, *J* = 8 Hz), 7.21 (m, 2H), 7.50 (d, 1H, *J* = 4 Hz), 7.55 (d, 1H, *J* = 4 Hz), 7.80 (d, 2H, *J* = 8 Hz).

^{13}C{^{1}H} NMR (100 MHz, $CDCl_3$, 298K) δ (ppm) 55.3, 99.6, 110.9, 114.2, 120.5, 122.8, 123.4, 123.7, 126.4, 129.5, 154.5, 156.0, 159.9.

4-(benzofuran-2-yl)benzene (7c): [14]

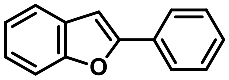

7c

The crude product was purified by column chromatography using silica gel (100-200 mesh) with 5% ethylacetate in hexane. The compound was obtained as a colorless solid

^{1}H NMR (400 MHz, $CDCl_3$, 298K) δ (ppm) 7.04 (s, 1H), 7.24-7.31 (m, 3H), 7.34 (t, 1H, *J* = 8 Hz), 7.44 (t, 2H, *J* = 9 Hz), 7.51 (d, 1H, *J* = 10 Hz), 7.59 (d, 1H, *J* = 8 Hz), 7.88 (d, 2H, *J* = 9 Hz).

^{13}C{^{1}H} NMR (100 MHz, $CDCl_3$, 298K) δ (ppm) 101.3, 111.1, 120.8, 122.9, 124.2, 124.9, 128.5, 128.7, 130.5, 131.0, 154.8, 155.9.

4-(benzofuran-2-yl)3, 5-dimethylbenzene (7d):[22]

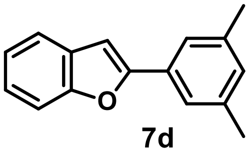

7d

The crude product was purified by column chromatography using silica gel (100-200 mesh) with 5% ethylacetate in hexane. The compound was obtained as a light colorless solid.

^{1}H NMR (400 MHz, $CDCl_3$, 298K) δ (ppm) 2.39 (s, 6H), 7.00 (s, 1H), 7.21-7.30 (m, 3 H), 7.51—7.53 (m. 3H), 7.57 (d, 1 H, *J* = 8 Hz).

^{13}C{^{1}H} NMR (100 MHz, $CDCl_3$, 298K) δ (ppm) 21.3, 101.0, 111.1, 120.8, 122.8, 122.8, 124.0, 129.3, 130.3, 130.4, 138.3, 154.8, 156.3.

4-(benzofuran-2-yl)biphenyl (7e):[23]

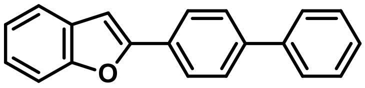

7e

The crude product was purified by column chromatography using silica gel (100-200 mesh) with 5% ethylacetate in hexane. The compound was obtained as a colorless solid

^{1}H NMR (400 MHz, $CDCl_3$, 298K) δ (ppm) 7.07 (s, 1H), 7.4-7.30 (m, 2H), 7.38 (t, 1H, *J* = 4 Hz), 7.45 (t, 2H, *J* = 8 Hz), 7.55 (d, 1H, *J* = 8 Hz), 7.60 (d, 1H, *J* = 8Hz), 7.65 (d, 2H, *J* = 8 Hz), 7.69 (d, 2H, *J* = 10 Hz), 7.95 (d, 2H, *J* = 8 Hz).

^{13}C{^{1}H} NMR (100 MHz, $CDCl_3$, 298K) δ (ppm) 101.4, 111.1, 120.9, 122.9, 124.3, 125.3, 126.9, 127.4, 127.6, 128.8, 129.2, 129.4, 140.4, 141.2, 154.9, 155.7.

2-(4-Methoxyphenyl)thiazole (11b):[24]

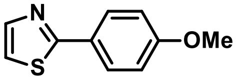

11b

The crude product was purified by column chromatography using silica gel (100-200 mesh) with 10% ethylacetate in hexane. The compound was obtained as a light yellow colored oil.

^{1}H NMR (400 MHz, $CDCl_3$, 298K) δ (ppm) 3.86 (s, 3H), 6.96 (dd, 2H, J_1 = 7 Hz, J_2 = 4 Hz), 7.25 (m, 1H), 7.81 (s, 1H), 7.91 (dd, 2H, J_1 = 9 Hz, J_2 = 3 Hz)

^{13}C{^{1}H} NMR (100 MHz, $CDCl_3$, 298K) δ (ppm) 55.4, 114.3, 117.8, 126.6, 128.0, 143.4, 161.1, 168.3.

2-Phenylthiazole (11c):[24]

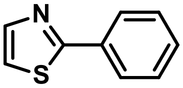

11c

The crude product was purified by column chromatography using silica gel (100-200 mesh) with 10% ethylacetate in hexane. The compound was obtained as a colorless oil.

^{1}H NMR (400 MHz, $CDCl_3$, 298K) δ (ppm) 7.34 (d, 1H, J = 4 Hz), 7.43-7.76 (m, 4H), 7.87 (d, 1H, J = 4 Hz), 7.97 (d, 2H, J = 8 Hz).

^{13}C{^{1}H} NMR (100 MHz, $CDCl_3$, 298K) δ (ppm) 118.9, 126.6, 128.9, 130.1, 133.7, 143.7, 168.5.

2-**(3, 5-Dimethylphenyl)thiazole (11d):**[24]

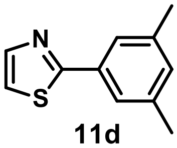

11d

The crude product was purified by column chromatography using silica gel (100-200 mesh) with 10% ethylacetate in hexane. The compound was obtained as a colorless oil.

^{1}H NMR (400 MHz, $CDCl_3$, 298K) δ (ppm) 2.4 (s, 6H), 7.07 (s, 1H), 7.30 (d, 1H, J = 4 Hz), 7.59 (s, 2H), 7.84 (d, 1H, J = 4 Hz).

^{13}C{^{1}H} NMR (100 MHz, $CDCl_3$, 298K) δ (ppm) 21.2, 118.5, 124.4, 131.7, 133.4, 138.6, 143.5, 168.8.

2-Biphenylthiazole (11e):[25]

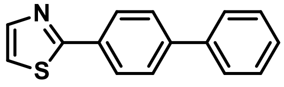

11e

The crude product was purified by column chromatography using silica gel (100-200 mesh) with 10% ethylacetate in hexane. The compound was obtained as a brown colored oil.

^{1}H NMR (400 MHz, $CDCl_3$, 298K) δ (ppm) 7.34 (d, 1H, J = 4 Hz), 7.40 (d, 1H, J = 4 Hz), 7.47 (t, 2H, J = 8 Hz), 7.65 (d, 2H, J = 8 Hz), 7.69 (d, 2H, J = 9 Hz), 7.90 (d, 1H, J = 4 Hz), 8.05 (d, 2H, J = 9 Hz).

^{13}C{^{1}H} NMR (100 MHz, $CDCl_3$, 298K) δ(ppm) 118.8, 126.9, 127.0, 127.6, 127.8, 128.9, 132.5, 140.2, 142.7, 143.7, 168.1.

2-**(4-Methylphenyl)thiazole (11f):**[24]

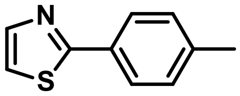

11f

The crude product was purified by column chromatography using silica gel (100-200 mesh) with 10% ethylacetate in hexane. The compound was obtained as a colorless oil.

^{1}H NMR (400 MHz, $CDCl_3$, 298K) δ (ppm) 2.4 (s, 3H), 7.23 (m, 2H), 7.27 (m, 1H), 7.82-7.85 (m, 3H).

^{13}C{^{1}H} NMR (100 MHz, $CDCl_3$, 298K) δ(ppm) 21.4, 118.3, 126.5, 129.5, 129.6, 130.9, 140.2, 143.5, 168.6.

22. ^{1}H and ^{13}C NMR spectra of the catalytic products.

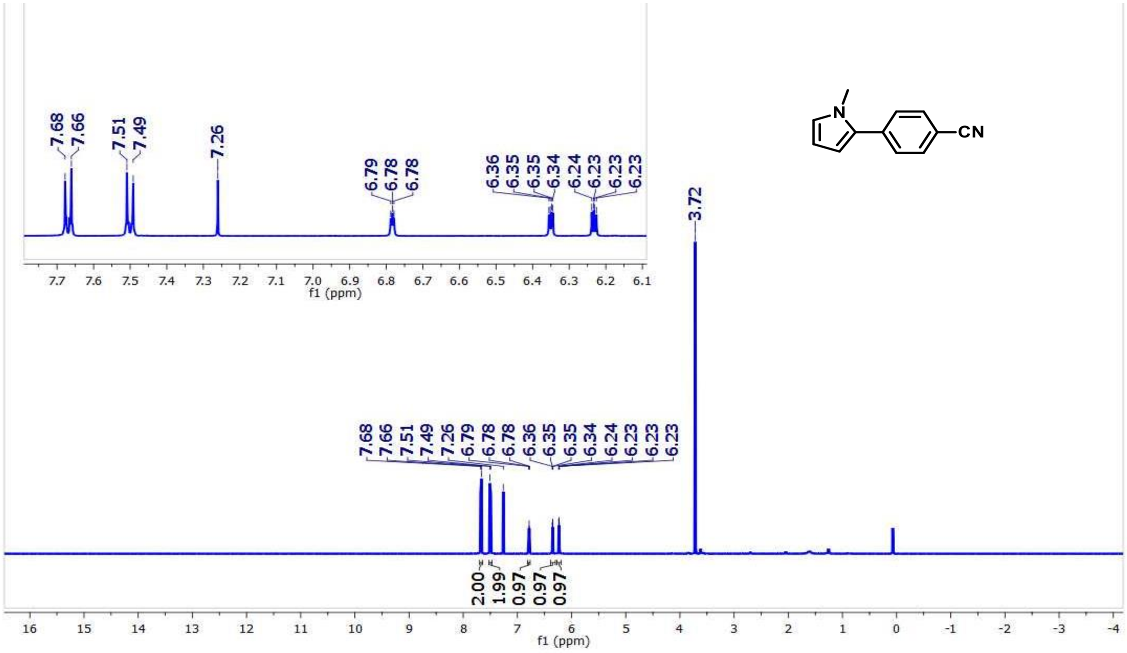

Figure S28. ^{1}H NMR ($CDCl_3$) spectrum of 4-(1-methylpyrrole)benzonitrile (**4a**).

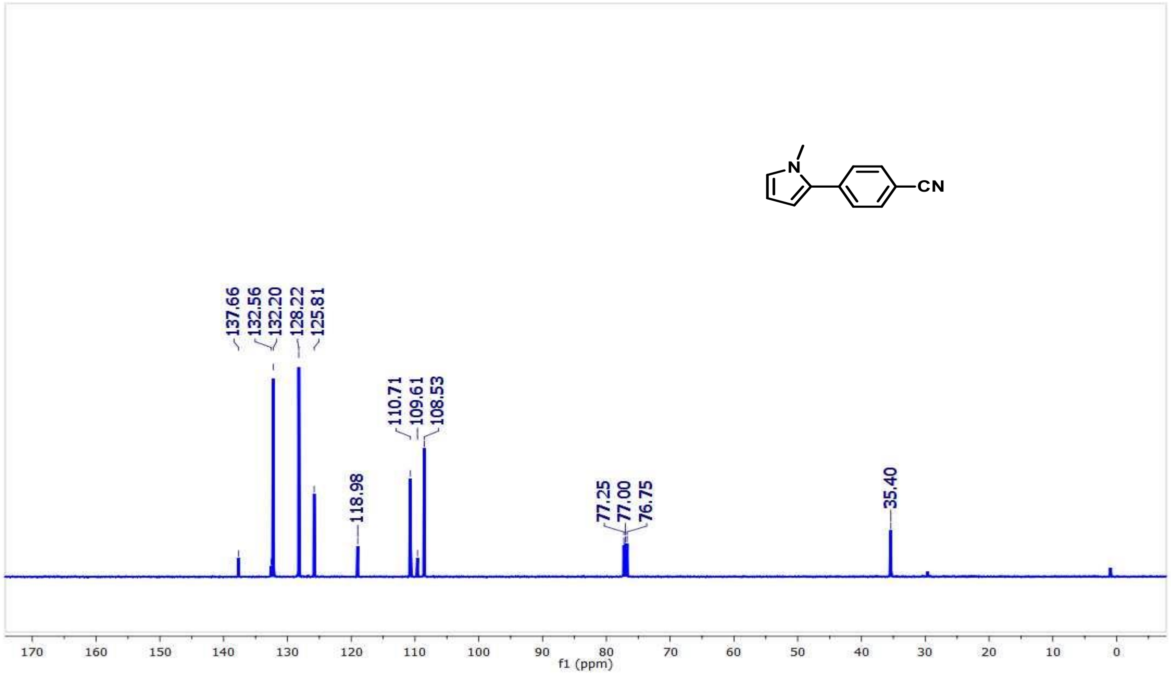

Figure S29. ^{13}C NMR ($CDCl_3$) spectrum of 4-(1-methylpyrrole)benzonitrile (**4a**).

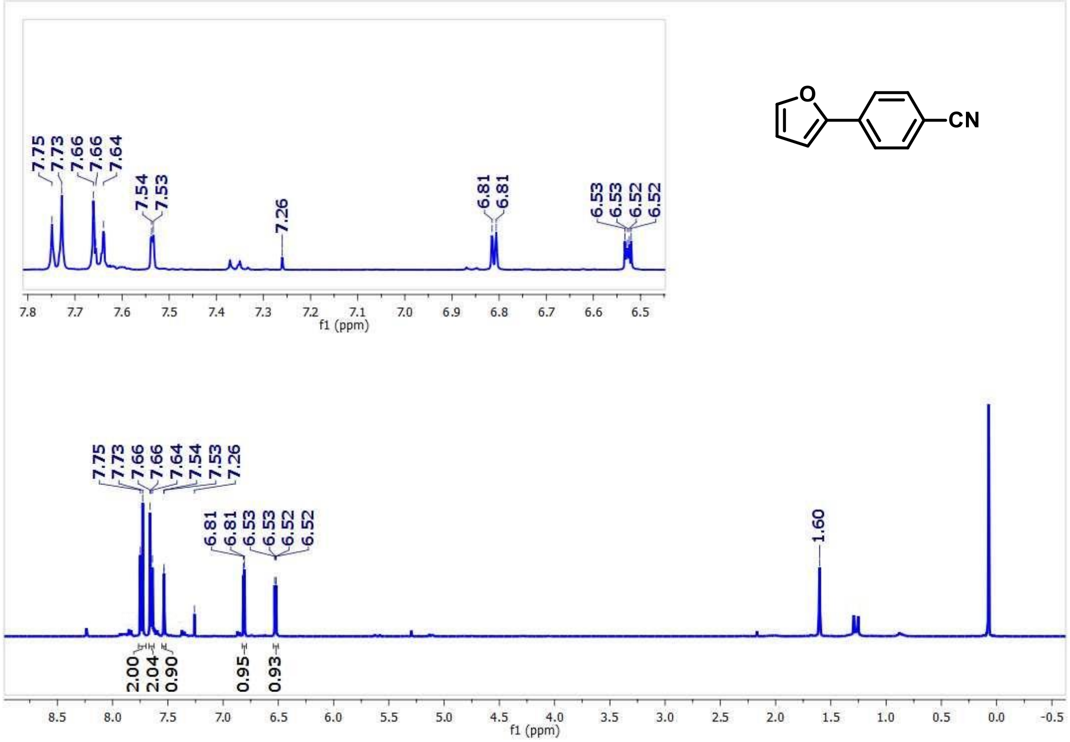

Figure S30. ^{1}H NMR ($CDCl_3$) spectrum of 2-(4-cyanophenyl)furan (**5a**).

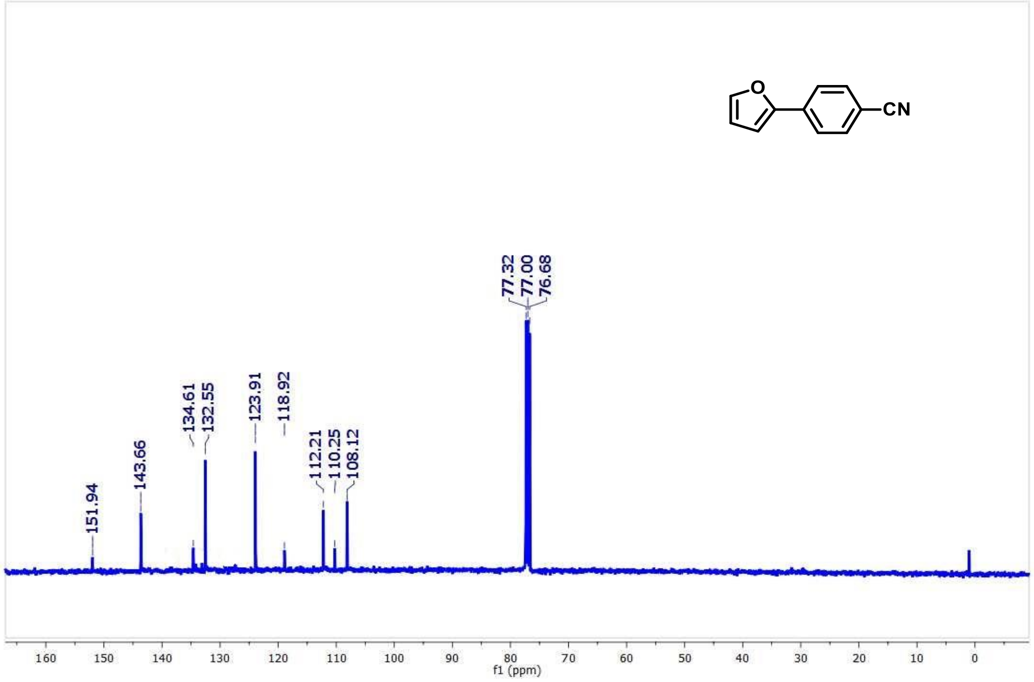

Figure S31. ^{13}C NMR ($CDCl_3$) spectrum of 2-(4-cyanophenyl)furan (**5a**).

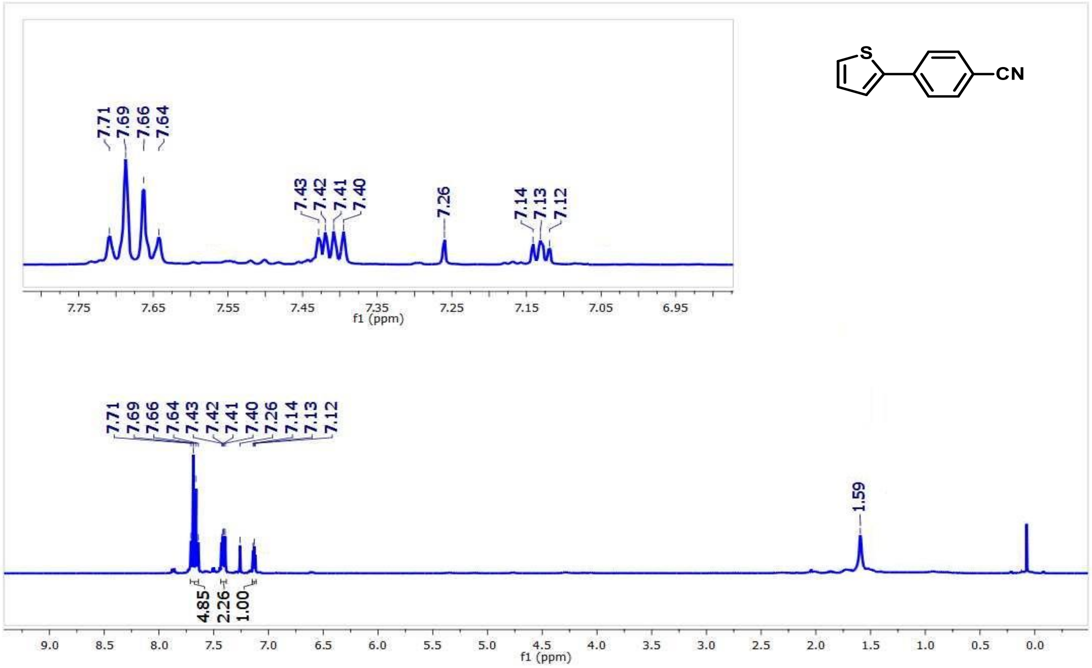

Figure S32. ^{1}H NMR ($CDCl_3$) spectrum of 2-(4-cyanophenyl)thiophene (**6a**).

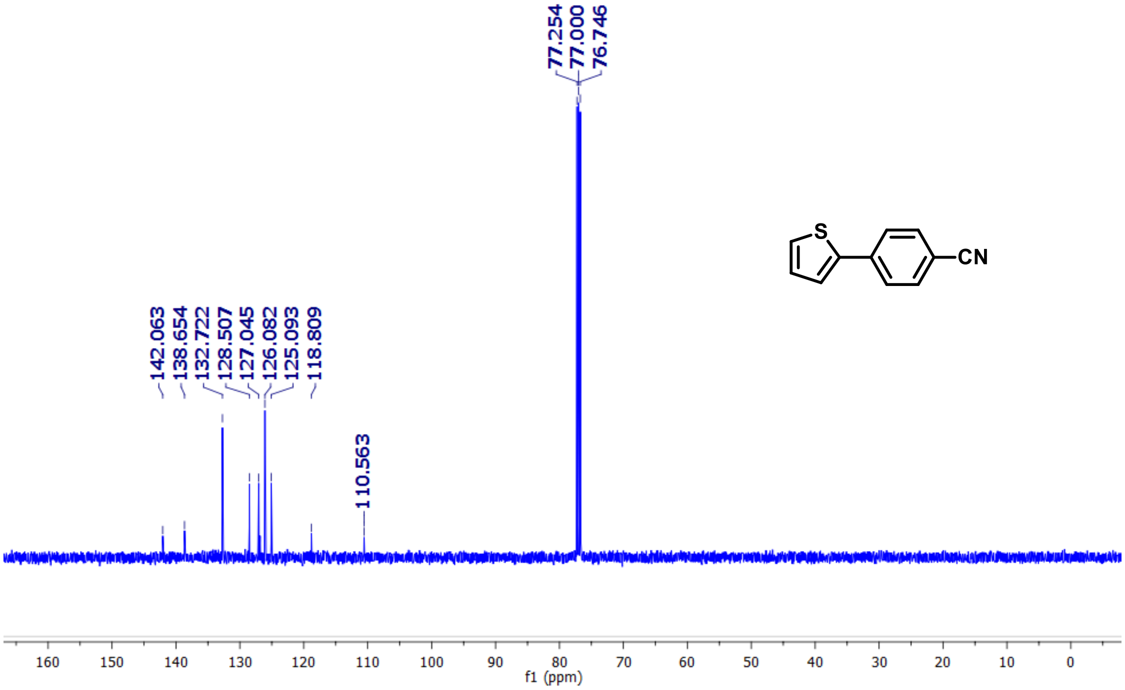

Figure S33. ^{13}C NMR ($CDCl_3$) spectrum of 2-(4-cyanophenyl)thiophene (**6a**).

.

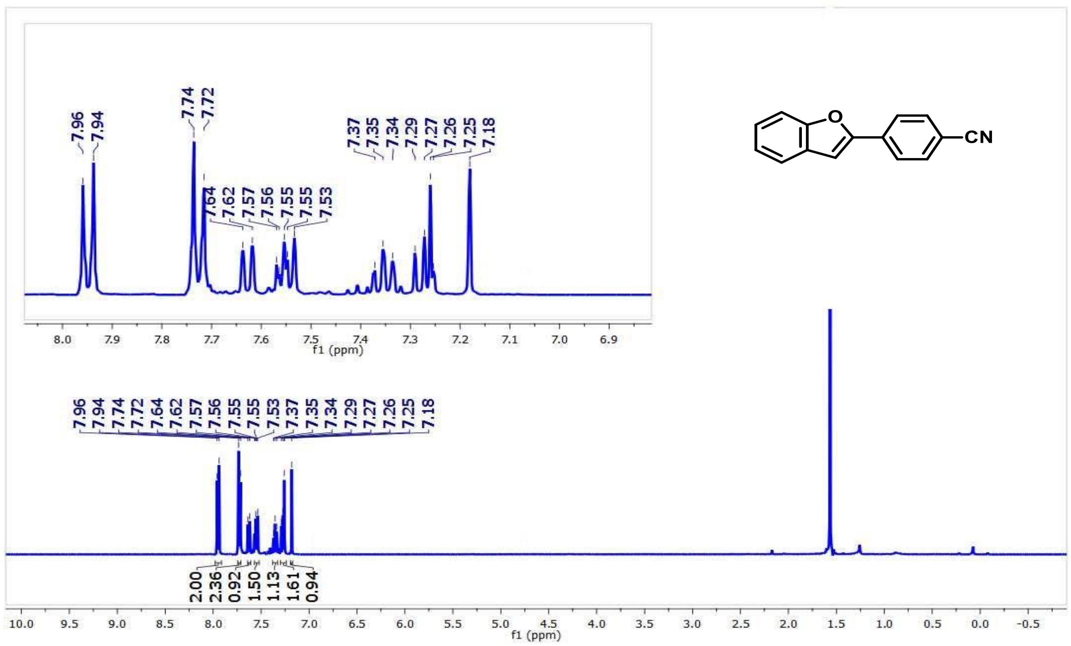

Figure S34. ^{1}H NMR ($CDCl_3$) spectrum of 4-(benzofuran-2-yl)benzonitrile (**7a**).

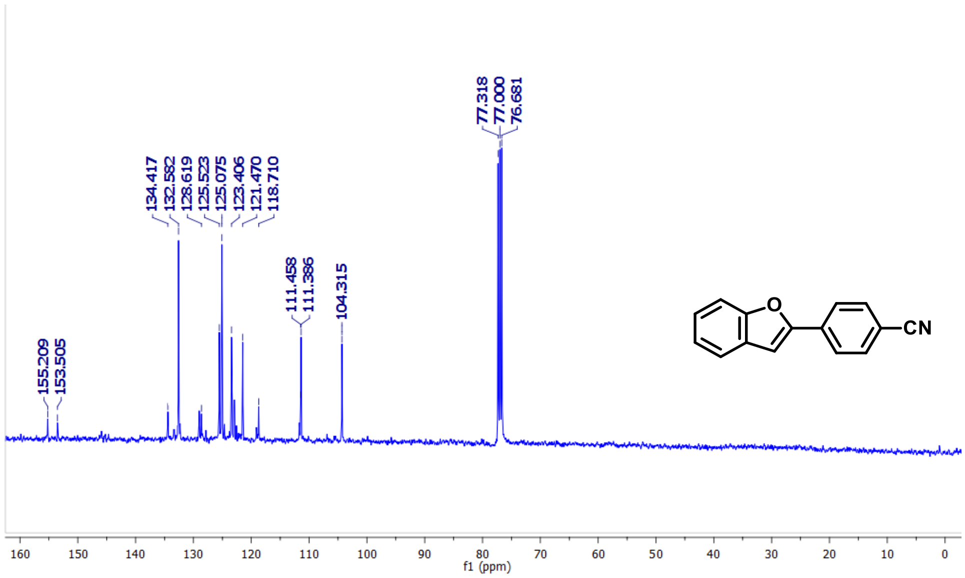

Figure S35. ^{13}C NMR ($CDCl_3$) spectrum of 4-(benzofuran-2-yl)benzonitrile (**7a**).

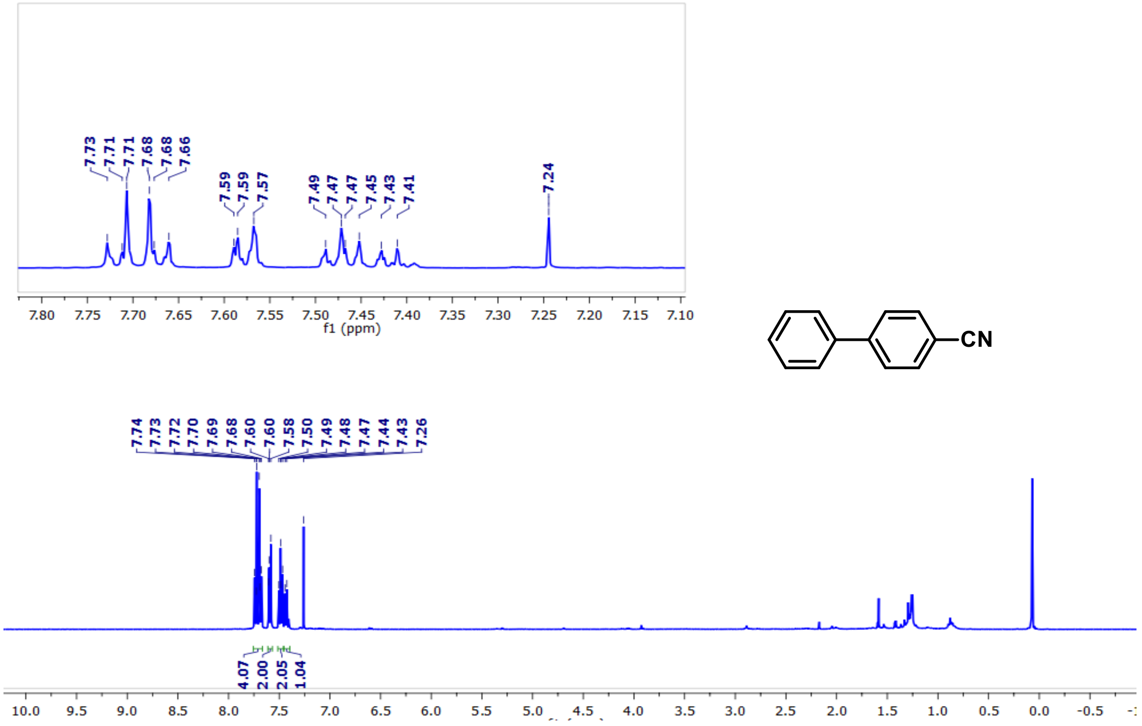

Figure S36. ^{1}H NMR ($CDCl_3$) spectrum of 4-cyanobiphenyl (**8a**).

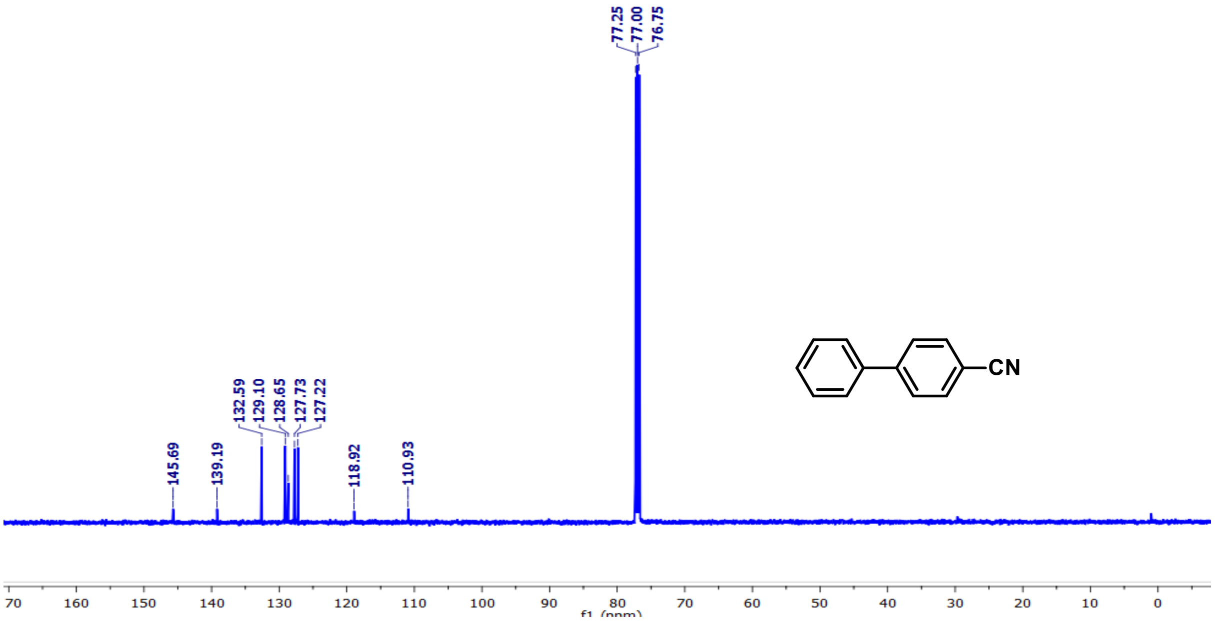

Figure S37. ^{13}C NMR ($CDCl_3$) spectrum of 4-cyanobiphenyl (**8a**).

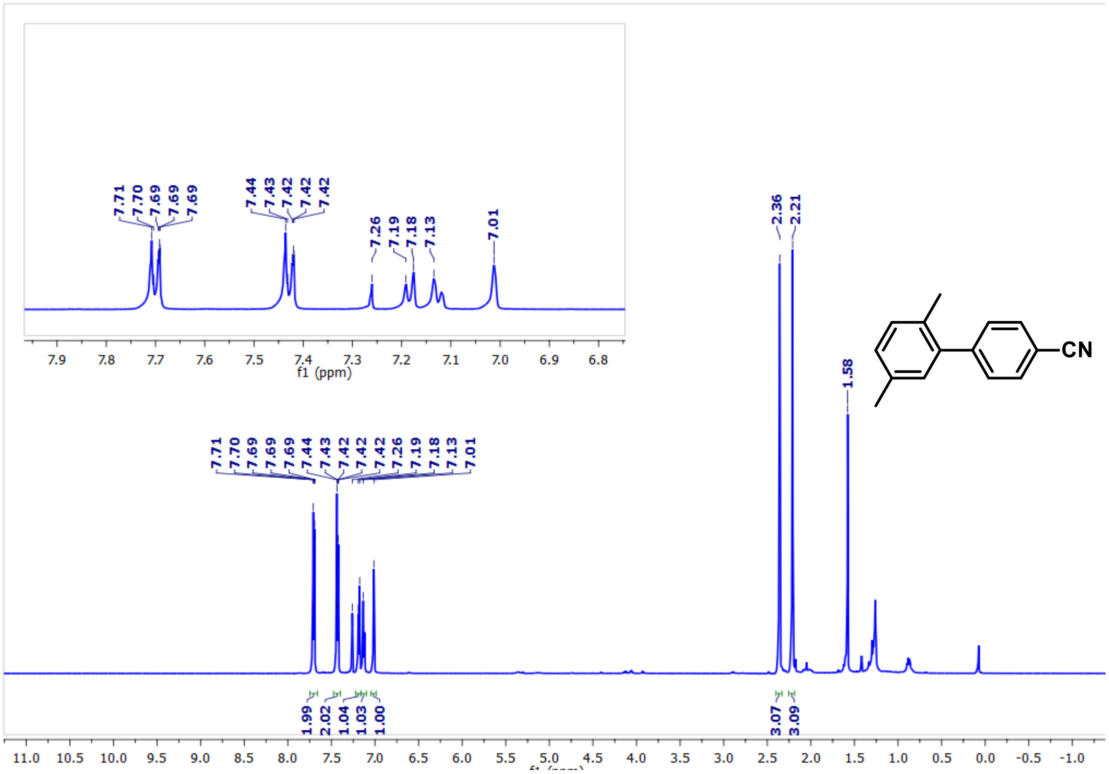

Figure S38. ^{1}H NMR ($CDCl_3$) spectrum of 1-cyano-4-(2, 5-dimethylbenzene) (**9a**).

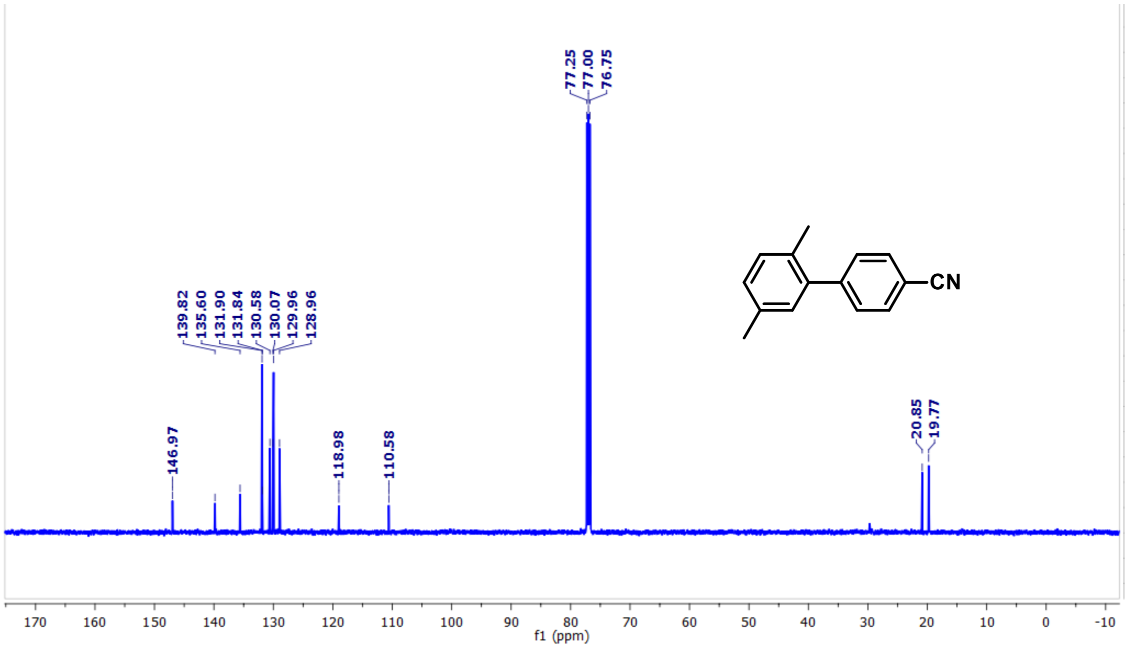

Figure S39. ^{13}C NMR ($CDCl_3$) spectrum of 1-cyano-4-(2, 5-dimethylbenzene) (**9a**).

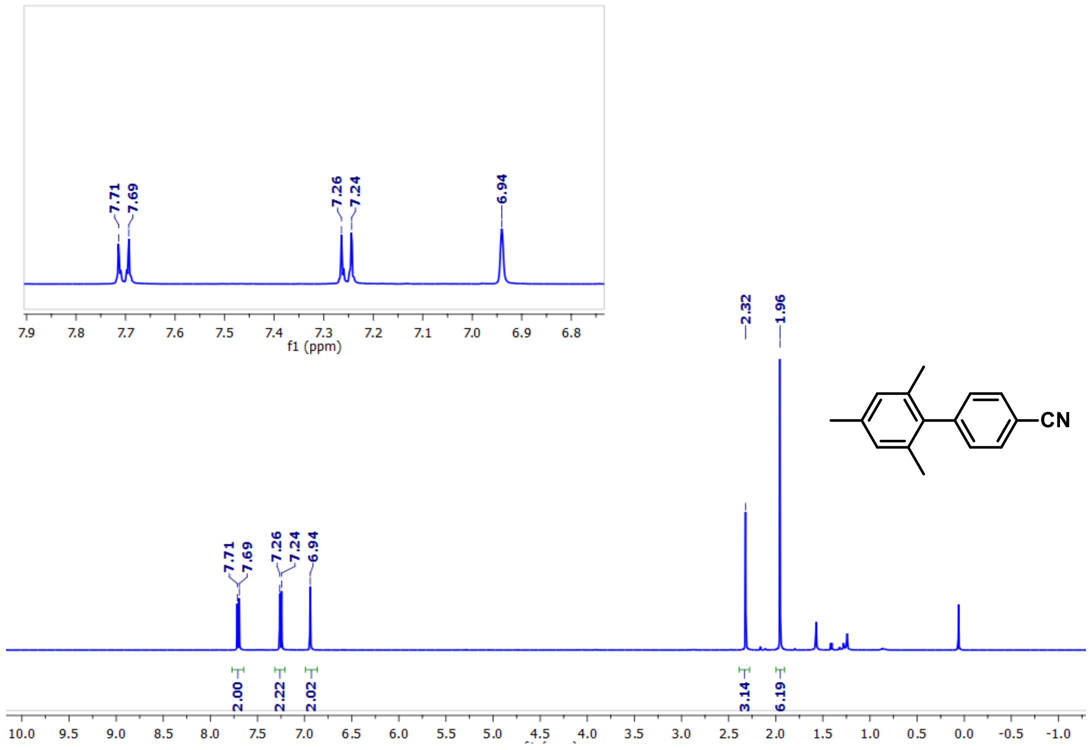

Figure S40. ^{1}H NMR ($CDCl_3$) spectrum of 1-cyano-4-mesitylbenzene (**10a**).

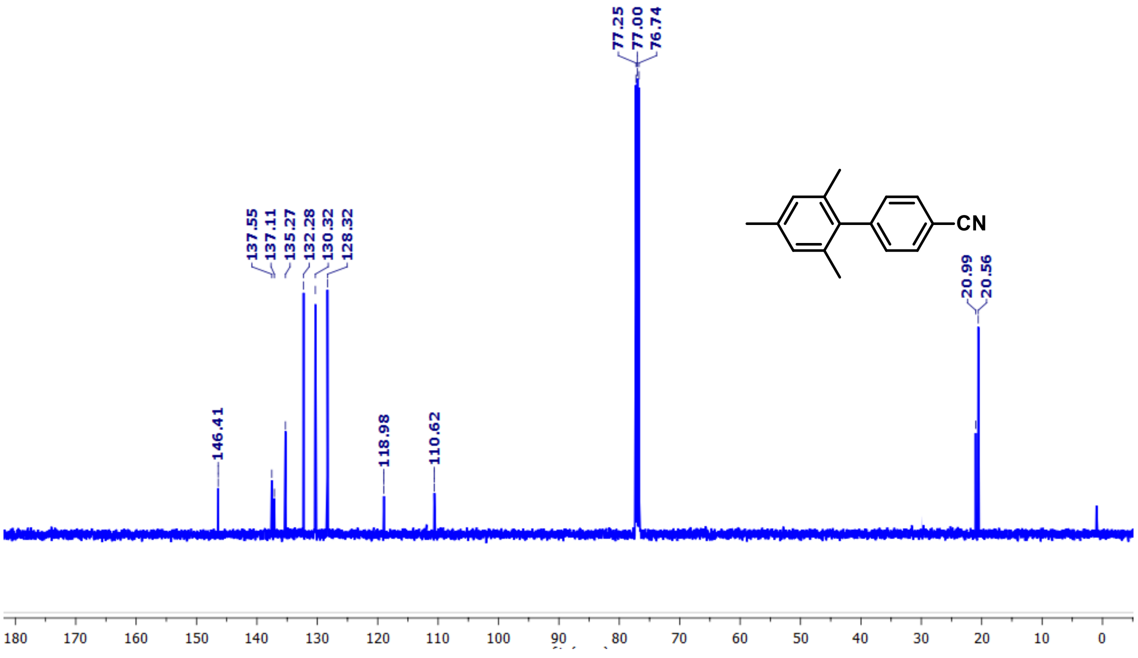

Figure S41. ^{13}C NMR ($CDCl_3$) spectrum of 1-cyano-4-mesitylbenzene (**10a**).

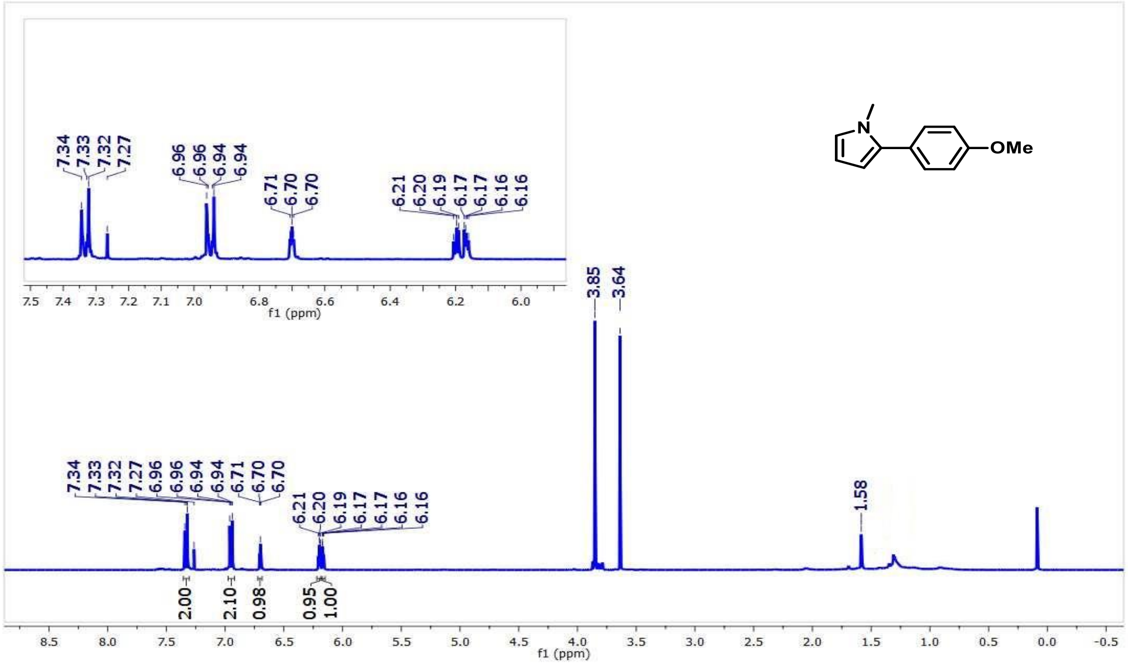

Figure S42. ^{1}H NMR ($CDCl_3$) spectrum of 4-(1-methylpyrrole)anisole (**4b**).

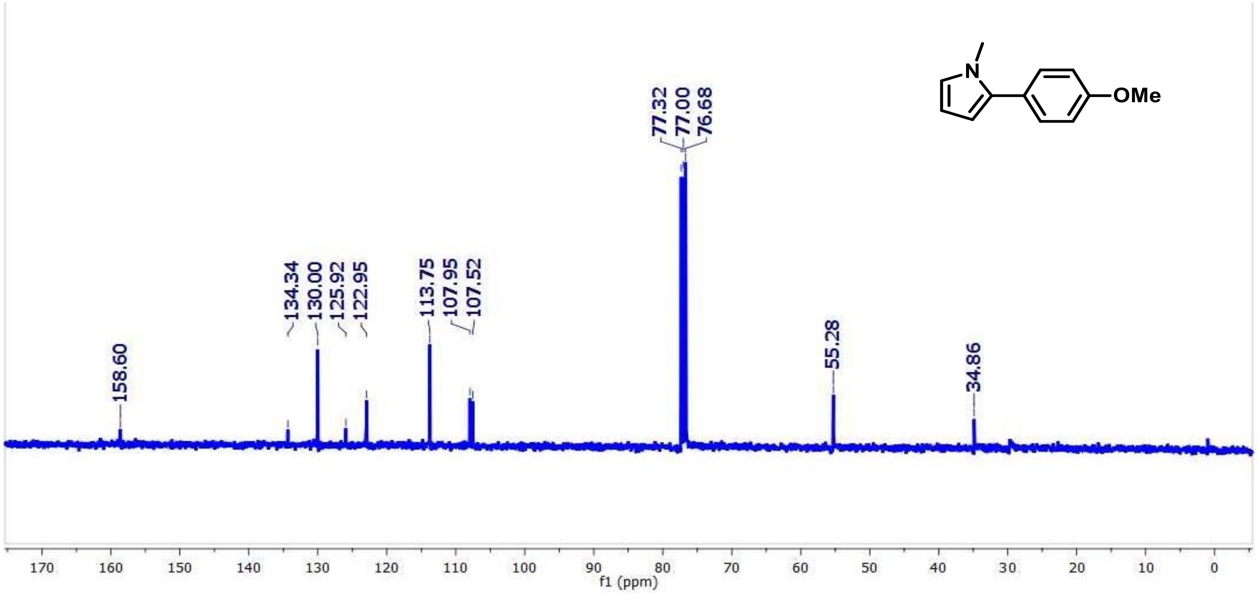

Figure S43. ^{13}C NMR ($CDCl_3$) spectrum of 4-(1-methylpyrrole)anisole (**4b**).

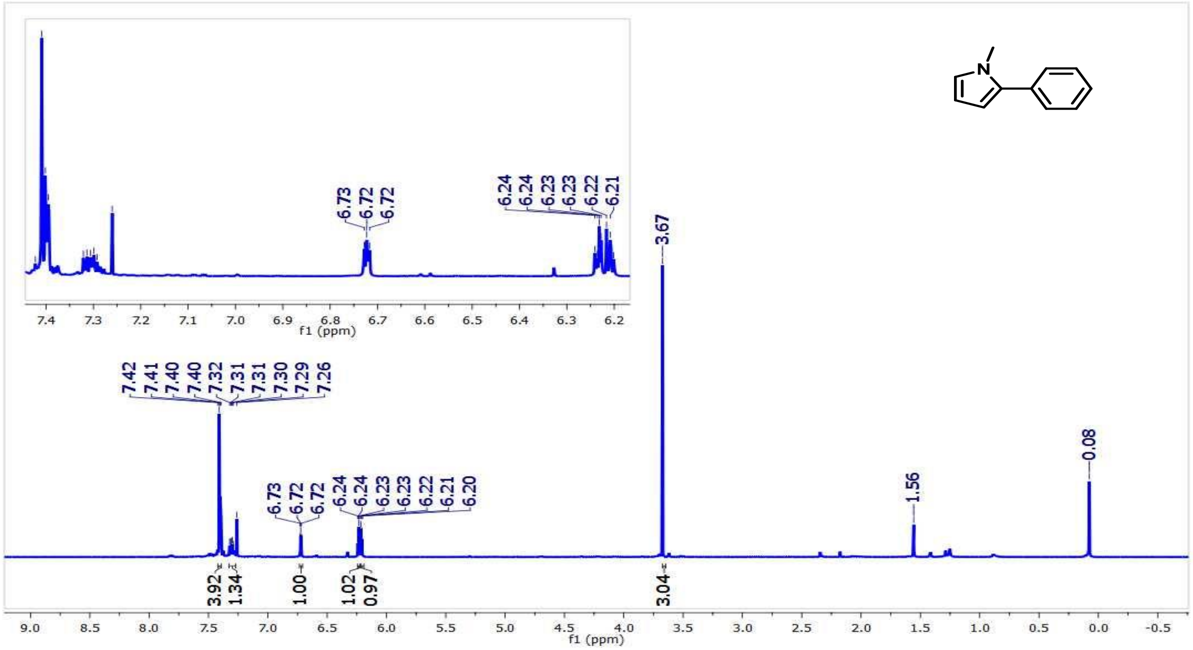

Figure S44. ^{1}H NMR ($CDCl_3$) spectrum of 4-(1-methylpyrrole)benzene (**4c**).

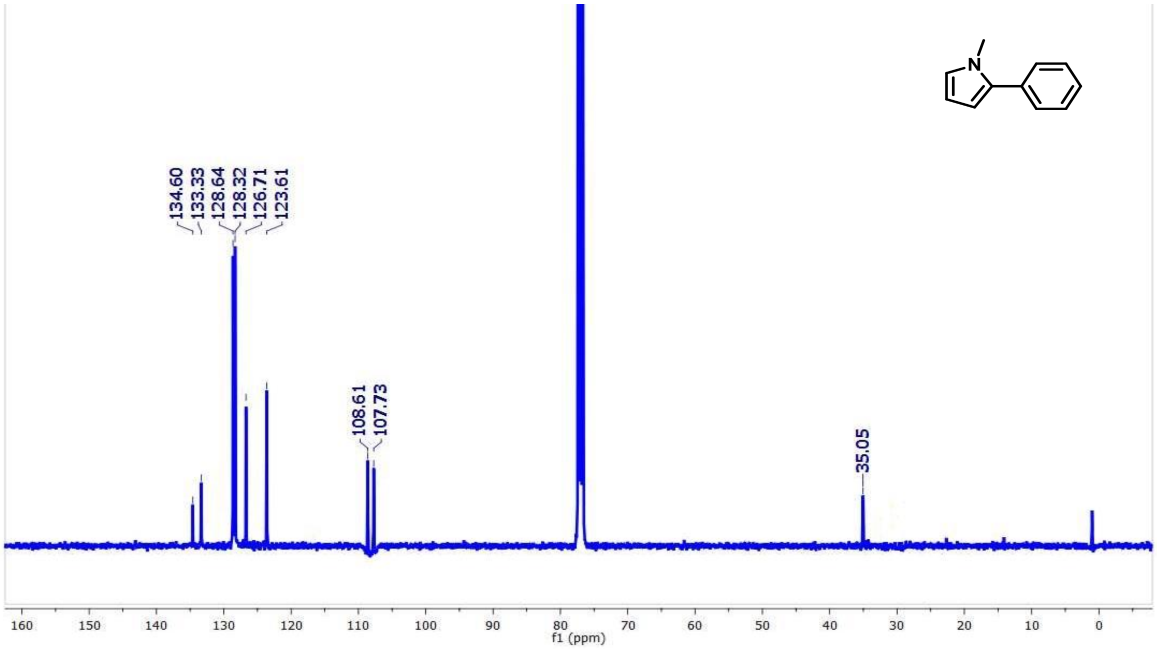

Figure S45. ^{13}C NMR ($CDCl_3$) spectrum of 4-(1-methylpyrrole)benzene (**4c**).

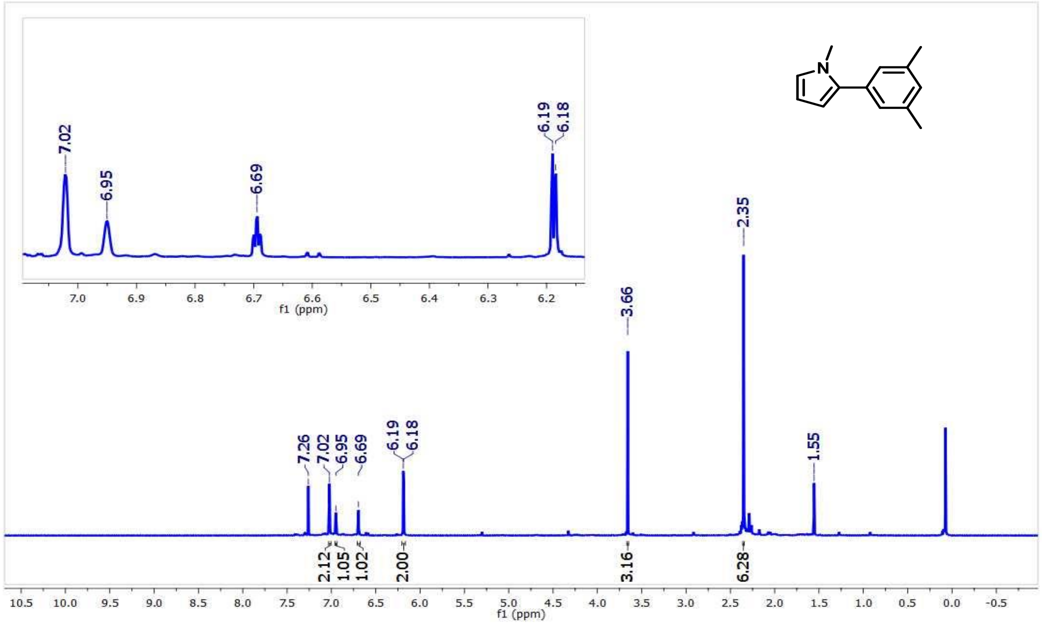

Figure S46. ^{1}H NMR ($CDCl_3$) spectrum of 4-(1-methylpyrrole)3, 5-dimethylbenzene (**4d**).

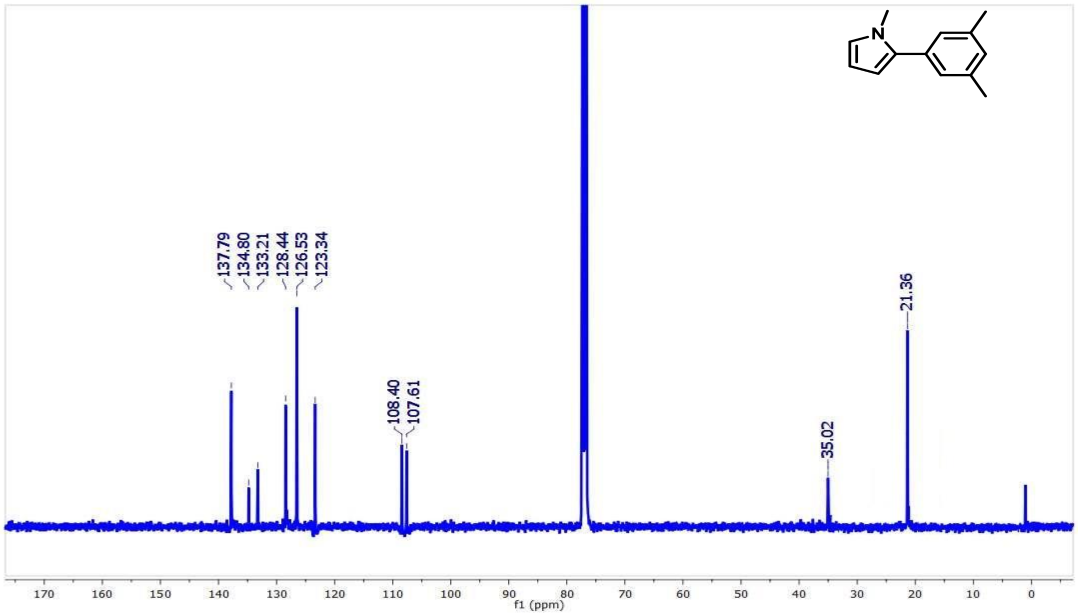

Figure S47. ^{13}C NMR ($CDCl_3$) spectrum of 4-(1-methylpyrrole)3, 5-dimethylbenzene (**4d**).

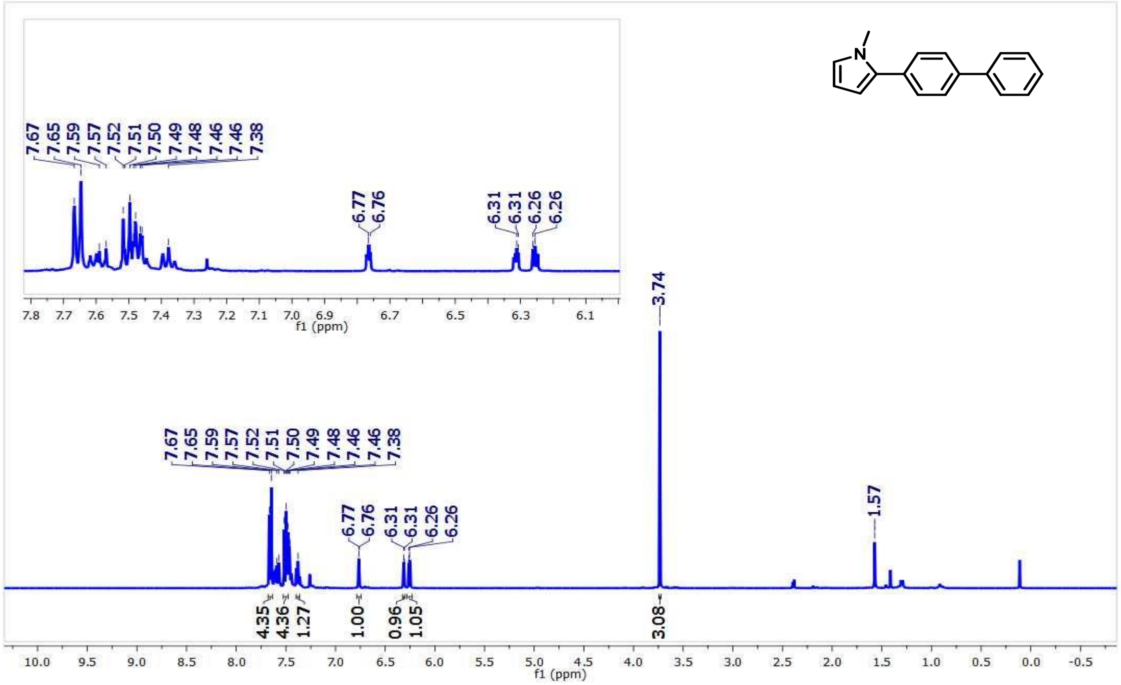

Figure S48. ^{1}H NMR ($CDCl_3$) spectrum of 4-(1-methylpyrrole)biphenyl (**4e**).

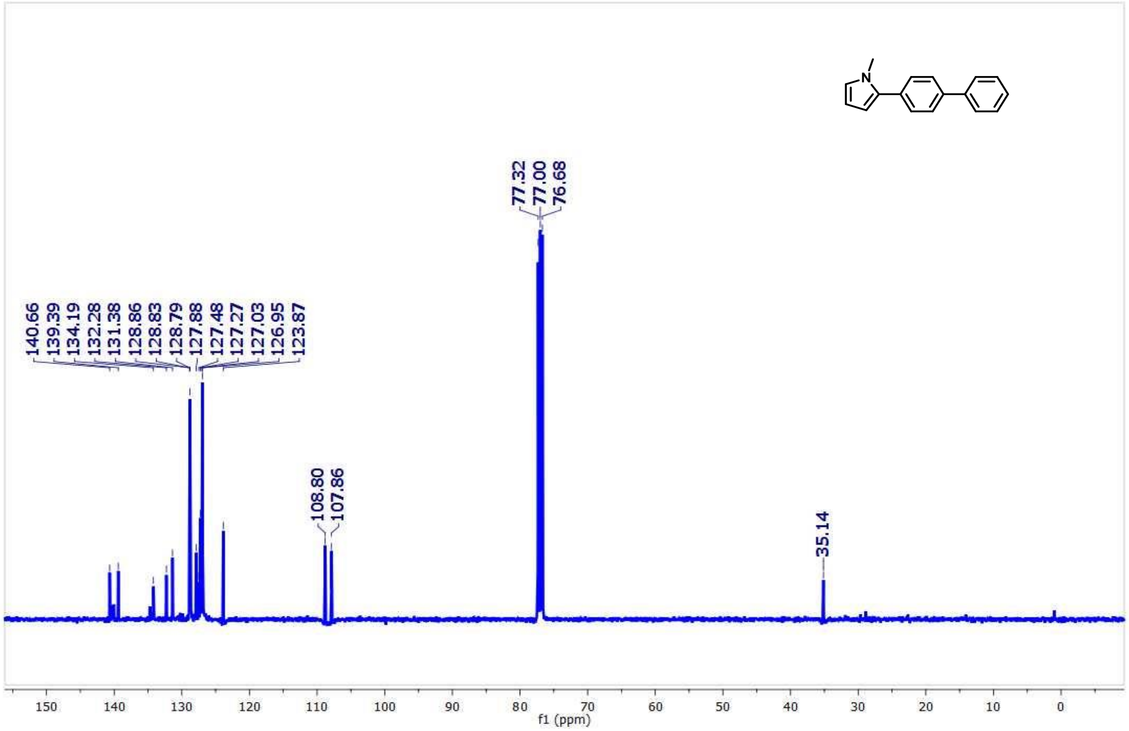

Figure S49. ^{13}C NMR ($CDCl_3$) spectrum of 4-(1-methylpyrrole)biphenyl (**4e**).

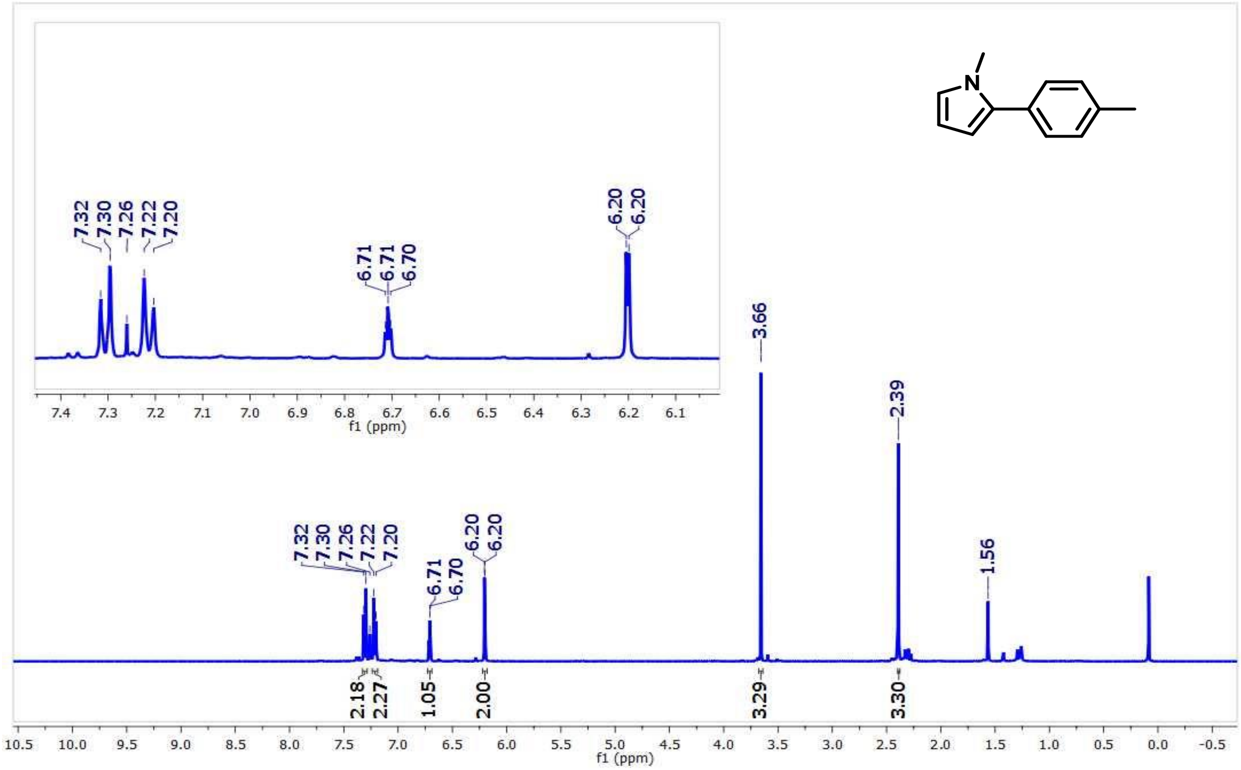

Figure S50. ^{1}H NMR ($CDCl_3$) spectrum of 4-(1-methylpyrrole)toluene (**4f**).

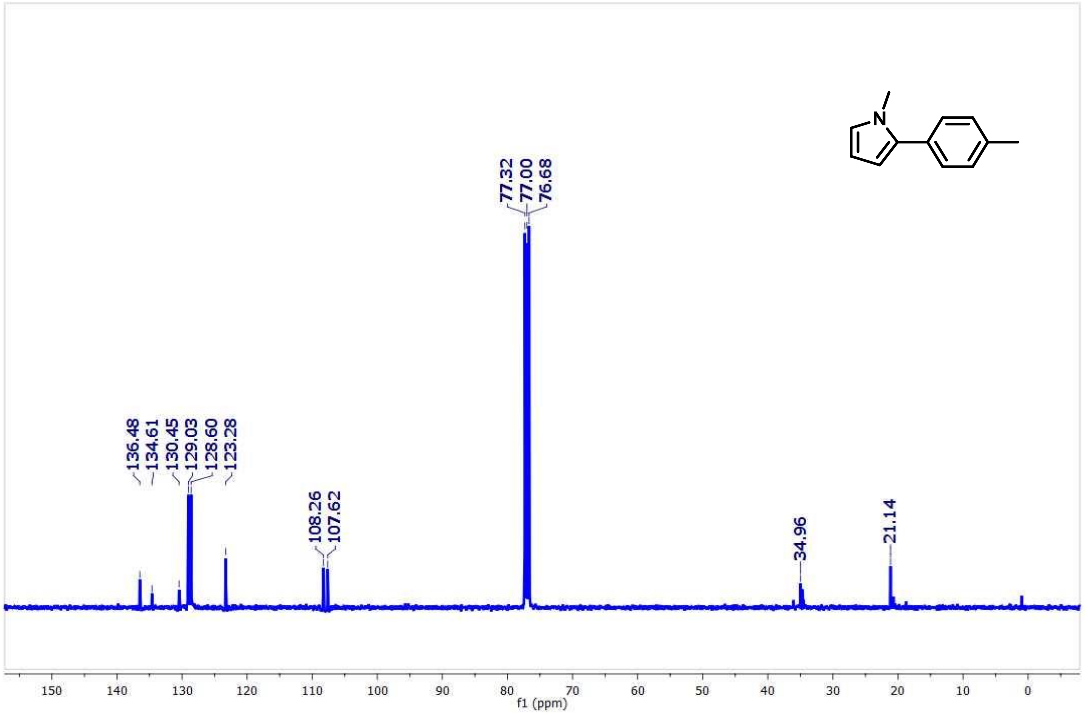

Figure S51. ^{13}C NMR ($CDCl_3$) spectrum of 4-(1-methylpyrrole)toluene (**4f**).

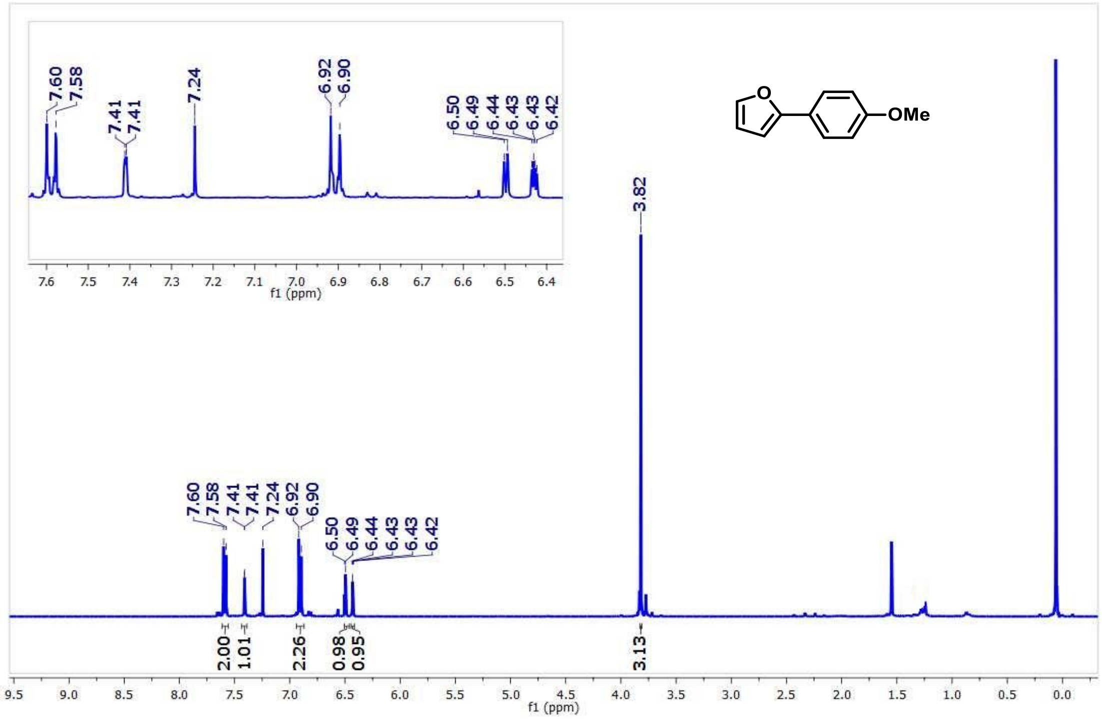

Figure S52. ^{1}H NMR ($CDCl_3$) spectrum of 2-(4-methoxyphenyl)furan (**5b**).

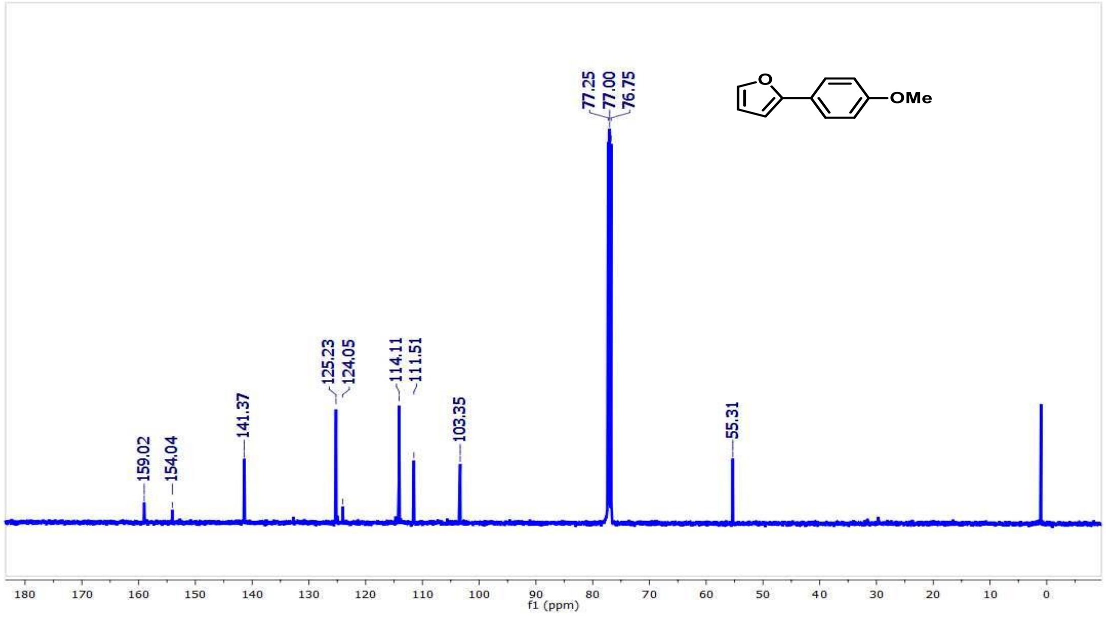

Figure S53. ^{13}C NMR ($CDCl_3$) spectrum of 2-(4-methoxyphenyl)furan (**5b**).

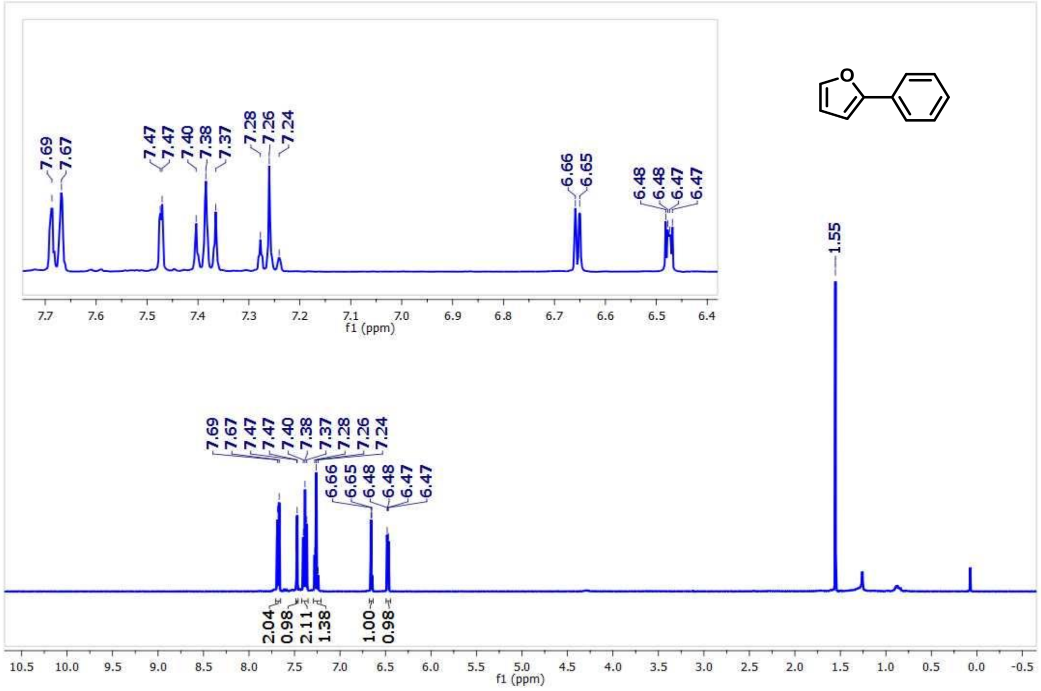

Figure S54. ^{1}H NMR ($CDCl_3$) spectrum of 2-phenylfuran (**5c**).

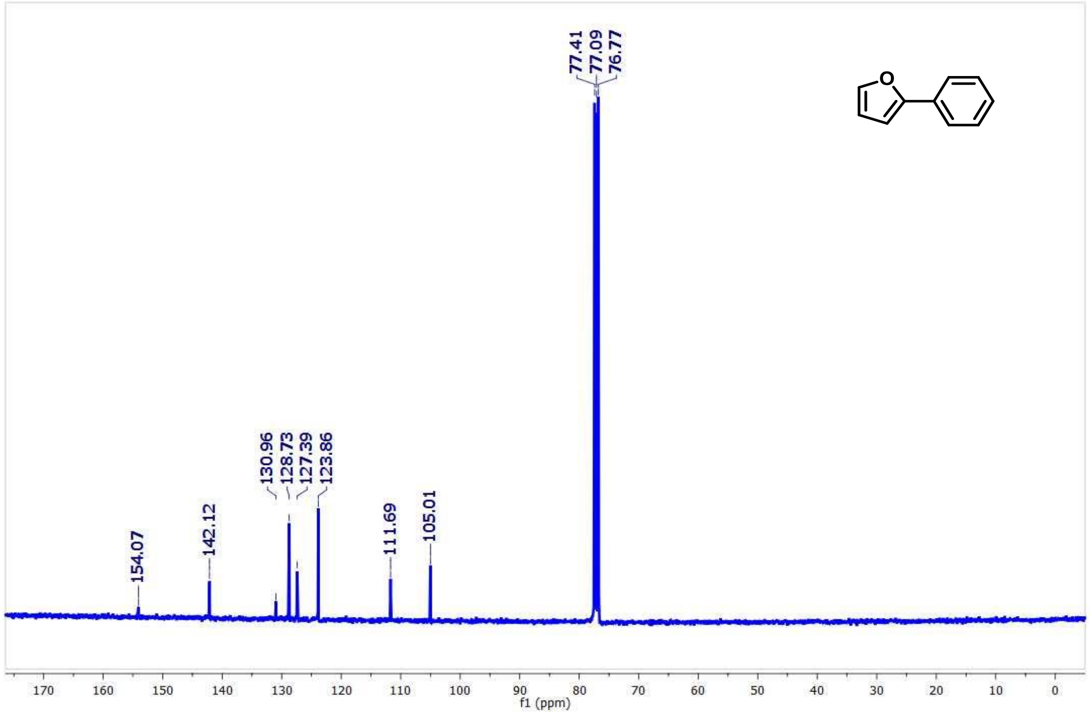

Figure S55. ^{13}C NMR ($CDCl_3$) spectrum of 2-phenylfuran (**5c**).

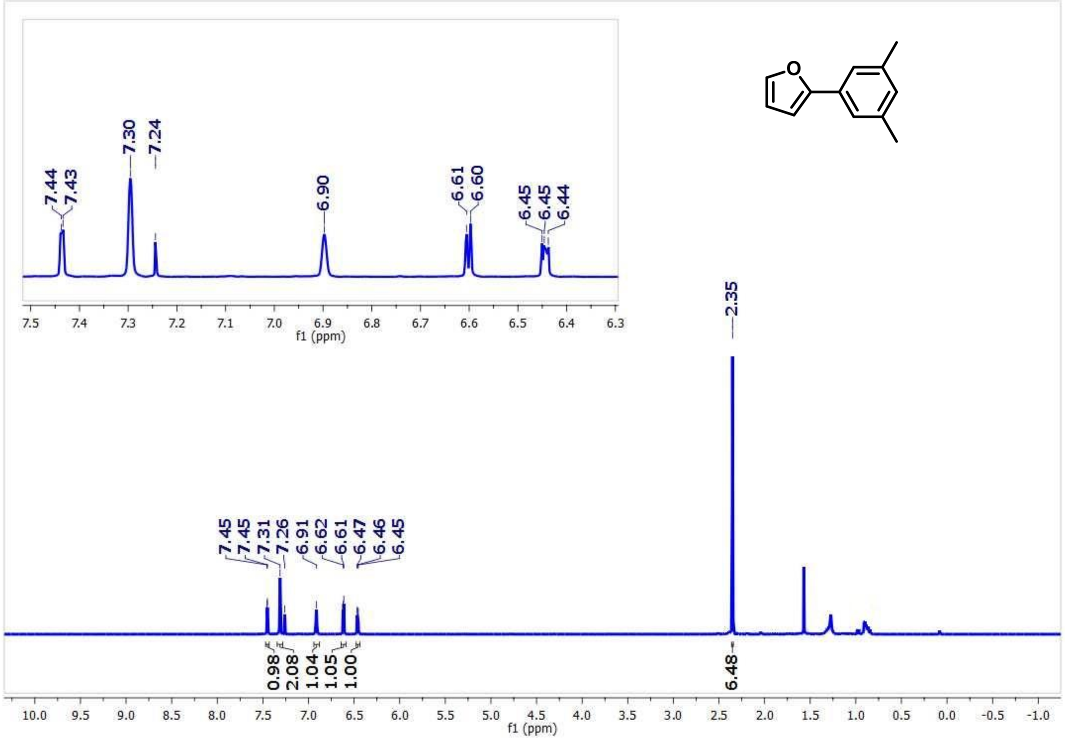

Figure S56. ^{1}H NMR ($CDCl_3$) spectrum of 2-(3, 5-dimethylphenyl)furan (**5d**).

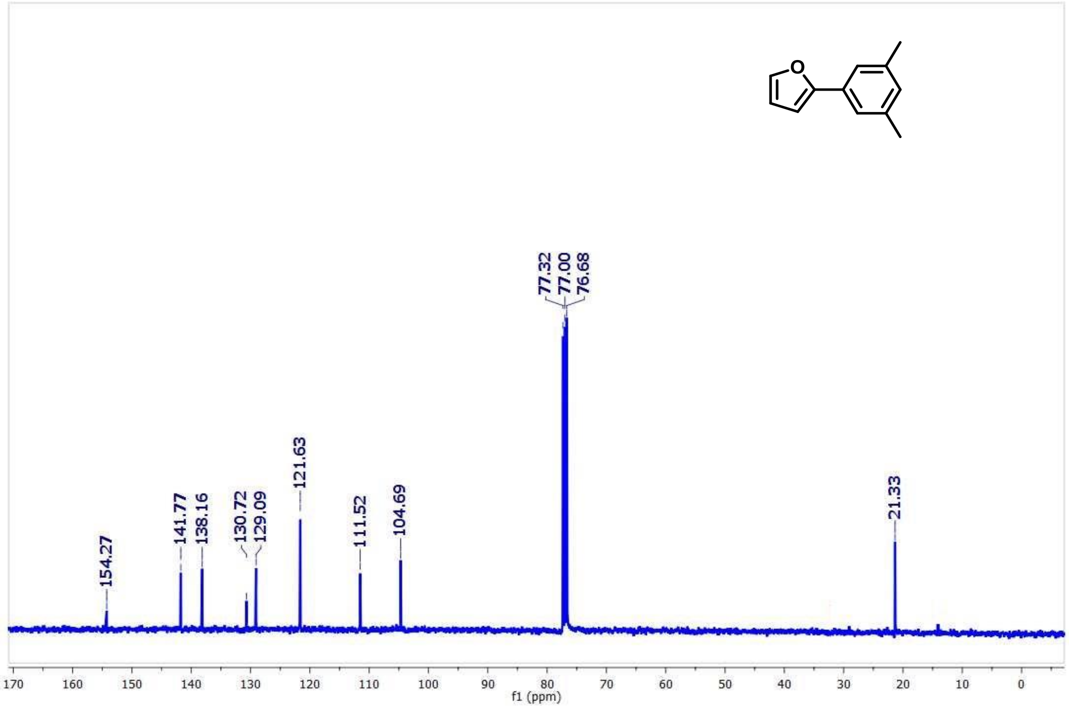

Figure S57. ^{13}C NMR ($CDCl_3$) spectrum of 2-(3, 5-dimethylphenyl)furan (**5d**).

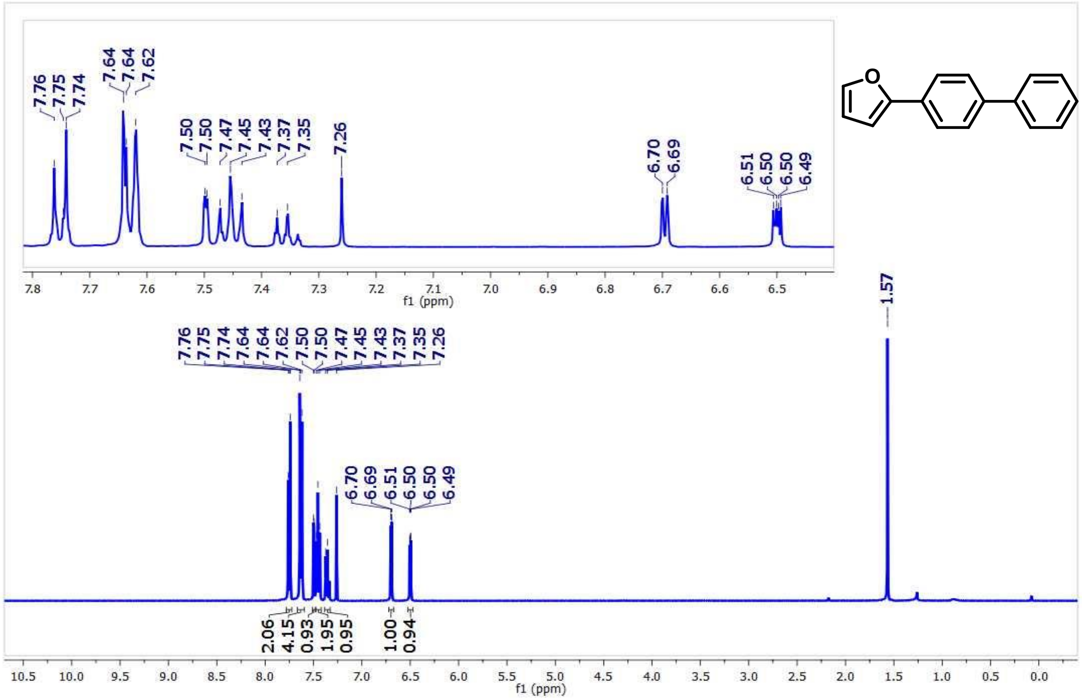

Figure S58. ^{1}H NMR ($CDCl_3$) spectrum of 2-biphenylfuran (**5e**).

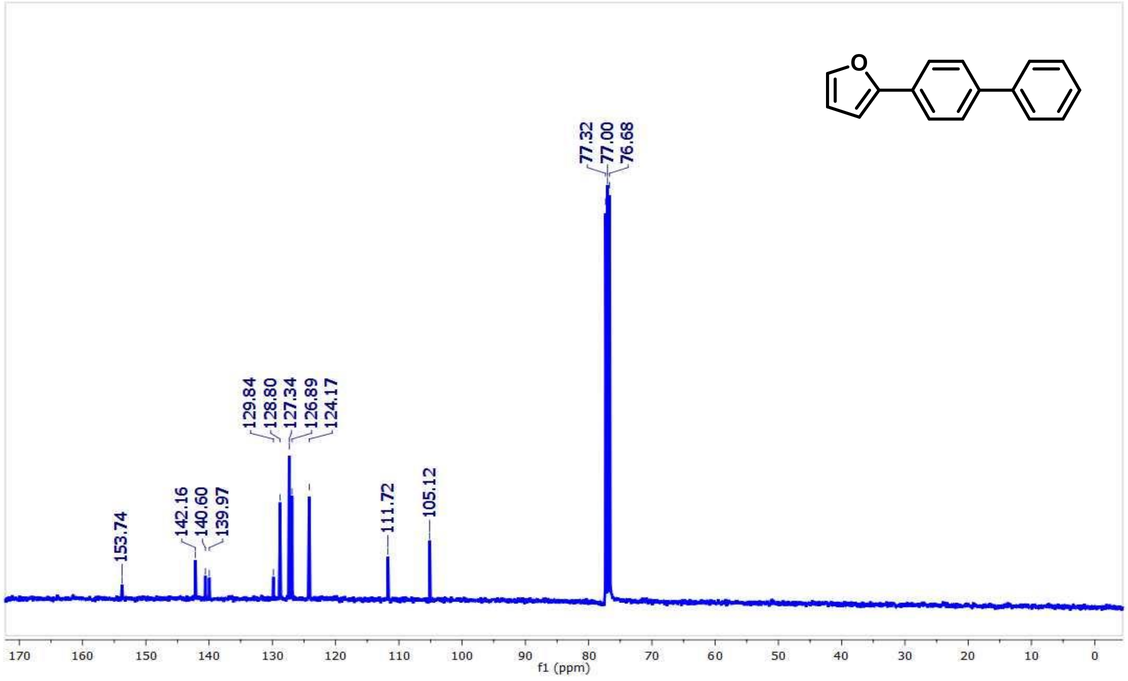

Figure S59. ^{13}C NMR ($CDCl_3$) spectrum of 2-biphenylfuran (**5e**).

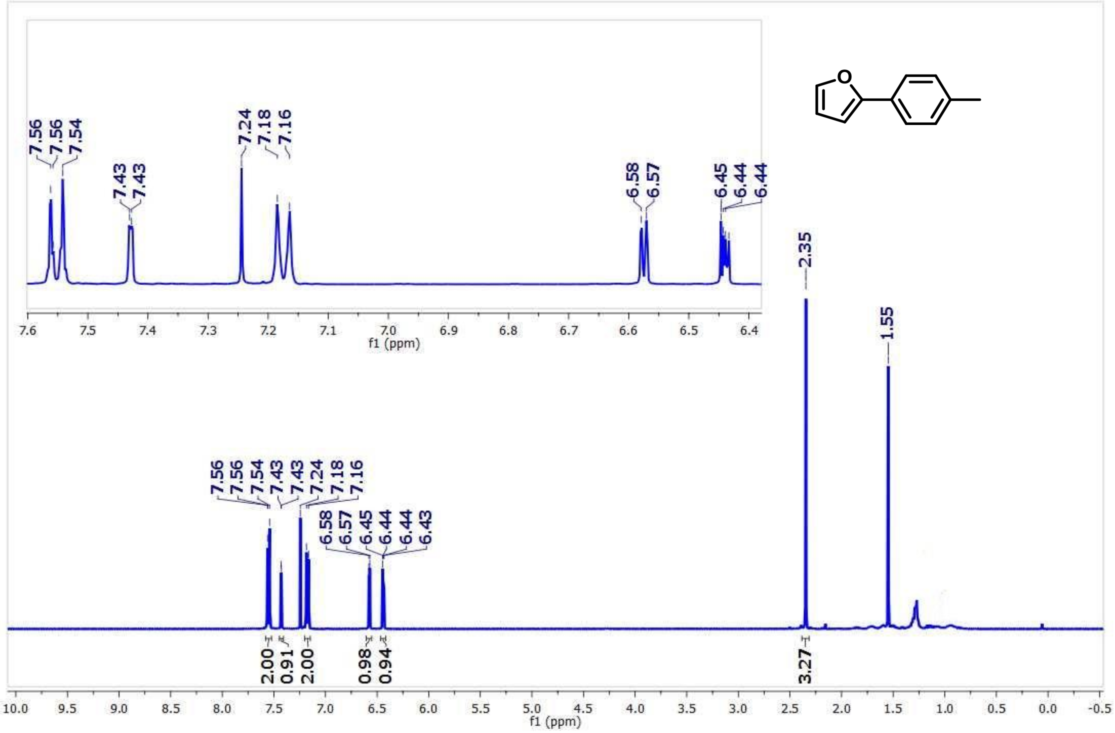

Figure S60. ^{1}H NMR ($CDCl_3$) spectrum of 2-(4-methylphenyl)furan (**5f**).

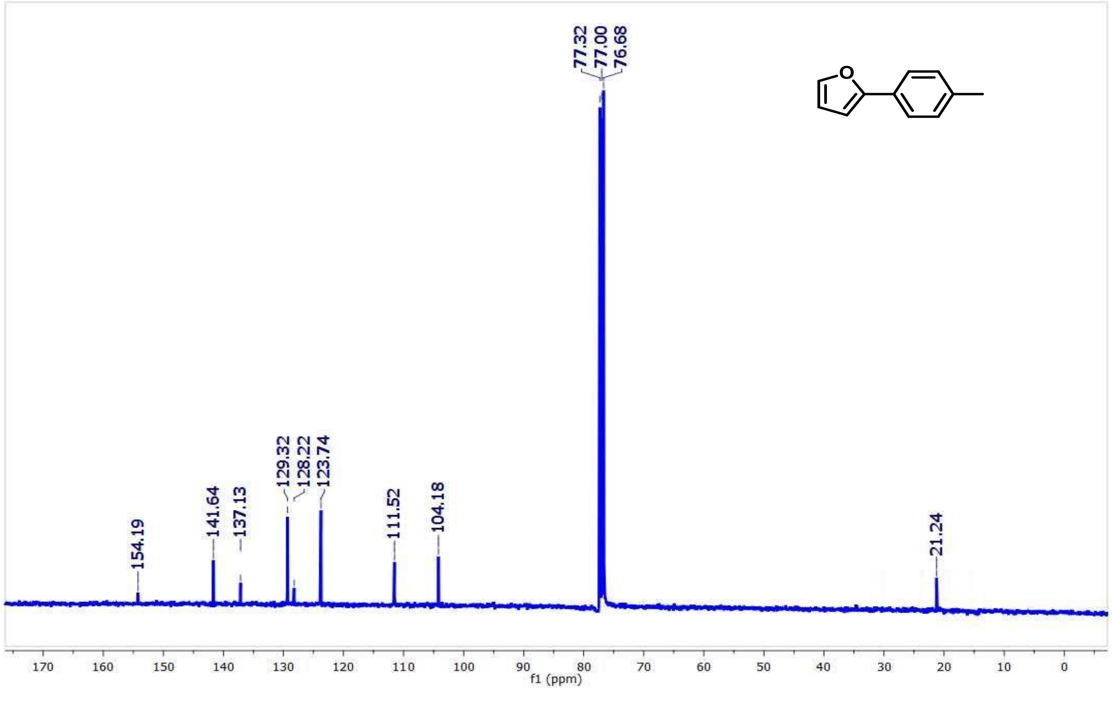

Figure S61. ^{13}C NMR ($CDCl_3$) spectrum of 2-(4-methylphenyl)furan (**5f**).

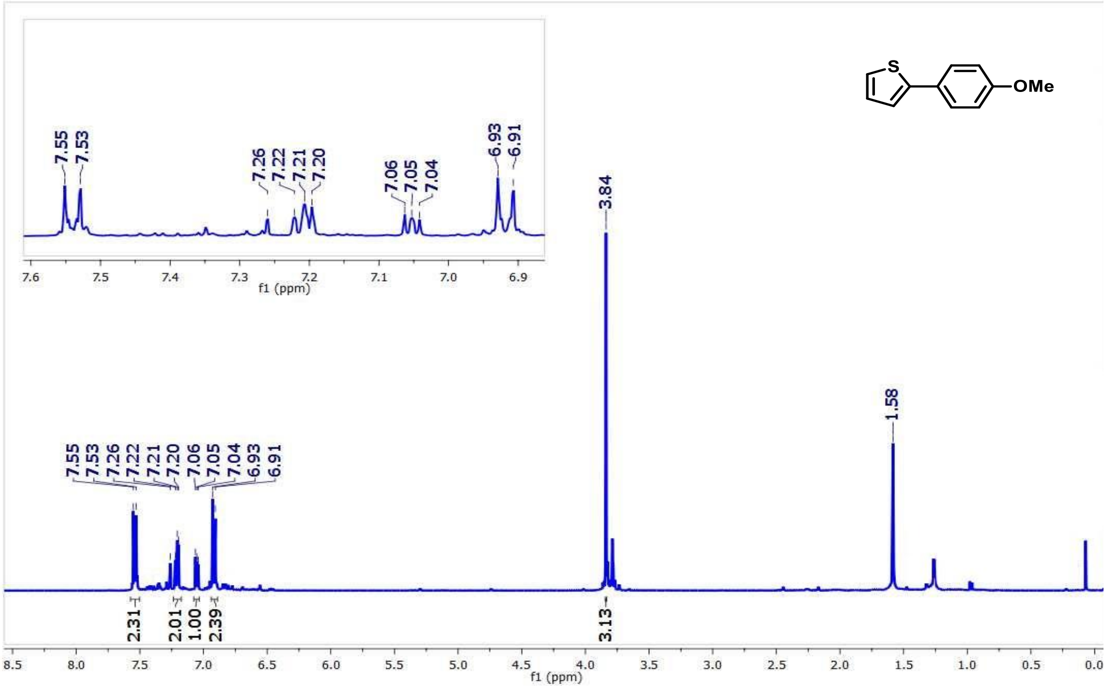

Figure S62. ^{1}H NMR ($CDCl_3$) spectrum of 2-(4-methoxyphenyl)thiophene (**6b**).

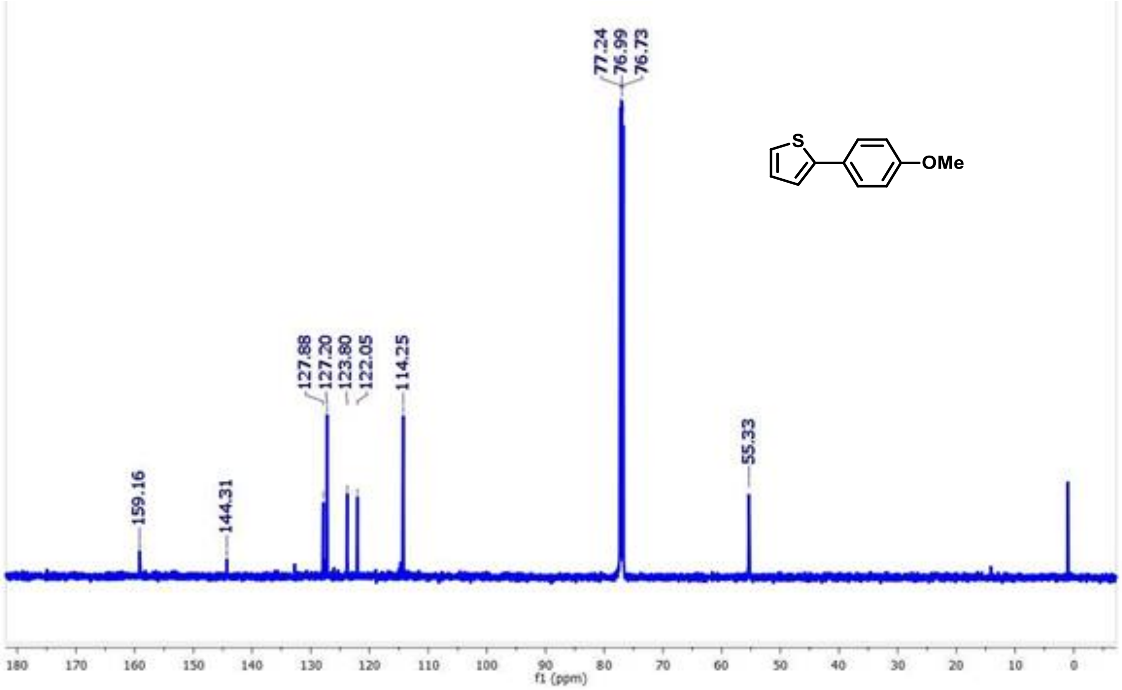

Figure S63. ^{13}C NMR ($CDCl_3$) spectrum of 2-(4-methoxyphenyl)thiophene (**6b**).

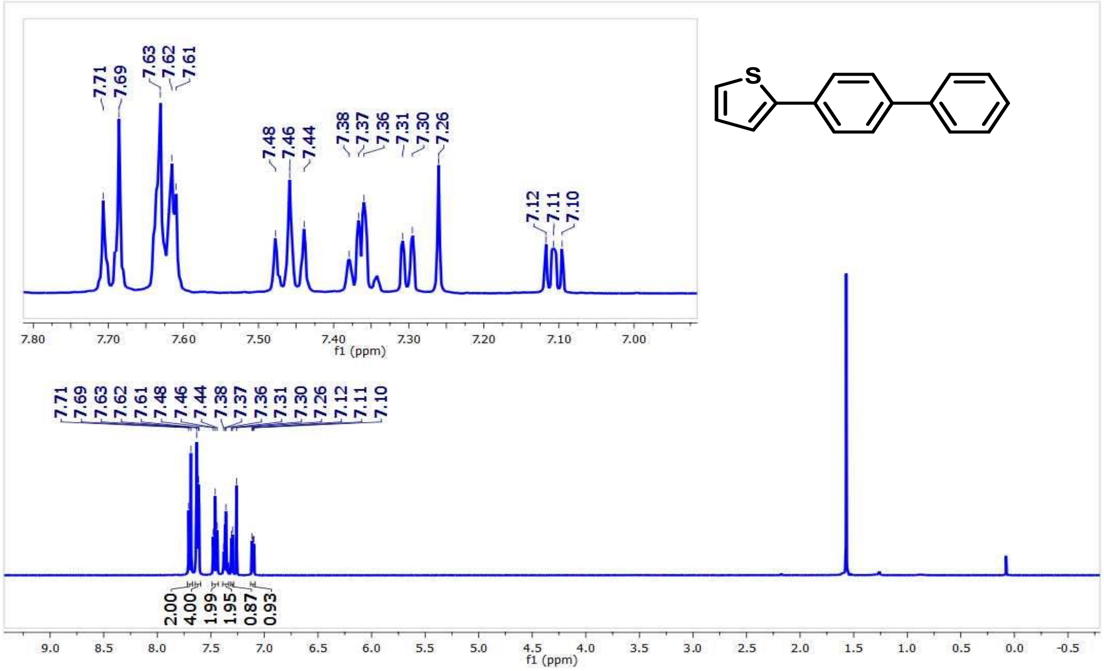

Figure S64. ^{1}H NMR ($CDCl_3$) spectrum of 2-biphenylthiophene (**6e**).

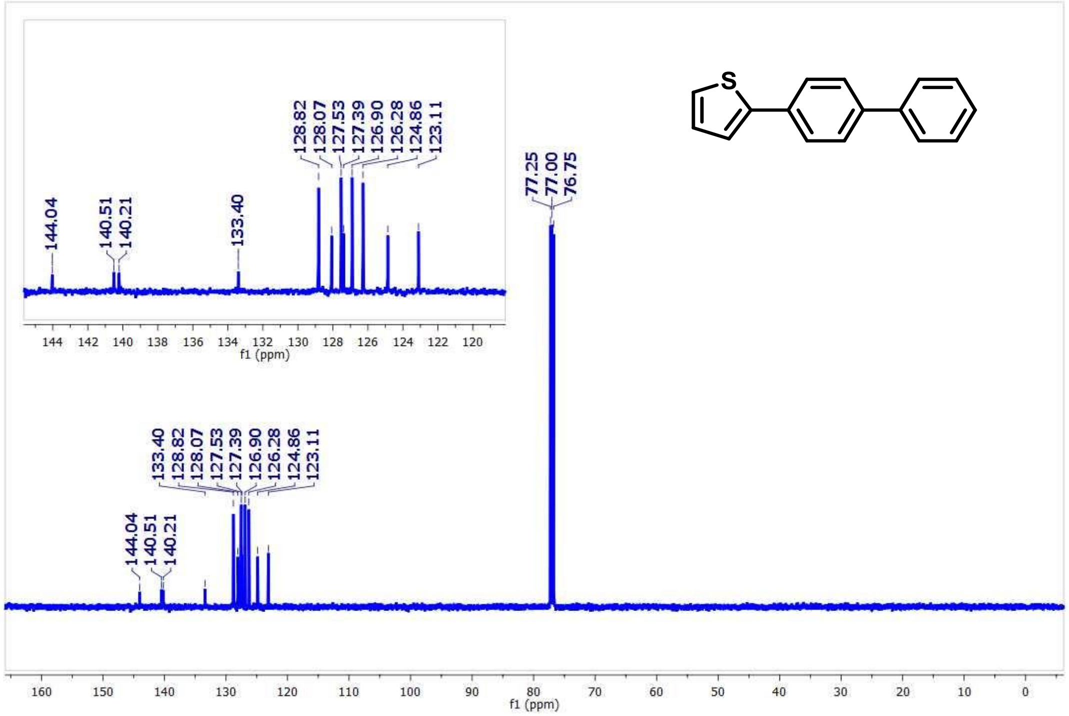

Figure S65. ^{13}C NMR ($CDCl_3$) spectrum of 2-biphenylthiophene (**6e**).

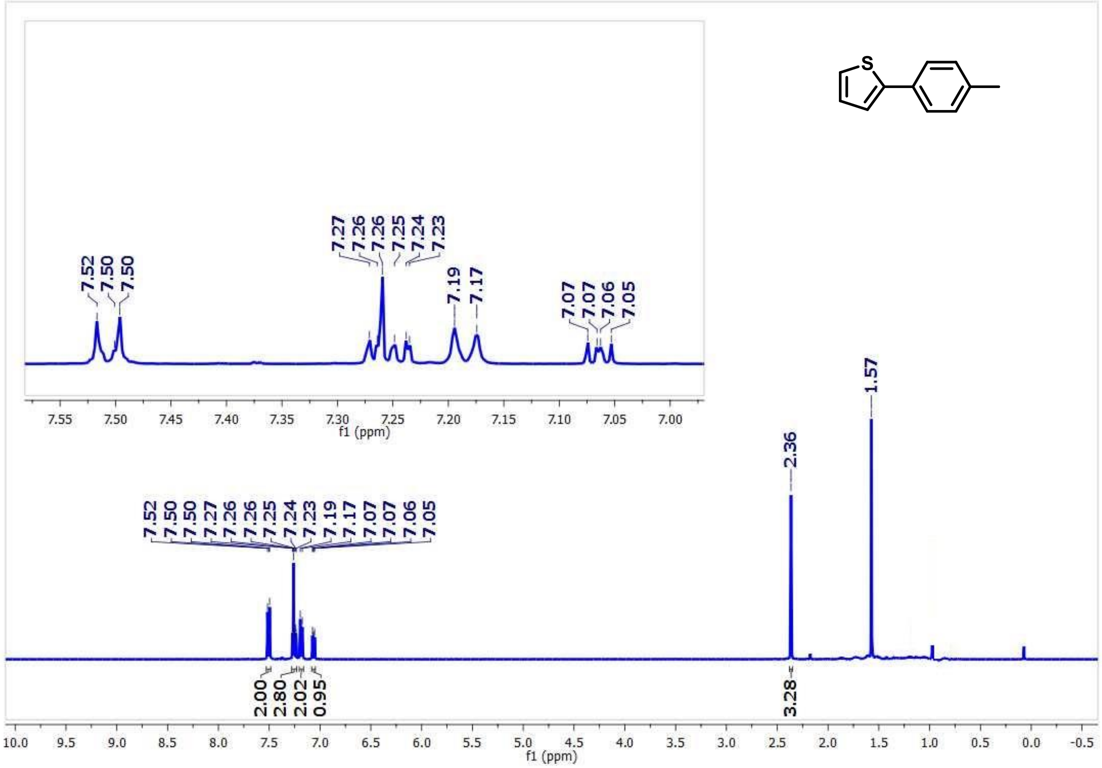

Figure S66. ^{1}H NMR ($CDCl_3$) spectrum of 2-(4-methylphenyl)thiophene (**6f**).

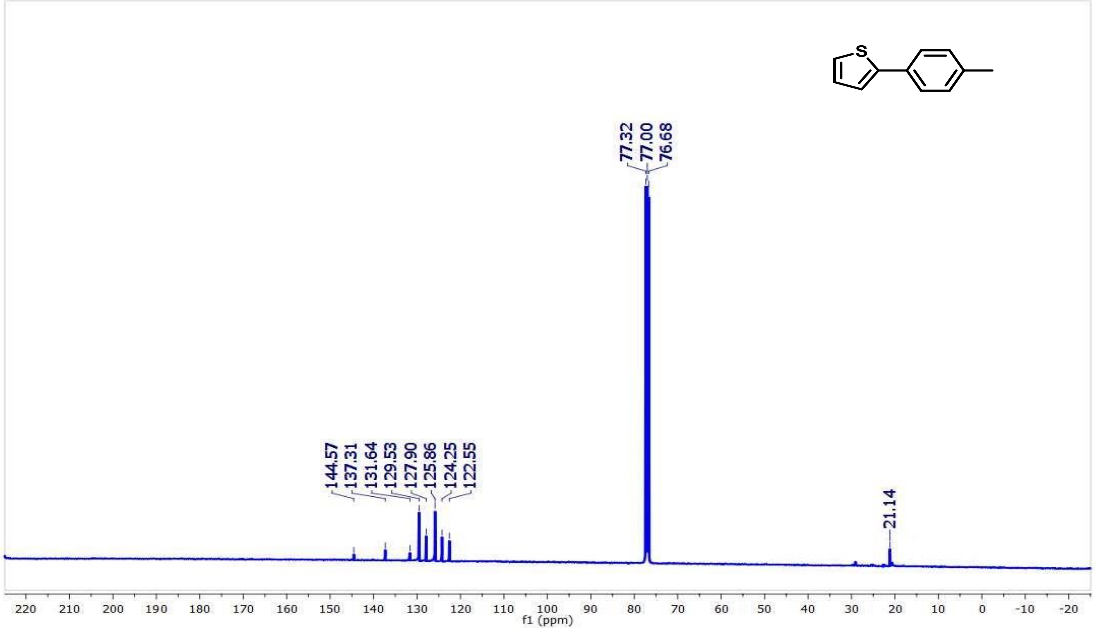

Figure S67. ^{13}C NMR ($CDCl_3$) spectrum of 2-(4-methylphenyl)thiophene (**6f**).

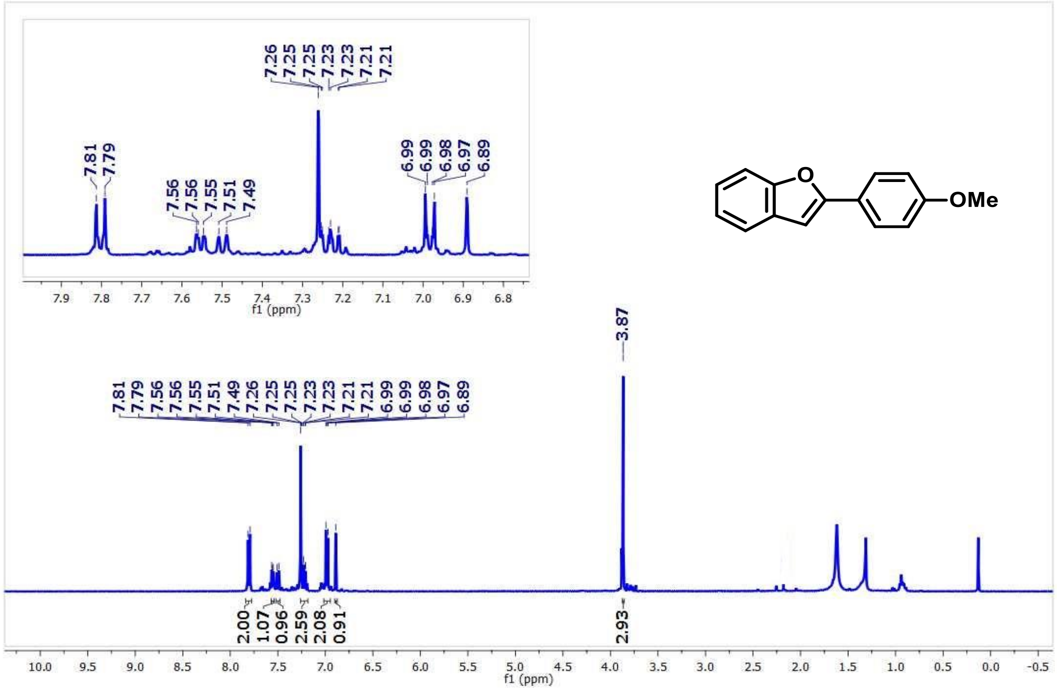

Figure S68. ^{1}H NMR ($CDCl_3$) spectrum of 4-(benzofuran-2-yl)anisole (**7b**).

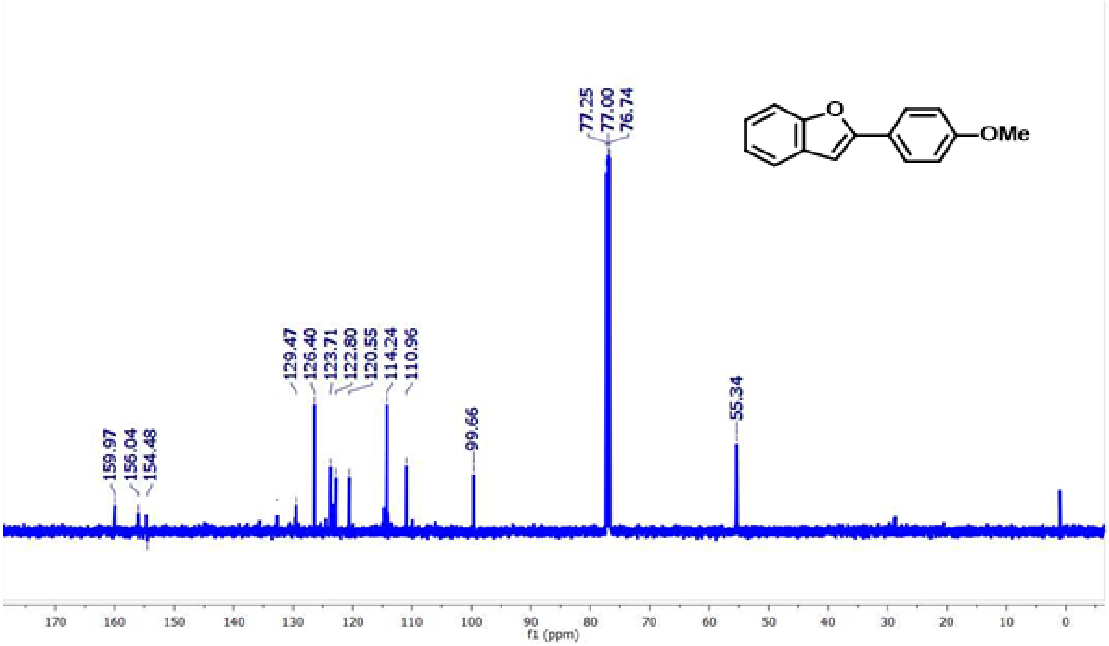

Figure S69. ^{13}C NMR ($CDCl_3$) spectrum of 4-(benzofuran-2-yl)anisole (**7b**).

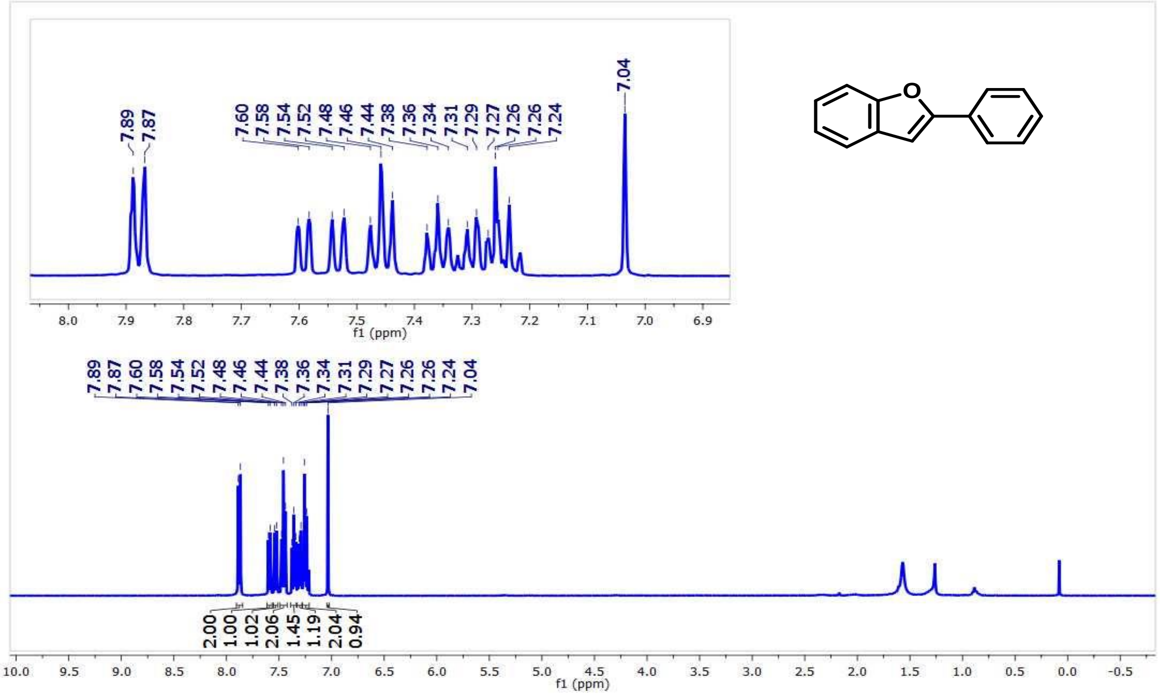

Figure S70. ^{1}H NMR ($CDCl_3$) spectrum of 4-(benzofuran-2-yl)benzene (**7c**).

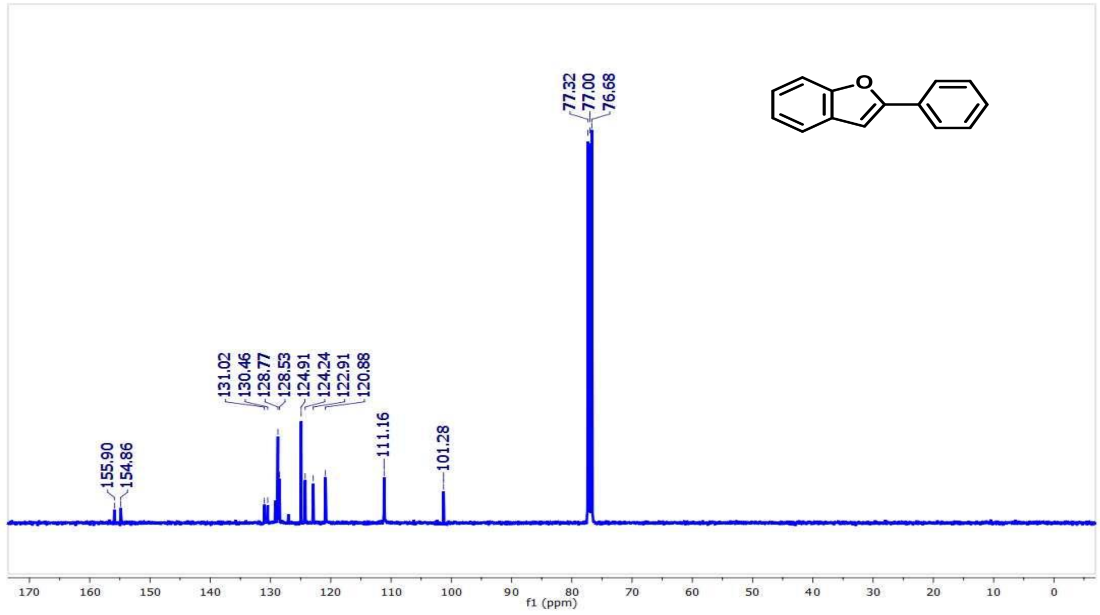

Figure S71. ^{13}C NMR ($CDCl_3$) spectrum of 4-(benzofuran-2-yl)benzene (**7c**).

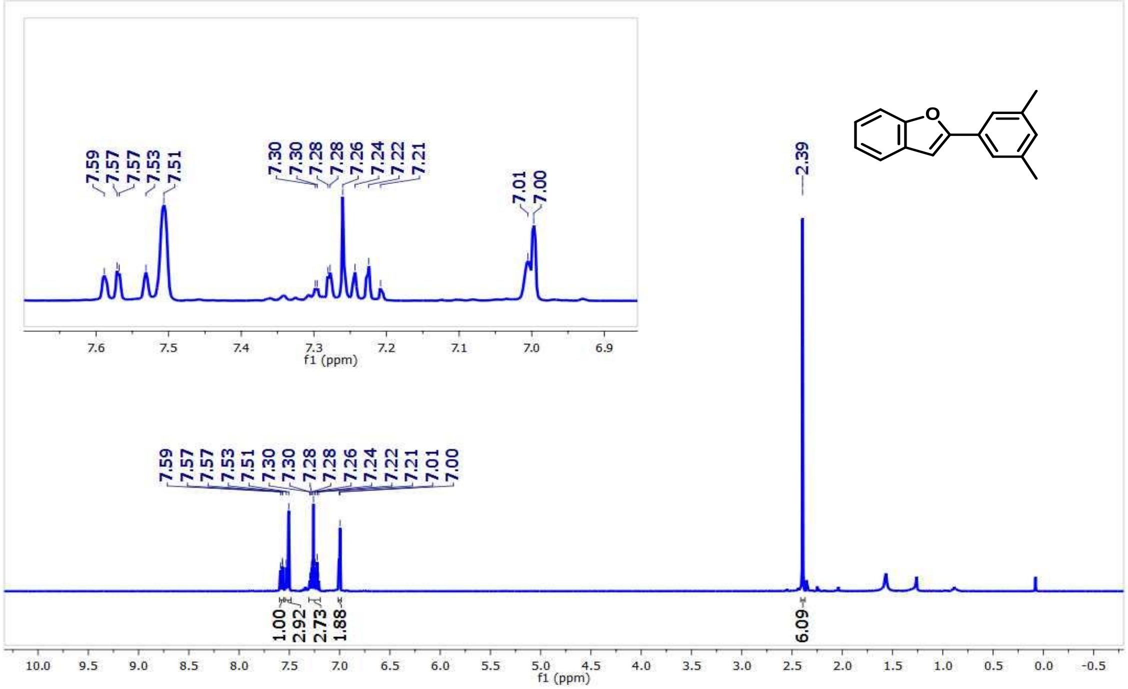

Figure S72. ^{1}H NMR ($CDCl_3$) spectrum of 4-(benzofuran-2-yl)-3, 5-dimethylbenzene (**7d**).

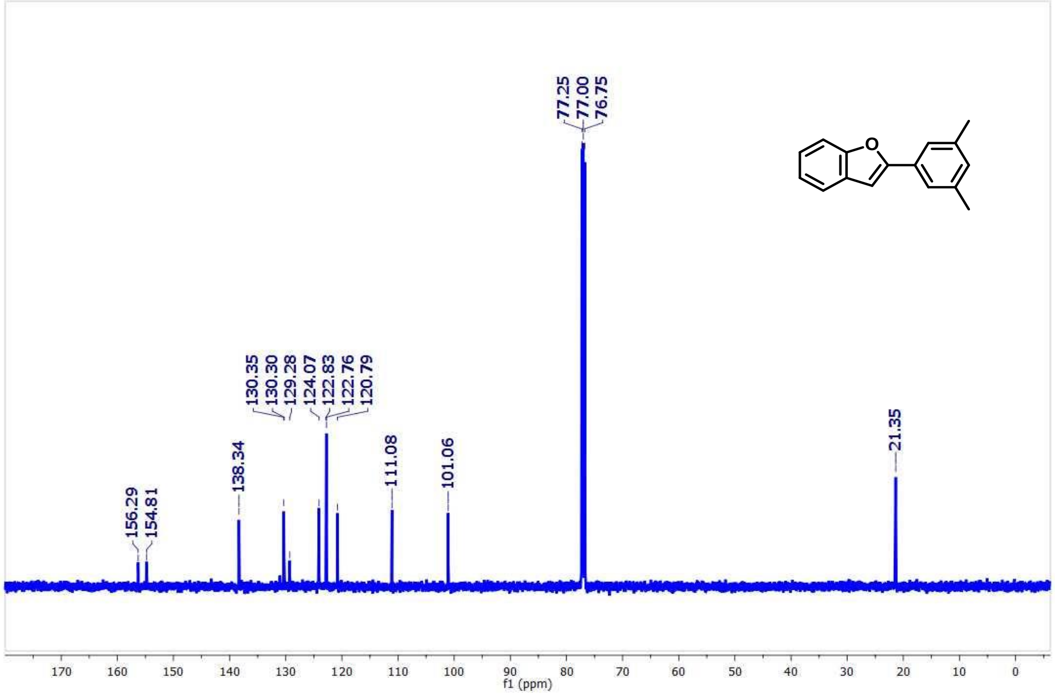

Figure S73. ^{13}C NMR ($CDCl_3$) spectrum of 4-(benzofuran-2-yl)-3, 5-dimethylbenzene (**7d**).

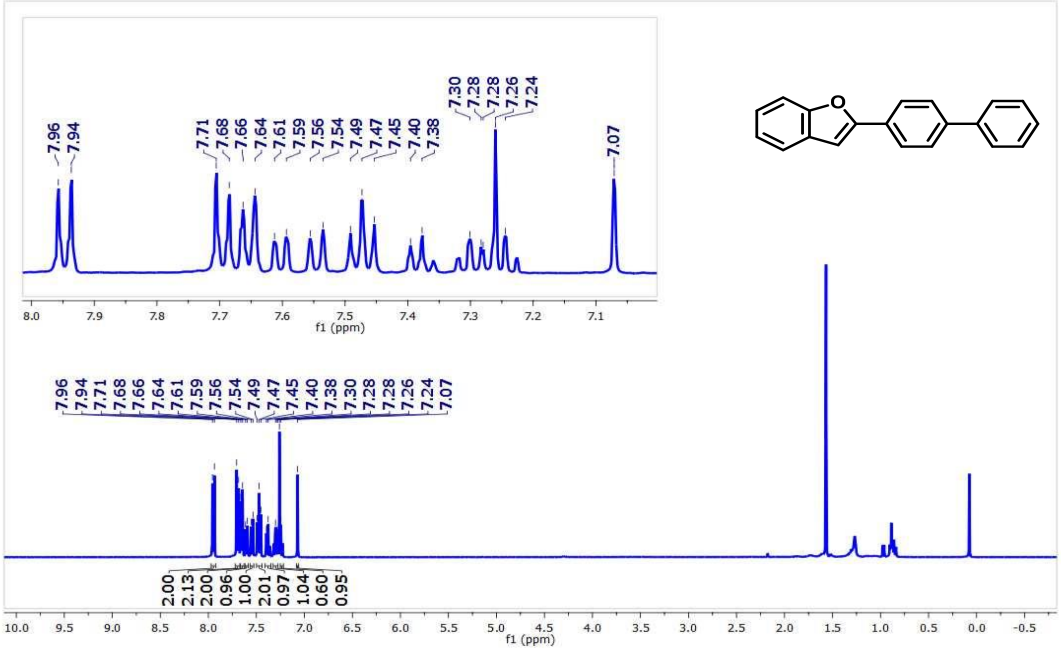

Figure S74. ^{1}H NMR ($CDCl_3$) spectrum of 4-(benzofuran-2-yl)biphenyl (**7e**).

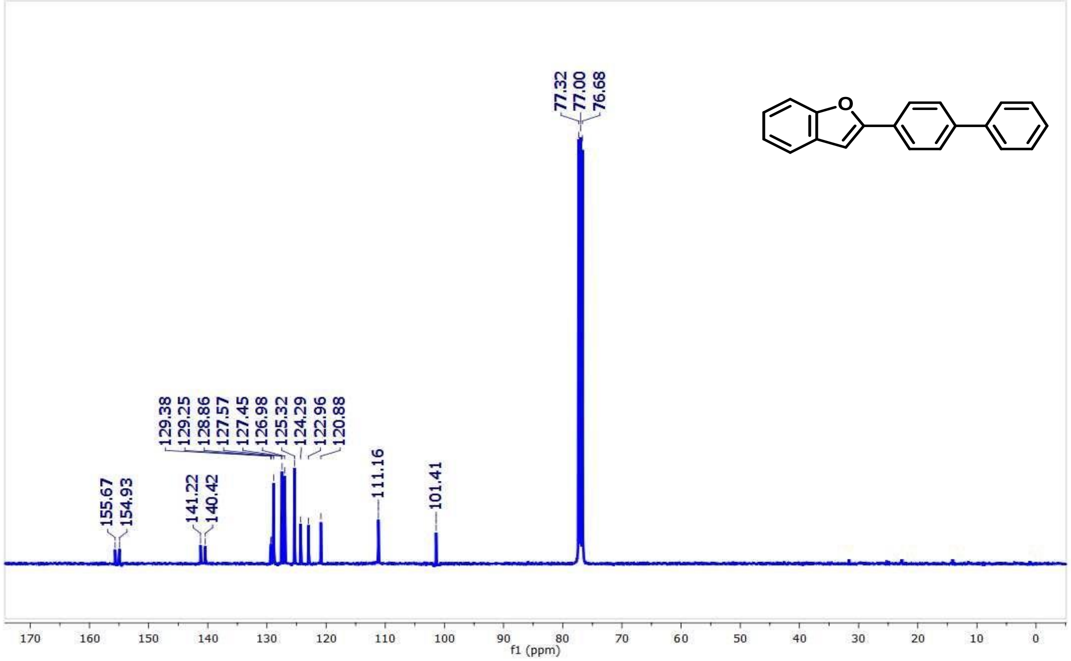

Figure S75. ^{13}C NMR ($CDCl_3$) spectrum of 4-(benzofuran-2-yl)biphenyl (**7e**).

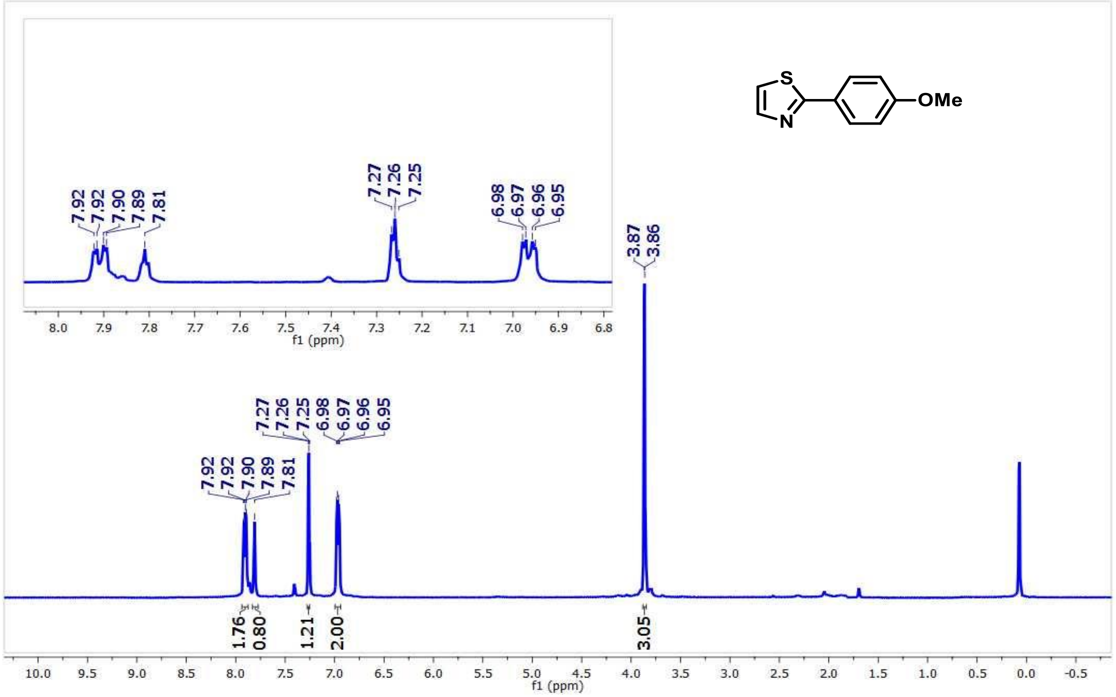

Figure S76. ^{1}H NMR ($CDCl_3$) spectrum of 2-(4-methoxyphenyl)thiazole (**11b**).

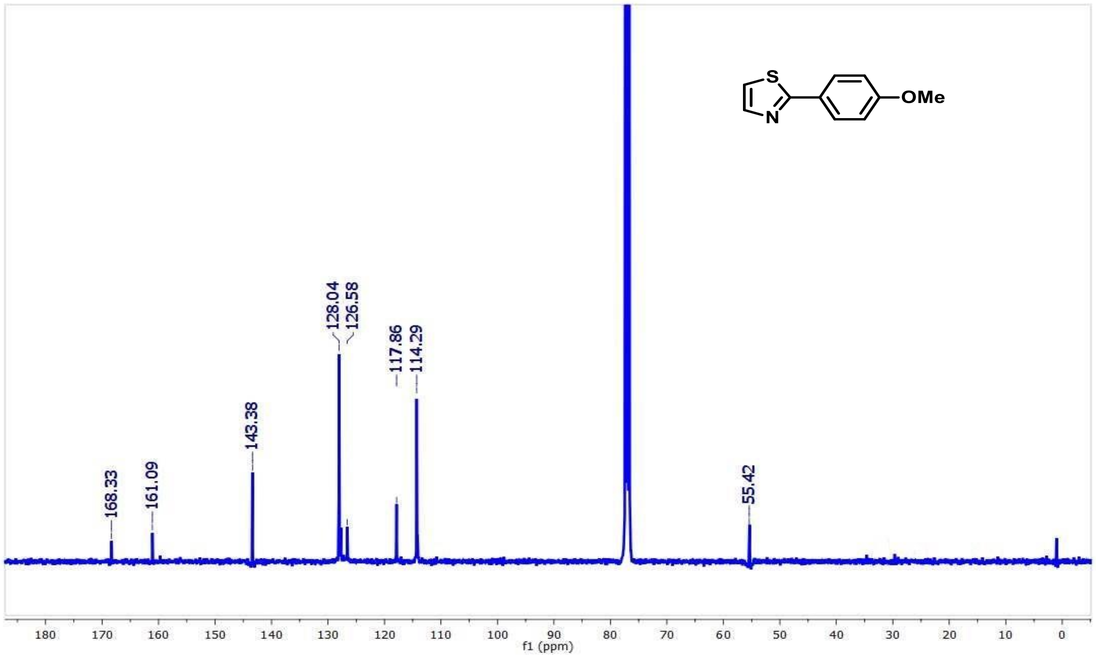

Figure S77. ^{13}C NMR ($CDCl_3$) spectrum of 2-(4-methoxyphenyl)thiazole (**11b**).

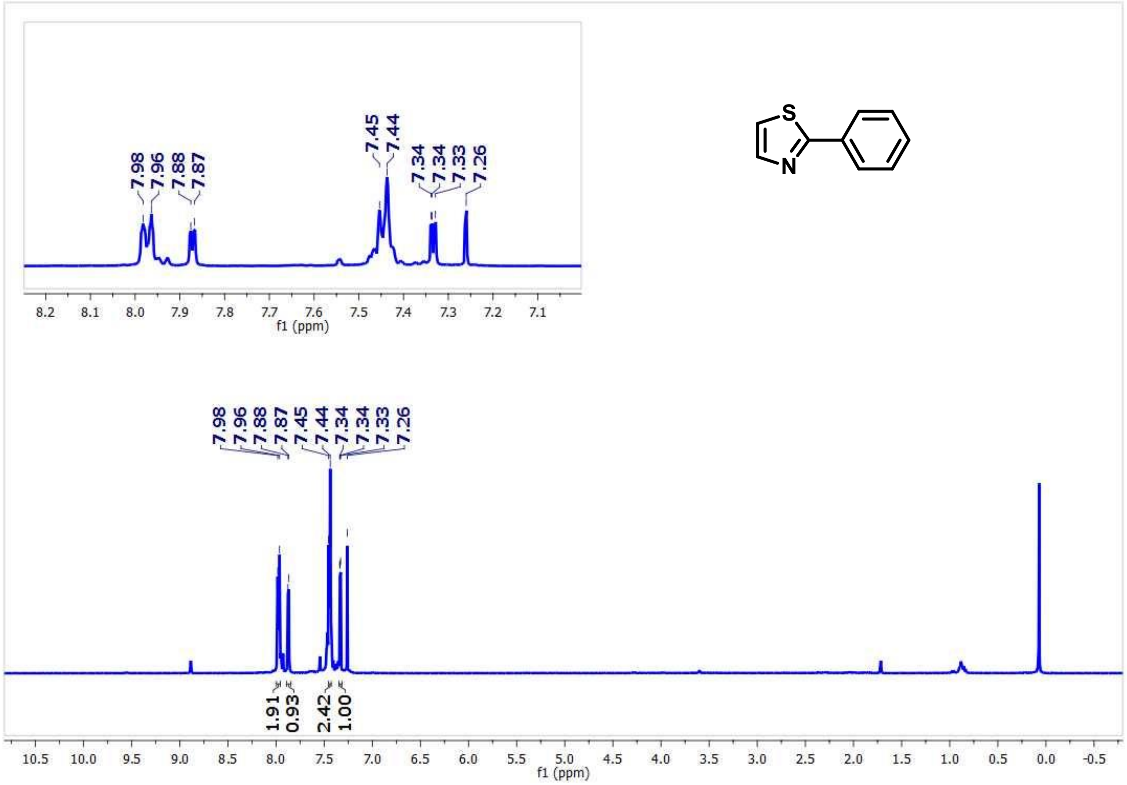

Figure S78. ^{1}H NMR ($CDCl_3$) spectrum of 2-phenylthiazole (**11c**).

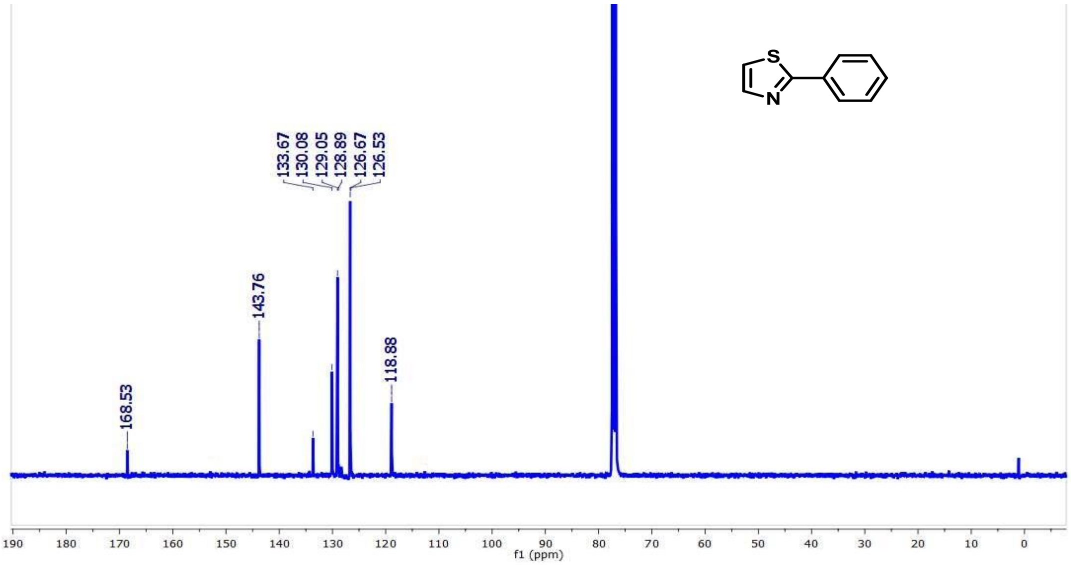

Figure S79. ^{13}C NMR ($CDCl_3$) spectrum of 2-phenylthiazole (**11c**).

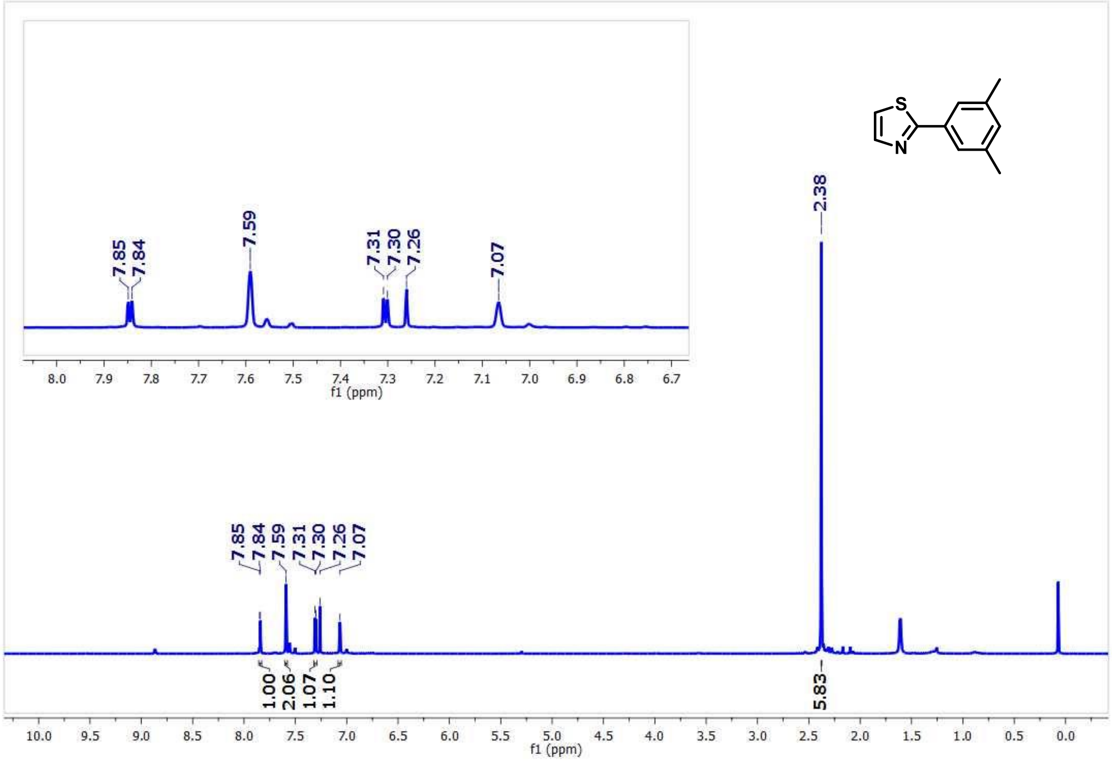

Figure S80. ^{1}H NMR ($CDCl_3$) spectrum of 2-(3, 5-dimethylphenyl)thiazole (**11d**).

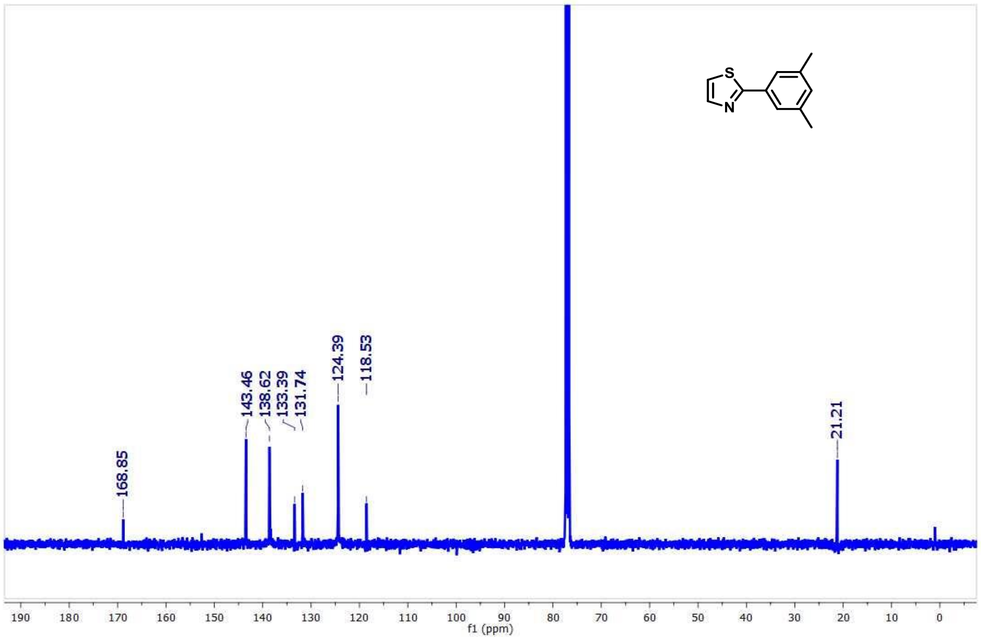

Figure S81. ^{13}C NMR ($CDCl_3$) spectrum of 2-(3, 5-dimethylphenyl)thiazole (**11d**).

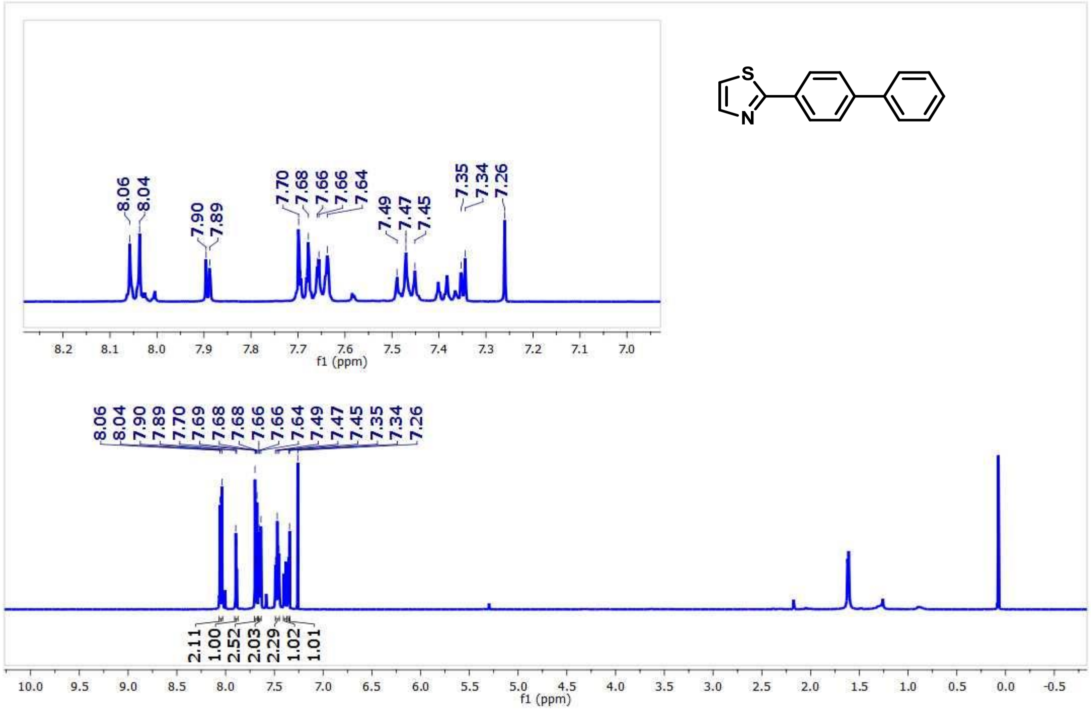

Figure S82. ^{1}H NMR ($CDCl_3$) spectrum of 2-biphenylthiazole (**11e**).

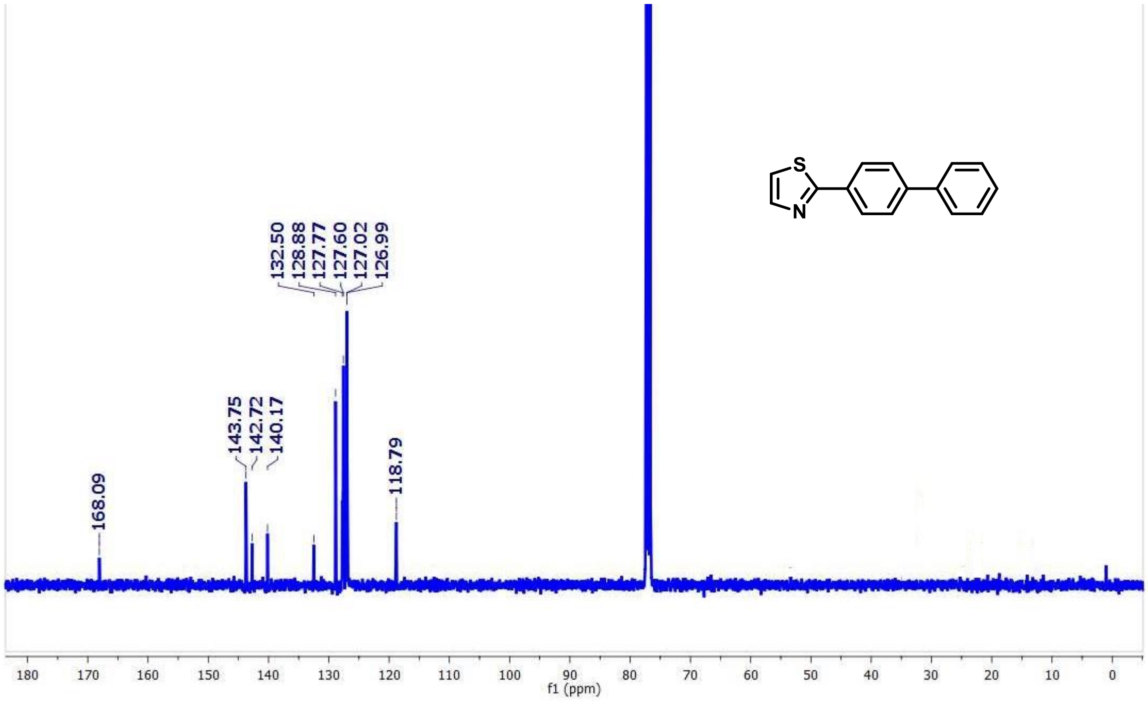

Figure S83. ^{13}C NMR ($CDCl_3$) spectrum of 2-biphenylthiazole (**11e**).

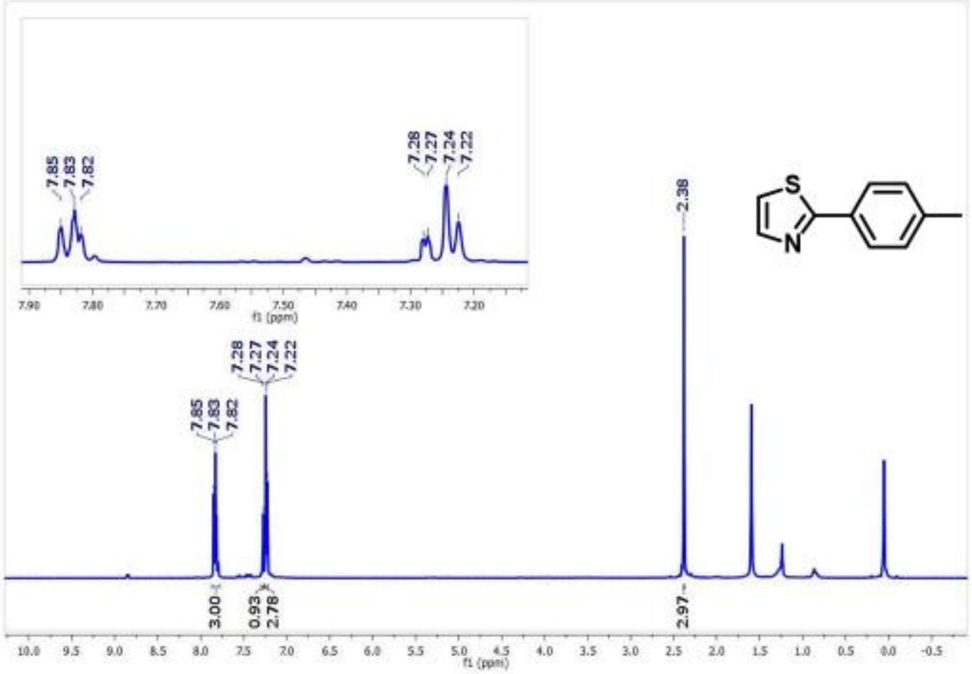

Figure S84. ^{1}H NMR ($CDCl_3$) spectrum of 2-(4-methylphenyl)thiazole (**11f**).

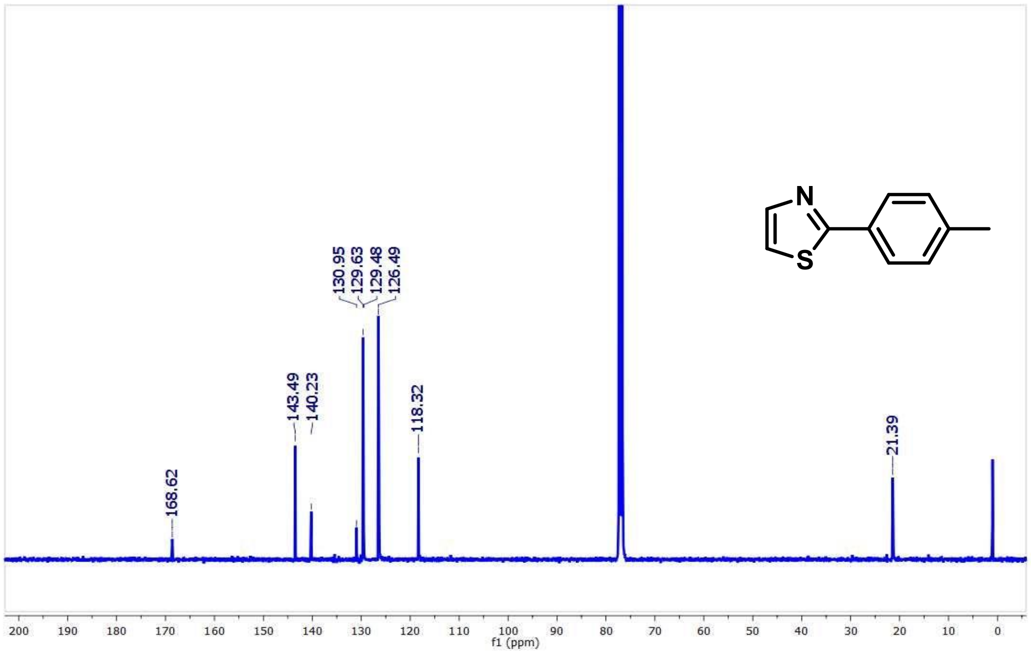

Figure S85. ^{13}C NMR ($CDCl_3$) spectrum of 2-(4-methylphenyl)thiazole (**11f**).

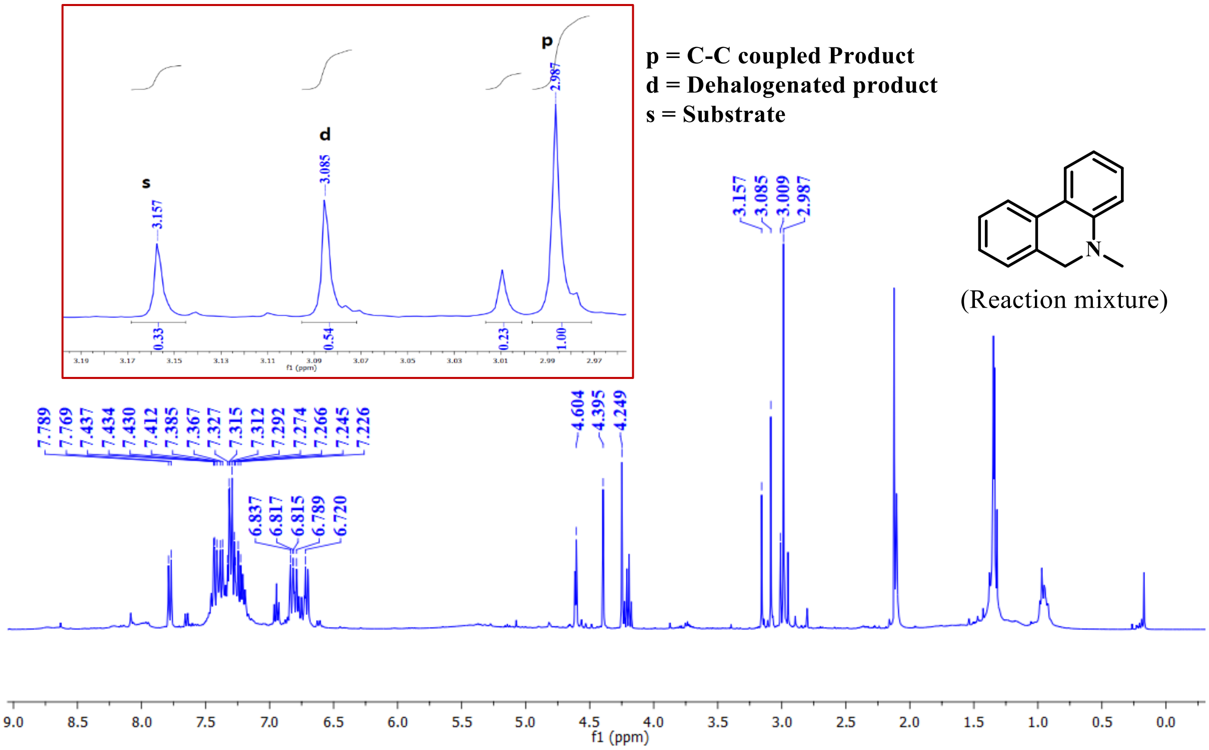

Figure S86. Reaction mixture ^{1}H NMR ($CDCl_3$) spectrum of 5-methyl-5,6-dihydrophenanthridine (**12a**).

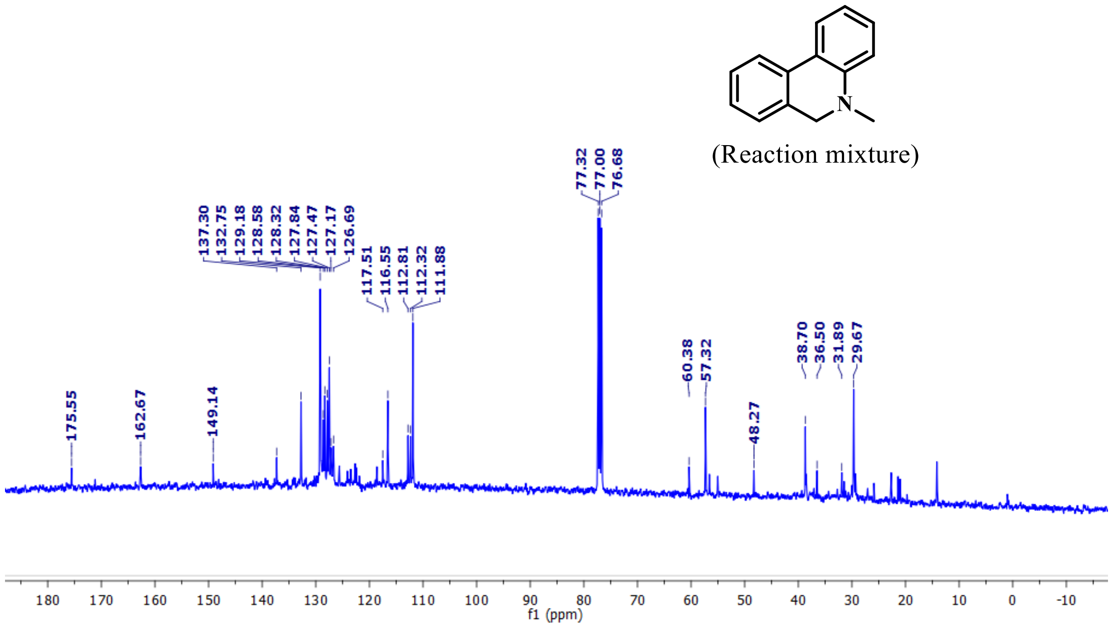

Figure S87. Reaction mixture ^{13}C NMR ($CDCl_3$) spectrum of 5-methyl-5,6-dihydrophenanthridine (**12a**).

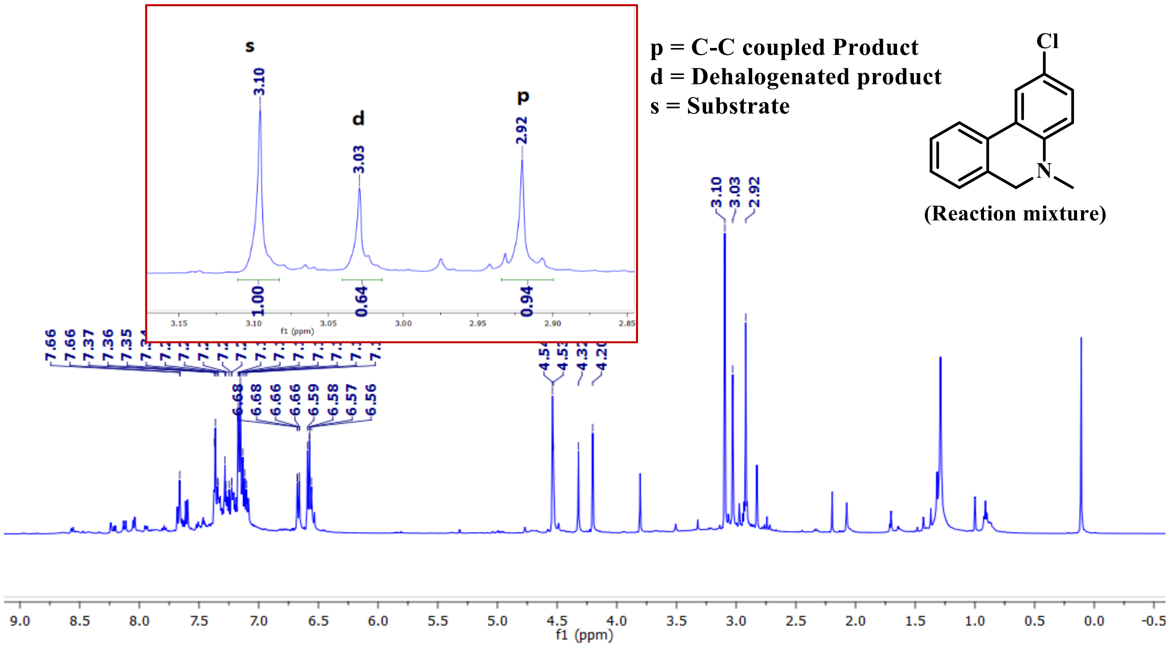

Figure S88. Reaction mixture ^{1}H NMR ($CDCl_3$) spectrum of 2-chloro-5-methyl-5,6-dihydrophenanthridine (**12b**).

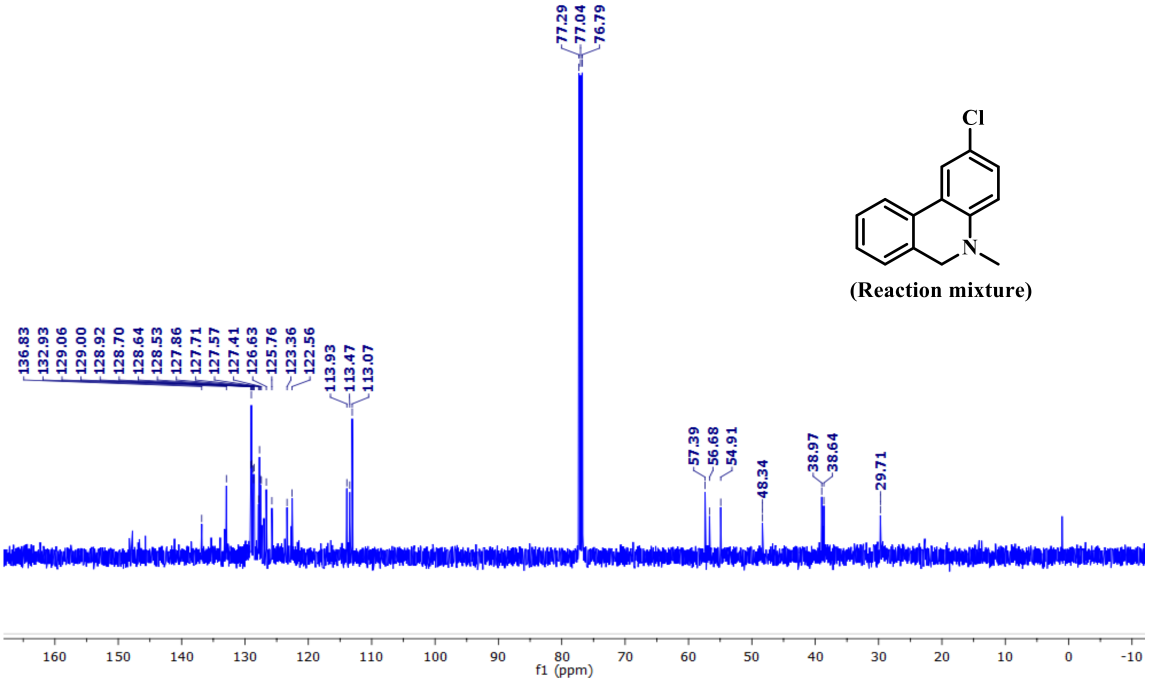

Figure S89. Reaction mixture ^{13}C NMR ($CDCl_3$) spectrum of 2-chloro-5-methyl-5,6-dihydrophenanthridine (**12b**).

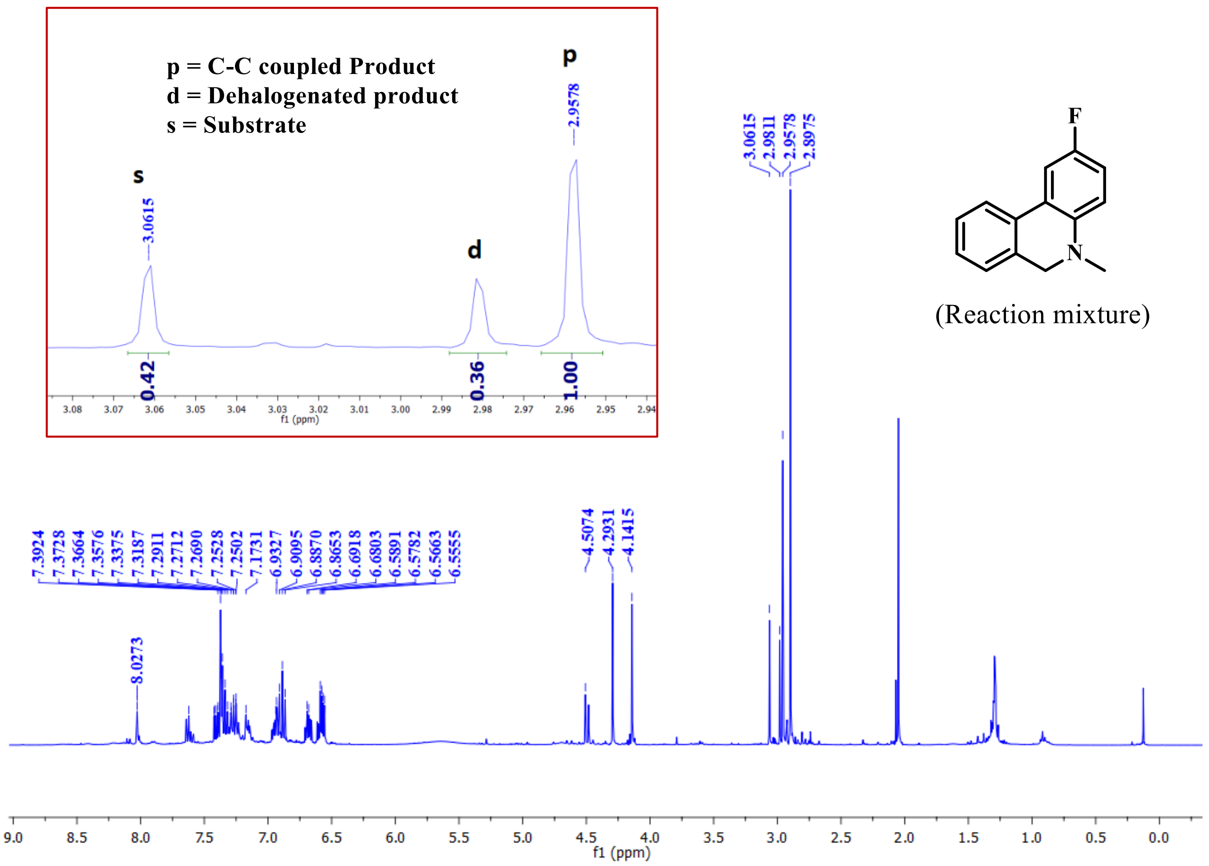

Figure S90. Reaction mixture ^{1}H NMR ($CDCl_3$) spectrum of 2-fluoro-5-methyl-5,6-dihydrophenanthridine (**12c**).

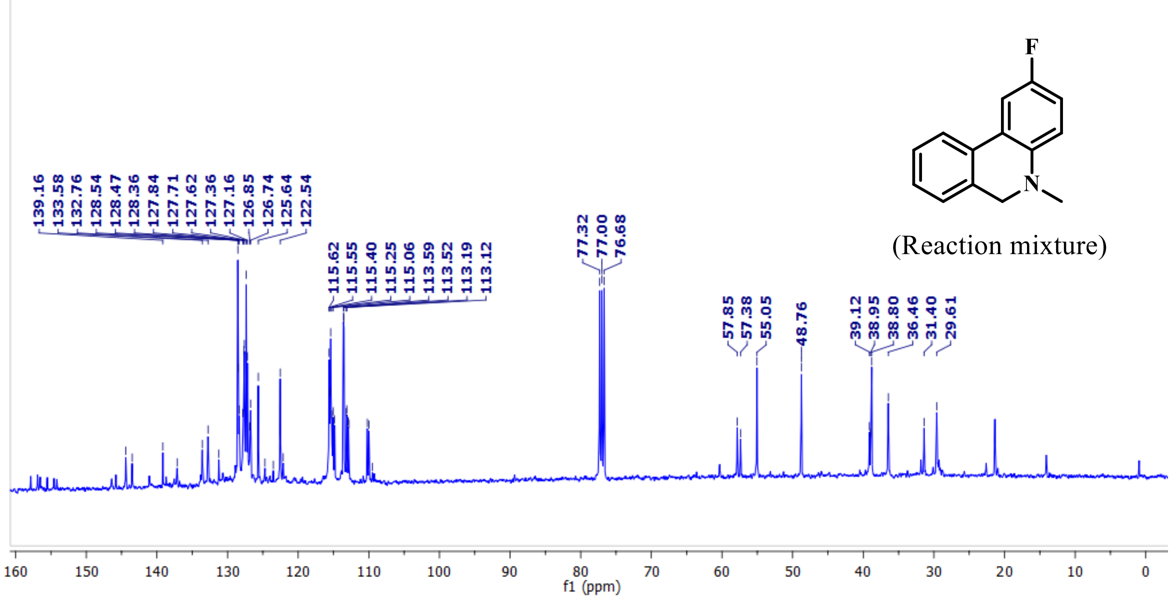

Figure S91. Reaction mixture ^{13}C NMR ($CDCl_3$) spectrum of 2-fluoro-5-methyl-5,6-dihydrophenanthridine (**12c**).

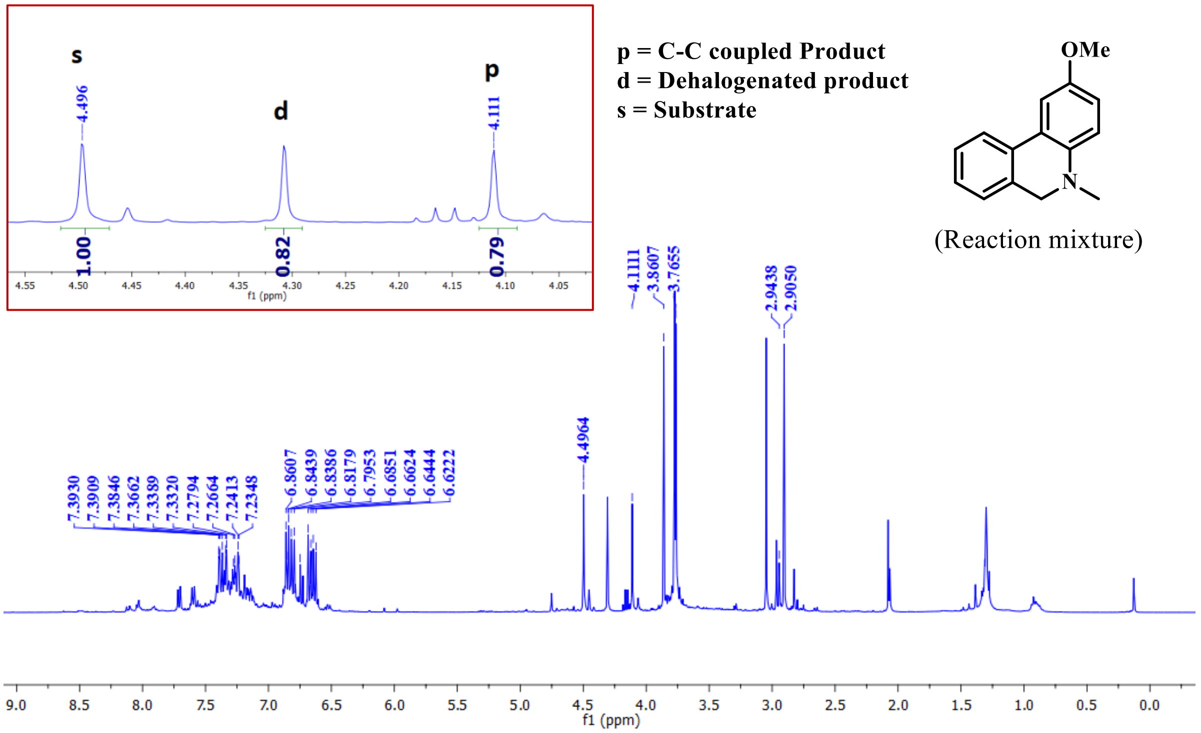

Figure S92. Reaction mixture ^{1}H NMR ($CDCl_3$) spectrum of 2-methoxy-5-methyl-5,6-dihydrophenanthridine (**12d**).

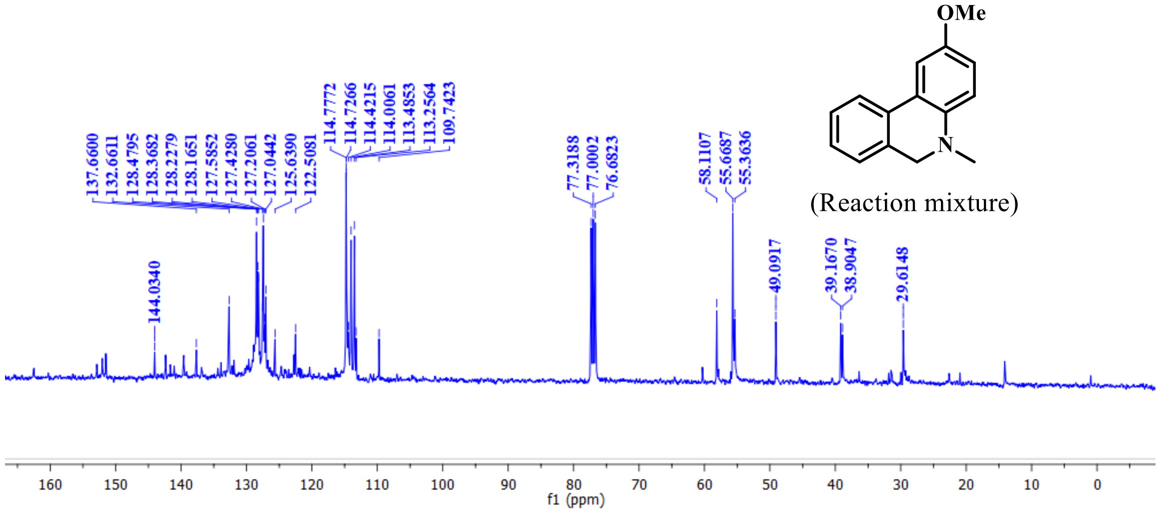

Figure S93. Reaction mixture ^{13}C NMR ($CDCl_3$) spectrum of 2-methoxy-5-methyl-5,6-dihydrophenanthridine (**12d**).

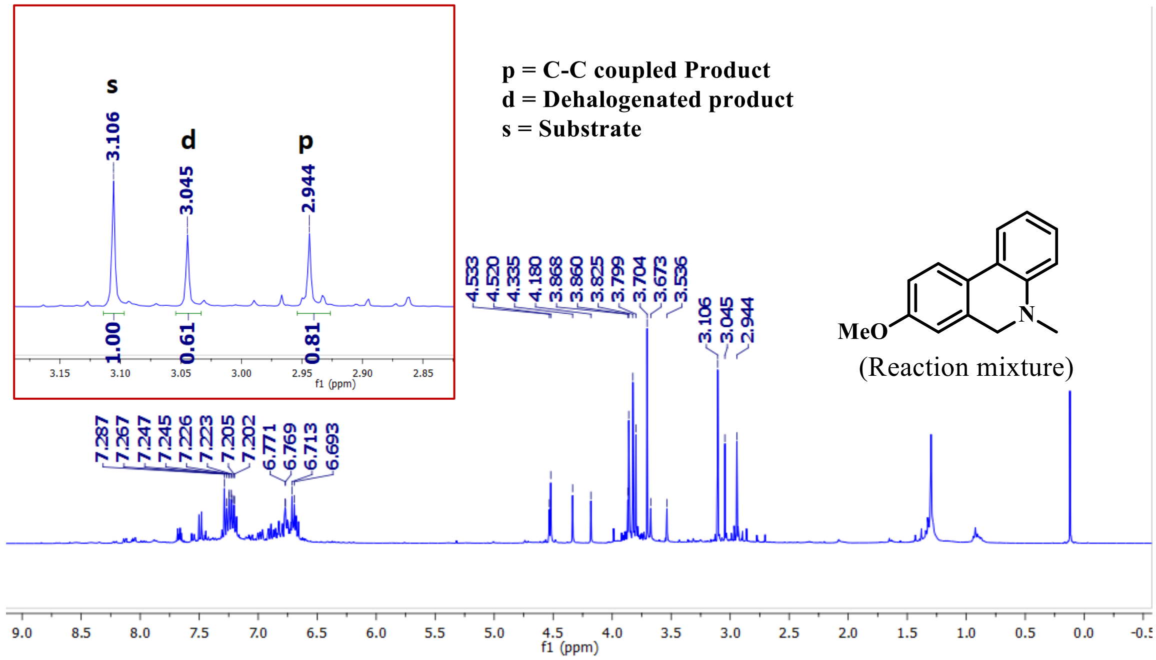

Figure S94. Reaction mixture ^{1}H NMR ($CDCl_3$) spectrum of 8-methoxy-5-methyl-5,6-dihydrophenanthridine (**12e**).

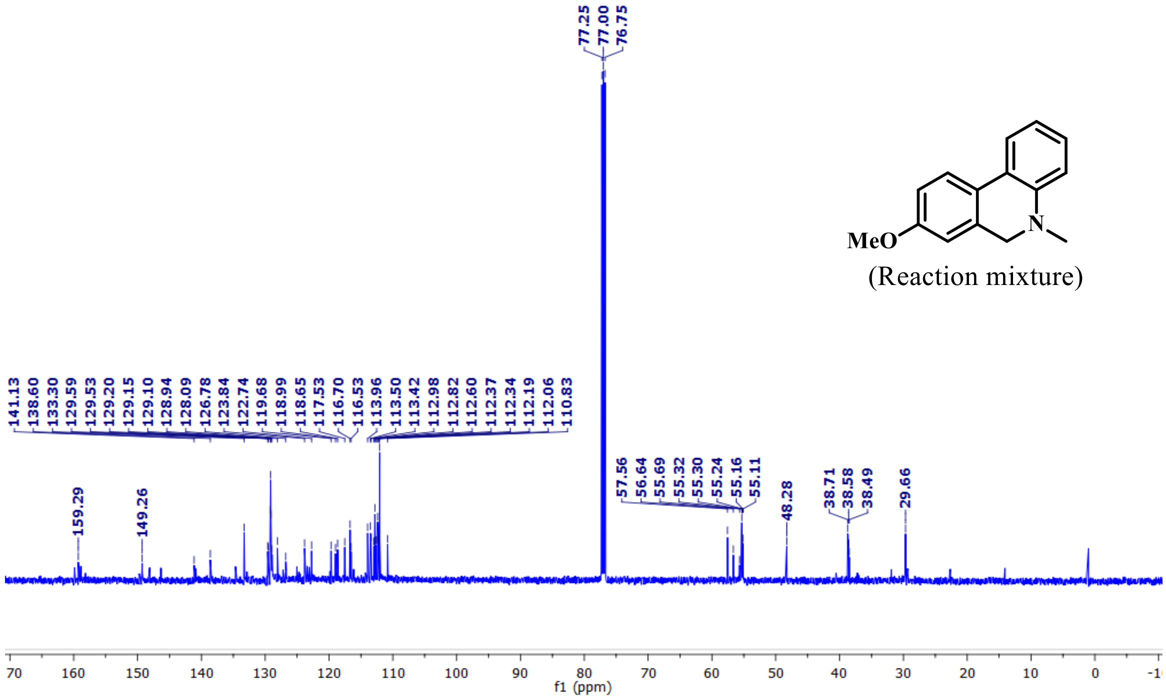

Figure S95. Reaction mixture ^{13}C NMR ($CDCl_3$) spectrum of 8-methoxy-5-methyl-5,6-dihydrophenanthridine (**12e**).

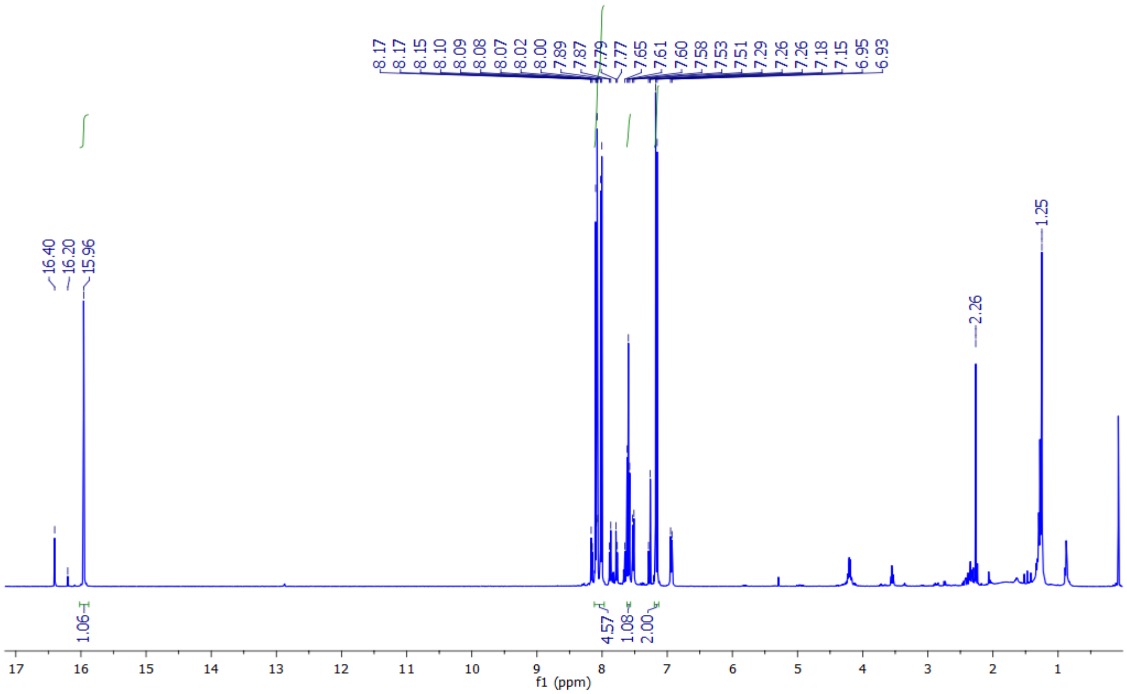

Figure S96. ^{1}H NMR ($CDCl_3$) spectrum of the phenalenyl parts, obtained after column chromatography of the catalytic reaction mixture.

23. Mass spectra:

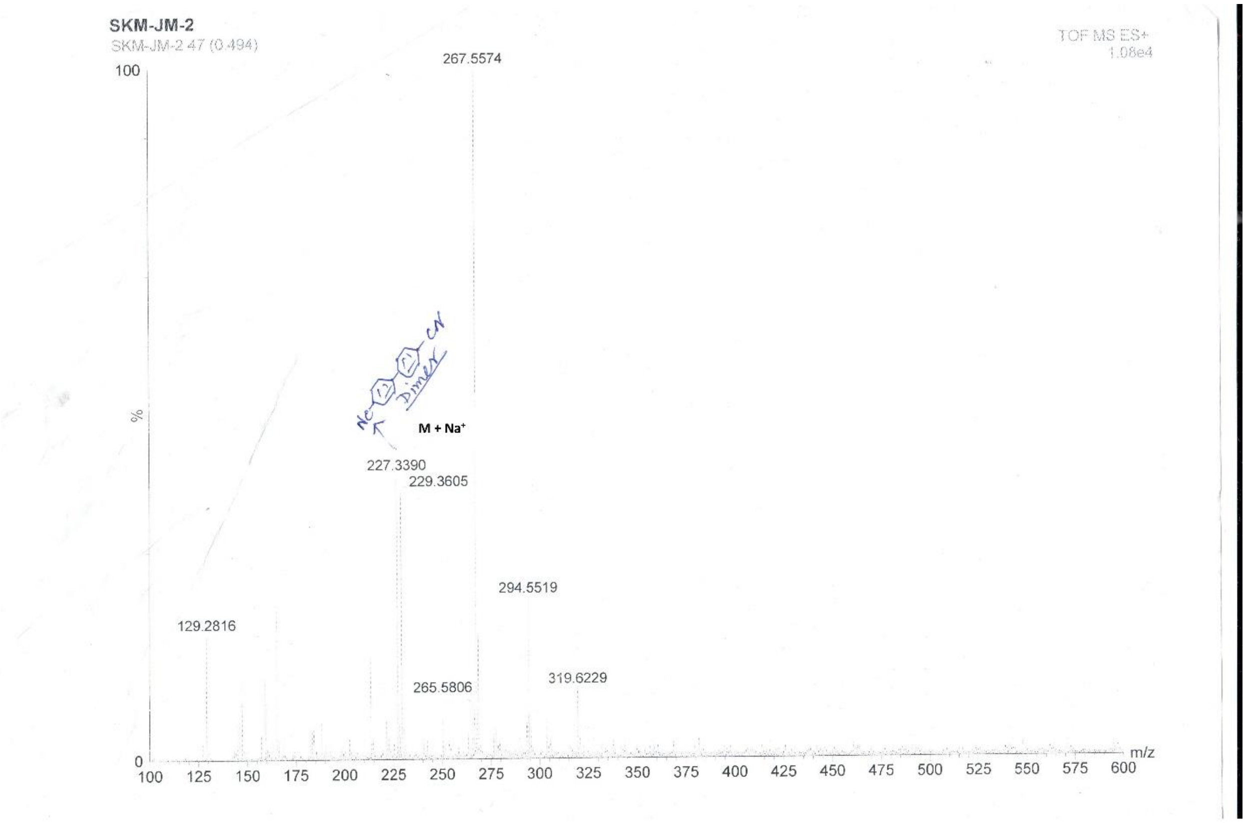

Figure S97. Mass spectrum of reaction mixture for the chlorobenzonitrile by doubly-reduced PLY(O,O)-K complex.

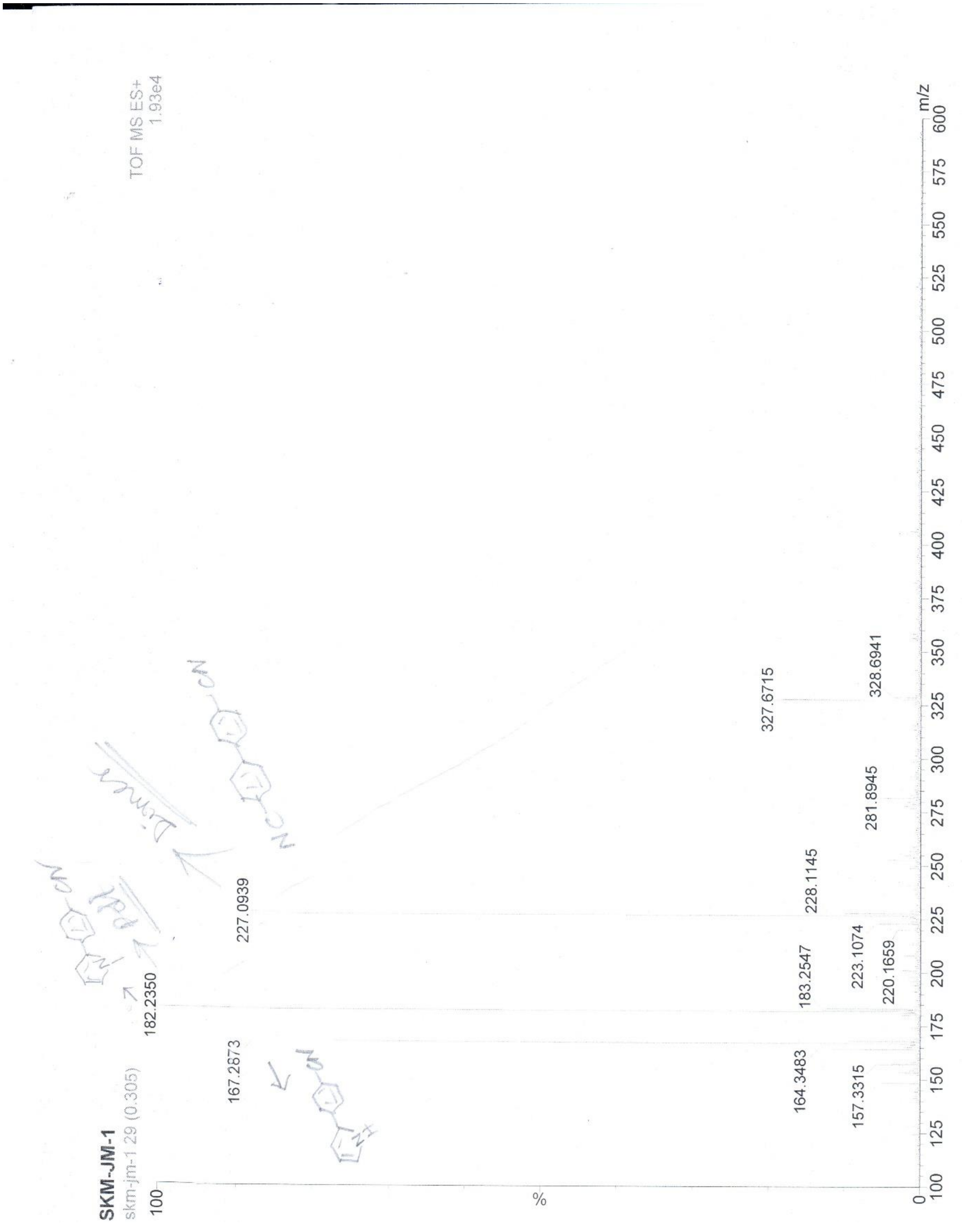

Figure S98. Reaction mixture mass spectrum of a catalytic reaction between N-methylpyrrole and chlorobenzonitrile.

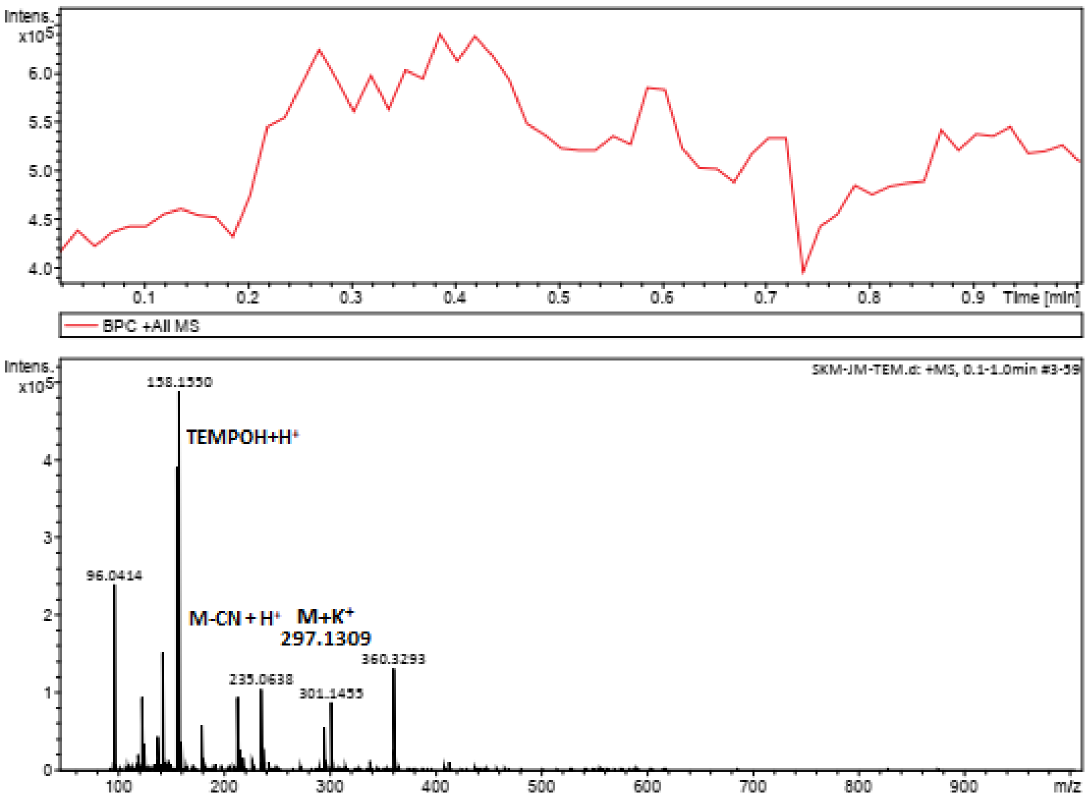

Figure S99. Mass spectrum of reaction mixture for radical trapping experiment with TEMPO radical.

24. Computational study for the catalytic reactions:

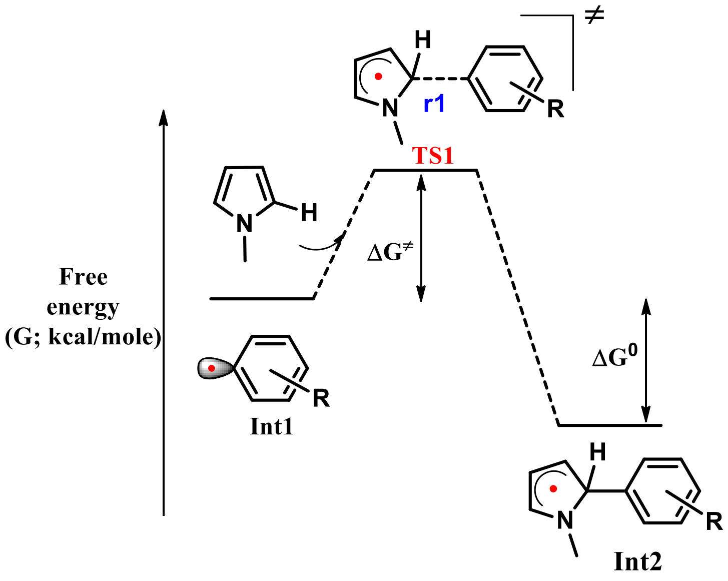

Figure S100. Energy profile diagram for aryl radical attack to N-methylpyrrole.

Substitution (R)	Energy barrier ($\Delta G^{\neq}$) (kcal/mole)	Total Gibbs free energy change (ΔG^{0}) (kcal/mole)
R = 3, 5-diMe	15.5	-9.78
R = 4-Me	14.2	-11.6
R = 4-H	14.9	-11.1
R = 4-OMe	14.4	-12.2
R = 4-Ph	13.9	-11.7

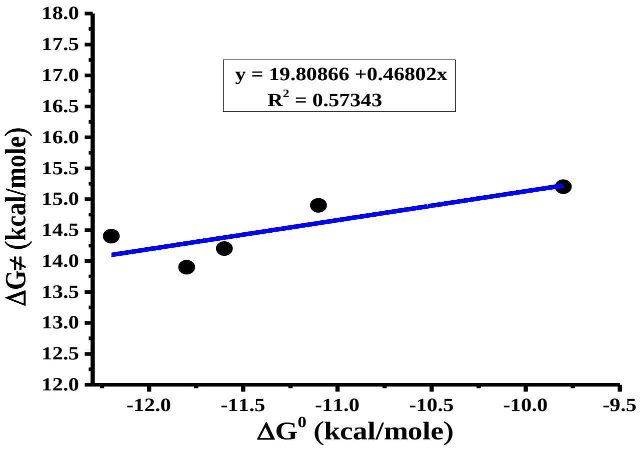

Figure S101. Plot of reaction exothermicity and transition state energy barriers.

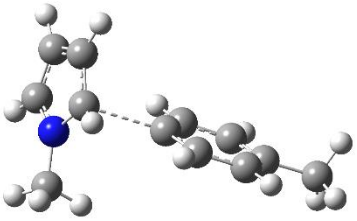

TS1(Me)

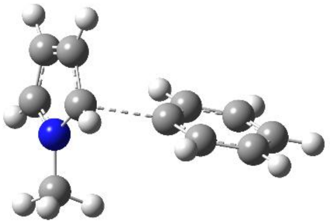

TS1(H)

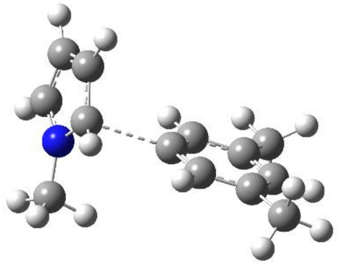

TS1(diMe)

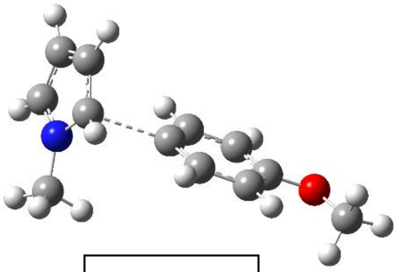

TS1(OMe)

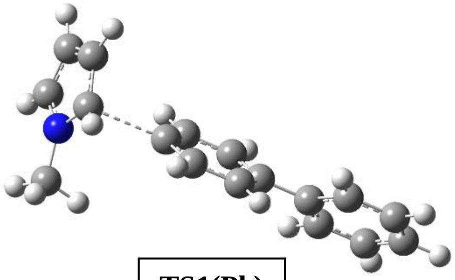

TS1(Ph)

Figure S102: Optimized geometries the transition states of with different aryl radical corresponding to **TS1**.

Table S7: Energies, enthalpies, and free energies (in Hartree) of the optimized structures of all transition states and intermediates.

Structure	ZPE	ΔE	ΔH	ΔG	E	H	G	IF(cm^{-1})	Infrared
**Int1(Me)**	0.11449	0.12071	0.12165	0.08323	-270.78244	-270.78149	-270.81991		
**TS2(Me)**	0.22503	0.23797	0.23891	0.18076	-520.16446	-520.16351	-520.22166	-245.5	38.4
**Int2(Me)**	0.22703	0.23954	0.24048	0.18579	-520.20892	-520.20798	-520.26267		
**N-Me-pyrrole**	0.11012	0.11571	0.11665	0.08105	-249.38960	-249.38866	-249.42426		
**Int1(diMe)**	0.14166	0.14982	0.15076	0.10585	-310.07536	-310.07442	-310.11934		
**TS2(diMe)**	0.25213	0.26699	0.26793	0.20429	-559.45676	-559.45581	-559.51946	-253.7	37.5
**Int2(diMe)**	0.25420	0.26859	0.26954	0.21005	-559.50059	-559.49965	-559.55913		

**Int1(H)**	0.08720	0.09160	0.09254	0.09254	-231.49115	-231.49021	-231.52361		
**TS2(H)**	0.19785	0.20886	0.20981	0.15794	-480.87332	-480.87238	-480.92425	-245.5	39.4
**Int2(H)**	0.19991	0.21048	0.21142	0.16210	-480.91716	-480.91622	-480.96555		
**Int1(OMe)**	0.11977	0.12666	0.12761	0.08806	-345.98524	-345.98430	-346.02385		
**TS2(OMe)**	0.23024	0.24388	0.24482	0.18638	-595.36782	-595.36688	-595.42532	-235.1	38.9
**Int2(OMe)**	0.23223	0.24545	0.24639	0.19076	-595.41282	-595.41187	-595.46750		
**Int1(Ph)**	0.16821	0.17711	0.17805	0.13296	-462.47732	-462.47638	-462.52146		
**TS2(Ph)**	0.27863	0.29433	0.29527	0.23068	-711.86003	-711.85909	-711.92368	-233.6	43.6
**Int2(Ph)**	0.28064	0.29592	0.29687	0.23564	-711.90411	-711.90317	-711.96440		

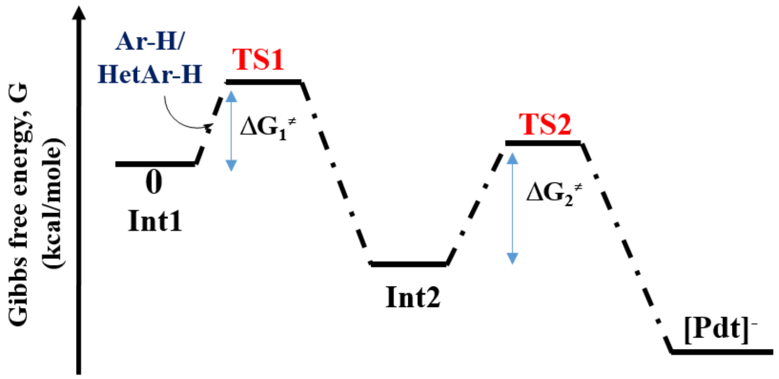

Figure S103: Energy profile diagram for full reaction of direct C-H arylation of arenes/heteroarenes.

Table S8: Activation energy barriers for different transition states for different arenes and heteroareness.

**Arenes/ HetAr-H**	$\Delta G_1^{\neq}$ (kcal/mole)	$\Delta G_2^{\neq}$ (kcal/mole)
N-Methylpyrrole	13.1	17.9
Furan	12.8	9.1
Thiophene	14.0	9.2
Benzofuran	13.0	7.5
Benzene	16.9	10.4
Xylene	15.7	12.5

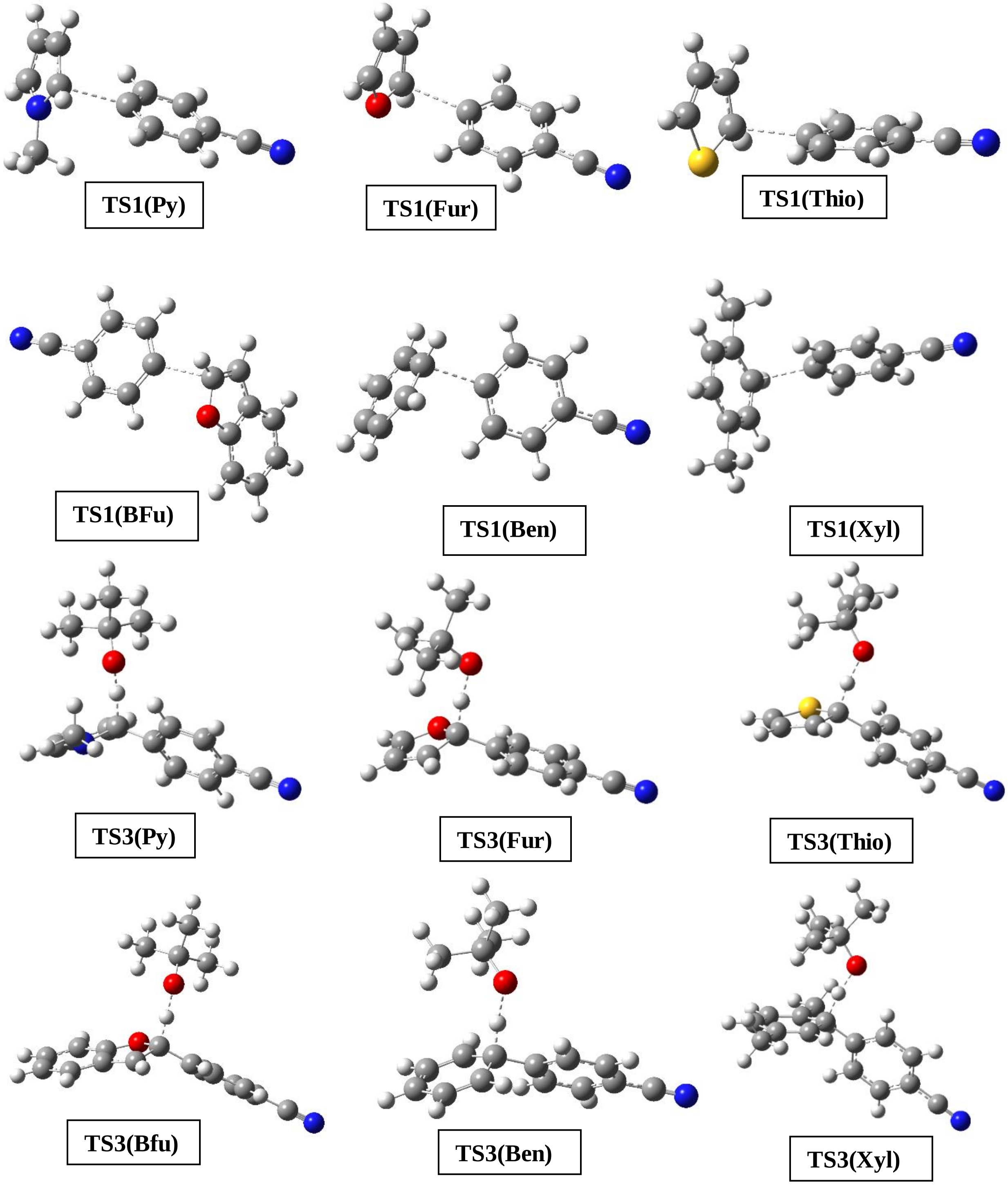

Figure S104: Optimized structures of all the transition states (corresponding to **TS1** and **TS2**) for various arenes and heteroarenes with 4-cyanophenyl radical.

Table S9: Energies, enthalpies, and free energies (in Hartree) of the optimized structures of all transition states and intermediates with theoretical method b3lyp/6-31+g(d); (CPCM; n, n-dimethylformamide).

Structure	ZPE	ΔE	ΔH	ΔG	E	H	G	IF(cm^{-1})	Infrared
**$1a^{2-}$**	0.15474	0.16775	0.16870	0.11429	-1250.08055	-1250.07961	-1250.13402		
**$1a^{-}$**	0.17093	0.17187	0.11715	0.18076	-1250.02407	-1250.02313	-1250.07785		
**Int1(CN)**	0.08596	0.09208	0.09302	0.05505	-323.73923	-323.73828	-323.77626		
**N-Me-pyrrole**	0.11012	0.11571	0.11665	0.08105	-249.38960	-249.38866	-249.42426		
**$^{-}O^{t}Bu$**	0.12043	0.12664	0.12759	0.09194	-233.0638	-233.06292	-233.09856		
**$HO^{t}Bu$**	0.13534	0.14192	0.14287	0.10651	-233.56515	-233.56421	-233.60057		
**TS2(N-py)**	0.19673	0.20864	0.20959	0.15466	-573.12508	-573.12413	-573.17906	-173.6	50.0
**Int2(N-py)**	0.19856	0.21006	0.21100	0.16002	-573.16802	-573.16708	-573.21806		
**TS3(N-py)**	0.31615	0.33608	0.33702	0.26548	-806.21766	-806.21671	-806.28825	-1325	49402.7
**Pdt^{-1}(N-py)**	0.18548	0.19706	0.19800	0.14649	-572.70526	-572.70432	-572.75583		
**Furan**	0.06982	0.07355	0.07449	0.04353	-229.96801	-229.96707	-229.99802		
**TS2(Fur)**	0.15609	0.16709	0.16803	0.11546	-553.70232	-553.70138	-553.75394	-209.8	49.6
**Int2(Fur)**	0.15836	0.16899	0.16994	0.11971	-553.75668	-553.75574	-553.80597		
**TS3(Fur)**	0.27589	0.29416	0.29510	0.22694	-786.82287	-786.82192	-786.89009	-762.0	12148.2
**Pdt^{-1}(Fur)**	0.14549	0.15593	0.15687	0.10808	-553.30276	-553.30182	-553.3506		
**Thiophene**	0.06654	0.07063	0.07158	0.03930	-552.95046	-552.94951	-552.98180		
**TS2(Thio)**	0.15277	0.16419	0.16513	0.11110	-876.68266	-876.68172	-876.73575	-255.6	28.0
**Int2(Thio)**	0.15547	0.16656	0.16751	0.11639	-876.73912	-876.73817	-876.78929		
**TS3(Thio)**	0.27360	0.29223	0.29318	0.22404	-1109.80517	-1109.80423	-1109.87337	-439.5	5664.9

**Pdt^{-1}(Thi)**	0.14253	0.15345	0.15439	0.10430	-876.28343	-876.28249	-876.33259		
**Benzene**	0.10036	0.10477	0.10571	0.07289	-232.16641	-232.16547	-232.19829		
**TS2(Ben)**	0.18649	0.19814	0.19908	0.14543	-555.89499	-555.89405	-555.94770	-314.4	7.3
**Int2(Ben)**	0.18839	0.19980	0.20075	0.14844	-555.93330	-555.9323	-555.98467		
**TS3(Ben)**	0.30827	0.32682	0.32776	0.25945	-788.99937	-788.99843	-789.06675	-124.7	498.4
**Pdt^{-1}(Ben)**	0.17630	0.18746	0.18840	0.13747	-555.49285	-555.49190	-555.54284		
**Xylene**	0.15465	0.16192	0.16287	0.12210	-310.75063	-310.74968	-310.79045		
**TS2(Xyl)**	0.24099	0.25631	0.25725	0.19432	-634.47984	-634.47890	-634.54183	-286.1	13.0
**Int2(Xyl)**	0.24341	0.25817	0.25911	0.19984	-634.51878	-634.51784	-634.57711		
**TS3(Xyl)**	0.36120	0.38355	0.38449	0.30807	-867.58033	-867.57939	-867.65581	-993.1	9473.3
**Pdt^{-1}(Xyl)**	0.23117	0.24562	0.24657	0.18866	-634.06698	-634.06604	-634.12395		
**BenzoFur**	0.11707	0.12306	0.12401	0.08692	-383.57876	-383.57782	-383.61491		
**TS2(BFu)**	0.20314	0.21656	0.21750	0.15902	-707.31307	-707.31212	-707.37061	-201.7	38.1
**Int2(BFu)**	0.20567	0.21871	0.21965	0.16343	-707.37073	-707.36979	-707.42601		
**TS3(BFu)**	0.32391	0.34447	0.34542	0.27163	-940.43980	-940.43885	-940.51264	-317.4	3358.9
**Pdt^{-1}(BFu)**	0.19263	0.20557	0.20651	0.15176	-706.92026	-706.91931	-706.97406		

Table S10: Energies, enthalpies, and free energies (in Hartree) of the optimized structures of all transition states and intermediates with theoretical method wB97XD/6-31+g(d); (CPCM; n, n-dimethylformamide).

Structure	ZPE	ΔE	ΔH	ΔG	E	H	G	IF(cm^{-1})	Infrared
**$1a^{2-}$**	0.15703	0.16987	0.17081	0.11658	-1249.84104	-1249.84010	-1249.89433		
**$1a^{-}$**	0.16043	0.17286	0.17380	0.11940	-1249.78693	-1249.78599	-1249.84039		

**Sub**	0.09074	0.09796	0.09890	0.05829	-783.87263	-783.87168	-783.91229		
**Sub^{-}**	0.08730	0.09556	0.09651	0.05329	-783.92734	-783.92640	-783.96962		
**Int1(CN)**	0.08713	0.09316	0.09411	0.05629	-323.60734	-323.60639	-323.64421		
**N-Me-pyrrole**	0.11155	0.11627	0.11722	0.08363	-249.29259	-249.29164	-249.32523		
**$^{-}O^{t}Bu$**	0.12311	0.12924	0.13019	0.09461	-232.97528	-232.97433	-233.00991		
**$HO^{t}Bu$**	0.13716	0.14373	0.14467	0.10832	-233.47407	-233.47313	-233.50948		
**TS2(CN)**	0.19929	0.21194	0.21288	0.15658	-572.90122	-572.90028	-572.95658	-253.2	114.3
**Int2(CN)**	0.20140	0.21365	0.21460	0.16109	-572.94984	-572.94889	-573.00239		
**TS3(CN)**	0.32145	0.34086	0.34180	0.27297	-805.92210	-805.92116	-805.99000	-1430	14429.4
**Pdt^{-1}(CN)**	0.18806	0.19874	0.19968	0.15022	-572.48650	-572.48556	-572.53502		

Table S11: Energies, enthalpies, and free energies (in Hartree) of the optimized structures of different molecules (Opt) and their monoreduced moieties' single point (SP) calculation at the *w*B97XD theory level with 6-31+g(d) basis set considering CPCM (n,n-dimethylformamide) solvent model.

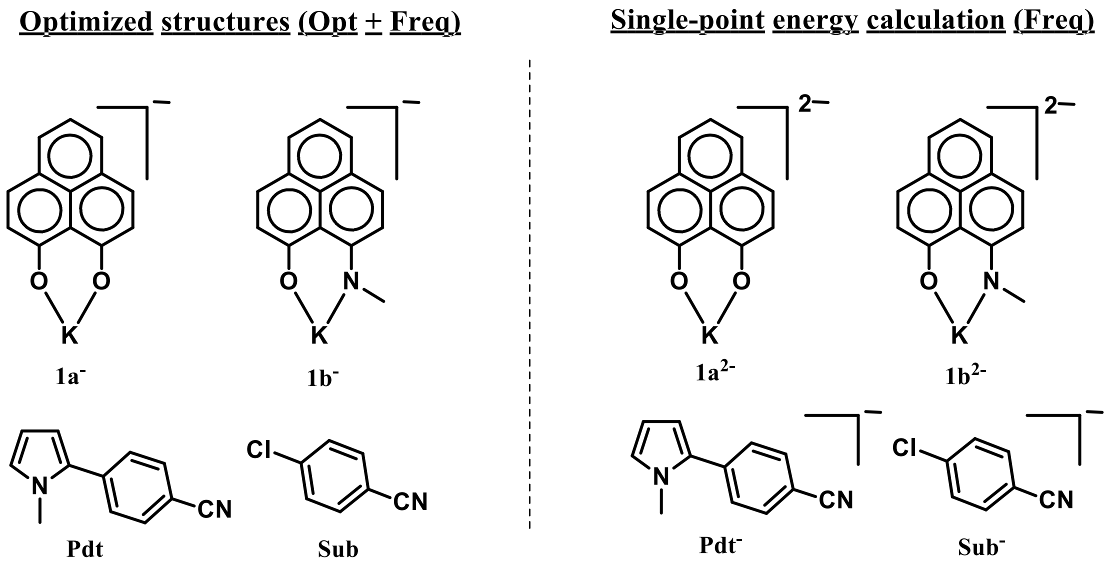

Structure	ZPE	ΔE	ΔH	ΔG	E	H	G	IF(cm^{-1})	Infrared
**$1a^-$(O+F)**	0.16043	0.17286	0.17380	0.11940	- 1249.78693	- 1249.78599	- 1249.84039		
**$1b^-$(O+F)**	0.20080	0.21473	0.21568	0.15741	- 1269.13923	- 1269.13828	- 1269.19655		
**Pdt(O+F)**	0.19292	0.20447	0.20541	0.15478	-572.40819	-572.40724	-572.45787		
**Sub(O+F)**	0.09074	0.09796	0.09890	0.05829	-783.87263	-783.87168	-783.91229		
**$1a^{2-}$(SP)**	0.15708	0.16861	0.16955	0.11892	- 1249.83720	- 1249.83626	- 1249.88689		
**$1b^{2-}$(SP)**	0.19716	0.21023	0.21118	0.15701	- 1269.18550	- 1269.18455	- 1269.23872		
**Pdt^-(SP)**	0.18837	0.19849	0.19943	0.15158	-572.48337	-572.48243	-572.53029		
**Sub^-(SP)**	0.08626	0.09414	0.09508	0.05222	-783.92151	-783.92057	-783.96343		

Table S12: Energies, enthalpies, and free energies (in Hartree) of the optimized structures of different monoreduced molecules (Opt) and their neutral moieties' single point (SP) calculation at the *w*B97XD theory level with 6-31+g(d) basis set considering CPCM (n,n-dimethylformamide) solvent model.

Optimized structures (Opt + Freq) **Single-point energy calculation (Freq)**

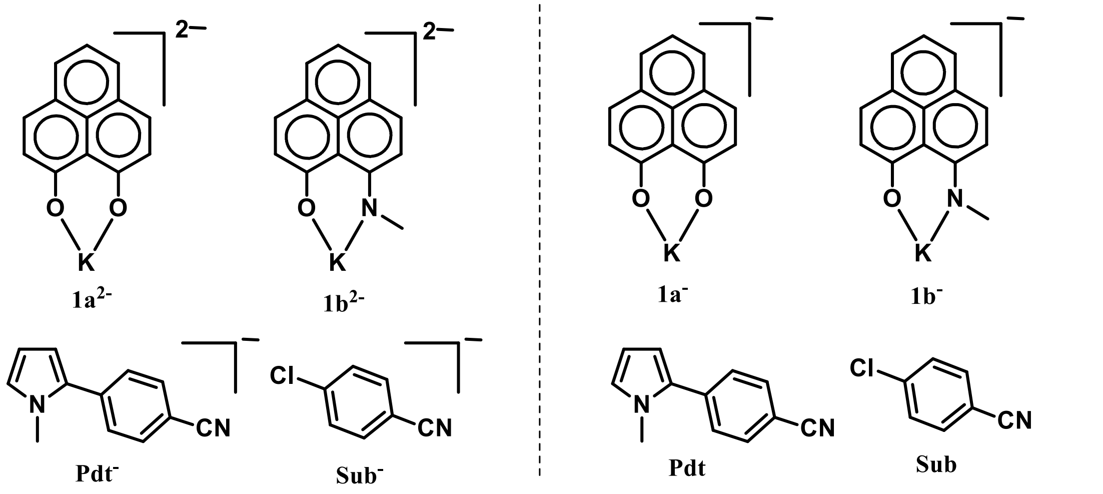

Structure	ZPE	ΔE	ΔH	ΔG	E	H	G	IF(cm^{-1})	Infrared
$1a^{2-}$(O+F)	0.15703	0.16987	0.17081	0.11658	-1249.84104	-1249.84010	-1249.89433		
$1b^{2-}$(O+F)	0.19700	0.21125	0.21219	0.15503	-1269.18957	-1269.18863	-1269.24579		
Pdt^{-} (O+F)	0.18891	0.20089	0.20183	0.14961	-572.48798	-572.48703	-572.53926		
Sub^{-} (O+F)	0.08730	0.09556	0.09651	0.05329	-783.92734	-783.92640	-783.96962		
$1a^{-}$(SP)	0.15975	0.17191	0.17285	0.11991	-1249.78295	-1249.78200	-1249.83495		
$1b^{-}$(SP)	0.19992	0.21351	0.21445	0.15858	-1269.13548	-1269.13454	-1269.19040		
Pdt(SP)	0.19214	0.20334	0.20429	0.1550	-572.40230	-572.40136	-572.45062		
Sub(SP)	0.08945	0.09656	0.09751	0.05723	-783.86648	-783.86554	-783.90581		

Table S13: Electron transfer energy barriers for substrate activation process.

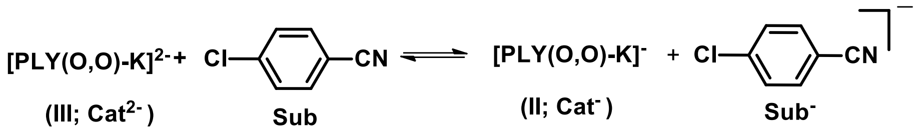

Computational method	Reorganization Energy (eV)	Driving force ΔG^0 (kcal/mol)	Activation energy barrier $\Delta G^{\neq}$ (kcal/mol)
Basis set: 6-31+g(d) Solvation: CPCM	$\lambda 1 = 0.3165$	-2.14	$\Delta G^{\neq}_{\lambda 1} = 3.82$
	$\lambda 2 = 0.3787$	-2.14	$\Delta G^{\neq}_{\lambda 2} = 5.22$
Basis set: 6-31+g(d) Solvation: SMD	$\lambda 1 = 0.3175$	1.03	$\Delta G^{\neq}_{\lambda 1} = 2.38$
	$\lambda 2 = 0.3550$	1.03	$\Delta G^{\neq}_{\lambda 2} = 2.59$
Basis set:def2tzvpp Solvation: CPCM	$\lambda 1 = 0.3027$	- 2.49	$\Delta G^{\neq}_{\lambda 1} = 0.72$
	$\lambda 2 = 0.3878$	- 2.49	$\Delta G^{\neq}_{\lambda 2} = 1.16$
Basis set: def2tzvpp Solvation: SMD	$\lambda 1 = 0.2892$	1.39	$\Delta G^{\neq}_{\lambda 1} = 2.43$
	$\lambda 2 = 0.2545$	1.39	$\Delta G^{\neq}_{\lambda 2} = 2.43$

Table S14: Electron transfer energy barriers for catalyst regeneration process.

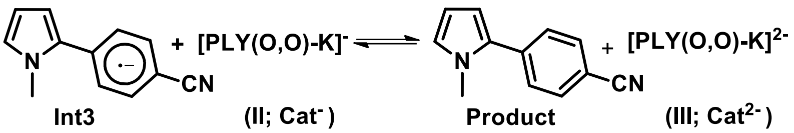

Computational method	Reorganization Energy (eV)	Driving force ΔG^0 (kcal/mol)	Activation energy barrier $\Delta G^{\neq}$ (kcal/mol)
Basis set: 6-31+g(d) Solvation: CPCM	$\lambda 1$ = 0.3998	17.3	$\Delta G^{\neq}_{\lambda 1}$ = 18.9
	$\lambda 2$ = 0.3924	17.3	$\Delta G^{\neq}_{\lambda 2}$ = 19.0
Basis set: 6-31+g(d) Solvation: SMD	$\lambda 1$ = 0.3768	14.7	$\Delta G^{\neq}_{\lambda 1}$ = 15.7
	$\lambda 2$ = 0.4057	14.7	$\Delta G^{\neq}_{\lambda 2}$ = 15.5
Basis set:def2tzvpp Solvation: CPCM	$\lambda 1$ = 0.4189	17.5	$\Delta G^{\neq}_{\lambda 1}$ = 19.1
	$\lambda 2$ = 0.4131	17.5	$\Delta G^{\neq}_{\lambda 2}$ = 19.2
Basis set: def2tzvpp Solvation: SMD	$\lambda 1$ = 0.3914	14.2	$\Delta G^{\neq}_{\lambda 1}$ = 14.9
	$\lambda 2$ = 0.3965	14.2	$\Delta G^{\neq}_{\lambda 2}$ = 14.9

Table S15: Electron transfer energy barriers for radical chain propagation process**.**

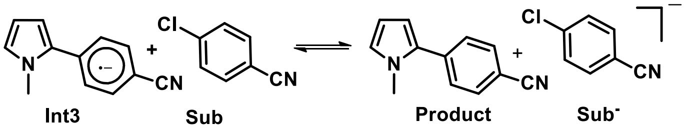

Computational method	Reorganization Energy (eV)	Driving force ΔG^0 (kcal/mol)	Activation energy barrier $\Delta G^{\neq}$ (kcal/mol)
Basis set: 6-31+g(d) Solvation: CPCM	$\lambda 1$ = 0.3655	15.1	$\Delta G^{\neq}_{\lambda 1}$ = 16.4
	$\lambda 2$ = 0.4201	15.1	$\Delta G^{\neq}_{\lambda 2}$ = 15.8
Basis set: 6-31+g(d) Solvation: SMD	$\lambda 1$ = 0.3590	15.7	$\Delta G^{\neq}_{\lambda 1}$ = 17.4
	$\lambda 2$ = 0.4254	15.7	$\Delta G^{\neq}_{\lambda 2}$ = 16.6
Basis set:def2tzvpp Solvation: CPCM	$\lambda 1$ = 0.3567	15.0	$\Delta G^{\neq}_{\lambda 1}$ = 16.4
	$\lambda 2$ = 0.4361	15.0	$\Delta G^{\neq}_{\lambda 2}$ = 15.6
Basis set: def2tzvpp Solvation: SMD	$\lambda 1$ = 0.3396	15.6	$\Delta G^{\neq}_{\lambda 1}$ = 17.5
	$\lambda 2$ = 0.4307	15.6	$\Delta G^{\neq}_{\lambda 2}$ = 16.4

Table S16: Energies, enthalpies, and free energies (in Hartree) of the optimized structures of different monoreduced molecules (Opt) and their neutral moieties' single point (SP) calculation at the *w*B97XD theory level with 6-31+g(d) basis set considering SMD (n,n-dimethylformamide) solvent model.

Structure	ZPE	ΔE	ΔH	ΔG	E	H	G	IF(cm^{-1})	Infrared
**$1a^{2-}$(O+F)**	0.15717	0.16994	0.17088	0.11707	-1249.8315	-1249.8305	-1249.8843		
**$1a^{2-}$(SP)**	0.15684	0.16842	0.16937	0.11883	-1249.8280	-1249.8271	-1249.8776		
**$1a^{1-}$(O+F)**	0.16046	0.17285	0.17379	0.11988	-1249.7748	-1249.7739	-1249.8278		
**$1a^{1-}$(SP)**	0.15993	0.17201	0.17296	0.12031	-1249.7705	-1249.7695	-1249.8222		
**Pdt^{-} (O+F)**	0.18900	0.20093	0.20187	0.14982	-572.4955	-572.4945	-572.5466		
**Pdt^{-}(SP)**	0.18868	0.19867	0.19961	0.15216	-572.4908	-572.4898	-572.5373		
**Pdt(O+F)**	0.19299	0.20446	0.20541	0.15496	-572.4171	-572.4162	-572.4666		
**Pdt(SP)**	0.19213	0.20329	0.20424	0.15510	-572.4113	-572.4103	-572.4595		
**Sub^{-} (O+F)**	0.08725	0.09548	0.09643	0.05326	-783.9302	-783.9293	-783.9725		
**Sub^{-}(SP)**	0.08629	0.09416	0.09510	0.05220	-783.9244	-783.9235	-783.9664		
**Sub(O+F)**	0.09072	0.09793	0.09887	0.05826	-783.8779	-783.8769	-783.9176		
**Sub(SP)**	0.08944	0.09656	0.09751	0.05719	-783.8719	-783.8709	-783.9112		

Table S17: Energies, enthalpies, and free energies (in Hartree) of the optimized structures of different monoreduced molecules (Opt) and their neutral moieties' single point (SP) calculation at the *w*B97XD theory level with def2tzvpp basis set considering CPCM (n,n-dimethylformamide) solvent model.

Structure	ZPE	ΔE	ΔH	ΔG	E	H	G	IF(cm^{-1})	Infrared

**1a^{2-}(O+F)**	0.15699	0.16962	0.17056	0.11714	-1250.0848	-1250.0839	-1250.1373		
**1a^{2-}(SP)**	0.15696	0.16829	0.16923	0.11928	-1250.0808	-1250.0798	-1250.1298		
**1a^{1-}(O+F)**	0.16025	0.17260	0.17354	0.11934	-1250.0316	-1250.0307	-1250.0849		
**1a^{1-}(SP)**	0.15969	0.17171	0.17265	0.12004	-1250.0273	-1250.0264	-1250.0790		
**Pdt^{-}(O+F)**	0.18852	0.20036	0.20130	0.14946	-572.6757	-572.6748	-572.7266		
**Pdt^{-}(SP)**	0.18805	0.19799	0.19893	0.15164	-572.6710	-572.6700	-572.7173		
**Pdt(O+F)**	0.19242	0.20391	0.20486	0.15429	-572.5966	-572.5956	-572.6462		
**Pdt(SP)**	0.19193	0.20297	0.20391	0.15509	-572.5905	-572.5895	-572.6383		
**Sub^{-}(O+F)**	0.08884	0.09688	0.09782	0.05513	-784.0640	-784.0630	-784.1057		
**Sub^{-}(SP)**	0.08666	0.09436	0.09530	0.05282	-784.0589	-784.0580	-784.1005		
**Sub(O+F)**	0.09060	0.09778	0.09872	0.05820	-784.0097	-784.0087	-784.0493		
**Sub(SP)**	0.08931	0.09638	0.09733	0.05712	-784.0033	-784.0023	-784.0425		

Table S18: Energies, enthalpies, and free energies (in Hartree) of the optimized structures of different monoreduced molecules (Opt) and their neutral moieties' single point (SP) calculation at the *w*B97XD theory level with def2tzvpp basis set considering SMD (n,n-dimethylformamide) solvent model.

**Structure**	**ZPE**	**ΔE**	**ΔH**	**ΔG**	**E**	**H**	**G**	**IF(cm^{-1})**	**Infrared**
**1a^{2-}(O+F)**	0.15689	0.16954	0.17048	0.11719	-1250.0778	-1250.0769	-1250.1302		
**1a^{2-}(SP)**	0.15686	0.16820	0.16914	0.11932	-1250.0741	-1250.0731	-1250.1229		
**1a^{1-}(O+F)**	0.16036	0.17264	0.17359	0.11985	-1250.0206	-1250.0197	-1250.0734		

**1a^{1-}(SP)**	0.15974	0.17174	0.17269	0.12025	-1250.0166	-1250.0157	-1250.0681		
**Pdt^{-} (O+F)**	0.18862	0.20041	0.20136	0.14964	-572.6829	-572.6820	-572.7337		
**Pdt^{-}(SP)**	0.18815	0.19805	0.19899	0.15181	-572.6782	-572.6773	-572.7245		
**Pdt(O+F)**	0.19243	0.20386	0.20481	0.15446	-572.6050	-572.6040	-572.6544		
**Pdt(SP)**	0.19156	0.20268	0.20363	0.15459	-572.5991	-572.5982	-572.6472		
**Sub^{-} (O+F)**	0.08858	0.09659	0.09753	0.05487	-784.0670	-784.0660	-784.1087		
**Sub^{-}(SP)**	0.08669	0.09438	0.09532	0.05281	-784.0618	-784.0609	-784.1034		
**Sub(O+F)**	0.09059	0.09776	0.09871	0.05819	-784.0146	-784.0137	-784.0542		
**Sub(SP)**	0.08933	0.09641	0.09736	0.05713	-784.0083	-784.0074	-784.0476		

25. Coordinates of all DFT optimized structures:

PLY(O,O)-K (1a)

0 1

C	-3.42480600	-1.20319500	-0.00027000
C	-4.13187500	0.00000800	-0.00012600
C	-1.27383800	0.00000000	0.00001800
C	-2.01929800	-1.22413200	-0.00023200
H	-5.21816500	0.00001100	-0.00018000
C	0.06188400	2.48825800	0.00007700
C	-1.29737700	-2.46504500	-0.00030600
C	0.06187100	-2.48826400	0.00007700
H	0.60728200	3.42901000	0.00002400
H	0.60726400	-3.42901900	0.00016600
C	0.87443800	1.27432200	-0.00009300
C	0.16365900	-0.00000400	0.00009800
C	0.87443300	-1.27433300	0.00036700
C	-2.01929200	1.22413700	0.00019100
C	-1.29736400	2.46504600	0.00032300
C	-3.42480100	1.20320900	0.00009000
H	-1.86410600	3.39477000	0.00049900
H	-3.96141100	2.14982600	0.00021000
H	-3.96142200	-2.14981000	-0.00044800
H	-1.86412400	-3.39476600	-0.00055200

O	2.13565600	-1.40001400	0.00089500
O	2.13566200	1.39999500	-0.00053400
K	4.34728100	0.00000400	-0.00020500

[PLY(O,O)-K]$^{-1}$

-1 2

C	-3.44984600	-1.20842800	-0.00016700
C	-4.14717500	0.00000900	-0.00022300
C	-1.27175800	0.00000100	0.00006800
C	-2.02263400	-1.23409700	-0.00005100
H	-5.23622700	0.00001300	-0.00034600
C	0.08184600	2.47014200	0.00007700
C	-1.30348000	-2.45397500	-0.00000400
C	0.08183100	-2.47014800	0.00018500
H	0.61776100	3.41893600	-0.00000100
H	0.61774000	-3.41894500	0.00025400
C	0.88530300	1.28677900	0.00027800
C	0.17508400	-0.00000400	0.00021900
C	0.88529700	-1.28678900	0.00027200
C	-2.02262600	1.23410300	0.00003000
C	-1.30346500	2.45397700	0.00002800
C	-3.44983800	1.20844200	-0.00014900
H	-1.86016900	3.39153500	-0.00007600

H	-3.98700300	2.15577400	-0.00021400
H	-3.98701700	-2.15575600	-0.00024700
H	-1.86018900	-3.39152900	-0.00008300
O	2.17658500	-1.40909300	0.00016900
O	2.17659200	1.40906800	0.00026900
K	4.31781300	0.00000500	-0.00032500

[PLY(O,O)-K]$^{-2}$

-2 1

C	-3.48037300	-1.21647000	0.00122100
C	-4.16408300	0.00000500	-0.00003300
C	-1.27539300	0.00000000	0.00000300
C	-2.03129300	-1.24558000	0.00020000
H	-5.25687000	0.00000600	-0.00004600
C	0.09875400	2.45732200	0.00027000
C	-1.30963700	-2.44849300	-0.00028400
C	0.09874600	-2.45732400	-0.00022500
H	0.62800800	3.41285300	0.00050200
H	0.62799700	-3.41285700	-0.00044400
C	0.89504100	1.29504200	0.00011800
C	0.18346100	-0.00000200	0.00002300
C	0.89503800	-1.29504600	-0.00005400
C	-2.03128900	1.24558400	-0.00021200

C	-1.30963100	2.44849400	0.00029100
C	-3.48036900	1.21647700	-0.00126900
H	-1.85489900	3.39504400	0.00020000
H	-4.02025100	2.16385300	-0.00120400
H	-4.02025700	-2.16384500	0.00114600
H	-1.85490900	-3.39504100	-0.00020600
O	2.21542000	-1.41608400	-0.00015200
O	2.21542300	1.41606700	0.00025900
K	4.30371600	0.00000400	-0.00005700

PLY(O,O)-K_3[18-crown-6]$_3$ (3)

0 1

K	3.03158900	-1.67518800	0.00127100
K	2.19977200	2.29567700	-0.04062900
O	1.69575700	-0.00752800	1.42550400
O	2.47790400	-3.36118800	2.53058300
O	1.18038800	3.67343200	-2.34063900
O	2.45026000	-3.41515100	-2.46114400
O	4.84925600	-1.85236000	-2.43446700
O	6.34138100	-2.21195500	-0.01236400
O	0.22447300	4.83794500	0.04203200
O	1.58210300	-0.06378300	-1.41044300
O	1.06967000	-3.86635000	0.05247000

O	4.92213900	-1.87418000	2.44728300
O	1.40939600	3.70453700	2.33499000
O	4.08442200	2.78032400	2.31858400
O	5.25110900	1.86570400	-0.18738800
O	3.84520200	2.76097100	-2.57578100
C	-1.66211300	1.06390800	0.11973000
C	0.30878200	0.27751000	-1.24507900
C	-2.39324800	1.36860900	-1.10199000
C	-0.32075400	0.51658900	2.52974700
H	0.20982400	0.33524500	3.46540400
C	-0.30971200	0.52873600	0.07403700
C	1.06930600	-3.70194800	-2.31902300
H	0.68792100	-4.19849900	-3.22873900
H	0.49864800	-2.77637400	-2.15985600
C	-2.31190300	1.35851800	1.38633100
C	-1.62712500	1.02809100	2.56398900
H	-2.09432100	1.24919900	3.52591800
C	0.40651800	0.30770300	1.34524700
C	0.88610100	-4.62505300	-1.12976100
H	-0.12728400	-5.05953000	-1.15236500
H	1.61221100	-5.45235600	-1.17949300
C	-0.60736500	4.65782000	-1.09702200
H	-1.37506500	5.44998700	-1.14062600

H	-1.11967000	3.68820300	-1.05540100
C	-0.50071600	0.48113400	-2.37578900
H	-0.04440000	0.27321800	-3.34465300
C	4.95415800	1.88492900	-2.55684800
H	5.52157100	1.96365100	-3.50193900
H	4.63442400	0.83970200	-2.43609700
C	1.09122100	-3.63136700	2.41693900
H	0.52726300	-2.70435600	2.23805800
H	0.71622200	-4.09705400	3.34552700
C	1.80089900	3.45639400	-3.59022500
H	2.31133800	4.37315700	-3.93208400
H	1.05067900	3.17693300	-4.34827100
C	0.88579100	-4.58776800	1.25832200
H	1.60262100	-5.42164700	1.32698200
H	-0.13297900	-5.00773100	1.30071400
C	6.26523600	-2.31185900	2.38518100
H	6.30781600	-3.41149500	2.33529200
H	6.82496900	-1.99185400	3.28073200
C	0.46861200	4.76017900	2.42108300
H	0.98952200	5.73238700	2.42039600
H	-0.11148400	4.68067300	3.35461100
C	2.70068900	-2.36162200	-3.39255100
H	2.21928100	-2.58795500	-4.35984300

C	-0.49350800	4.66486800	1.25704500
H	-1.26026700	5.45167800	1.36521700
C	2.80426000	2.33029300	-3.45683200
H	2.32743800	1.42437200	-3.05092300
H	3.22153100	2.11518300	-4.45578900
C	2.76612000	-2.29313300	3.43416700
H	2.28501200	-2.48044900	4.40974400
C	-1.80019300	1.01641200	-2.32277100
H	-2.32673800	1.24815200	-3.25079000
C	0.23910000	4.73241700	-2.34904500
H	-0.42889300	4.64358300	-3.22084100
H	0.76169700	5.70170500	-2.41210200
C	6.86874700	-1.59423100	-1.17282900
H	7.95901700	-1.75636900	-1.23408000
H	6.68579500	-0.51022000	-1.13595300
C	2.15436200	3.50856000	3.51832900
H	1.48478800	3.24932500	4.35515500
H	2.70152100	4.42838500	3.78746200
C	5.97277700	2.26373100	0.96755700
H	6.14587100	3.35115200	0.95773600
H	6.95671200	1.76416900	0.99369000
C	4.26393400	-2.27625500	3.64663300
H	4.52101000	-1.58485200	4.46531200

H	4.59689100	-3.28575900	3.93170600
C	-3.60712300	2.00958300	1.40218000
H	-4.01208200	2.33071500	2.36101800
C	4.19539700	-2.30114600	-3.61861200
H	4.56057400	-3.30467300	-3.88568800
H	4.42133500	-1.61896000	-4.45429500
C	3.13158800	2.37212700	3.30447000
H	3.64412400	2.16741000	4.26030000
H	2.60843100	1.46604000	2.95997100
C	5.17589800	1.89011800	2.20202500
H	4.83273000	0.84818800	2.12461400
H	5.83083300	1.96776600	3.08896300
C	6.22434900	-2.18070200	-2.41034800
H	6.74025000	-1.77135600	-3.29605900
H	6.35671100	-3.27426600	-2.41829500
C	-4.20699700	2.40886700	0.20777500
H	-5.13460800	2.98595400	0.24337700
C	-3.66370600	2.06411700	-1.02880200
H	-4.12724700	2.39255400	-1.95809000
C	6.93845100	-1.71175600	1.17011000
H	6.84966500	-0.61545600	1.20653800
H	8.01265100	-1.96518800	1.19549100
C	5.85671600	2.27624600	-1.40356100

H	6.84200100	1.79314000	-1.52187100
H	6.01090200	3.36650400	-1.40805500
H	2.29957800	-1.41324900	-2.99698900
H	2.39353300	-1.34246800	3.01669000
H	-0.99855600	3.69038100	1.27513400
K	-4.94294900	-0.47473000	-0.00290400
O	-5.96480600	-0.67255800	-2.77126300
O	-7.14433100	0.10811400	1.89552800
O	-5.07840400	-1.67362800	2.75669500
O	-3.90431000	-2.46435200	-1.95564600
O	-7.87944900	0.17602600	-0.84398200
O	-3.21598800	-2.48163900	0.78670600
C	-7.08347600	-0.45632300	3.19498000
H	-7.55823600	0.21934700	3.92578300
C	-3.95939800	-1.87901900	-3.24942800
H	-3.49986900	-2.55318000	-3.99297800
H	-3.40885200	-0.92733700	-3.25733300
C	-7.77853700	0.73932600	-2.14293500
H	-7.07254500	1.58383800	-2.14164500
H	-8.76249600	1.10677600	-2.47927900
C	-5.63161300	-0.65634400	3.57566000
H	-5.08166200	0.28662400	3.43994100
H	-5.57808800	-0.94629800	4.63891500

C	-8.38958100	1.08561600	0.11681100
H	-9.40066100	1.42077600	-0.16967000
H	-7.74027400	1.97140000	0.19245900
C	-8.45928000	0.39055700	1.46061800
H	-8.97047000	1.05365300	2.17872400
H	-9.04855900	-0.53691300	1.37372200
C	-2.56162500	-2.64328800	-1.50954000
H	-2.03469000	-1.68103100	-1.45847200
H	-2.01943400	-3.29353700	-2.21733500
C	-5.40775100	-1.65781600	-3.62655900
H	-5.45771100	-1.32403600	-4.67633900
H	-5.97267800	-2.60028100	-3.53944300
C	-2.57445100	-3.32040200	-0.15703900
H	-3.09660900	-4.29059200	-0.21805000
H	-1.53145400	-3.49534500	0.14256500
C	-7.29511500	-0.32851400	-3.10148600
H	-7.95290800	-1.21086400	-3.03836600
H	-7.34878600	0.06355800	-4.13116900
C	-3.19203800	-2.99015100	2.10767000
H	-3.80815600	-3.90273700	2.17686000
C	-3.70615600	-1.92728300	3.05338200
H	-3.60582900	-2.29200700	4.08988700
H	-3.10472100	-1.01300100	2.93978200

H	-7.61730200	-1.41997600	3.22109800
H	-2.16170600	-3.24579800	2.40104100

PLY-K_2[18-crown-6]$_2$ (2)

0 2

K	-1.96080100	1.06325400	-0.00068400
K	0.98348200	-1.96285600	0.00082700
O	0.09078700	0.16983900	1.41750400
O	-2.16800900	2.82229000	2.46063800
O	2.55270600	-2.30616300	-2.36356100
O	-2.16908100	2.81718000	-2.46530400
O	-3.57129900	0.28599400	-2.44467400
O	-4.91755800	-0.29676300	0.00121900
O	4.07786500	-2.30797900	0.00325300
O	0.09130600	0.16898000	-1.41776100
O	-1.18274100	3.84400700	-0.00360300
O	-3.57251600	0.29248300	2.44593500
O	2.54886300	-2.30617600	2.36766300
O	-0.13215700	-3.31157100	2.46201000
O	-1.67156000	-3.27492700	-0.00124600
O	-0.12781900	-3.31269300	-2.46195300
C	3.02817600	1.98453100	-0.00015600
C	1.19332900	0.86143600	-1.28912200

C	3.66753800	2.37675300	-1.23074900
C	1.87778400	1.28086100	2.46692800
H	1.42493800	0.99433900	3.41492900
C	1.79430700	1.22734900	-0.00015200
C	-1.05115600	3.69535900	-2.37840200
H	-0.97711300	4.30399400	-3.29574700
H	-0.11774300	3.12876200	-2.26238100
C	3.66724400	2.37725300	1.23043400
C	3.05610500	2.00406200	2.45073700
H	3.53104600	2.29900700	3.38608900
C	1.19296500	0.86205800	1.28882900
C	-1.24389200	4.61393400	-1.19086300
H	-0.44599900	5.37405200	-1.19692900
H	-2.21424600	5.13301800	-1.26263400
C	4.51527700	-1.65068900	-1.17890800
H	5.61711400	-1.64936900	-1.23234200
H	4.16740000	-0.60885900	-1.19477400
C	1.87840200	1.27982800	-2.46722700
H	1.42582500	0.99288400	-3.41523400
C	-1.53547700	-3.17802500	-2.38596400
H	-2.00731100	-3.58183200	-3.29930200
H	-1.82291900	-2.12040700	-2.29038400
C	-1.04954700	3.69954300	2.37138300

H	-0.11658500	3.13212000	2.25577500
H	-0.97445300	4.30979700	3.28756500
C	1.93110200	-2.75270000	-3.55201700
H	2.10781000	-3.83077400	-3.70343900
H	2.33944500	-2.21383300	-4.42307000
C	-1.24259000	4.61609100	1.18233000
H	-2.21261000	5.13582600	1.25389100
H	-0.44427800	5.37578100	1.18643300
C	-4.90474200	-0.17711100	2.39266000
H	-5.60906600	0.66824700	2.33875500
H	-5.14920000	-0.76417000	3.29465100
C	3.96284600	-2.39217100	2.38653200
H	4.28330800	-3.44680600	2.36877700
H	4.36252600	-1.92518600	3.30103800
C	-1.99616700	1.80104800	-3.44715500
H	-1.73160100	2.24988500	-4.41964900
C	4.51351100	-1.65107400	1.18627400
H	5.61526300	-1.65003000	1.24148900
C	0.44564000	-2.47155800	-3.46360900
H	0.27846900	-1.41867200	-3.19518800
H	-0.01448500	-2.68751300	-4.44296400
C	-1.99542500	1.80802000	3.44447100
H	-1.72983200	2.25859800	4.41587500

C	3.05669100	2.00306700	-2.45104700
H	3.53185300	2.29763900	-3.38640400
C	3.96674700	-2.39165100	-2.38022100
H	4.36770100	-1.92429400	-3.29397800
H	4.28753900	-3.44618300	-2.36222700
C	-5.07445300	-1.07075400	-1.17454300
H	-6.07878400	-1.52687800	-1.20438600
H	-4.33092600	-1.88271100	-1.19684500
C	1.92516300	-2.75206800	3.55526400
H	2.33234900	-2.21310900	4.42679700
H	2.10112900	-3.83017300	3.70731000
C	-2.04109700	-3.95830200	1.18584200
H	-1.61053600	-4.97189100	1.19732600
H	-3.13883300	-4.05519600	1.24423900
C	-3.31128200	1.07767700	3.60561700
H	-3.27072800	0.42823600	4.49564100
H	-4.11965700	1.81055400	3.75139100
C	4.87955900	3.11964200	1.20402700
H	5.33412900	3.40220600	2.15172300
C	-3.31138600	1.06907500	-3.60609700
H	-4.12060100	1.80080400	-3.75298400
H	-3.27059200	0.41784200	-4.49479500
C	0.43997100	-2.47027900	3.46431900

H	-0.02188900	-2.68567700	4.44297400
H	0.27372900	-1.41741100	3.19520400
C	-1.53963400	-3.17657800	2.38370500
H	-1.82664200	-2.11890700	2.28730200
H	-2.01306700	-3.57987500	3.29645100
C	-4.90265400	-0.18600900	-2.39063700
H	-5.14528200	-0.77678300	-3.29069600
H	-5.60864600	0.65817200	-2.34032700
C	5.47312600	3.48267400	-0.00016800
H	6.40046400	4.05257900	-0.00017100
C	4.87984900	3.11915300	-1.20435600
H	5.33464400	3.40133100	-2.15205900
C	-5.07720700	-1.06601400	1.17970400
H	-4.33535100	-1.87939200	1.20595700
H	-6.08245500	-1.52007000	1.21016600
C	-2.03865400	-3.95930800	-1.18854400
H	-3.13622800	-4.05677900	-1.24881500
H	-1.60754200	-4.97267300	-1.19856800
H	-1.19913600	1.10955700	-3.13598100
H	-1.19924200	1.11510700	3.13428800
H	4.16583800	-0.60917200	1.20184700

PLY(O,O)-K (1a; for top ring, NICS)

0 1

C	0.56941300	0.45180900	-0.09782000
C	0.56039700	1.63751900	-0.82348700
C	0.58647000	-0.87502400	-2.16480600
C	0.58228200	-0.79464600	-0.73771300
H	0.55046100	2.59356300	-0.31330900
Bq	0.56264000	0.42307900	-1.51333300
Bq	1.56255000	0.43652200	-1.51158500
C	0.59381600	-0.88053000	-4.98113500
C	0.59114300	-2.01330200	0.01459800
C	0.60331200	-3.21808200	-0.60150100
H	0.59719200	-0.93087900	-6.06429900
H	0.61000400	-4.14595100	-0.04046300
C	0.60351500	-2.16508200	-4.29463400
C	0.59949100	-2.13896700	-2.83922700
C	0.60845200	-3.36223500	-2.05068600
C	0.57723800	0.35550000	-2.89210500
C	0.58142900	0.30188900	-4.32332900
C	0.56440700	1.58061100	-2.21266400
H	0.57454900	1.23834800	-4.87431400
H	0.55758300	2.49741900	-2.79413200
H	0.56690000	0.47859600	0.98751300

H	0.58790800	-1.94955300	1.09923900
O	0.62038000	-4.53213200	-2.52393500
O	0.61466900	-3.20879500	-5.00394400
K	0.63542600	-5.60453700	-4.68753600

$[PLY(O,O)\text{-}K]^{-1}$ (for top ring, NICS)

-1 2

C	0.56440400	0.46132900	-0.09064400
C	0.56055100	1.64571300	-0.81948600
C	0.58651300	-0.87311200	-2.16373300
C	0.58018800	-0.79145200	-0.73109900
H	0.55066600	2.60333800	-0.30832100
Bq	0.56214000	0.42869400	-1.51019800
Bq	1.56205500	0.44131600	-1.50631200
C	0.59649300	-0.88552800	-4.97809800
C	0.58416500	-2.00830000	0.01449400
C	0.60101600	-3.21804300	-0.60749100
H	0.60124200	-0.93556000	-6.06229000
H	0.60649700	-4.14664300	-0.04564400
C	0.58297600	-2.16848400	-4.29876200
C	0.59945000	-2.13682700	-2.83821400
C	0.62885500	-3.36739900	-2.05128700
C	0.57932400	0.36257700	-2.89331400

C	0.58864800	0.30462900	-4.31922000
C	0.56949800	1.59209500	-2.20911500
H	0.59034400	1.24090400	-4.87313900
H	0.56705000	2.50940000	-2.79160600
H	0.55716700	0.48799300	0.99564100
H	0.57235000	-1.94741600	1.10057900
O	0.68704700	-4.53532800	-2.52911600
O	0.54776300	-3.21631400	-5.00347600
K	0.63522500	-5.67244100	-4.72475700

4-Chlorobenzonitrile

0 1

C	0.57953000	-1.21996100	0.00000700
C	-0.81336300	-1.21928200	-0.00002600
C	-1.51452300	-0.00004300	0.00000200
C	-0.81336000	1.21924900	-0.00001000
C	0.57949600	1.21994000	-0.00000600
C	1.26034800	-0.00002400	0.00002700
H	1.12692100	-2.15623000	0.00002100
H	-1.35406300	-2.15990200	-0.00002000
H	-1.35412500	2.15983100	-0.00000600
H	1.12692900	2.15618400	0.00000100
C	-2.94820400	-0.00000200	0.00001200

N	-4.11292300	0.00005200	0.00000200
Cl	3.01560300	0.00002900	-0.00000300

Chlorobenzene

0 1

C	0.17802600	-1.21894300	-0.00001200
C	1.57687400	-1.20995700	0.00002700
C	2.27813000	0.00000300	-0.00001700
C	1.57685900	1.20996300	0.00000800
C	0.17802300	1.21894400	0.00000700
C	-0.50150900	-0.00000900	-0.00004000
H	-0.37164800	-2.15473200	0.00000000
H	2.11410400	-2.15444200	0.00003200
H	2.11410000	2.15444400	0.00001100
H	-0.37168100	2.15471400	0.00002300
Cl	-2.26869100	0.00000000	0.00000600
H	3.36445200	0.00001800	-0.00001600

4-CyanoMesitylBenzene radical anion (Pdt$^-$)

-1 2

C	-2.74820900	0.84621900	-0.88988900
C	-1.36907600	0.84024400	-0.87517600
C	-0.60719900	0.00166700	-0.00051000
C	-1.36491700	-0.83848300	0.87635000
C	-2.74395400	-0.84665000	0.89563000
C	-3.50571800	-0.00080900	0.00413000
H	-3.27963300	1.48784500	-1.58966400
H	-0.84366800	1.48075100	-1.58072400
H	-0.83599700	-1.47824600	1.58000200
H	-3.27206300	-1.48912200	1.59714200
C	-4.90038600	-0.00200200	0.00635300
N	-6.08768600	-0.00291700	0.00827100
C	0.87271400	0.00160000	-0.00266200
C	1.61149700	1.21064300	0.16722500
C	1.60974800	-1.20796800	-0.17899300
C	3.01210600	1.18455200	0.15655300
C	3.01021400	-1.18338400	-0.17398500
C	3.74084000	-0.00019700	-0.00648200
H	3.55158100	2.12054500	0.30088500
H	3.54846900	-2.11840800	-0.32919400
C	0.92218200	-2.53368700	-0.43824100

H	0.52958700	-2.99250400	0.47841000
H	0.07018900	-2.41623600	-1.11738300
H	1.62367400	-3.24839800	-0.88283400
C	5.25300800	-0.00685900	0.02719900
H	5.63363300	-0.15342800	1.04837800
H	5.66385900	-0.81622100	-0.58783200
H	5.66754200	0.94002100	-0.33794300
C	0.92671000	2.53955300	0.41749900
H	0.53627000	2.99393900	-0.50231400
H	0.07376800	2.42794900	1.09636900
H	1.62921700	3.25529600	0.85879900

Int2

-1 2

C	-0.62911400	-0.22268600	-1.21679600
C	0.11857600	-0.27929800	0.00009600
C	-0.62906900	-0.22150500	1.21695700
C	-2.00527200	-0.11957900	1.23495300
C	-2.76421400	-0.06404200	0.00005000
C	-2.00531600	-0.12074600	-1.23482600
H	-0.09304200	-0.26597200	-2.16438000
H	-0.09296600	-0.26387800	2.16456400
H	-2.53517900	-0.08304500	2.18442300

H	-2.53526100	-0.08511700	-2.18430900
C	1.59111800	-0.39559400	0.00013700
C	2.41644100	-1.51654900	0.00022400
C	3.77232900	-1.07707400	0.00047200
H	2.06447100	-2.54143900	0.00034600
C	3.74469400	0.30647400	0.00009100
H	4.66231600	-1.69458000	0.00077200
H	4.54738200	1.03205400	0.00001700
N	2.43058000	0.71494200	-0.00023000
C	1.99001300	2.10381000	-0.00083200
H	1.38996900	2.32157600	-0.88975300
H	1.39010600	2.32240600	0.88797800
H	2.87081200	2.74933300	-0.00120300
C	-4.15241700	0.03987100	0.00002300
N	-5.33846900	0.12936700	-0.00002000

Int1

0 2

C	1.57953200	-0.19704800	0.66083200
C	2.18774100	-1.55153800	0.36510300
C	3.30054400	-1.37421400	-0.43940600
C	3.44041200	-0.01747600	-0.73191700
N	2.37259700	0.69724000	-0.20314000

H	1.74328200	0.08388900	1.72338100
H	1.79080800	-2.47680700	0.76028300
H	3.95248800	-2.15507300	-0.81198300
H	4.18343600	0.48542300	-1.33654900
C	2.45957100	2.10455900	0.12628000
H	3.01318500	2.62859000	-0.65857700
H	1.45446700	2.53552000	0.17971800
H	2.96687600	2.28450700	1.08928500
C	0.07899700	-0.10609300	0.39760000
C	-0.82759800	-0.07901800	1.46271900
C	-0.41448700	-0.08984300	-0.91441800
C	-2.20092600	-0.04315600	1.23426800
H	-0.45504100	-0.08410600	2.48447700
C	-1.78143500	-0.04603300	-1.15898900
H	0.28784800	-0.10535900	-1.74209500
C	-2.68677500	-0.02497600	-0.08224200
H	-2.89892100	-0.02139200	2.06516800
H	-2.15986200	-0.02955800	-2.17621800
C	-4.09852700	0.02008800	-0.32863800
N	-5.24414900	0.05745300	-0.52886700

TS1

0 2

C	2.24249300	0.41051000	0.86138100
C	2.63008300	-0.87147300	1.27533300
C	3.21527500	-1.51526000	0.16519900
C	3.22791700	-0.60304400	-0.87997100
N	2.68428600	0.57775900	-0.44053500
H	2.00496600	1.27619500	1.46335700
H	2.47714800	-1.28060400	2.26453100
H	3.60313800	-2.52419100	0.12583600
H	3.57609500	-0.70254700	-1.89872900
C	-0.10075700	0.18674600	0.54196300
C	-0.98346400	1.14344000	1.02013100
C	-0.51368000	-0.90614700	-0.20463100
C	-2.34531600	1.00594800	0.73592900
H	-0.64158800	1.99267400	1.60942800
C	-1.87232300	-1.04728500	-0.49576100
H	0.20245700	-1.64610200	-0.55436600
C	-2.79028700	-0.08981200	-0.02483600
H	-3.06515400	1.73622500	1.09520300
H	-2.23190700	-1.89038900	-1.07918200
C	2.39668400	1.73614800	-1.26551200
H	2.61078100	2.65380400	-0.70914200

H	3.03375900	1.71329700	-2.15262100
H	1.34512900	1.74718700	-1.57508100
C	-4.18597600	-0.23212500	-0.32172800
N	-5.31838700	-0.34653500	-0.56435300

^{t}BuOH

0 1

O	0.01325900	-0.00017600	1.45215900
H	0.94382400	0.00188400	1.72867200
C	-0.00533400	-0.00001600	0.01400900
C	0.69430700	-1.26338700	-0.50999500
H	0.65905500	-1.31910400	-1.60428400
H	1.75131900	-1.27513800	-0.21193800
H	0.21574400	-2.15905100	-0.10037200
C	-1.49012900	-0.00419400	-0.35745000
H	-1.98611800	0.88091600	0.05430700
H	-1.62211800	-0.00462500	-1.44478600
H	-1.98114600	-0.89201700	0.05438900
C	0.68697600	1.26752000	-0.50959700
H	0.65116800	1.32353100	-1.60385100
H	0.20331300	2.16021000	-0.09948100
H	1.74396800	1.28526900	-0.21172500

^{t}BuO^{-}

-1 1

O	0.00002100	-0.00001500	1.48405900
C	-0.00001900	-0.00001400	0.15973500
C	0.98745900	-1.07958200	-0.43683100
H	1.03488000	-1.12999600	-1.54099900
H	1.99851200	-0.87484300	-0.05674500
H	0.69248300	-2.06872400	-0.05840000
C	-1.42876900	-0.31528600	-0.43680100
H	-2.13792600	0.43442000	-0.05777900
H	-1.49634500	-0.33050300	-1.54097200
H	-1.75680100	-1.29353000	-0.05727100
C	0.44130800	1.39489000	-0.43680800
H	0.46209000	1.46086000	-1.54097700
H	1.44505800	1.63430400	-0.05761000
H	-0.24199100	2.16808900	-0.05749100

TS2

-1 2

C	0.41840600	1.06216600	-0.18479600
C	0.84987100	1.82173900	-1.37429400
C	1.67660300	2.87338200	-0.97589700
C	1.72593900	2.89024800	0.41332200

N	0.88227600	1.92588600	0.93116000
H	1.11242200	-0.00459500	-0.01659700
H	0.63825000	1.51248800	-2.39089000
H	2.20026300	3.56877000	-1.62358600
H	2.30545000	3.51870600	1.07840100
C	1.06318500	1.37445800	2.26194700
H	1.50663800	2.14161000	2.90753700
H	0.09019900	1.09392000	2.68487300
H	1.69986000	0.48017200	2.23008100
C	-1.00015100	0.57127100	-0.09512500
C	-1.31955100	-0.56486900	0.68564100
C	-2.03681100	1.14714400	-0.86303200
C	-2.61017400	-1.06783500	0.73214800
H	-0.50902400	-1.07673800	1.19731700
C	-3.33122500	0.65224800	-0.82895200
H	-1.80365600	2.01405000	-1.47647600
C	-3.64595000	-0.46477400	-0.02256500
H	-2.83592000	-1.94211800	1.33718000
H	-4.11894400	1.12840300	-1.40794200
O	1.74630300	-1.10417900	0.57052900
C	2.73221500	-1.70409600	-0.19662700
C	3.21369800	-2.97154800	0.55056000
H	4.00902100	-3.51069100	0.01319000

H	3.59176600	-2.69084500	1.54135100
H	2.36813500	-3.65444200	0.69796900
C	3.93792500	-0.74924500	-0.39676400
H	3.60882200	0.16539600	-0.90366900
H	4.34237900	-0.46056400	0.58153000
H	4.75000400	-1.20122900	-0.98722800
C	2.18118800	-2.12013700	-1.58530000
H	1.30393200	-2.76445200	-1.45186600
H	1.86227900	-1.23168800	-2.14378200
H	2.92141400	-2.65931400	-2.19593400
C	-4.97434100	-0.97616600	0.01908100
N	-6.06780200	-1.38836100	0.05858700

TS1(Fur)

0 2

C	-2.47368700	-0.52973800	0.77562400
C	-3.40339500	1.06870500	-0.42507500
C	-3.48184000	-0.06746500	-1.18272100
H	-2.19835000	-0.94823300	1.73050400
H	-3.69557200	2.09435300	-0.58939400
H	-3.89653700	-0.14255100	-2.17792400
C	-0.10726300	-0.29666100	0.40663800

C	0.38641900	0.99638700	0.33474000
C	0.68024000	-1.42941200	0.26931500
C	1.75636100	1.17094400	0.10886300
H	-0.26086300	1.86082000	0.45248300
C	2.05111700	-1.26052200	0.04709700
H	0.26003700	-2.42977400	0.32833600
C	2.58518400	0.04078500	-0.03348500
H	2.18303900	2.16724900	0.04583100
H	2.70267100	-2.12178100	-0.06384900
C	3.98908400	0.21710800	-0.26426800
N	5.13008300	0.36043700	-0.45197600
O	-2.82614200	0.79738700	0.78487300
C	-2.91403300	-1.11460700	-0.39888700
H	-2.80499600	-2.15538500	-0.66818900

TS2(Fur)

-1 2

C	-0.40002600	1.01518700	-0.11070200
C	-1.47341800	2.91700000	0.58973900
C	-1.59783000	2.89714300	-0.78969500
H	-1.05481400	-0.05151400	0.05369000
H	-1.84406700	3.59832300	1.34185300

H	-2.13435500	3.63539100	-1.37320200
C	1.01970400	0.57404000	-0.07297000
C	1.61424200	-0.00002300	-1.21990800
C	1.77822600	0.61052100	1.11696300
C	2.90380600	-0.51148600	-1.18542600
H	1.05775600	-0.04411600	-2.15079000
C	3.07175800	0.10487000	1.16291100
H	1.34421000	1.04867400	2.00835800
C	3.65057400	-0.46626200	0.01118000
H	3.34322300	-0.94232000	-2.07950700
H	3.64164800	0.15117400	2.08563100
O	-1.64144000	-1.31081300	0.34006900
C	-3.01528400	-1.47680700	0.10711200
C	-3.45334200	-2.85648200	0.65055300
H	-4.52255900	-3.04613400	0.48536100
H	-2.88349500	-3.65275800	0.15667500
H	-3.25719600	-2.91732800	1.72775500
C	-3.32373100	-1.42114900	-1.41023900
H	-3.01736500	-0.45288500	-1.82251800
H	-2.76412900	-2.20572200	-1.93414700
H	-4.39183800	-1.56193900	-1.62362400
C	-3.83587400	-0.37716300	0.82567500
H	-3.64442600	-0.41126700	1.90517800

H	-3.54406300	0.61444900	0.46197500
H	-4.91579700	-0.49712700	0.66650200
C	4.97943000	-0.98602700	0.05261900
N	6.06666300	-1.41003500	0.08619500
O	-0.73273300	1.85482000	1.04379100
C	-0.92287600	1.77691400	-1.27445000
H	-0.85812800	1.45163200	-2.30359500

TS1(Thio)

0 2

C	-2.12325300	0.90721300	-0.44152500
C	-3.47625500	-0.93822800	0.57034500
C	-3.16844900	0.05123000	1.47652500
H	-1.81079100	1.65002200	-1.16190500
H	-4.00595500	-1.86502600	0.74507200
H	-3.45925600	0.00929000	2.52013900
C	0.19794700	0.39888700	-0.32308300
C	1.07174100	1.47412600	-0.37662100
C	0.60247300	-0.90721800	-0.09689700
C	2.43815200	1.22853900	-0.20332200
H	0.72296300	2.48891000	-0.54937400
C	1.96701400	-1.15839700	0.07979400

H	-0.11020400	-1.72674400	-0.06281900
C	2.88222700	-0.08855400	0.02527700
H	3.15457600	2.04323700	-0.24372800
H	2.32406000	-2.16832800	0.25626800
C	4.28137900	-0.34358900	0.20508400
N	5.41864100	-0.55080800	0.35134800
S	-2.87694600	-0.56656400	-1.01814000
C	-2.43866300	1.12317800	0.89880900
H	-2.12063100	2.00619400	1.44083300

TS2(Thio)

-1 2

C	0.35673600	0.94373600	0.20154200
C	1.79856900	3.04145800	-0.40811100
C	1.59359800	2.89204000	0.96450200
H	0.99018200	-0.12256700	0.26519600
H	2.39475700	3.79085200	-0.91185000
H	2.04008700	3.56821200	1.68827900
C	-1.07275000	0.50831600	0.12422900
C	-1.92424000	0.56382100	1.24833200
C	-1.59315300	-0.06377200	-1.05855900
C	-3.22462200	0.07287800	1.20131600

H	-1.56975600	1.00549400	2.17257700
C	-2.89079300	-0.55231300	-1.12362300
H	-0.96820600	-0.12880600	-1.94396700
C	-3.72359300	-0.49197800	0.01236900
H	-3.86014500	0.13349200	2.07901200
H	-3.26570400	-0.98247100	-2.04672900
O	1.52830000	-1.46367300	0.45357200
C	2.84995500	-1.78313500	0.11551200
C	3.27910900	-3.05698100	0.88362600
H	4.30837600	-3.35853100	0.64623400
H	3.21371200	-2.88441200	1.96478600
H	2.61209100	-3.89046000	0.63235900
C	3.81669000	-0.63274300	0.49286200
H	3.54024700	0.28533900	-0.03729400
H	3.76012000	-0.43211000	1.56961700
H	4.85926200	-0.87210700	0.24445400
C	2.97503700	-2.06075000	-1.40444500
H	2.30772500	-2.88324600	-1.68973200
H	2.68192100	-1.17249000	-1.97638800
H	3.99815100	-2.33325900	-1.69722300
C	-5.06137700	-0.99096100	-0.04511600
N	-6.15398600	-1.39763500	-0.09250400
S	0.96213400	1.81073100	-1.34071700

C	0.79276600	1.81795800	1.32200200
H	0.58294800	1.55275800	2.35246200

TS1(Ben)

0 2

C	0.12142000	-0.45487900	-0.39356100
C	0.49774700	0.87342700	-0.26633600
C	1.01562500	-1.51239200	-0.31163800
C	1.84739600	1.16563800	-0.04751700
H	-0.22980300	1.67695100	-0.33874600
C	2.36753300	-1.22669100	-0.09534900
H	0.69123400	-2.54498200	-0.41091000
C	2.78038300	0.11343200	0.03684700
H	2.17979600	2.19394900	0.05552100
H	3.09692800	-2.02807600	-0.02935800
C	4.16532200	0.41004500	0.25847300
N	5.29094700	0.65115000	0.43859000
C	-2.05046900	-0.95929300	-0.61638900
C	-2.42090100	-1.15326000	0.74450000
C	-3.14045400	-0.17417300	1.42171100
C	-3.56583600	0.98365900	0.75053100
C	-3.28707500	1.14258700	-0.61755600
C	-2.56886500	0.17078600	-1.30604500

H	-2.10791200	-2.05664600	1.25928200
H	-3.38997500	-0.31384100	2.46974600
H	-4.13093800	1.74377100	1.28165200
H	-3.65191100	2.02000500	-1.14421000
H	-2.36954300	0.28591300	-2.36731400
H	-1.69545200	-1.80842100	-1.19182300

<u>TS2(Ben)</u>

-1 2

C	1.03643700	0.52078800	-0.06021900
C	1.55227800	-0.31553100	-1.07219000
C	1.90144800	0.89129600	0.98599700
C	2.86797600	-0.76031600	-1.04930700
H	0.90131000	-0.63409500	-1.87991400
C	3.22134000	0.44777200	1.03181800
H	1.54153700	1.54654900	1.77231700
C	3.71692600	-0.38281800	0.01122300
H	3.24248700	-1.40472600	-1.83809300
H	3.87075100	0.74895400	1.84767900
C	5.07166500	-0.84081400	0.04785600
N	6.17638200	-1.21383100	0.07643800
C	-0.40944600	0.97692300	-0.12538800
C	-0.95799200	1.57902000	1.12483600

C	-1.82202500	2.64781200	1.11515000
C	-2.16914200	3.31257800	-0.08980000
C	-1.57302500	2.87580600	-1.30315500
C	-0.70935500	1.80858100	-1.33627300
H	-2.24360300	2.99861600	2.05528000
H	-1.78953200	3.41165400	-2.22538100
H	-1.00758400	-0.06272400	-0.30491800
O	-1.67266700	-1.42262700	-0.73344200
C	-2.70433600	-1.93873500	0.05376200
C	-3.17402500	-3.29435400	-0.53024500
H	-3.53078800	-3.15743900	-1.55841900
H	-2.33718900	-4.00313200	-0.55341800
H	-3.98713000	-3.74289200	0.05723500
C	-2.22421400	-2.17600300	1.50982100
H	-1.37430200	-2.86959800	1.51393900
H	-1.89332600	-1.23171500	1.95695200
H	-3.01299200	-2.59751000	2.14788600
C	-3.91704000	-0.97212500	0.08330900
H	-4.28549300	-0.80648700	-0.93668800
H	-4.74849200	-1.35958200	0.68811800
H	-3.61507500	-0.00315800	0.49668400
H	-0.72777600	1.09055500	2.06870300
H	-2.84479000	4.16191700	-0.07926000

H	-0.25617700	1.51336700	-2.27940700

TS1(Bfu)

0 2

C	1.09219100	1.58526800	0.97542500
C	2.45312200	-0.12375500	0.67313600
C	2.52185300	0.66079300	-0.49835100
H	0.55697300	2.25719900	1.62766900
C	-1.10779900	0.74263600	0.41313000
C	-1.40546600	-0.52469500	0.88594500
C	-1.98366900	1.52131600	-0.32558600
C	-2.66990100	-1.05252200	0.60100600
H	-0.68977200	-1.10089800	1.46507500
C	-3.25002700	0.99802400	-0.60894300
H	-1.71178200	2.51064500	-0.68274000
C	-3.58850200	-0.28843700	-0.14502200
H	-2.94619700	-2.04197600	0.95195500
H	-3.96935200	1.57440200	-1.18238000
C	-4.88474700	-0.82727700	-0.43666200
N	-5.93808100	-1.26518300	-0.67359000
O	1.60759600	0.45842900	1.58562800
C	1.65803800	1.78312000	-0.26522200

C	3.15540300	-1.30919100	0.85248000
C	3.97123400	-1.71324500	-0.20863600
H	4.54346300	-2.63129800	-0.11781500
C	4.06583700	-0.95253700	-1.39342000
H	4.71033300	-1.30081600	-2.19484300
C	3.34936300	0.23174100	-1.55365300
H	3.42653500	0.81056500	-2.46887600
H	3.07525800	-1.88588200	1.76769800
H	1.44875700	2.60948000	-0.92939500

TS2(Bfu)

-1 2

C	-0.29719300	-0.30273600	0.35678700
C	-2.19607500	-1.18199700	-0.57562200
C	-2.26130700	-1.49569600	0.81641800
H	-0.38033500	0.91278200	0.56561600
C	1.15482600	-0.61379500	0.19159000
C	2.00293100	-0.59259800	1.31893500
C	1.72941200	-0.85442400	-1.07083400
C	3.36799800	-0.81380100	1.19697800
H	1.58798600	-0.39905700	2.30317600
C	3.09648000	-1.07734000	-1.20657900
H	1.09489100	-0.87301800	-1.94893600

C	3.93005700	-1.05943000	-0.07285700
H	4.00407500	-0.79931500	2.07596900
H	3.52196900	-1.26867300	-2.18647600
O	-0.52613000	2.30251300	0.99705400
C	-0.61670100	3.31684300	0.03473600
C	-0.64048600	4.68984100	0.74756400
H	-0.71477700	5.52429500	0.03714500
H	-1.49705100	4.74387700	1.42998400
H	0.27333000	4.82352600	1.33879700
C	-1.91491100	3.16788000	-0.79737400
H	-1.92689100	2.19645000	-1.30413900
H	-2.78922300	3.21674500	-0.13703500
H	-2.01624500	3.95444400	-1.55730700
C	0.59957500	3.28005900	-0.92475600
H	1.53014900	3.39435600	-0.35566600
H	0.64107600	2.31918500	-1.45045700
H	0.55850500	4.07857100	-1.67745900
C	5.33462800	-1.28961400	-0.20672500
N	6.48066100	-1.47831600	-0.31569300
O	-1.03601800	-0.54276400	-0.88754000
C	-1.07603800	-1.01826800	1.39703200
C	-3.20079400	-1.50591200	-1.47148600
C	-4.33056000	-2.18256100	-0.96449800

H	-5.13686800	-2.45225300	-1.63996400
C	-4.42468500	-2.50959300	0.40033200
H	-5.30764500	-3.02935900	0.76230000
C	-3.40827700	-2.17652200	1.29875100
H	-3.49211600	-2.43223700	2.35112000
H	-3.11756500	-1.25097000	-2.52339100
H	-0.79399500	-1.07114900	2.44011100

TS1(Xyl)

0 2

C	-0.55467600	0.37504500	0.46865300
C	-0.89033900	0.05932900	-0.83953700
C	-1.47999500	0.41845800	1.50148400
C	-2.22716100	-0.21943800	-1.13805600
H	-0.13943800	0.03262000	-1.62447700
C	-2.81996300	0.14140900	1.21022900
H	-1.18898900	0.66307200	2.51982300
C	-3.19063900	-0.17742500	-0.11050900
H	-2.52722700	-0.46625700	-2.15180300
H	-3.57231800	0.17125900	1.99240100
C	-4.56247000	-0.46269900	-0.41289100
N	-5.67764300	-0.69457800	-0.65870700
C	1.63425100	0.65060800	0.98902900

C	2.05460100	-0.70205600	1.09797900
C	2.78203500	-1.32123300	0.08293900
C	3.15125300	-0.54243100	-1.03153600
C	2.81924000	0.81795600	-1.10826300
C	2.09279900	1.44945200	-0.09808100
H	3.15525900	1.39826600	-1.96437900
H	1.26267600	1.15022300	1.87943900
C	1.75652200	2.91753700	-0.15597100
H	2.06323000	3.43283400	0.76180000
H	2.24894500	3.40605700	-1.00129400
H	0.67467300	3.06921600	-0.26069100
H	3.72600900	-0.99633400	-1.83521100
H	1.77077900	-1.26904500	1.98087000
C	3.17484200	-2.77849700	0.16998600
H	2.60551000	-3.38352200	-0.54624200
H	4.23617600	-2.92045600	-0.06090600
H	2.98640600	-3.18197600	1.16889400

TS2(Xyl)

-1 2

C	-1.23075600	-0.23501300	0.06024400
C	-1.66149600	0.85030200	-0.72891300
C	-2.21815300	-1.00273900	0.71098200

C	-3.01095900	1.16081800	-0.86599500
H	-0.91253600	1.46615000	-1.21638600
C	-3.57196200	-0.70262900	0.59527500
H	-1.91739100	-1.85937000	1.30690600
C	-3.98056500	0.38578900	-0.19909800
H	-3.32081000	2.00293500	-1.47682600
H	-4.31451200	-1.30970700	1.10381100
C	-5.36967700	0.70104400	-0.32830500
N	-6.50268600	0.95741400	-0.43565100
C	0.24267100	-0.59014500	0.14459900
C	0.72378500	-1.15355400	1.44137700
C	1.65289300	-2.17529800	1.44559700
C	2.11588900	-2.80432600	0.26356100
C	1.59152200	-2.40024400	-0.99883600
C	0.67081200	-1.37953300	-1.04576800
H	2.05146200	-2.51066600	2.40318100
H	0.83002700	0.56329000	0.00633700
C	0.31911500	-0.48287200	2.73111900
H	0.54985500	0.59108200	2.70329300
H	-0.75625200	-0.55940300	2.93498900
H	0.84960000	-0.91963400	3.58378400
O	1.27681800	1.85929000	-0.24090500
C	2.65431500	2.11722400	-0.11971500

C	3.15123700	1.82343100	1.31662600
H	2.60107000	2.43958800	2.03793800
H	2.98080400	0.77165000	1.56999100
H	4.22231200	2.03613200	1.43244100
C	3.46822500	1.25733000	-1.11710000
H	3.30047200	0.19224800	-0.92532100
H	3.14723300	1.46938800	-2.14441500
H	4.54600300	1.45617200	-1.04801100
C	2.90379600	3.61024700	-0.43283100
H	2.33984300	4.24051300	0.26505200
H	3.96665100	3.87573500	-0.35315600
H	2.56792500	3.84536600	-1.44990500
H	2.83736600	-3.61515700	0.32244100
H	0.25863200	-1.08206900	-2.00911800
C	2.04425200	-3.10886000	-2.25789000
H	1.85700300	-4.18847400	-2.19845900
H	1.52697800	-2.72435800	-3.14242800
H	3.12286200	-2.98344400	-2.41892500

TS1(OMe)

0 2

C	2.31197600	0.42498000	0.89461400
C	2.75571300	-0.83930900	1.32978100

C	3.48660800	-1.41899800	0.26958300
C	3.53234800	-0.48367400	-0.76020900
N	2.86913000	0.64750600	-0.35489300
H	1.97912300	1.26358100	1.48931200
H	2.53202100	-1.28063400	2.29134700
H	3.93992700	-2.40086400	0.24796800
H	3.98403700	-0.53791600	-1.74059100
C	0.05492700	0.09342800	0.42346800
C	-0.89071900	0.91588400	1.01085800
C	-0.30894600	-0.91890400	-0.45795700
C	-2.25552100	0.73693200	0.70968500
H	-0.60577900	1.70661700	1.70292600
C	-1.66036200	-1.10232100	-0.76803200
H	0.43833300	-1.56897000	-0.90758900
C	-2.63472100	-0.27439700	-0.18314600
H	-2.99283800	1.38419500	1.17120600
H	-1.97807600	-1.88152400	-1.45584200
C	2.61089000	1.81949800	-1.17741000
H	2.69190700	2.72408800	-0.56988900
H	3.35109400	1.86680200	-1.97766100
H	1.60773000	1.77461900	-1.61508800
O	-3.93231200	-0.53631700	-0.54874700
C	-4.97186200	0.26759500	0.01071200

H	-4.85005400	1.32193500	-0.26335300
H	-5.90196900	-0.11236900	-0.41262800
H	-5.00285400	0.17414900	1.10243100

TS1(Ph)

0 2

C	1.39805600	0.35011900	0.94318500
C	1.82765700	-0.94251900	1.30503400
C	2.61131900	-1.44099000	0.24148900
C	2.70227300	-0.43181200	-0.71299500
N	2.01496500	0.66548900	-0.25780700
H	1.03889000	1.14314800	1.58357300
H	1.56055700	-1.45419700	2.21950200
H	3.07016900	-2.41787200	0.16943400
H	3.20111900	-0.41262000	-1.67160500
C	-0.82515800	0.05626300	0.34388100
C	-1.79226000	0.84171500	0.95584600
C	-1.13789600	-0.89262700	-0.61977800
C	-3.13633700	0.67274100	0.57930300
H	-1.52997000	1.58031600	1.71162600
C	-2.48163000	-1.05401600	-0.99623400
H	-0.36381400	-1.50465200	-1.07777800
C	-3.47705600	-0.27233000	-0.39547000

H	-3.91046800	1.27799700	1.04558900
H	-2.74927900	-1.78885000	-1.75211400
C	1.79066400	1.89435800	-1.00365400
H	1.83204500	2.75141600	-0.32716000
H	2.57163800	2.00440600	-1.75763400
H	0.81259600	1.87826400	-1.49676100
H	-4.51603200	-0.40119100	-0.68566200

TS1(diMe)

0 2

C	1.94756200	-1.03180800	-0.40701800
C	2.38393300	-0.41686000	-1.59844400
C	3.19216200	0.68308200	-1.23563000
C	3.29166600	0.68271900	0.15254800
N	2.58588300	-0.38829300	0.64245300
H	1.57400300	-2.03664100	-0.26883700
H	2.10421900	-0.72486500	-2.59661900
H	3.66106500	1.39698600	-1.89939900
H	3.80790800	1.35658700	0.82148000
C	-0.25451200	-0.36328900	-0.18864800
C	-1.24121200	-1.33258500	-0.20462800
C	-0.53139200	0.99131600	-0.06279500
C	-2.59261200	-0.93803900	-0.08405600

H	-0.99760600	-2.39015000	-0.30511100
C	-1.86848600	1.40665400	0.06863900
H	0.26789900	1.73035200	-0.06406000
C	-2.87839100	0.42656500	0.05233300
C	2.36492400	-0.68639200	2.04900500
H	2.39304500	-1.76713100	2.20811900
H	3.15480500	-0.22379200	2.64289800
H	1.39309100	-0.30430200	2.38024600
H	-3.91644900	0.73990700	0.14775800
C	-2.22067200	2.86874700	0.23554800
H	-1.35102500	3.50892900	0.06151200
H	-3.01080700	3.17123100	-0.46071100
H	-2.58851200	3.07434500	1.24838100
C	-3.69875700	-1.97006000	-0.11541800
H	-4.66671000	-1.52501000	0.13262000
H	-3.78557100	-2.42681500	-1.10886300
H	-3.50581200	-2.78187900	0.59478800

TS1(Ph)

0 2

C	-3.63348800	0.87856500	0.07740800
C	-4.00093000	0.86230000	-1.28305500
C	-4.58115800	-0.39710700	-1.54917100

C	-4.61844700	-1.09700300	-0.34645100
N	-4.09563400	-0.30029300	0.64109500
H	-3.43203600	1.73125400	0.71001000
H	-3.82477900	1.66569800	-1.98512400
H	-4.94233600	-0.76266300	-2.50094900
H	-4.97587200	-2.09194600	-0.12187900
C	-1.32808500	0.57554500	0.03406300
C	-0.50660200	1.52793400	0.62015300
C	-0.81145900	-0.55761900	-0.57786600
C	0.88280500	1.33287900	0.59953400
H	-0.91616200	2.41272100	1.10455600
C	0.57731300	-0.74886800	-0.59288400
H	-1.46347300	-1.28882300	-1.05078300
C	1.44489400	0.19292300	-0.00537300
H	1.53068000	2.06323700	1.07785700
H	0.98995600	-1.62542100	-1.08614400
C	-3.87163400	-0.69846400	2.02252900
H	-4.09039200	0.13854100	2.68995700
H	-4.53652400	-1.52759600	2.26924400
H	-2.83282700	-1.01132100	2.17386700
C	2.91914900	-0.01179300	-0.02515400
C	3.79846500	1.06913200	-0.22498800
C	3.47554700	-1.29196100	0.15680900

C	5.18228800	0.87817300	-0.23995900
H	3.39582000	2.06421800	-0.38990700
C	4.85909400	-1.48473400	0.13865800
H	2.82148500	-2.14060900	0.33427700
C	5.72001000	-0.40023200	-0.05874600
H	5.83953500	1.72778400	-0.40250100
H	5.26410400	-2.48163400	0.28831000
H	6.79568400	-0.54941200	-0.07149100

TS1(Me)

0 2

C	1.90999800	0.39821600	0.88411700
C	2.30881000	-0.87890400	1.32696300
C	3.01459400	-1.49230000	0.26871900
C	3.09280700	-0.56607400	-0.76725300
N	2.47334300	0.59191000	-0.36777900
H	1.61514900	1.25334100	1.47559800
H	2.07121400	-1.30552900	2.29184100
H	3.43094000	-2.49052400	0.25250900
H	3.53999400	-0.64336800	-1.74814600
C	-0.35174300	0.15314000	0.42292600
C	-1.26343200	0.98504500	1.05375200
C	-0.75408000	-0.82345400	-0.47838200

C	-2.63178500	0.83576300	0.76368600
H	-0.94359800	1.74978800	1.75995200
C	-2.12067900	-0.96025800	-0.76366800
H	-0.02978700	-1.47614100	-0.96131500
C	-3.07761300	-0.13540200	-0.14522600
H	-3.35655600	1.48575400	1.25072600
H	-2.44732700	-1.71840600	-1.47369200
C	2.25694100	1.76744400	-1.19708800
H	2.37473100	2.67233400	-0.59598900
H	2.99526400	1.78119100	-2.00035500
H	1.25114700	1.75819500	-1.63106100
C	-4.55066400	-0.30670100	-0.44654200
H	-5.14113700	0.50926100	-0.01992800
H	-4.73759700	-0.33160200	-1.52580200
H	-4.93476900	-1.24675200	-0.03190200

[PLY(O,O)-K]$^{2-}$ (III; Cat^{2-})

-2 1

C	-3.46793200	1.21436800	0.00015100
C	-4.14547400	-0.00000100	0.00021700
C	-1.26622100	0.00000000	-0.00005500
C	-2.01989800	1.24265200	0.00001500
H	-5.23747200	-0.00000100	0.00032600

C	0.10619600	-2.44615500	-0.00005800
C	-1.30091800	2.43816200	-0.00000900
C	0.10619500	2.44615500	-0.00009300
H	0.63705400	-3.39937300	-0.00004100
H	0.63705300	3.39937300	-0.00007300
C	0.89709700	-1.28831500	-0.00019500
C	0.18265200	0.00000000	-0.00017200
C	0.89709700	1.28831600	-0.00017400
C	-2.01989700	-1.24265300	0.00004600
C	-1.30091700	-2.43816300	0.00005300
C	-3.46793200	-1.21436900	0.00018300
H	-1.84566900	-3.38373300	0.00015400
H	-4.00610000	-2.16095200	0.00026000
H	-4.00610100	2.16095100	0.00021000
H	-1.84567000	3.38373300	0.00006000
O	2.21013600	1.40049100	-0.00011300
O	2.21013600	-1.40048900	-0.00021100
K	4.26865500	-0.00000100	0.00011800

26. References:

1. R. C. Haddon, R. Rayford, & A. M. Hirani, *J. Org. Chem.* 1981, **46**, 4587-4588.

2. S. De, S. Mishra, B. N. Kakde, D. Dey, & A. Bisai, *J. Org. Chem.* 2013, **78**, 7823-7844.

3. M. J. Frisch, *et al.* Gaussian 16, Revision B.01, Fox, Gaussian, Inc., Wallingford CT, 2016.

4. A. D. Becke, *J. Chem. Phys*. 1993, **98**, 5648−5652.

5. Y. Zhao, & D. G. Truhlar, *Theor. Chem. Acc.*, 2008, **120**, 215−241.

6. D. Geuenich, K. Hess, F. Koehler, & R. Herges, *Chem. Rev.* 2005, **105**, 3758-3772.

7. R. Herges, & D. Geuenich, *J. Phys. Chem.* 2001, **A 105**, 3214-3220.

8. H. Wei, Y. Liu, T. Y. Gopalakrishna, H. Phan, X. Huang, L. Bao, J. Guo, , J. Zhou, S. Luo, J. Wu, & Z. Zeng, *J. Am. Chem. Soc*. 2017, **139**, 15760-15767.

9. Z. Chen, C. S. Wannere, , C. Corminboeuf, R. Puchta, & P. R. Schleyer, *Chem. Rev.* 2005, **105**, 3842-3888.

10. O. V. Dolomanov, L. J. Bourhis, R. J. Gildea, J. A. K. Howard, & H. Puschmann, *J. Appl. Cryst*. 2009, **42**, 339–341.

11. G. M. Sheldrick, *Acta Cryst*. 2015, **A71**, 3–8.

12. D. T. Gryko, O. Vakuliuk, D. Gryko, & B. Koszarna, *J. Org. Chem.* 2009, **74**, 9517-9520.

13. S. Crespi, S. Protti, & M. Fagnoni, *J. Org. Chem.* 2016, **81**, 9612-9619.

14. S. Paul, K. K. Das, S. Manna, & S. Panda, *Chem. Eur. J.* 2020, **26**, 1922–1927.

15. M, Wang, X. Yuan, H. Li, L. Ren, Z. Sun, Y. Hou, & W. Chu, *Catal. Commun.*, 2015, **58**, 154-157.

16. N. G. W. Cowper, C. P. Chernowsky, O. P. Williams, & Z. K. Wickens, *J. Am. Chem. Soc.*, 2020, **142**. 2093-2098.

17. J. R. Naber, B. P. Fors, X. Wu, J. T. Gunn, & S. L. Buchwald *HETEROCYCLES*, 2010, **80**, 1215 – 1226.

18. F. Yu, R. Mao, M. Yu, X. Gu, & Y. Wang, *J. Org. Chem.* 2019, **84**, 9946−9956.

19. S. Zhang, Z. Tang, W. Bao, J. Li, B. Guo, S. Huang, Y. Zhang & Y. Rao, *Org. Biomol. Chem.*, 2019, **17**, 4364–4369.

20. V. Hornillos, M. Giannerini, C. Vila, M. Fañanás-Mastral, & B. L. Fering, *Org. Lett.* 2013, **15**, 5114-5120.

21. X. Li, F. Feng, C. Ren, Y. Teng, Q. Hu, & Z. Yuan, *Synlett*, 2019, **30**, 2131-2135.

22. J. Cao, Z-L. Chen, S-M. Li, G-F. Zhu, Y-Y. Yang, C. Wang, W-Z. Chen, J-T. Wang, J-Q. Zhang, & L. Tang, *Eur. J. Org. Chem.*, 2018, **22**, 2774-2779.

23. T. Wang, S. Shi, M. H. Vilhelmsen, T. Zhang, M. Rudolph, F. Rominger, A. S. K. Hashmi, *Chem. Eur. J.* 2013, **19**, 12512-12516.

24. S. Tani, T. N. Uehara, J. Yamaguchia, & K. Itami, *Chem. Sci.*, 2014, **5**, 123–135.

25. Prez-Perarnau, *et. al.*, *Angew. Chem. Int. Ed.* 2014, **53**, 10150 –10154.
